# Antioxidant Activity and Metabolomic Characterization of *Lactiplantibacillus plantarum* MCS1903 Isolated from Naturally Fermented Tofu Whey

**DOI:** 10.3390/microorganisms14061348

**Published:** 2026-06-16

**Authors:** Yuanchun Yue, Changgang Wang, Xinjian Yang, Dan Yang, Changlu Ma

**Affiliations:** 1 College of Food and Bioengineering, Beijing Vocational College of Agriculture, Beijing 102442, China; yueyuanchungreat@163.com (Y.Y.); 15910470913@163.com (C.W.); 71937@bvca.edu.cn (X.Y.); 2 Food Science and Engineering, Beijing University of Agriculture, Beijing 102206, China; 90333@bvca.edu.cn

**Keywords:** tofu whey, *Lactiplantibacillus plantarum*, antioxidant, stress tolerance, metabolomics, Nrf2/Keap1-HO-1 pathway

## Abstract

Naturally fermented tofu whey is a nutrient-rich byproduct of tofu production that harbors diverse lactic acid bacteria (LAB) with potential probiotic properties. However, the antioxidant mechanisms of these LAB, particularly the roles of different cellular fractions and their metabolic basis, remain unclear. This study aimed to isolate LAB from naturally fermented tofu whey and evaluate their antioxidant activities across cellular fractions, combining in vitro assays, 16S rDNA-based identification, metabolomic profiling, and cellular validation to elucidate the underlying mechanisms. Six LAB strains were isolated and screened for 2,2-diphenyl-1-picrylhydrazyl and hydroxyl radical scavenging capacity and environmental stress tolerance. Among the identified isolates, *Lactiplantibacillus plantarum* MCS1903 exhibited the highest extracellular antioxidant activity. Non-targeted metabolomic analysis of cell-free supernatant revealed distinct metabolic profiles compared with the MRS control, with significant enrichment of antioxidant-related metabolites and pathways. In Caco-2 cells, MCS1903 supernatant (<5%, *v*/*v*) showed no significant cytotoxicity and effectively alleviated H_2_O_2_-induced oxidative stress by modulating the Nrf2/Keap1-HO-1 signaling pathway. These findings indicate that tofu whey is a valuable source of functional LAB, and MCS1903 represents a promising candidate for probiotic and functional food applications, supporting the valorization of tofu whey and development of natural antioxidant probiotics derived from fermented food byproducts.

## 1. Introduction

Naturally fermented tofu whey, a traditional byproduct of tofu production, is rich in lactic acid bacteria (LAB) and has long been used as a natural coagulant in artisanal tofu processing [1,2,3]. Unlike chemical coagulants, it contributes to the unique texture and flavor of tofu while harboring a complex microbial community with potential probiotic properties [4]. Among these microorganisms, LAB species have attracted considerable attention for their health-promoting effects, particularly their antioxidant capacity, which is mediated through the production of bioactive compounds including gallic acid, ferulic acid, caffeic acid, and p-coumaric acid, as well as superoxide dismutase [5,6,7,8,9,10]. The Probio-Ichnos database shows that 229 of the 1565 recorded strains of *Lactiplantibacillus plantarum* (*L. plantarum*) exhibit antioxidant activity, highlighting the significant strain specificity of antioxidant properties within this species [11]. In particular, some *L. plantarum* strains enhance the host antioxidant resistance system by regulating antioxidant enzyme expression, thereby reducing cell injury induced by reactive oxygen species (ROS) [12]. The above observations confirm the significant potential of LAB as natural antioxidants. Certain LAB, including *L. plantarum*, can produce low amounts of hydrogen peroxide (H_2_O_2_) when grown under aerobic conditions, mainly through the action of pyruvate oxidase (POX). Nevertheless, these strains are also equipped with a comprehensive ROS-detoxification system, which includes a manganese-containing pseudocatalase (Mn-Kat) and the glutathione–glutaredoxin system, enabling them to efficiently eliminate H_2_O_2_ and preserve redox homeostasis [13]. Thus, the net antioxidant outcome of a given LAB strain is determined by the balance between ROS generation and elimination.

Oxidative stress occurs due to excessive ROS accumulation that overwhelms the cellular antioxidant defense capacity, and exerts a key influence on the progression of various gastrointestinal dysfunctions, such as inflammatory bowel disease (IBD), colorectal carcinoma, and metabolic syndromes [14]. ROS including superoxide anions, H_2_O_2_, and hydroxyl (OH^−^) radicals possess strong chemical activity, which may cause damage to intracellular proteins, lipids and DNA. This damage disrupts the integrity of the gut mucosal epithelial barrier and triggers abnormal inflammatory responses, thereby exacerbating disease progression [15,16]. Cellular stress response is a critical defense mechanism that maintains intracellular homeostasis against harmful stimuli such as oxidative stress, hypoxia, and DNA damage. Recently developed comprehensive databases, including ASTRA and CRESTA, have systematically summarized stress-responsive genes and conserved signaling pathways, providing a standardized framework for investigating the molecular regulatory mechanisms underlying cellular adaptation to stress [17,18]. The cellular antioxidant defense system relies primarily on enzymes, such as superoxide dismutase 1 (SOD1) and catalase (CAT). SOD1 catalyzes the conversion of superoxide anions into H_2_O_2_, which is subsequently decomposed into water and oxygen by CAT, thereby reducing ROS toxicity [19]. The expression of these antioxidant enzymes is tightly regulated by the nuclear factor erythroid 2-related factor 2 (Nrf2)/Kelch-like ECH-associated protein 1 (Keap1) signaling pathway, a central regulator of cellular redox homeostasis. In physiological states, Nrf2 is retained in the cytoplasm by Keap1 and undergoes ubiquitination and degradation [20]. Under oxidative stress conditions, Keap1 alters its conformation and subsequently liberates Nrf2, promoting its nuclear translocation, where it associates with antioxidant response elements and initiates downstream antioxidant-related genes expression, such as heme oxygenase-1 (*HO-1*), *SOD1*, and *CAT*. HO-1 further enhances antioxidant capacity by catalyzing the degradation of heme into bioactive molecules with antioxidant and anti-inflammatory properties [21]. Therefore, identifying natural agents capable of mitigating oxidative stress—either through direct ROS scavenging or via regulation of the Nrf2/Keap1 signaling pathway—holds considerable promise for the prevention and treatment of gastrointestinal disorders. In this context, LAB have emerged as promising candidates owing to their intrinsic antioxidant properties and their ability to modulate host redox homeostasis.

Despite growing interest in LAB-derived antioxidants, several limitations remain in current research. Most studies focus on single antioxidant indicators or isolated cellular fractions, often neglecting systematic comparisons among whole cells, intracellular components, and extracellular metabolites [22]. This limits a comprehensive understanding of the antioxidant mechanisms of LAB. In addition, although tofu whey is widely recognized as a valuable natural source of LAB [1], systematic studies integrating functional screening of antioxidant activity, metabolomic profiling of active strains, and mechanistic validation in relevant cellular models are still scarce. Existing research on tofu whey-derived LAB has primarily focused on their application in tofu gelation and flavor formation, with limited exploration of their antioxidant potential and underlying molecular mechanisms. Furthermore, whether LAB derived from tofu whey exerts antioxidant effects through the Nrf2/Keap1 signaling pathway remains unclear.

In this study, we aimed to systematically evaluate the antioxidant capacity of six LAB isolated from naturally fermented tofu whey and to elucidate their underlying molecular mechanisms. Specifically, the antioxidant capacities of different bacterial fractions, including whole cells, intracellular extracts, and cell-free supernatants (CFS), were comparatively analyzed. Among six LAB isolates, the CFS consistently exhibited higher 2,2-Diphenyl-1-Picrylhydrazyl (DPPH) and hydroxyl radical scavenging activities than whole cells or intracellular extracts, and the CFS of *L. plantarum* MCS1903 showed the strongest activity, leading to its selection for further study. Non-targeted metabolomics was employed to characterize the metabolic profiles associated with antioxidant activity. Furthermore, the protective effects of selected strains were evaluated in intestinal epithelial cells, with particular emphasis on the regulation of the Nrf2/Keap1 signaling pathway. This integrated approach provides a comprehensive framework for understanding the antioxidant mechanisms of LAB. It offers a theoretical and technical basis for the development of natural antioxidant probiotics and the high-value utilization of tofu whey. As far as we are aware, the present investigation is the first of its kind to systematically compare the antioxidant activities of multiple bacterial fractions from tofu whey-derived LAB while integrating metabolomic and cellular analyses to clarify their underlying functional mechanisms.

## 2. Materials and Methods

### 2.1. Isolation and Purification of LAB

Tofu sour paste was collected from a local farmer in Longmen Village, Chicheng County, Zhangjiakou City, Hebei Province, China. The samples were collected in centrifuge tubes. Bacteria were isolated using the gradient plate dilution method. Specimens were gradiently diluted in 0.85% (*w*/*v*) sterile normal saline, followed by spreading on de Man, Rogosa, and Sharpe (MRS, Beijing Land Bridge Technology Co., Ltd., Beijing, China) agar plates. The prepared plates were cultivated at 37 °C for 48 h under anaerobic conditions. Subsequently, the colonies were selected and subjected to repeated streaking on MRS agar plates for purification. This process was performed at least three times until uniform colony morphology was obtained, indicating pure cultures. Single colonies were then cultivated in MRS broth at 37 °C for 24 h. All LAB strains were cultured under the same conditions as described above [23].

### 2.2. Molecular Classification via 16S rDNA Gene Sequencing

Molecular identification was performed using 16S rDNA gene sequencing. Genomic total bacterial DNA was purified via genomic DNA extraction kits, and DNA purity was assessed using micro-ultraviolet spectrophotometry. Universal primers 27F and 1492R were adopted to amplify the 16S rDNA sequence. Polymerase chain reaction (PCR) was conducted and the products were analyzed according to Kang et al. (2020) [24]. Amplified PCR products were verified via agarose gel electrophoresis and then submitted to Novogene Co., Ltd. (Beijing, China) for sequencing. Resulting sequences were aligned against reference sequences in the NCBI database using the NCBI BLAST online tool (https://blast.ncbi.nlm.nih.gov, accessed on 8 April 2026) to identify the strain. Phylogenetic analysis was performed in MEGA 7 with neighbor-joining algorithm, and branch support was assessed using 1000 bootstrap replicates [24].

### 2.3. Preparation of Bacterial Components

#### 2.3.1. Bacterial Suspension

Activated strains were inoculated (3% *v*/*v*) into MRS broth and incubated at 37 °C for 24 h. Then, cells were harvested as described by Bouvard et al. [25], and re-suspended in distilled water to reach final density of roughly 1.0 × 10^9^ CFU/mL (*OD*_600_ = 1.0).

#### 2.3.2. Intracellular Contents

Cell pellets obtained as described above were resuspended in distilled water and disrupted by ultrasonication on ice (power: 200 W; cycle: 3 s on, 6 s off; total duration: 20 min). Centrifugation (6000× *g*, 10 min, 4 °C) was performed to sediment cell debris and recover soluble intracellular components in the supernatant, which was then collected as the cell-free extract [26].

#### 2.3.3. Cell-Free Supernatant

After incubating the activated cultures as described in Section 2.3.1, the fermentation broth was centrifuged (5000× *g*, 15 min, 4 °C) to remove intact cells while avoiding cell disruption. The CFS was prepared according to the previous report [27].

### 2.4. Screening of Strains with Antioxidant Properties

#### 2.4.1. DPPH Radical Scavenging Ability Determination

The free radical scavenging activity of LAB, including whole cells, cell-free extracts, and extracellular secretions, was evaluated using the DPPH assay, following a previously published method with slight modifications [28]. In brief, 1 mL of sample (CFS, cell-free extracts, or bacterial suspension) was mixed with 1.2 mL of a 0.1 mM DPPH solution prepared in absolute ethanol. After thorough vortexing to homogeneity, the mixture was kept in dark at 25 °C for 30 min, and then its absorbance at 517 nm was determined using a microplate reader. A control reaction containing an equal volume of ethanol instead of the sample, and a blank containing ethanol instead of the DPPH solution, were prepared in parallel. Vitamin C (0.5 mM) was used as a positive control.

#### 2.4.2. Hydroxyl (OH^−^) Radical Scavenging Ability Determination

Hydroxyl radical scavenging activity was measured in accordance with the procedure established by Dinh [29] with slight modifications, based on the Fenton reaction principle. The reaction mixture consisted of 1.0 mL of sample (whole cells, cell-free extract, or extracellular secretions), 1.0 mL of 9 mM salicylic acid (in absolute ethanol), 1.0 mL of 9 mM ferrous sulfate (FeSO_4_), and 1.0 mL of 8.8 mM H_2_O_2_. The mixture was vortexed thoroughly and incubated at 37 °C for 30 min in a water bath. After incubation, the absorbance of the mixture was measured at 510 nm against a distilled water blank using a UV–visible spectrophotometer. Equal-volume distilled water replaced samples for control setup, while distilled water was used in place of H_2_O_2_ in blank samples to calibrate background absorbance. Scavenging activity against hydroxyl radicals was quantified according to the formula presented below:

Scavenging activity (%) = [1 − (A_sample_ − A_blank_)/A_control_] × 100%.

### 2.5. Cell Viability Assay

CFS-induced cytotoxicity in Caco-2 cells was evaluated via the CCK-8 assay kit (Solarbio, Beijing, China). Briefly, cells were cultivated in MEM containing 20% fetal bovine serum and 1% penicillin–streptomycin at 37 °C with 5% CO_2_. Then, cells were seeded at a density of 2 × 10^4^ cells per well in 96-well plates. After treatment, 10 μL of CCK-8 reagent was added to each well, and the plates were incubated at 37 °C for 2 h. Absorbance values were determined at 450 nm via a microplate reader. The detailed procedure was described by Yue et al. [27].

### 2.6. Measurement of ROS in Cells

Intracellular ROS levels in Caco2 cells treated with bacterial extracellular secretions were quantified by the fluorescent probe 2′,7′-dichlorodihydrofluorescein diacetate (DCFH-DA, Solarbio, Beijing, China). The 96-well plates were inoculated with 2 × 10^4^ cells per well and incubated overnight at 37 °C. The growth medium was then changed to fresh medium with CFS at concentrations of 0, 0.5, 2.5, 5.0, 8.0, and 12% *v*/*v*. After a pre-incubation for 4 h, oxidative stress was induced by adding H_2_O_2_ (final concentration 1.25 mM) for 8 h. After the treatment, the cells were rinsed with phosphate-buffered saline and then loaded with 10 μM DCFH-DA at 37 °C for 20 min in the dark. Following a final wash, fluorescence intensity was measured by microplate reader under 485 nm excitation and 535 nm emission. Untreated cells and cells treated with H_2_O_2_ alone served as negative and positive controls, respectively [30]. Relative ROS levels were calculated as the percentage of fluorescence intensity compared to the H_2_O_2_-treated control group.

### 2.7. Western Blot Analysis

Western blot analysis was conducted following a previously described protocol [27]. After CFS treatment, Caco-2 cells were lysed, and protein extracts were prepared [31]. Immunoblotting was performed using primary antibodies specific for Nrf2, HO-1, Keap1, SOD1, and CAT (all at 1:1000), followed by HRP-conjugated secondary antibody (1:5000) and ECL detection [27]. Protein band densities were measured via densitometry in ImageJ 1.53 software (National Institutes of Health, Bethesda, MD, USA), with signal intensities normalized against internal reference proteins (β-actin or histone H3).

### 2.8. Metabolomics Analysis of the Chemical Composition of CFS

To characterize the metabolic profiles of LAB strains, metabolomics analysis was performed using an ultra-performance liquid chromatography–triple time-of-flight mass spectrometry (UPLC–TripleTOF) system (ABSCIEX) following previously described protocols [32]. Briefly, samples were separated on a BEH C18 column with a gradient elution and analyzed by electrospray ionization in both positive and negative ion modes. Equal volumes of supernatant from each cohort were pooled to create the quality control (QC) samples, which served to minimize technical data variance. Data analysis was conducted via the Majorbio cloud platform (Shanghai Majorbio Bio-Pharm Technology Co., Ltd., Shanghai, China, https://cloud.majorbio.com) [33]. Multivariate statistical analyses (PCA, PLS-DA) and KEGG pathway enrichment were conducted to identify differential metabolites and significantly enriched pathways. The *p*-values from KEGG pathway enrichment analysis were adjusted for multiple testing using the Benjamini–Hochberg (BH) procedure. Pathways with BH-adjusted *p* < 0.05 were considered statistically significant.

### 2.9. Statistical Analysis

All assays were carried out in triplicate, and results are expressed as mean ± standard deviation (SD). Intergroup differences were analyzed by one-way analysis of variance (ANOVA) using SPSS 20.0 software (SPSS Inc., Chicago, IL, USA), with Tukey’s test used for post hoc comparisons. Statistical significance was defined as *p* < 0.05. Untargeted metabolomics analysis was performed according to Zong et al. [1].

## 3. Results

### 3.1. Isolation and Identification of LAB from Tofu Acid Whey

**Figure 1:**
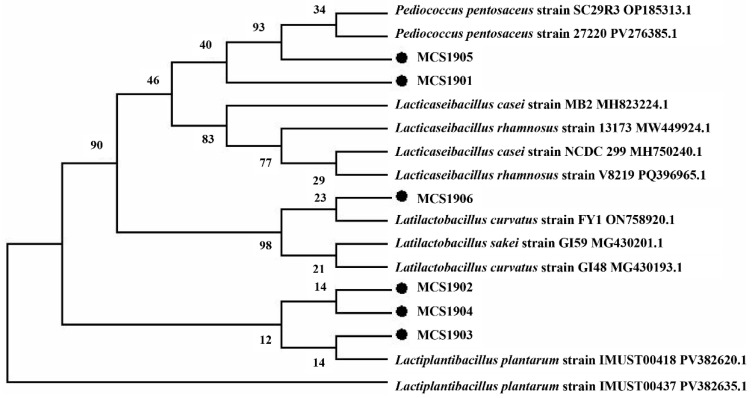
Phylogenetic tree illustrating the relationships among strains MCS1901, MCS1902, MCS1903, MCS1904, and MCS1905, based on 16S rDNA gene sequences. Numbers at branching nodes represent bootstrap values from 1000 replicate analyses.

Based on 16S rDNA sequencing and phylogenetic analysis (Figure 1), strains MCS1902, MCS1903, and MCS1904 showed high sequence identity (>99%) with *L. plantarum* reference strains. MCS1901 exhibited high similarity (≥99%) to *Lacticaseibacillus casei*, while MCS1905 clustered closely with *Pediococcus pentosaceus* reference sequences. Phylogenetic analysis further confirmed the taxonomic classification of all isolates.

### 3.2. Antioxidant Activities of LAB Isolation Components

#### 3.2.1. DPPH Radical Scavenging Activity

Evaluation of the antioxidant activity of LAB fractions using the DPPH radical scavenging assay revealed that the CFS of all LAB strains exhibited significantly higher DPPH radical scavenging activity than their corresponding whole cells and cell-free extracts (*p* < 0.05 for all) (Table 1). Moreover, the CFS of *L*. *plantarum* MCS1903 and MCS1904 exhibited the highest scavenging rates, reaching 71.00 ± 3.58% and 69.7 ± 9.3%, respectively, which were markedly superior to those of other strains, but were comparable with that of vitamin C.

#### 3.2.2. Hydroxyl (OH^−^) Radical Scavenging Ability

Assessment of the OH^−^ radical scavenging activity (Table 2) showed that all fractions exhibited a certain degree of OH^−^ radical scavenging capacity, with CFS demonstrating the strongest effect (27.3–67.5%), followed by the cell-free extracts (6.9–28.8%), and whole cells (3.9–31.6%). Consistent with DPPH results, the CFS of strains MCS1903 and MCS1904 exhibited significantly higher OH^−^ radical scavenging activity than other strains, suggesting that secreted metabolites of these strains contribute substantially to antioxidant activity.

Among the six LAB strains isolated from tofu whey, the CFS of *L*. *plantarum* MCS1903 exhibited the highest DPPH and hydroxyl radical scavenging activities, significantly outperforming its whole cells and intracellular extracts (Table 1 and Table 2). Therefore, only the CFS was used for subsequent metabolomic and cellular analyses.

### 3.3. Analysis of the Metabolomics Difference

**Figure 2:**
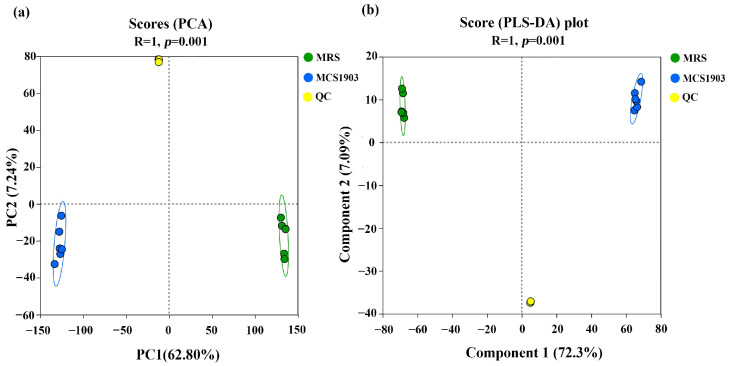
Multivariate statistical analysis of metabolic profiles comparing MCS1903 CFS treatment and the MRS control groups. (**a**) PCA score plot. Green, MRS control group; blue, MCS1903 treatment group; yellow, QC samples. PC1, principal component 1; PC2, principal component 2. (**b**) PLS-DA score plot. Green, MRS control group; blue, MCS1903 treatment group; yellow, QC samples. Component 1, the first latent variable of PLS-DA; Component 2, the second latent variable of PLS-DA.

Non-targeted metabolomic analysis of MCS1903 CFS was performed using liquid chromatography–tandem mass spectrometry. PCA revealed clear separation of the MCS1903 and MRS groups; the first two principal components explained 62.80% and 7.24% of the total variation (Figure 2a). Partial least squares discriminant analysis (PLS-DA) further confirmed distinct metabolic profiles between the two groups (R^2^Y = 0.994, Q^2^ = 0.984, *p* < 0.001; Figure 2b). Permutation testing validated the robustness of the PLS-DA model (Figure S1).

**Figure 3:**
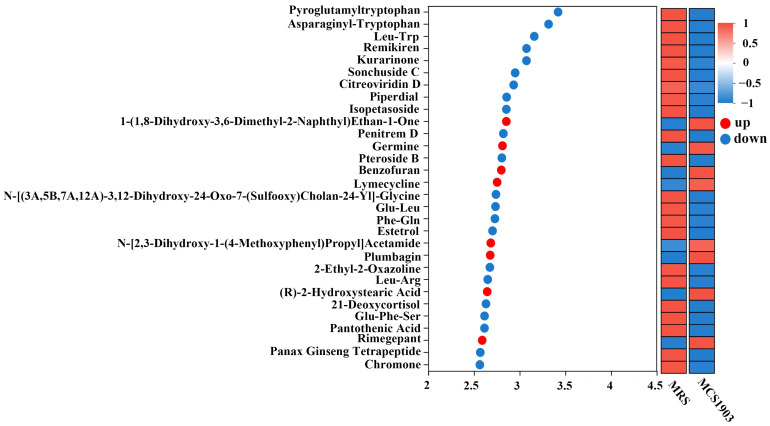
Variable importance value ranking, and expression heatmap of differential metabolites.

In total, 1284 metabolites were identified (Table S1), 503 were significantly increased, and 781 were decreased in the MCS1903-treated group compared to MRS controls (*p* < 0.05, log_2_ fold change [FC] > 1; Figure 3). Variable importance in projection (VIP) analysis identified 30 metabolites with VIP > 2.5 as key discriminators, including pyroglutamyltryptophan, asparaginyl-tryptophan, kurarinone, and pantothenic acid. Hierarchical clustering analysis confirmed consistent metabolic differences between groups.

**Figure 4:**
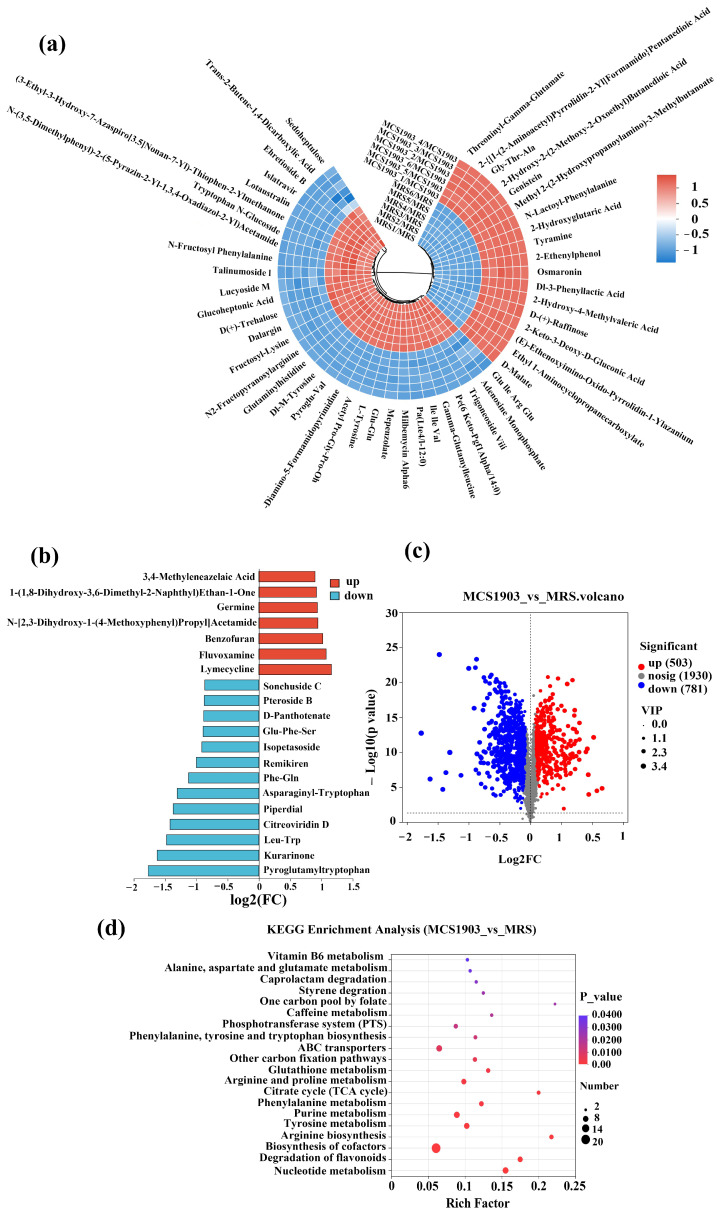
Visualization analysis of differential metabolites between MCS1903 CFS treatment and MRS control groups. A total of 1284 metabolites were identified in the analysis. (**a**) Circular heatmap showing hierarchical clustering. Red indicates high expression in the treatment group, blue indicates low expression. (**b**) Bar plot of differential metabolites. The x-axis shows log_2_FC. (**c**) Volcano plot. (**d**) KEGG pathway enrichment bubble chart. Only significantly enriched pathways with BH-adjusted *p* < 0.05 are presented. The color of each dot represents the BH-adjusted *p*-value, the size indicates the number of differential metabolites.

To identify potential antioxidant-related metabolites in the CFS of MCS1903, non-targeted metabolomic analysis was performed to characterize metabolic profiles under different culture conditions. The circular heatmap (Figure 4a) showed clear separation between groups, indicating distinct metabolite patterns. Changes were observed across several metabolite classes associated with antioxidant activity, including phenolic acids, flavonoids, organic acids, and vitamin-related compounds.

To further identify metabolites with significant changes, the top 20 differential metabolites were visualized in a bar chart (Figure 4b). Among these, 3,4-methylenedioxycinnamic acid, a phenolic acid derivative with reported antioxidant activity, and gemine showed the highest upregulation. In contrast, pantothenic acid (vitamin B5), asparaginyl-tryptophan, and kurarinone were significantly downregulated.

Differential metabolites were further analyzed using a volcano plot (Figure 4c). Based on the criteria VIP > 1, |log_2_FC| ≥ 1, and *p* < 0.05, a total of 503 metabolites were significantly upregulated and 781 downregulated, indicating substantial metabolic variation between groups.

KEGG pathway enrichment identified the biosynthesis of cofactors, which includes key antioxidant-related cofactors such as glutathione and thioredoxin, as the most significantly enriched pathway (Figure 4d). Additional enriched pathways included glutathione metabolism, phenylalanine metabolism, tyrosine metabolism, arginine and proline metabolism, arginine biosynthesis, purine metabolism, citrate cycle (TCA cycle), nucleotide metabolism, degradation of flavonoids, ABC transporters, and other carbon fixation pathways.

### 3.4. Effects of MCS1903 CFS on Caco2 Cells

#### 3.4.1. Effect of CFS Derived from MCS1903 on Viability of Caco2 Cells

**Figure 5:**
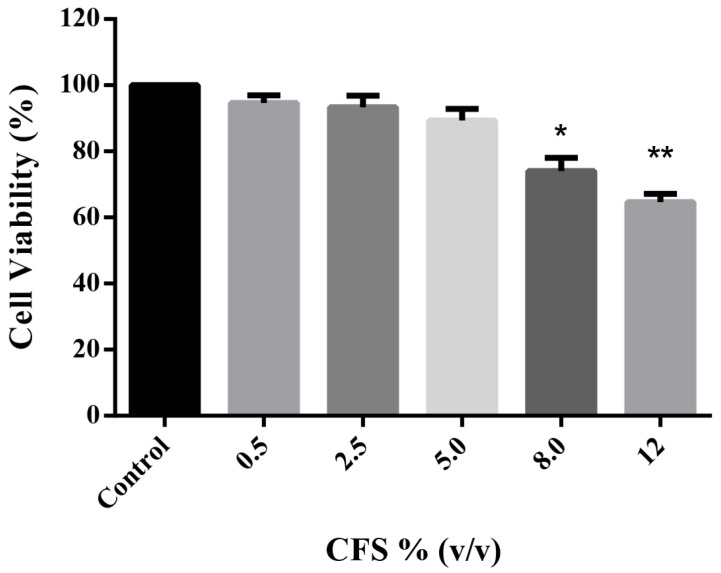
Cytotoxicity of different concentrations of CFS on Caco-2 cells. Data are exhibited as mean ± SD derived from three separate experiments. Versus the control group, * *p* < 0.05 and ** *p* < 0.01 were defined as statistically significant differences.

CFS of MCS1903 (Figure 5) and MRS medium (Figure S2) showed no significant cytotoxicity at concentrations up to 5% (*v*/*v*) after 24 h treatment in Caco2 cells. Therefore, CFS concentrations below 5% (*v*/*v*) were used in subsequent experiments.

#### 3.4.2. Effect of CFS by MCS1903 on ROS Activity in Caco2 Cells

**Figure 6:**
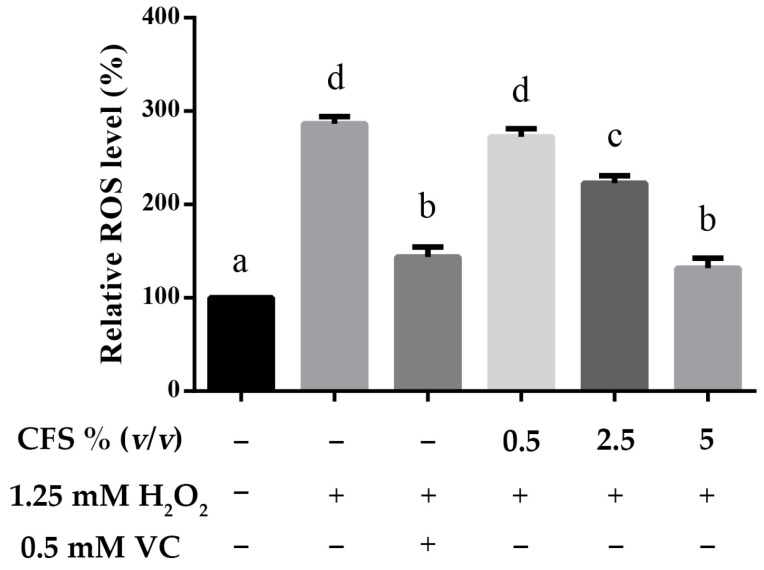
Effects of different concentrations of CFS derived from MCS1903 on intracellular ROS level in Caco-2 cells. Significant intergroup differences (*p* < 0.05) are marked by different letters (a–d).

As shown in Figure 6, H_2_O_2_ treatment significantly increased intracellular ROS levels in Caco-2 cells, compared with the control treatment, while the concentrations of 2.5% and 5% (*v*/*v*) CFS of MCS1903 intervention significantly inhibited the ROS fluorescent signal.

#### 3.4.3. Effect of CFS by MCS1903 on Nrf2/Keap1-HO-1 Pathway in Caco2 Cells

**Figure 7:**
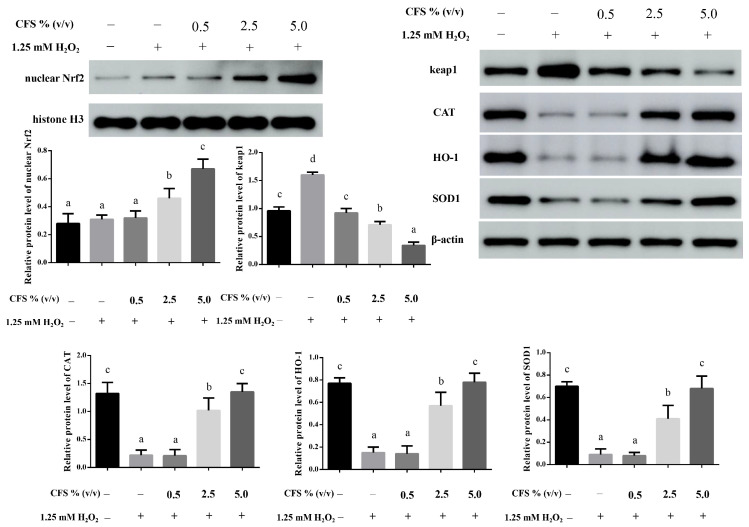
Effects of different concentrations of CFS derived from MCS1903 on the Nrf2/Keap1-HO-1 pathway in Caco-2 cells. Significant intergroup differences (*p* < 0.05) are marked by different letters (a–d).

Immunoblot analysis showed that H_2_O_2_ treatment increased the expression of cytoplasmic Keap1 in Caco-2 cells compared with the control treatment (Figure 7). However, no significant increase in nuclear Nrf2 levels was observed, suggesting that H_2_O_2_-induced oxidative stress did not promote Nrf2 nuclear translocation under the experimental conditions. In contrast, treatment with MCS1903-derived CFS reduced the elevated cytoplasmic Keap1 and increased nuclear Nrf2 levels relative to the H_2_O_2_-treated group.

Analysis of the expression of Nrf2-regulated antioxidant enzymes showed decreased expression of HO-1, CAT, and SOD in Caco-2 cells in the H_2_O_2_-treated group, indicating impairment of the cellular antioxidant defense system. In contrast, MCS1903 CFS treatment significantly increased the expression levels of HO-1, CAT, and SOD1 compared with the H_2_O_2_ treatment.

## 4. Discussion

This study demonstrates that *L*. *plantarum* MCS1903, isolated from naturally fermented tofu whey, exhibits strong antioxidant activity primarily mediated by its extracellular metabolites. Among the isolated strains, the CFS of MCS1903 showed the highest radical scavenging capacity, outperforming intracellular extracts and whole cells, indicating that secreted bioactive compounds are the major contributors to its antioxidant function. Metabolomic profiling further revealed a distinct enrichment of antioxidant-related metabolites, including phenolic acid derivatives and vitamin-associated compounds, suggesting a metabolic basis for this activity. In vitro experiments using Caco-2 cells confirmed that MCS1903 CFS effectively attenuates H_2_O_2_-induced oxidative stress, at least in part through modulation of the Nrf2/Keap1-HO-1 signaling pathway. Collectively, these findings highlight the functional potential of tofu whey-derived LAB as a source of natural antioxidants and support the application of MCS1903 as a candidate postbiotic in functional food development.

A key direction in this field is to move beyond confirming antioxidant activity and to identify the specific cellular fractions responsible for this effect [32,34,35]. In this study, the antioxidant activities of whole cells, intracellular extracts, and CFS were systematically compared across six LAB strains. The CFS of all strains exhibited significantly higher DPPH and OH^−^ radical scavenging activities than the other fractions (*p* < 0.05). Among them, MCS1903 showed the highest activity. These findings indicate that the antioxidant activity of MCS1903 is primarily associated with its extracellular metabolites. These findings are consistent with previous studies demonstrating that LAB-derived CFS exhibits strong antioxidant activity. For instance, Wang et al. (2021) [12] reported that the CFS of *L. plantarum* ZLP001 exerted significant antioxidant protective effects in IPEC-J2 cells. Elhalik et al. (2024) [36] reported that *L*. *plantarum* strains isolated from Egyptian dairy products exhibited DPPH scavenging activities ranging from 71.8% to 93.8%, comparable to that observed for MCS1903. Lee et al. (2025) [37] further reported that heat-killed cells and metabolites of *Latilactobacillus curvatus* and *Latilactobacillus sakei* isolated from green tripe exhibited significant ABTS radical scavenging and FRAP reducing activities. However, contrasting findings have also been reported. Son et al. [9] observed that intact cells of *Lactiplantibacillus paraplantarum* SC61, rather than its CFS, exhibited higher DPPH radical scavenging activity. This discrepancy may be attributed to strain-specific differences, as distinct LAB strains possess unique cell wall structures and metabolite secretion profiles. Specifically, SC61 may express elevated levels of cell wall-bound antioxidant proteins (e.g., surface layer proteins), whereas MCS1903 tends to secrete active metabolites into the extracellular environment. Furthermore, the isolation source may influence metabolic strategies; MCS1903 was isolated from tofu sour liquid, an acidic niche that may have favored the evolution of a more efficient extracellular secretion mechanism. The hydroxyl radical scavenging capacity of MCS1903 CFS (67.50 ± 0.01%) exceeded that of vitamin C (50.43%), suggesting the presence of potent metal-chelating or radical-quenching metabolites in its secretome.

Non-targeted metabolomic analysis revealed substantial metabolic reprogramming in the CFS of MCS1903 compared with the MRS control. Metabolomic profiling revealed a clear separation between MCS1903 and the control group, indicating substantial metabolic reprogramming. Notably, phenolic acid derivatives, flavonoids, and vitamin-related metabolites were identified as key contributors to the observed antioxidant activity. Of particular interest, 3,4-methylenedioxycinnamic acid—a phenolic acid with well-documented antioxidant and anti-inflammatory properties [38,39]—was significantly enriched in the CFS of MCS1903 (log_2_FC > 3). The free radical scavenging capacity of phenols is achieved via hydrogen atom transfer and electron donation processes [40,41], providing a mechanistic basis for the enhanced antioxidant capacity of MCS1903 CFS. Similarly, prior reports have identified diverse phenolic acids and flavonoids—including gallic acid, chlorogenic acid, catechin, quercetin, and kaempferol—as key antioxidant metabolites produced by LAB. In addition, bioactive peptides generated during fermentation contribute to antioxidant activity [36,37,42,43]. Compared with these reports, the present study highlights distinctive features of the MCS1903 metabolite profile. Notably, at least 12 phenolic acid derivatives were detected in its CFS, indicating a broader metabolic spectrum than that reported for other *L. plantarum* strains. This enhanced diversity may be attributed to adaptation to the acidic environment of tofu sour liquid, potentially promoting more efficient secondary metabolism and extracellular secretion.

Comparative analysis with other metabolomic studies further highlights the unique metabolic features of MCS1903. In this study, indole lactic acid and phenyl lactic acid were significantly enriched in MCS1903 CFS, suggesting that these metabolites may play a conserved role in the antioxidant function of various LAB. MCS1903 exhibited enrichment of a wider variety of phenolic acid derivatives and vitamin-related metabolites, reflecting strain-specific differences in metabolic profiles. Similarly, Rezaie et al. (2024) [43] used GC-MS to characterize volatile components in postbiotics and successfully identified acetic acid and hexanol as well as pyrogallol. Among them, short-chain fatty acids such as acetic acid are the main substances contributing to antioxidant activity. In contrast to the predominance of short-chain fatty acids reported in these postbiotics, MCS1903 CFS exhibited a more diverse array of antioxidant metabolites, including phenolic acids, flavonoids, organic acids, and vitamins, suggesting that it may exert more comprehensive antioxidant effects through multi-component synergistic effects.

KEGG pathway enrichment analysis further elucidated the metabolic basis underlying the antioxidant activity of MCS1903. Pathways related to the biosynthesis of cofactors, arginine and proline metabolism, tyrosine metabolism, TCA cycle, and ABC transporters were significantly enriched (*p* < 0.01). Cofactors such as glutathione and thioredoxin are core components of the cellular antioxidant system. Additionally, arginine and proline metabolism, as well as tyrosine metabolism, contribute to the synthesis of bioactive metabolites with radical-scavenging properties, consistent with previous studies showing that these pathways support the production of antioxidant amino acid derivatives in LAB [44]. Di Chiano et al. (2024) [45] also demonstrated that LAB-derived CFS exerted antioxidant and anti-inflammatory responses by triggering the NRF2 pathway, corroborating the metabolic findings of the present study.

The functional relevance of MCS1903 CFS was further validated in vitro using Caco-2 cells, a well-established model of the intestinal epithelial barrier. The CFS exhibited no cytotoxicity at concentrations up to 5% (*v*/*v*) and significantly reduced H_2_O_2_-induced ROS production at 2.5% and 5% (*v*/*v*), indicating a clear protective effect against oxidative stress. Given that oxidative stress in the gastrointestinal tract is closely associated with chronic conditions such as IBD, colorectal cancer, and metabolic disorders [14], these findings suggest that MCS1903 CFS may have potential in mitigating intestinal oxidative damage in vivo.

The safety profile of live *L. plantarum* has been consistently demonstrated. According to a review by Echegaray et al. (2023) [46], this species carries both a Qualified Presumption of Safety (QPS) from European Food Safety Authority (EFSA) and Generally Recognized as Safe (GRAS) status from the US Food and Drug Administration (FDA). Furthermore, a range of investigations have confirmed that live *L. plantarum* can attach to Caco-2 cells without inducing toxic effects. For instance, Stojanov et al. (2024) [47] recently found that *L. plantarum*, whether freely suspended or embedded in nanofibers, did not reduce Caco-2 cell viability and lacked any hemolytic activity. In light of this well-established safety record, our work concentrated on the CFS as a postbiotic preparation for examining extracellular metabolites—an approach routinely adopted in LAB research. The observation that the CFS of *L. plantarum* MCS1903 is non-toxic to Caco-2 cells (Figure 5) is therefore in line with the known safety of this bacterial species.

The Keap1/Nrf2/HO-1 signaling pathway is pivotal in modulating cellular antioxidant defenses [6]. In this study, H_2_O_2_ treatment alone induced only a modest increase in Keap1 expression (1.4-fold) and limited Nrf2 nuclear translocation (1.1-fold), reflecting an insufficient endogenous response to oxidative stress. This was further evidenced by reduced expression of downstream antioxidant enzymes and increased ROS accumulation.

In contrast, treatment with MCS1903 CFS markedly restored redox balance. The CFS significantly downregulated Keap1 expression and enhanced Nrf2 nuclear translocation, leading to substantial upregulation of antioxidant enzymes, including HO-1, SOD1, and CAT. These findings demonstrate that MCS1903 CFS activates the Nrf2 pathway at multiple levels, promoting both Nrf2 release and downstream transcriptional activity.

The extent of Nrf2 activation observed here is comparable to previous reports on *L*. *plantarum* strains, which have been shown to enhance Nrf2 signaling and antioxidant enzyme expression under oxidative stress conditions [45]. Notably, the present findings indicate that a postbiotic preparation (CFS) can achieve similar or even stronger effects, suggesting that secreted metabolites alone are sufficient to trigger this protective pathway.

Furthermore, H_2_O_2_ treatment alone did not induce substantial Nrf2 nuclear translocation in this study, in contrast to a previous report using HT-29 cells [30] that used a longer exposure (22 h) and measured total Nrf2 protein. Preliminary experiments showed that 22 h H_2_O_2_ exposure caused excessive cytotoxicity (>50% cell detachment) in Caco-2 cells, making it difficult to assess Nrf2 nuclear translocation; therefore, a shorter exposure (8 h) was used to capture early signaling events. This discrepancy likely reflects differences in experimental conditions such as exposure duration, cell type, and the specific Nrf2 endpoint. Collectively, these findings suggest that although H_2_O_2_ can initiate an antioxidant response, this response is often inadequate under severe oxidative stress, thereby necessitating exogenous modulators to enhance cellular defense mechanisms.

In addition to modulating intracellular signaling, MCS1903 CFS demonstrated direct antioxidant activity, as evidenced by DPPH and OH^−^ radical scavenging assays. This indicates a dual mode of action: direct neutralization of free radicals and activation of endogenous antioxidant defenses via the Nrf2 pathway. Such combined effects likely underlie the strong cytoprotective properties observed. Emerging evidence suggests that LAB-derived metabolites such as exopolysaccharides (EPS) and indole-3-lactic acid can activate the Nrf2 pathway by modulating Keap1-Nrf2 interactions [40,42]. The detection of indole lactic acid in MCS1903 CFS supports its potential contribution to Nrf2 activation. Nevertheless, the key bioactive components mediating these beneficial effects have not yet been clarified, and further in vivo experiments are still needed for verification. Future studies should therefore focus on the activity-guided identification of active compounds, validation in animal models, comprehensive safety evaluation of the live strain itself, and further investigation of the strain’s effects on intestinal barrier function and gut microbiota.

## 5. Conclusions

In this study, LAB were isolated and identified from naturally fermented tofu whey, and their antioxidant activities were systematically evaluated across whole cells, intracellular extracts, and extracellular secretions. Among the isolates, *L*. *plantarum* MCS1903 exhibited the strongest extracellular antioxidant activity, primarily associated with its CFS. Non-targeted metabolomic analysis revealed distinct alterations in metabolic profiles, including the enrichment of compounds associated with antioxidant functions, providing insight into the potential biochemical basis of this activity. In vitro experiments using Caco-2 cells further demonstrated that the CFS of MCS1903 can attenuate oxidative stress, likely through modulation of the Nrf2/Keap1-HO-1 signaling pathway. Collectively, these findings contribute to a better understanding of the functional properties of LAB derived from tofu whey and highlight the relevance of extracellular metabolites in mediating antioxidant effects. While the results support the potential application of MCS1903 as a source of postbiotic components for functional foods, further studies—particularly in vivo validation and identification of key active compounds—are required to confirm its efficacy and safety.

## Figures and Tables

**Table 1:** DPPH radical scavenging activity (%) of extracellular secretions, cell-free extracts, and whole cells of LAB isolates.

Samples	Cell-Free Supernatant	Cell-Free Extracts	Whole Cells
MCS1901	46.76 ± 2.68 ^Ab^	16.71 ± 1.81 ^Bc^	18.57 ± 2.51 ^Bab^
MCS1902	45.33 ± 1.61 ^Ab^	13.83 ± 0.32 ^Bc^	12.80 ± 3.0 ^Bbb^
MCS1903	71.00 ± 3.58 ^Aa^	31.00 ± 9.55 ^Bb^	25.60 ± 5.2 ^Ba^
MCS1904	69.70 ± 9.3 ^Aa^	41.10 ± 4.55 ^Ba^	20.90 ± 4.30 ^Cab^
MCS1905	45.80 ± 11.92 ^Ab^	15.60 ± 5.7 ^Bc^	19.50 ± 5.8 ^Bab^
MCS1906	38.20 ± 1.5 ^Ab^	36.00 ± 0.8 ^Aab^	18.90 ± 6.8 ^Bab^
Vitamin C	71.40 ± 1.61		

Note: Statistical differences (*p* < 0.05) are marked by different uppercase letters for comparisons across the same row, and by different lowercase letters for comparisons within the same column.

**Table 2:** Hydroxyl free radical scavenging rate (%) of extracellular secretions, cell-free extracts, and whole cells of LAB isolates.

Samples	Cell-Free Supernatant	Cell-Free Extracts	Whole Cells
MCS1901	56.9 ± 0.03 ^Ab^	22.3 ± 0.16 ^Bab^	11.6 ± 0.57 ^Bab^
MCS1902	46.1 ± 0.06 ^Ab^	20.8 ± 0.67 ^Bc^	20.8 ± 0.49 ^Bb^
MCS1903	67.5 ± 0.01 ^Aa^	28.8 ± 0.70 ^Ba^	31.6 ± 0.08 ^Ba^
MCS1904	62.7 ± 0.02 ^Aa^	20.0 ± 0.01 ^Bc^	7.9 ± 0.94 ^Cb^
MCS1905	33.2 ± 0.03 ^Ab^	18.7 ± 0.2 ^Bc^	7.8 ± 0.94 ^Bab^
MCS1906	27.3 ± 0.01 ^Ab^	6.9 ± 0.27 ^Bc^	18.9 ± 0.71 ^Cab^
Vitamin C	50.43 ± 1.61		

Note: Statistical differences (*p* < 0.05) are marked by different uppercase letters for comparisons across the same row, and by different lowercase letters for comparisons within the same column.

## Data Availability

The original data presented in the study are openly available in the OMIX database (China National Center for Bioinformation, CNCB) under accession number OMIX016601 (BioProject: PRJCA063028). The data will be made publicly available upon publication of the manuscript. The supplementary data are available from the corresponding author upon reasonable request.

## Supplementary Materials

The following supporting information can be downloaded at https://www.mdpi.com/article/10.3390/microorganisms14061348/s1. Supplementary Table S1: Full list of identified metabolites, including raw *p*-values, FDR-adjusted *p*-values (Benjamini–Hochberg correction), FC, and VIP values. Supplementary Figure S1: Permutation test of the PLS-DA model for the metabolomic analysis. Supplementary Figure S2: Cytotoxicity analysis of different concentrations of MRS medium on Caco-2 cells.

## Abbreviations

The following abbreviations are used in this manuscript:

LAB	Lactic Acid Bacteria
CFS	Cell-Free Supernatant
ROS	Reactive Oxygen Species
DPPH	2,2-Diphenyl-1-picrylhydrazyl
OH^−^	Hydroxyl Radical
MRS	de Man, Rogosa, and Sharpe (medium)
CFU	Colony-Forming Units
OD600	Optical Density at 600 nm
PCR	Polymerase Chain Reaction
rDNA	Ribosomal Deoxyribonucleic Acid
BLAST	Basic Local Alignment Search Tool
PCA	Principal Component Analysis
PLS-DA	Partial Least Squares Discriminant Analysis
VIP	Variable Importance in Projection
KEGG	Kyoto Encyclopedia of Genes and Genomes
UPLC	Ultra-Performance Liquid Chromatography
TOF	Time of Flight
ESI	Electrospray Ionization
QC	Quality Control
SD	Standard Deviation
ANOVA	Analysis of Variance
CAT	Catalase
SOD1	Superoxide Dismutase 1
HO-1	Heme Oxygenase-1
Nrf2	Nuclear Factor Erythroid 2–Related Factor 2
Keap1	Kelch-like ECH-associated Protein 1
H_2_O_2_	Hydrogen Peroxide
DCFH-DA	2′,7′-Dichlorodihydrofluorescein Diacetate
ECL	Enhanced Chemiluminescence
PVDF	Polyvinylidene Fluoride
SDS-PAGE	Sodium Dodecyl Sulfate–Polyacrylamide Gel Electrophoresis
HRP	Horseradish Peroxidase
FC	Fold Change
