# Supplementary material for: Antioxidant Activity and Metabolomic Characterization of Lactiplantibacillus plantarum MCS1903 Isolated from Naturally Fermented Tofu Whey
Source: Microorganisms. 2026 Jun 16;14(6):1348. doi: 10.3390/microorganisms14061348 (PMC13304304; doi:10.3390/microorganisms14061348)
Supplement: Supplementary file 1 [file microorganisms-14-01348-s001.zip › Supplement figures.pdf]

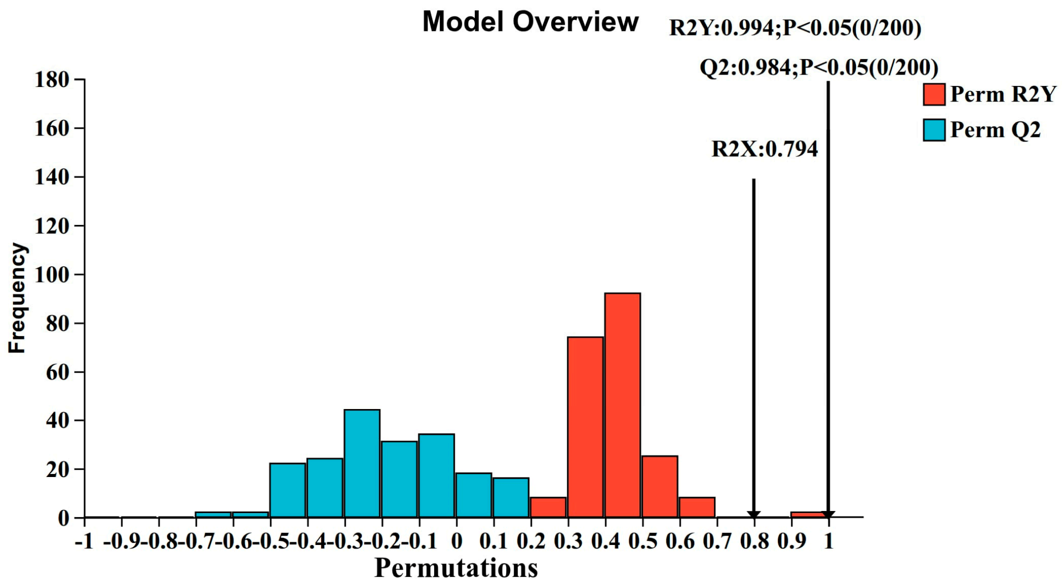

Figure S1. Permutation test of the PLS-DA model for metabolic profile analysis. Red bars, distribution of permuted R^2Y values; blue bars, distribution of permuted Q^2 values. The black vertical lines indicate the original R^2Y (0.994) and Q^2 (0.984) values of the PLS-DA model.

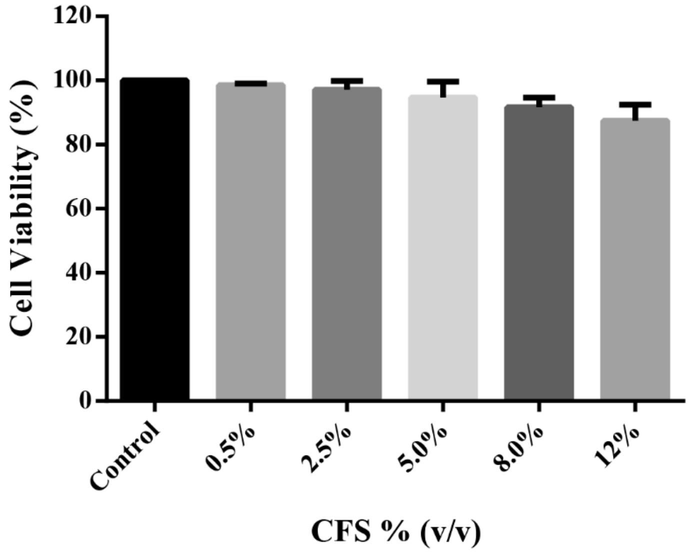

Figure S2. Cytotoxicity of different concentrations of MRS on Caco-2 cells. Cells were treated with indicated concentrations of MRS for 24 h, and cell viability was assessed using the CCK-8 assay. Data are presented as the mean ± SD of three independent experiments.
