# Supplementary material for: Antioxidant Activity and Metabolomic Characterization of Lactiplantibacillus plantarum MCS1903 Isolated from Naturally Fermented Tofu Whey
Source: Microorganisms. 2026 Jun 16;14(6):1348. doi: 10.3390/microorganisms14061348 (PMC13304304; doi:10.3390/microorganisms14061348)
Supplement: Supplementary file 1 [file microorganisms-14-01348-s001.zip › Supplementary Table S1.pdf]

Metabolite	P_value	FDR	VIP_pred_(	VIP_PLS-D	FC(MCS19	Log2FC(M	Significant	Regulate	level	KEGG Com	KEGG Com	KEGG Com	KEGG Path
Pyroglutar	1.84E-13	5.40E-12	3.421	3.4203	0.2933	-1.7695	yes	down	B(i)	-	-	-	-
Asparaginy	1.07E-10	1.04E-09	3.3171	3.316	0.4043	-1.3065	yes	down	B(ii)	-	-	-	-
Leu-Trp	1.05E-24	1.22E-20	3.1605	3.1602	0.3593	-1.4767	yes	down	B(i)	-	-	-	-
Remikiren	9.96E-23	1.79E-19	3.0759	3.0756	0.5	-1	yes	down	B(ii)	C07465	-	-	-
Kurarinone	6.77E-07	2.04E-06	3.0753	3.0733	0.3239	-1.6264	yes	down	B(i)	C17446;_	-	-	-
Sonchusid	1.69E-10	1.52E-09	2.9514	2.9505	0.5484	-0.8668	yes	down	B(ii)	-	-	-	-
Citreovirid	2.06E-05	4.51E-05	2.9348	2.933	0.3736	-1.4206	yes	down	B(ii)	-	-	-	-
Piperdial	8.23E-08	3.15E-07	2.8582	2.857	0.3869	-1.3701	yes	down	B(ii)	C09711	-	-	-
Isopetasos	5.02E-17	6.73E-15	2.8551	2.8547	0.5311	-0.9128	yes	down	B(ii)	-	-	-	-
1-(1,8-Dih	3.21E-11	3.80E-10	2.8545	2.8538	1.8917	0.9197	yes	up	B(i)	-	-	-	-
Penitrem D	1.56E-17	2.70E-15	2.8223	2.8221	0.5936	-0.7524	yes	down	B(ii)	C20596;_	-	-	-
Germine	1.61E-07	5.70E-07	2.8125	2.8116	1.9127	0.9356	yes	up	B(i)	C10807	-	-	-
Pteroside I	4.99E-24	3.15E-20	2.8057	2.8055	0.5456	-0.874	yes	down	B(ii)	-	-	-	-
Benzofurai	7.35E-13	1.69E-11	2.7996	2.7991	2.0314	1.0225	yes	up	B(i)	C14512	-	-	-
Lymecyclir	1.47E-05	3.31E-05	2.754	2.754	2.2333	1.1592	yes	up	B(ii)	-	-	-	-
N-[(3A,5B,	6.54E-10	4.84E-09	2.7408	2.7406	0.5757	-0.7965	yes	down	B(ii)	-	-	-	-
Glu-Leu	3.05E-09	1.79E-08	2.7361	2.7347	0.5733	-0.8027	yes	down	B(i)	-	-	-	-
Phe-Gln	2.01E-07	6.92E-07	2.7296	2.7301	0.4582	-1.1258	yes	down	B(i)	-	-	-	-
Estetrol	4.97E-14	1.87E-12	2.704	2.7033	0.5972	-0.7436	yes	down	B(ii)	_;C22548	-;Hormone	-;Steroid h	-
N-[2,3-Dil	0.000109	0.000209	2.6846	2.6861	1.9227	0.9431	yes	up	B(i)	-	-	-	-
Plumbagir	1.70E-20	1.13E-17	2.678	2.6778	1.5097	0.5943	yes	up	B(ii)	C10387	-	-	-
2-Ethyl-2-	3.37E-09	1.95E-08	2.6746	2.6731	0.591	-0.7587	yes	down	B(ii)	-	-	-	-
Leu-Arg	3.85E-12	6.58E-11	2.6511	2.6506	0.5649	-0.824	yes	down	B(i)	-	-	-	-
(R)-2-Hyd	9.53E-17	1.14E-14	2.6454	2.6452	1.6427	0.7161	yes	up	B(i)	C03042	-	-	-
21-Deoxy(	7.08E-21	5.90E-18	2.632	2.6318	0.6036	-0.7283	yes	down	B(ii)	C05497;_	Hormones	Steroid ho	-
Glu-Phe-S	7.83E-23	1.52E-19	2.6162	2.616	0.5386	-0.8927	yes	down	B(i)	-	-	-	-
Pantothen	9.40E-17	1.13E-14	2.6154	2.6149	0.5867	-0.7693	yes	down	B(i)	C00864;_	Vitamins a	Vitamins;-	map01100
Rimegepai	6.55E-12	1.02E-10	2.5904	2.5905	1.6322	0.7068	yes	up	B(ii)	-	-	-	-
Panax Gins	3.52E-13	9.27E-12	2.5698	2.5696	0.6366	-0.6515	yes	down	B(ii)	-	-	-	-
Chromone	3.96E-09	2.24E-08	2.5649	2.5637	0.6203	-0.6891	yes	down	B(i)	-	-	-	-
Thrombox	1.99E-21	2.21E-18	2.563	2.5628	0.5928	-0.7544	yes	down	B(ii)	-	-	-	-
N-[4-[1-(3	5.10E-13	1.25E-11	2.5587	2.5583	0.6292	-0.6684	yes	down	B(i)	-	-	-	-
Leucyl-Ser	2.86E-11	3.46E-10	2.5499	2.5501	0.6276	-0.6722	yes	down	B(ii)	-	-	-	-
D-Panthot	3.29E-08	1.41E-07	2.5491	2.5498	0.5415	-0.8851	yes	down	B(i)	-	-	-	-
Glu Phe	1.02E-10	1.00E-09	2.5473	2.5471	0.6029	-0.7301	yes	down	B(i)	-	-	-	-
3,4-Methy	8.96E-11	9.00E-10	2.5225	2.5228	1.8628	0.8975	yes	up	B(ii)	-	-	-	-
Sagopilone	9.55E-11	9.50E-10	2.5054	2.505	1.7253	0.7869	yes	up	B(ii)	-	-	-	-

Fluvoxamii	3.28E-05	6.89E-05	2.5048	2.5045	2.1065	1.0749	yes	up	B(ii)	C07571	-	-	-
Asp-Val-P	1.73E-18	4.54E-16	2.4961	2.4958	0.6158	-0.6994	yes	down	B(i)	-	-	-	-
Ala Glu Gl	8.90E-08	3.37E-07	2.4847	2.4834	0.6172	-0.6962	yes	down	B(i)	-	-	-	-
5-Tetradec	6.30E-15	3.38E-13	2.4648	2.4644	1.7324	0.7928	yes	up	B(i)	-	-	-	-
Trimetrexa	1.19E-11	1.68E-10	2.4572	2.4574	1.5736	0.654	yes	up	B(ii)	C11154	-	-	-
N,N-Dieth	4.20E-10	3.32E-09	2.4514	2.4515	0.5699	-0.8113	yes	down	B(i)	-	-	-	-
Thr-Met	1.09E-07	4.03E-07	2.4507	2.4504	0.5597	-0.8372	yes	down	B(i)	-	-	-	-
Ala-Glu-Le	1.42E-20	1.00E-17	2.4425	2.4423	0.6319	-0.6621	yes	down	B(i)	-	-	-	-
Furegrelat	2.33E-10	2.00E-09	2.4379	2.4382	1.5793	0.6593	yes	up	B(i)	-	-	-	-
Arg-Val	4.59E-19	1.49E-16	2.4329	2.4326	0.6326	-0.6606	yes	down	B(i)	-	-	-	-
Ac-Thr-D-	1.12E-08	5.51E-08	2.4271	2.4264	0.6585	-0.6026	yes	down	B(ii)	-	-	-	-
Val Arg	5.80E-13	1.38E-11	2.4267	2.4264	0.6751	-0.5669	yes	down	B(i)	-	-	-	-
Exo-Dehyc	2.18E-10	1.89E-09	2.4234	2.4229	1.5665	0.6476	yes	up	B(ii)	-	-	-	-
(S,E)-Zeara	1.50E-12	3.05E-11	2.4207	2.4205	1.7326	0.7929	yes	up	B(ii)	C09981	-	-	-
(-)-Fustin	8.41E-22	1.23E-18	2.4203	2.4201	0.6445	-0.6338	yes	down	B(i)	-	-	-	-
Valtrate	1.55E-10	1.42E-09	2.4146	2.4146	1.6006	0.6786	yes	up	B(ii)	C09801	-	-	-
Isorhamne	2.01E-09	1.26E-08	2.4096	2.4094	0.6712	-0.5751	yes	down	B(ii)	-	-	-	-
1,2,3-Prop	1.48E-11	1.99E-10	2.3962	2.3964	1.4282	0.5142	yes	up	B(i)	C19806	-	-	-
L-Isoleucy	6.88E-11	7.24E-10	2.3954	2.3948	0.6768	-0.5633	yes	down	B(i)	-	-	-	-
(2R)-2-[[(2	1.71E-10	1.53E-09	2.395	2.3939	0.648	-0.626	yes	down	B(i)	-	-	-	-
Melilotosic	8.30E-11	8.44E-10	2.3883	2.3875	0.6646	-0.5894	yes	down	B(ii)	-	-	-	-
N-(4-Metl	3.36E-06	8.63E-06	2.3833	2.3814	0.5746	-0.7995	yes	down	B(i)	-	-	-	-
Ethyl 2-[(1	2.54E-08	1.12E-07	2.3804	2.3796	1.6497	0.7222	yes	up	B(i)	-	-	-	-
Genistin	1.66E-09	1.07E-08	2.3791	2.3787	0.671	-0.5756	yes	down	B(i)	C09126;_	-	-	map00946
Poly Thf N	1.18E-09	7.95E-09	2.3779	2.3778	1.7287	0.7897	yes	up	B(i)	-	-	-	-
2-(4-Meth	3.58E-10	2.90E-09	2.3757	2.3752	1.6705	0.7403	yes	up	B(i)	-	-	-	-
5-(2-Meth	3.48E-14	1.39E-12	2.3747	2.3741	0.7178	-0.4783	yes	down	B(i)	-	-	-	-
Glu-Ile	1.07E-05	2.47E-05	2.3722	2.3714	0.6014	-0.7336	yes	down	B(i)	-	-	-	-
(24S)-24-E	6.92E-15	3.65E-13	2.3708	2.3705	0.627	-0.6735	yes	down	B(ii)	-	-	-	-
Hydroxy-L	1.80E-13	5.32E-12	2.3679	2.3678	0.6802	-0.556	yes	down	B(ii)	-	-	-	-
Ethyl 3-Ox	1.39E-13	4.32E-12	2.3662	2.3662	1.6801	0.7486	yes	up	B(ii)	C02975	-	-	-
1-Deoxy-[	4.80E-21	4.32E-18	2.3584	2.3582	1.6023	0.6802	yes	up	B(i)	C11437	-	-	map01100
Lucidenic /	7.73E-11	7.99E-10	2.3527	2.3525	0.5916	-0.7572	yes	down	B(ii)	-	-	-	-
Cyclosqua	1.22E-13	3.87E-12	2.351	2.3505	0.6741	-0.5691	yes	down	B(ii)	-	-	-	-
Benzoylarc	1.05E-10	1.02E-09	2.3494	2.3491	0.6461	-0.6301	yes	down	B(ii)	-	-	-	-
Phenylalar	2.96E-11	3.55E-10	2.3459	2.3453	0.6672	-0.5838	yes	down	B(i)	-	-	-	-
Dl-Argini	9.77E-14	3.26E-12	2.3426	2.342	0.7379	-0.4385	yes	down	B(i)	C02385	-	-	-
6-[(Cycloh	1.12E-16	1.29E-14	2.3411	2.3409	1.4664	0.5523	yes	up	B(i)	-	-	-	-

Maesopsir	1.63E-21	2.01E-18	2.3403	2.3401	0.6353	-0.6544	yes	down	B(i)	_;C22553	-	-	map01100
N-Glycolyl	3.17E-09	1.85E-08	2.3379	2.3378	1.5373	0.6204	yes	up	B(ii)	-	-	-	-
Trans-2-H	5.57E-13	1.35E-11	2.3347	2.3341	0.7166	-0.4808	yes	down	B(i)	C01772	-	-	map01100
3-Isochror	2.24E-17	3.61E-15	2.3309	2.3306	1.4143	0.5001	yes	up	B(i)	C07728	-	-	map00624
PI	6.67E-15	3.55E-13	2.3286	2.3284	0.677	-0.5627	yes	down	B(ii)	C18210;_	-	-	-
(5Z)-3-Hy	5.56E-16	4.61E-14	2.3284	2.3281	1.6347	0.709	yes	up	B(i)	-	-	-	-
Lys Glu Th	2.93E-07	9.68E-07	2.3261	2.3242	0.6301	-0.6663	yes	down	B(i)	-	-	-	-
Leu-Pro-A	7.90E-12	1.20E-10	2.324	2.324	0.6365	-0.6517	yes	down	B(i)	-	-	-	-
(S)-5,7-Dil	4.91E-10	3.79E-09	2.3211	2.3209	1.6183	0.6945	yes	up	B(ii)	-	-	-	-
2-(4-Ethyl	1.19E-07	4.35E-07	2.3179	2.317	1.5634	0.6446	yes	up	B(i)	-	-	-	-
N-(2-Amir	1.35E-13	4.23E-12	2.3129	2.3126	0.6567	-0.6067	yes	down	B(i)	-	-	-	-
Fipexide	1.38E-10	1.28E-09	2.309	2.3088	0.6938	-0.5273	yes	down	B(ii)	-	-	-	-
3-Ethyl-1H	2.42E-12	4.47E-11	2.3071	2.3069	1.6618	0.7327	yes	up	B(i)	-	-	-	-
Gdc-0834	2.77E-19	9.91E-17	2.3055	2.3053	0.6889	-0.5376	yes	down	B(i)	-	-	-	-
4-Hydroxy	5.88E-14	2.12E-12	2.3049	2.3044	0.7113	-0.4915	yes	down	B(i)	C00633	-	-	map01100
Val Leu Ala	3.90E-14	1.54E-12	2.3026	2.3022	0.678	-0.5607	yes	down	B(i)	-	-	-	-
Penitrem E	2.46E-13	6.91E-12	2.3002	2.3002	0.6518	-0.6174	yes	down	B(ii)	C20597;_	-	-	-
Gibberellir	1.52E-09	9.91E-09	2.2996	2.2995	0.6655	-0.5874	yes	down	B(ii)	C11868;_	-	-	-
Tyrosyl-Pr	1.91E-08	8.80E-08	2.2993	2.2999	0.7114	-0.4912	yes	down	B(ii)	-	-	-	-
2-Hydroxy	2.45E-14	1.06E-12	2.2992	2.2988	1.3039	0.3828	yes	up	B(i)	-	-	-	-
Methyl 2-/	6.96E-09	3.64E-08	2.2985	2.2978	1.4783	0.564	yes	up	B(i)	-	-	-	-
Ser-Phe	8.83E-09	4.48E-08	2.2957	2.2963	0.6676	-0.5829	yes	down	B(i)	-	-	-	-
N-AcetylIm	2.91E-13	7.92E-12	2.2932	2.2927	1.4508	0.5368	yes	up	B(i)	-	-	-	-
Ile-Ser	8.47E-21	6.81E-18	2.2924	2.2922	0.6759	-0.5651	yes	down	B(i)	-	-	-	-
Eugenosec	2.07E-11	2.64E-10	2.2868	2.2866	0.6893	-0.5367	yes	down	B(ii)	-	-	-	-
3-[5-(2-M	2.89E-12	5.19E-11	2.2861	2.2854	0.7469	-0.421	yes	down	B(i)	-	-	-	-
Cinncassio	1.21E-08	5.93E-08	2.2843	2.2848	0.6636	-0.5916	yes	down	B(ii)	-	-	-	-
2'-O-Meth	6.85E-12	1.06E-10	2.2682	2.268	1.5336	0.6169	yes	up	B(i)	-	-	-	-
2-Hydroxy	6.62E-10	4.89E-09	2.2584	2.258	1.5101	0.5947	yes	up	B(ii)	C05984	-	-	map00640
D-Leucyl-	7.48E-09	3.87E-08	2.2582	2.2573	0.696	-0.5229	yes	down	B(i)	-	-	-	-
Glutaminyl	8.85E-10	6.26E-09	2.2579	2.2581	1.491	0.5763	yes	up	B(i)	-	-	-	-
Xanthoang	1.78E-09	1.13E-08	2.2543	2.2538	0.6753	-0.5665	yes	down	B(ii)	-	-	-	-
Deoxychol	4.17E-20	2.32E-17	2.252	2.2518	0.7043	-0.5058	yes	down	B(ii)	C03033;_	-	-	map01100
Sorbose	2.28E-14	1.00E-12	2.2492	2.2487	0.7138	-0.4864	yes	down	B(i)	C01452;_	-	-	map02060
Arg-Ser-A	1.77E-19	7.40E-17	2.2477	2.2475	0.715	-0.4839	yes	down	B(i)	-	-	-	-
Ile-Pro-As	5.44E-15	2.98E-13	2.2446	2.2443	0.6409	-0.6417	yes	down	B(i)	-	-	-	-
Glu-Gly-Le	3.17E-10	2.60E-09	2.2407	2.241	0.68	-0.5565	yes	down	B(i)	-	-	-	-
5-Methylte	7.04E-21	5.90E-18	2.2364	2.2362	0.6888	-0.5379	yes	down	B(ii)	C00440	-	-	map01100

Fluprednis	1.03E-19	5.00E-17	2.2319	2.2317	0.6697	-0.5784	yes	down	B(ii)	C14618;_	-	-	-
Val-Met	1.80E-13	5.32E-12	2.2294	2.2289	0.6746	-0.5678	yes	down	B(i)	-	-	-	-
5-(Biotinar	3.35E-12	5.87E-11	2.2291	2.2289	1.4638	0.5497	yes	up	B(ii)	-	-	-	-
(S)-(-)-Alp	2.80E-21	2.84E-18	2.2278	2.2276	1.3617	0.4454	yes	up	B(i)	-	-	-	-
(2R)-2-Am	6.16E-11	6.59E-10	2.2265	2.2267	0.6878	-0.5399	yes	down	B(i)	-	-	-	-
Norcamph	1.64E-12	3.29E-11	2.224	2.2242	1.4297	0.5157	yes	up	B(i)	-	-	-	-
Ala-Val	9.19E-13	2.03E-11	2.2198	2.2191	0.7196	-0.4747	yes	down	B(i)	-	-	-	-
(1R,6R)-6-	2.53E-09	1.53E-08	2.2133	2.2134	0.6596	-0.6003	yes	down	B(ii)	C05817	-	-	map01100
L-Valyl-L-	1.86E-12	3.64E-11	2.2105	2.21	0.7019	-0.5106	yes	down	B(i)	-	-	-	-
L-2-Aminc	2.21E-12	4.17E-11	2.2104	2.2104	1.5684	0.6493	yes	up	B(ii)	C03872	-	-	map00564
Ile-Trp	3.48E-21	3.38E-18	2.2087	2.2085	0.6821	-0.5519	yes	down	B(i)	-	-	-	-
Fenamic A	5.26E-14	1.96E-12	2.2085	2.2081	1.6475	0.7203	yes	up	B(i)	C13697	-	-	-
N4-Acetyl	1.78E-14	8.23E-13	2.2062	2.2062	1.5241	0.608	yes	up	B(ii)	C05936	-	-	map01100
Glutamyl-'	6.26E-10	4.66E-09	2.2041	2.2044	0.7125	-0.489	yes	down	B(i)	-	-	-	-
Thymoper	1.85E-07	6.43E-07	2.2026	2.2015	1.4909	0.5762	yes	up	B(ii)	-	-	-	-
Dl-3-Pher	2.20E-14	9.73E-13	2.201	2.2006	1.2827	0.3592	yes	up	B(i)	C01479	-	-	map01110
(2S,4R)-4-	2.13E-11	2.71E-10	2.2007	2.2009	1.4986	0.5836	yes	up	B(i)	-	-	-	-
N-[5-(2,5-	5.73E-16	4.70E-14	2.1985	2.1983	0.6948	-0.5254	yes	down	B(i)	-	-	-	-
Trans-1,2-	8.81E-10	6.24E-09	2.1963	2.1955	1.5601	0.6417	yes	up	B(ii)	C04221	-	-	-
Tyr-Ile	2.70E-10	2.27E-09	2.196	2.1953	0.7032	-0.5081	yes	down	B(i)	-	-	-	-
L-Isoleucy	2.32E-08	1.04E-07	2.1948	2.1941	0.7244	-0.4652	yes	down	B(i)	-	-	-	-
Amastatin	1.49E-19	6.57E-17	2.1931	2.1929	0.7158	-0.4825	yes	down	B(ii)	C01552;_	-	-	-
Arjungenir	9.00E-13	2.00E-11	2.1896	2.1891	0.6751	-0.5667	yes	down	B(i)	-	-	-	-
3A,6B,7A,1	1.93E-10	1.69E-09	2.1877	2.1879	0.7203	-0.4732	yes	down	B(ii)	C01094	-	-	map01100
Asp-Lys-H	1.37E-10	1.28E-09	2.1855	2.1847	0.6938	-0.5274	yes	down	B(i)	-	-	-	-
N(Epsilon)	3.31E-15	2.02E-13	2.1847	2.1847	0.6475	-0.627	yes	down	B(ii)	-	-	-	-
Prostaglan	7.63E-07	2.27E-06	2.1843	2.1841	0.6895	-0.5365	yes	down	B(ii)	-	-	-	-
Ile-Phe	1.16E-09	7.86E-09	2.1816	2.181	0.6875	-0.5405	yes	down	B(i)	-	-	-	-
Dihydroou	5.59E-11	6.07E-10	2.179	2.1791	0.7183	-0.4774	yes	down	B(ii)	-	-	-	-
Noroxymo	5.21E-10	3.98E-09	2.1787	2.1791	1.5861	0.6655	yes	up	B(i)	-	-	-	-
Trp-Glu-A	5.76E-11	6.21E-10	2.1786	2.1777	1.4159	0.5017	yes	up	B(i)	-	-	-	-
12-Metho:	3.58E-20	2.08E-17	2.178	2.1778	0.7559	-0.4037	yes	down	B(i)	-	-	-	-
Val-Val	2.85E-11	3.46E-10	2.1777	2.177	0.724	-0.4659	yes	down	B(i)	-	-	-	-
Arg-Leu	4.47E-15	2.55E-13	2.1768	2.1765	0.7399	-0.4347	yes	down	B(i)	-	-	-	-
3-Methyl-	6.72E-14	2.38E-12	2.1758	2.1754	1.3879	0.4729	yes	up	B(i)	-	-	-	-
Ketoprofer	3.59E-13	9.42E-12	2.1752	2.1746	0.6974	-0.5199	yes	down	B(ii)	C01716	-	-	-
Dimethyl H	2.03E-09	1.27E-08	2.1724	2.1721	1.4618	0.5477	yes	up	B(i)	-	-	-	-
6-(4-O-Be	4.50E-08	1.85E-07	2.1717	2.1706	0.7042	-0.506	yes	down	B(ii)	C03423	-	-	map01110

Dl-Alanyl-	1.86E-19	7.40E-17	2.1693	2.1691	0.6781	-0.5604	yes	down	B(i)	-	-	-	-
Niacinamic	2.69E-14	1.14E-12	2.1688	2.1684	0.7578	-0.4001	yes	down	B(i)	C00153	Vitamins a	Vitamins	map01100
Gly-Ile	7.75E-14	2.69E-12	2.1677	2.1672	0.7303	-0.4534	yes	down	B(i)	-	-	-	-
Indoleacet	4.42E-14	1.71E-12	2.1672	2.1669	1.6098	0.6868	yes	up	B(i)	C00637	-	-	map01100
Glucose-6	9.74E-16	7.47E-14	2.1555	2.1552	0.7681	-0.3806	yes	down	B(ii)	-	-	-	-
Antimycin	2.22E-19	8.21E-17	2.1531	2.1529	0.7259	-0.4622	yes	down	B(ii)	C11339	-	-	-
Cosmosiin	1.98E-08	9.09E-08	2.153	2.1523	0.7221	-0.4698	yes	down	B(i)	C04608	-	-	-
Indole-3-l	7.04E-17	8.73E-15	2.1521	2.1518	1.3577	0.4411	yes	up	B(i)	C02043	-	-	-
Glutaminyl	4.88E-12	7.96E-11	2.1517	2.1512	0.7289	-0.4563	yes	down	B(i)	-	-	-	-
Gemfibroz	3.45E-12	6.00E-11	2.1513	2.1513	0.7057	-0.5029	yes	down	B(ii)	-	-	-	-
Ile-Glu-As	1.79E-08	8.31E-08	2.1462	2.1449	0.7095	-0.4951	yes	down	B(i)	-	-	-	-
Polypodos	2.93E-16	2.72E-14	2.1432	2.1431	0.7149	-0.4843	yes	down	B(ii)	-	-	-	-
Glycerylmo	4.62E-14	1.77E-12	2.1385	2.1384	0.6687	-0.5805	yes	down	B(ii)	-	-	-	-
Glu-Ser	2.09E-08	9.51E-08	2.1343	2.1343	0.7259	-0.4621	yes	down	B(i)	-	-	-	-
Tyramine	4.86E-19	1.53E-16	2.1341	2.1339	1.284	0.3606	yes	up	B(i)	C00483	Peptides	Amines	map01100
Nelfinavir	6.67E-18	1.36E-15	2.1303	2.13	0.7241	-0.4657	yes	down	B(ii)	C07257	-	-	-
Delta-Hex	9.39E-08	3.53E-07	2.1284	2.1284	1.5707	0.6514	yes	up	B(i)	-	-	-	-
Monocrota	3.16E-10	2.60E-09	2.124	2.1239	1.4814	0.5669	yes	up	B(ii)	C10350	-	-	-
Epothilone	0.000155	0.000289	2.119	2.1177	0.6755	-0.5659	yes	down	B(ii)	C12039;_	-	-	-
Cetyl Recir	6.42E-10	4.76E-09	2.1188	2.1186	1.4672	0.553	yes	up	B(ii)	-	-	-	-
Glu-Met-L	1.13E-13	3.63E-12	2.1159	2.1157	0.7003	-0.5139	yes	down	B(i)	-	-	-	-
Threonylpl	4.77E-12	7.82E-11	2.1158	2.1155	0.7012	-0.512	yes	down	B(i)	-	-	-	-
Glucohept	4.77E-13	1.18E-11	2.1122	2.1117	0.7947	-0.3314	yes	down	B(ii)	-	-	-	-
Glycyl-Phe	5.49E-11	5.98E-10	2.1089	2.1081	0.726	-0.4619	yes	down	B(ii)	-	-	-	-
Lyxosylam	1.63E-10	1.48E-09	2.1087	2.1088	1.4505	0.5366	yes	up	B(i)	-	-	-	-
L-(-)-Sorb	1.32E-13	4.16E-12	2.1085	2.1081	1.2867	0.3637	yes	up	B(i)	C08356	-	-	-
Hippuric A	8.39E-11	8.51E-10	2.1078	2.1078	0.6845	-0.5469	yes	down	B(i)	C01586	-	-	-
Lpe(O-9:0	6.81E-13	1.59E-11	2.1064	2.1061	0.7112	-0.4916	yes	down	B(i)	-	-	-	-
Cynaroside	3.52E-17	5.22E-15	2.1031	2.1028	0.7058	-0.5028	yes	down	B(ii)	-	-	-	-
D-Leucyl-	2.93E-09	1.73E-08	2.1009	2.1	0.7267	-0.4605	yes	down	B(i)	-	-	-	-
2-Hydroxy	3.12E-15	1.91E-13	2.0992	2.0989	1.2756	0.3512	yes	up	B(i)	-	-	-	-
3-Formyl F	1.02E-18	2.90E-16	2.0972	2.097	0.7553	-0.4048	yes	down	B(ii)	-	-	-	-
(2R)-2-[[(2	1.31E-20	9.52E-18	2.0967	2.0965	0.7123	-0.4894	yes	down	B(i)	-	-	-	-
Tyr-Glu	1.31E-14	6.35E-13	2.0908	2.0904	0.7169	-0.4802	yes	down	B(i)	-	-	-	-
Phenylalar	2.50E-07	8.40E-07	2.0884	2.0877	0.7259	-0.4621	yes	down	B(i)	-	-	-	-
Trp-Ser-A	8.44E-13	1.89E-11	2.0862	2.0863	1.3451	0.4277	yes	up	B(i)	-	-	-	-
Leu Val	7.05E-14	2.48E-12	2.0818	2.0813	0.7542	-0.407	yes	down	B(i)	-	-	-	-
Sibrafiban	1.67E-05	3.73E-05	2.0817	2.0805	1.4458	0.5319	yes	up	B(ii)	-	-	-	-

Alpha-Bixi	1.52E-05	3.41E-05	2.0816	2.0837	0.6497	-0.6221	yes	down	B(ii)	C08582	-	-	-
Val-Glu-Le	5.34E-14	1.97E-12	2.081	2.0806	0.7596	-0.3967	yes	down	B(i)	-	-	-	-
Beta-Alany	1.79E-14	8.23E-13	2.0782	2.0778	0.7605	-0.3949	yes	down	B(i)	-	-	-	-
Dethiobiot	2.60E-08	1.15E-07	2.0775	2.0769	1.4744	0.5601	yes	up	B(ii)	C01909	-	-	map01100
Lys-Val	2.61E-11	3.20E-10	2.0754	2.0748	0.7579	-0.3999	yes	down	B(i)	-	-	-	-
Glycyl-L-L	1.11E-15	8.19E-14	2.0731	2.0727	0.754	-0.4074	yes	down	B(i)	C02155	-	-	-
L-Tyrosine	1.05E-16	1.25E-14	2.0685	2.0682	0.7979	-0.3257	yes	down	B(i)	C00082	Peptides	Amino aci	map01100
3-(4-Meth	2.41E-08	1.07E-07	2.0672	2.0676	1.4062	0.4918	yes	up	B(i)	-	-	-	-
3,4-Dihydr	5.92E-08	2.35E-07	2.0663	2.0662	1.4812	0.5668	yes	up	B(i)	-	-	-	-
(3,7-Dimet	2.36E-10	2.03E-09	2.0656	2.0659	1.4298	0.5158	yes	up	B(i)	-	-	-	-
Glu-Glu-M	3.66E-19	1.22E-16	2.0649	2.0646	0.7231	-0.4678	yes	down	B(i)	-	-	-	-
Oxypurino	1.11E-09	7.58E-09	2.0632	2.0627	0.7035	-0.5075	yes	down	B(i)	C07599	-	-	-
L-Leucyl-L	2.10E-19	7.88E-17	2.0613	2.0612	0.7569	-0.4018	yes	down	B(i)	-	-	-	-
Ceanothin	1.48E-10	1.36E-09	2.0599	2.0595	0.7407	-0.433	yes	down	B(ii)	-	-	-	-
5'-Hydroxy	7.35E-13	1.69E-11	2.0597	2.0595	1.3023	0.381	yes	up	B(ii)	-	-	-	-
3-[1-(Benz	2.06E-11	2.63E-10	2.0586	2.0582	0.7457	-0.4232	yes	down	B(i)	-	-	-	-
Vpgpr Ente	1.51E-18	4.15E-16	2.0582	2.0581	0.7424	-0.4297	yes	down	B(ii)	-	-	-	-
Lysyl-Isole	1.08E-15	8.05E-14	2.057	2.0567	0.7781	-0.3619	yes	down	B(i)	-	-	-	-
Trp-Val	6.22E-08	2.46E-07	2.0526	2.0529	0.6925	-0.5301	yes	down	B(i)	-	-	-	-
(2R,3S)-2-	1.07E-17	2.04E-15	2.0507	2.0504	0.7776	-0.363	yes	down	B(i)	-	-	-	-
Adenosine	8.56E-14	2.94E-12	2.0493	2.0488	0.7862	-0.347	yes	down	B(i)	C00212;_	Nucleic ac	Nucleoside	map01100
Ile Asp Trp	3.68E-12	6.36E-11	2.0486	2.0486	0.7357	-0.4428	yes	down	B(i)	-	-	-	-
Gamma-L-	2.87E-09	1.70E-08	2.0467	2.0471	0.7206	-0.4728	yes	down	B(i)	C05283	-	-	-
Pro-Asn	9.32E-10	6.52E-09	2.0465	2.0457	0.7488	-0.4173	yes	down	B(i)	-	-	-	-
Asp Ile	8.14E-11	8.32E-10	2.0462	2.0465	0.7215	-0.4709	yes	down	B(i)	-	-	-	-
(2R,3S)-2-	1.07E-14	5.31E-13	2.0454	2.045	0.7612	-0.3937	yes	down	B(i)	-	-	-	-
(2S,3S,5R)-	4.44E-08	1.82E-07	2.0425	2.0425	0.7252	-0.4635	yes	down	B(ii)	-	-	-	-
2-Methylb	7.27E-14	2.55E-12	2.0415	2.0413	1.3426	0.425	yes	up	B(i)	C07215	-	-	map01100
Heliangin	8.69E-15	4.47E-13	2.04	2.0399	0.7317	-0.4507	yes	down	B(ii)	C09474;_	-	-	-
Octopamir	1.24E-16	1.42E-14	2.0394	2.0391	0.7858	-0.3477	yes	down	B(i)	C04227	-	-	-
3-Acetami	9.91E-05	0.000191	2.0388	2.0407	0.6595	-0.6004	yes	down	B(i)	-	-	-	-
Asp-Thr-Il	3.60E-13	9.43E-12	2.0346	2.0341	0.7816	-0.3556	yes	down	B(i)	-	-	-	-
Blumenol (	2.88E-14	1.20E-12	2.0332	2.0333	0.7628	-0.3906	yes	down	B(ii)	-	-	-	-
Trp-Ala-Tl	1.32E-12	2.74E-11	2.0329	2.033	1.3783	0.4629	yes	up	B(i)	-	-	-	-
2-Ethenylp	3.58E-18	8.16E-16	2.0286	2.0284	1.2291	0.2976	yes	up	B(i)	-	-	-	-
Leu Thr	1.28E-10	1.21E-09	2.0237	2.0229	0.7675	-0.3818	yes	down	B(i)	-	-	-	-
Pe(6 Keto-	1.16E-17	2.17E-15	2.0213	2.0212	0.7993	-0.3231	yes	down	B(ii)	-	-	-	-
Glu-Val	2.78E-10	2.32E-09	2.0197	2.0192	0.7634	-0.3895	yes	down	B(i)	-	-	-	-

5-Oxo-Pro	1.51E-11	2.02E-10	2.0151	2.0153	0.7492	-0.4165	yes	down	B(ii)	-	-	-	-
Glu-Gly	3.00E-10	2.48E-09	2.0128	2.0129	0.7276	-0.4588	yes	down	B(i)	-	-	-	-
N-(1-Carb	2.67E-14	1.14E-12	2.0087	2.0083	0.7887	-0.3424	yes	down	B(i)	-	-	-	-
Phe-Ile	2.13E-09	1.32E-08	2.0086	2.0079	0.6947	-0.5256	yes	down	B(i)	-	-	-	-
Arginyl-Gl	3.20E-10	2.63E-09	2.0074	2.0073	1.3655	0.4494	yes	up	B(ii)	-	-	-	-
Ser-Val	5.11E-13	1.25E-11	2.0072	2.0067	0.7522	-0.4108	yes	down	B(i)	-	-	-	-
Majoroside	4.36E-09	2.43E-08	2.0072	2.0073	0.7626	-0.3909	yes	down	B(ii)	C00303	-	-	-
Erdafitinib	1.40E-12	2.88E-11	2.0061	2.006	0.734	-0.4462	yes	down	B(ii)	-	-	-	-
Peg Mono	5.08E-10	3.90E-09	2.0058	2.0057	0.7183	-0.4774	yes	down	B(i)	-	-	-	-
Dg(Lte4/I-	5.39E-07	1.67E-06	2.0056	2.0041	0.7538	-0.4077	yes	down	B(ii)	-	-	-	-
4-Hydroxy	7.08E-10	5.18E-09	2.0056	2.0056	0.7117	-0.4907	yes	down	B(i)	-	-	-	-
(2S,3S)-2-	1.97E-19	7.64E-17	2.0036	2.0033	0.7797	-0.359	yes	down	B(i)	-	-	-	-
Aginoside	1.54E-14	7.28E-13	2.0035	2.0034	0.7958	-0.3294	yes	down	B(ii)	-	-	-	-
Ile Ile	4.02E-15	2.35E-13	2.0034	2.003	0.7467	-0.4214	yes	down	B(i)	-	-	-	-
Ginkgolide	2.06E-19	7.86E-17	2.0028	2.0026	0.7522	-0.4108	yes	down	B(ii)	C07603	-	-	-
(R)-1-O-[E	1.29E-20	9.52E-18	2.0022	2.002	0.7447	-0.4253	yes	down	B(ii)	-	-	-	-
Met-Glu-L	2.46E-15	1.58E-13	2.0021	2.0019	0.7336	-0.447	yes	down	B(i)	-	-	-	-
1,2,3,4-Tet	1.19E-15	8.78E-14	1.9929	1.9926	1.2922	0.3698	yes	up	B(ii)	-	-	-	-
Divanillylte	4.33E-17	6.01E-15	1.9913	1.991	0.7406	-0.4333	yes	down	B(ii)	-	-	-	-
D-Alanyl-l	2.08E-16	2.02E-14	1.9889	1.9887	0.7394	-0.4356	yes	down	B(i)	-	-	-	-
Anemonin	2.64E-08	1.16E-07	1.9887	1.9877	0.7363	-0.4417	yes	down	B(ii)	C16913	-	-	-
Argininosu	1.81E-08	8.38E-08	1.9869	1.9869	1.3537	0.4369	yes	up	B(i)	C03406	Peptides	Amino aci	map01100
Pteroside Z	3.35E-18	7.72E-16	1.9869	1.9867	0.732	-0.4501	yes	down	B(ii)	-	-	-	-
Isoleucylar	2.22E-14	9.77E-13	1.9865	1.9862	0.7729	-0.3716	yes	down	B(i)	-	-	-	-
Glu-Thr	2.13E-09	1.32E-08	1.986	1.9867	0.7782	-0.3617	yes	down	B(i)	-	-	-	-
D-Glucaro	8.97E-15	4.57E-13	1.986	1.9856	0.7298	-0.4544	yes	down	B(ii)	C21095;_	-	-	-
Alpha-Glu	8.19E-12	1.23E-10	1.9844	1.9839	0.7726	-0.3722	yes	down	B(i)	-	-	-	-
(2S)-3-Hy	4.64E-13	1.16E-11	1.9838	1.9833	0.765	-0.3864	yes	down	B(ii)	-	-	-	-
N-L-Gamr	6.05E-15	3.27E-13	1.983	1.9827	0.7839	-0.3512	yes	down	B(i)	-	-	-	-
1-(2-Hydr	2.46E-08	1.09E-07	1.9822	1.9823	0.7347	-0.4448	yes	down	B(ii)	-	-	-	-
P-Hydroxy	1.26E-17	2.28E-15	1.9804	1.9803	1.287	0.364	yes	up	B(i)	C03672	-	-	-
Nebraceta	1.55E-16	1.66E-14	1.98	1.9798	0.7211	-0.4717	yes	down	B(ii)	-	-	-	-
Phenylacet	4.58E-15	2.60E-13	1.9788	1.9784	0.7785	-0.3612	yes	down	B(i)	C07086	-	-	map01100
L-Prolyl-L-	2.00E-08	9.17E-08	1.9771	1.9769	1.362	0.4457	yes	up	B(ii)	-	-	-	-
Tsangane	1.09E-12	2.33E-11	1.9731	1.9728	0.7527	-0.4099	yes	down	B(ii)	-	-	-	-
Trp-Asn-Il	3.35E-08	1.43E-07	1.9711	1.9713	0.7321	-0.4499	yes	down	B(i)	-	-	-	-
(2S,3R)-2-	4.42E-16	3.86E-14	1.9692	1.969	0.7439	-0.4269	yes	down	B(i)	-	-	-	-
(1R,6S)-3-	3.42E-20	2.05E-17	1.9689	1.9688	1.2461	0.3174	yes	up	B(i)	-	-	-	-

Gly Leu Gl	1.79E-09	1.14E-08	1.9674	1.968	0.7343	-0.4456	yes	down	B(i)	-	-	-	-
Leu Glu	1.22E-17	2.24E-15	1.9653	1.965	0.7984	-0.3248	yes	down	B(i)	-	-	-	-
His-Gln-H	0.000127	0.00024	1.9637	1.9645	0.7156	-0.4828	yes	down	B(i)	-	-	-	-
Digitoxige	3.91E-05	8.11E-05	1.9636	1.9634	0.7407	-0.433	yes	down	B(ii)	-	-	-	-
Ala-Glu-M	1.52E-17	2.65E-15	1.962	1.9618	0.7413	-0.4318	yes	down	B(i)	-	-	-	-
His-Leu-S	7.65E-14	2.66E-12	1.9567	1.9562	0.7735	-0.3705	yes	down	B(i)	-	-	-	-
3,3'-Methy	2.13E-11	2.71E-10	1.9565	1.9569	0.7649	-0.3866	yes	down	B(i)	-	-	-	-
Leu-Ile	2.49E-09	1.50E-08	1.9563	1.9555	0.756	-0.4035	yes	down	B(i)	-	-	-	-
Hydroxypr	1.29E-18	3.57E-16	1.9528	1.9526	0.7244	-0.4652	yes	down	B(ii)	-	-	-	-
Dl-4-Hydr	4.41E-11	4.97E-10	1.949	1.9492	1.3144	0.3944	yes	up	B(i)	-	-	-	-
Ala-Met-S	3.05E-12	5.44E-11	1.946	1.9456	0.7644	-0.3876	yes	down	B(i)	-	-	-	-
(2R,3S)-2-	9.27E-13	2.04E-11	1.9442	1.9436	0.784	-0.3511	yes	down	B(i)	-	-	-	-
Ile Leu	7.27E-14	2.55E-12	1.9422	1.9418	0.7768	-0.3644	yes	down	B(i)	-	-	-	-
Gln-Ser-Il	3.75E-11	4.32E-10	1.9414	1.9411	0.7876	-0.3445	yes	down	B(i)	-	-	-	-
Glu-Gln	2.98E-10	2.47E-09	1.9389	1.9388	0.7568	-0.4021	yes	down	B(i)	-	-	-	-
L-Leucyl-L	1.82E-15	1.27E-13	1.9388	1.9387	0.783	-0.3529	yes	down	B(i)	-	-	-	-
Deoxychol	4.03E-14	1.58E-12	1.938	1.9379	0.7545	-0.4064	yes	down	B(ii)	-	-	-	-
Alanylpher	1.10E-15	8.16E-14	1.9367	1.9365	0.7792	-0.36	yes	down	B(i)	-	-	-	-
Thr Leu	9.17E-17	1.11E-14	1.9334	1.9331	0.7925	-0.3355	yes	down	B(i)	-	-	-	-
Leucyl-Glu	1.97E-16	1.98E-14	1.9322	1.932	0.7989	-0.324	yes	down	B(i)	-	-	-	-
6-(6-Amir	2.92E-13	7.94E-12	1.9314	1.9309	0.7897	-0.3405	yes	down	B(i)	C01255	-	-	map00930
Allyl Cyste	1.81E-18	4.63E-16	1.9293	1.9291	1.2659	0.3401	yes	up	B(ii)	-	-	-	-
Valylpheny	2.16E-14	9.56E-13	1.9277	1.9272	0.7716	-0.3741	yes	down	B(i)	-	-	-	-
R-(-)-Pher	6.32E-17	7.96E-15	1.9243	1.9241	1.2516	0.3237	yes	up	B(i)	-	-	-	-
Val Val	4.96E-14	1.86E-12	1.9239	1.9234	0.7694	-0.3783	yes	down	B(i)	-	-	-	-
Asp-Ala-L	1.66E-07	5.87E-07	1.9236	1.9239	1.3559	0.4393	yes	up	B(i)	-	-	-	-
Gln-Asp-L	3.74E-10	3.01E-09	1.9231	1.9229	0.7778	-0.3624	yes	down	B(i)	-	-	-	-
Nicotinuri	2.32E-15	1.51E-13	1.9185	1.9184	0.7354	-0.4435	yes	down	B(ii)	C05380	-	-	map00760
N-(3-Etho	5.19E-10	3.98E-09	1.9178	1.9175	0.7754	-0.3669	yes	down	B(i)	-	-	-	-
Hydroxypr	5.42E-15	2.98E-13	1.9171	1.9171	0.7907	-0.3388	yes	down	B(ii)	-	-	-	-
N-(3-Acet	2.92E-13	7.94E-12	1.9164	1.916	0.7798	-0.3589	yes	down	B(ii)	-	-	-	-
Ethyl 2-Me	1.23E-10	1.18E-09	1.9159	1.9158	1.3328	0.4144	yes	up	B(i)	-	-	-	-
Glu-Ala-Le	2.29E-07	7.78E-07	1.9153	1.9145	0.764	-0.3883	yes	down	B(i)	-	-	-	-
Ile-Met	2.43E-07	8.19E-07	1.9149	1.916	0.7596	-0.3967	yes	down	B(i)	-	-	-	-
N-(3-Metl	2.95E-17	4.50E-15	1.9146	1.9144	1.2514	0.3235	yes	up	B(i)	C04083	-	-	map01110
(2-Hydrox	2.31E-09	1.41E-08	1.9117	1.9119	1.3406	0.4229	yes	up	B(i)	-	-	-	-
Pip(Pgf2Al	3.00E-06	7.79E-06	1.9112	1.9111	0.759	-0.3979	yes	down	B(ii)	-	-	-	-
Tripdiolide	5.48E-11	5.98E-10	1.9112	1.9116	0.7689	-0.3791	yes	down	B(ii)	C09202;_	-	-	-

Lysyl-Pher	3.38E-09	1.96E-08	1.9103	1.9098	0.7677	-0.3813	yes	down	B(ii)	-	-	-	-
Thr-Val	6.05E-17	7.83E-15	1.9079	1.9077	0.8077	-0.3082	yes	down	B(i)	-	-	-	-
Leu Lys	4.82E-15	2.69E-13	1.9074	1.907	0.7992	-0.3234	yes	down	B(i)	-	-	-	-
Glu-Thr-Le	2.30E-11	2.88E-10	1.9035	1.9031	0.7806	-0.3573	yes	down	B(i)	-	-	-	-
Asp Phe	1.13E-08	5.57E-08	1.9022	1.9015	0.7536	-0.4081	yes	down	B(i)	-	-	-	-
(2S,2'S)-Py	2.72E-16	2.55E-14	1.9003	1.9001	1.2585	0.3317	yes	up	B(ii)	-	-	-	-
5-[(2,5-Dir	1.10E-08	5.42E-08	1.8991	1.8991	1.3327	0.4143	yes	up	B(i)	-	-	-	-
Val-Ser	9.40E-11	9.37E-10	1.8983	1.8981	0.7778	-0.3626	yes	down	B(i)	-	-	-	-
3-Hydroxy	4.15E-07	1.33E-06	1.8964	1.8964	1.3965	0.4818	yes	up	B(i)	-	-	-	-
Ile-Ile-Ala	3.06E-07	1.01E-06	1.8943	1.8952	0.7677	-0.3815	yes	down	B(ii)	-	-	-	-
Asp Pro Va	2.20E-12	4.14E-11	1.8917	1.8914	0.7675	-0.3818	yes	down	B(i)	-	-	-	-
Phe-Asp	7.18E-07	2.14E-06	1.8869	1.8877	0.7756	-0.3666	yes	down	B(i)	-	-	-	-
Arg-Tyr	1.58E-07	5.58E-07	1.8864	1.8869	0.7406	-0.4332	yes	down	B(i)	-	-	-	-
1-[(1-Ethy	1.11E-12	2.38E-11	1.8842	1.8842	0.8079	-0.3077	yes	down	B(i)	-	-	-	-
Gln-Thr-Le	4.89E-06	1.22E-05	1.8838	1.8829	1.3625	0.4462	yes	up	B(i)	-	-	-	-
Val-Pro-A	4.09E-12	6.86E-11	1.8836	1.8833	0.7645	-0.3875	yes	down	B(i)	-	-	-	-
(2S,3R,4S,5	5.15E-09	2.80E-08	1.8832	1.8837	1.3243	0.4052	yes	up	B(ii)	-	-	-	-
Penitrem E	1.59E-08	7.51E-08	1.8828	1.8823	0.7932	-0.3343	yes	down	B(ii)	C20731;_	-	-	-
2-Acetami	3.37E-08	1.44E-07	1.8801	1.8798	1.3291	0.4104	yes	up	B(i)	-	-	-	-
L-Cis-4-(H	2.15E-07	7.38E-07	1.8783	1.8789	1.3298	0.4112	yes	up	B(ii)	-	-	-	-
Glu Val Va	2.74E-08	1.20E-07	1.8778	1.8785	0.7568	-0.4019	yes	down	B(i)	-	-	-	-
Glu-Trp-G	1.76E-09	1.12E-08	1.8751	1.8753	0.7883	-0.3432	yes	down	B(i)	-	-	-	-
Thr-Lys-Ile	9.30E-18	1.81E-15	1.8743	1.8742	0.7662	-0.3841	yes	down	B(i)	-	-	-	-
1-(3,4-Difl	2.38E-10	2.04E-09	1.8707	1.8703	1.2592	0.3325	yes	up	B(i)	-	-	-	-
7,8-Dihydr	7.80E-11	8.05E-10	1.87	1.8701	0.7737	-0.3701	yes	down	B(ii)	-	-	-	-
N-(4-Tert-	4.06E-09	2.29E-08	1.8698	1.8695	1.4149	0.5007	yes	up	B(i)	-	-	-	-
N-Alpha-A	1.13E-14	5.58E-13	1.869	1.8689	1.2548	0.3275	yes	up	B(i)	-	-	-	-
Thr-Ile	7.80E-15	4.05E-13	1.8651	1.8647	0.81	-0.304	yes	down	B(i)	-	-	-	-
1-(2,5-Din	5.03E-07	1.57E-06	1.8627	1.8619	0.7418	-0.4309	yes	down	B(i)	-	-	-	-
9,10-Dihyc	3.87E-15	2.28E-13	1.8604	1.8602	1.2437	0.3146	yes	up	B(ii)	C19622;_;C	-	-	-
D-Glycero	7.38E-11	7.70E-10	1.8603	1.8602	1.3087	0.3881	yes	up	B(ii)	-	-	-	-
Ile-Lys	9.12E-15	4.64E-13	1.8574	1.8573	0.7617	-0.3927	yes	down	B(i)	-	-	-	-
SI(18:2_O/	3.77E-10	3.04E-09	1.8567	1.8568	0.7532	-0.4089	yes	down	B(i)	-	-	-	-
D-Glucuro	4.20E-08	1.74E-07	1.8558	1.8559	1.3508	0.4338	yes	up	B(ii)	C00191;C1	Carbohydr	Monosacc	map01100
7-Epizucch	1.02E-07	3.79E-07	1.8549	1.8547	1.2689	0.3436	yes	up	B(ii)	-	-	-	-
Gln-Leu	1.31E-06	3.69E-06	1.854	1.8545	0.7533	-0.4087	yes	down	B(i)	-	-	-	-
11-Hydrox	8.10E-14	2.80E-12	1.8536	1.8531	1.2753	0.3508	yes	up	B(i)	-	-	-	-
L-Prolinan	2.13E-15	1.42E-13	1.8522	1.8519	1.3038	0.3828	yes	up	B(i)	C19781;_	-	-	-

Piceol	1.12E-08	5.52E-08	1.8512	1.8513	1.3425	0.4249	yes	up	B(i)	-	-	-	-
Pg(Pgf2Al	2.81E-10	2.34E-09	1.8509	1.8505	0.8037	-0.3153	yes	down	B(ii)	-	-	-	-
Pentopril	3.45E-15	2.09E-13	1.8504	1.8502	0.8111	-0.302	yes	down	B(ii)	-	-	-	-
2-[(Anilinc	3.99E-12	6.73E-11	1.8492	1.8495	1.3071	0.3864	yes	up	B(i)	-	-	-	-
Pro-Tyr	1.95E-12	3.79E-11	1.845	1.8449	0.7925	-0.3355	yes	down	B(i)	-	-	-	-
Ser-Ile	2.69E-14	1.14E-12	1.8443	1.8439	0.8053	-0.3124	yes	down	B(i)	-	-	-	-
Ornithine	1.55E-18	4.20E-16	1.8429	1.8427	1.2462	0.3175	yes	up	B(i)	C01602;CC	-;Peptides	-;Amino a	map01100
Pc(Dime(1	6.42E-07	1.95E-06	1.8429	1.8414	0.8043	-0.3142	yes	down	B(ii)	-	-	-	-
Ser-Leu	9.74E-14	3.26E-12	1.8412	1.8408	0.8117	-0.301	yes	down	B(i)	-	-	-	-
Theanine	1.59E-18	4.26E-16	1.8409	1.8407	0.7577	-0.4003	yes	down	B(i)	C01047	-	-	-
2-Hydroxy	8.95E-17	1.09E-14	1.8406	1.8405	1.2045	0.2685	yes	up	B(i)	-	-	-	-
Gly-His-Ile	7.59E-11	7.87E-10	1.8394	1.8391	0.7861	-0.3473	yes	down	B(i)	-	-	-	-
Ala-Ile	5.16E-14	1.92E-12	1.8352	1.8349	0.8128	-0.299	yes	down	B(i)	-	-	-	-
Adenine	4.71E-10	3.66E-09	1.8321	1.8315	0.7792	-0.3599	yes	down	B(i)	C00147	Nucleic ac	Bases	map01100
Pyroglutar	1.45E-09	9.54E-09	1.8316	1.8312	0.7722	-0.3729	yes	down	B(i)	-	-	-	-
Gly-Glu-Le	8.97E-11	9.01E-10	1.8316	1.8314	0.7847	-0.3497	yes	down	B(i)	-	-	-	-
6Alpha-Hy	5.69E-08	2.27E-07	1.8315	1.8311	0.7406	-0.4332	yes	down	B(ii)	-	-	-	-
(3'X,5'A,9'X	2.57E-12	4.70E-11	1.8311	1.8312	0.799	-0.3237	yes	down	B(ii)	-	-	-	-
Buprenorp	1.79E-05	3.96E-05	1.8308	1.8321	0.77	-0.3772	yes	down	B(ii)	C08007	-	-	-
Kanzonol (	2.80E-11	3.40E-10	1.8296	1.8293	0.7639	-0.3885	yes	down	B(ii)	-	-	-	-
Fumagillol	1.03E-15	7.84E-14	1.8288	1.8287	1.291	0.3685	yes	up	B(ii)	C22618;_	-	-	map01100
Ala-Tyr	2.83E-13	7.76E-12	1.8283	1.828	0.7883	-0.3432	yes	down	B(i)	-	-	-	-
Lysyl-Serir	2.26E-08	1.01E-07	1.8281	1.8285	0.7387	-0.4369	yes	down	B(i)	-	-	-	-
O-Succiny	6.88E-17	8.58E-15	1.8274	1.8273	1.2231	0.2906	yes	up	B(i)	C01118;_	-	-	map01100
Glu-Ile-Le	4.01E-14	1.58E-12	1.8273	1.8272	0.8097	-0.3045	yes	down	B(i)	-	-	-	-
Tyrosylleu	1.15E-12	2.44E-11	1.8264	1.8259	0.7926	-0.3354	yes	down	B(i)	-	-	-	-
Trp-Leu-L	3.54E-09	2.04E-08	1.8263	1.8255	0.7755	-0.3668	yes	down	B(i)	-	-	-	-
Leu-Leu	2.55E-16	2.41E-14	1.8258	1.8255	0.813	-0.2987	yes	down	B(i)	C11332	-	-	-
(3B,9R)-5-	1.75E-09	1.12E-08	1.8237	1.8241	0.7939	-0.3329	yes	down	B(ii)	-	-	-	-
Glu-Met	1.72E-11	2.26E-10	1.8207	1.8201	0.8038	-0.3151	yes	down	B(i)	-	-	-	-
Lactarolide	2.52E-14	1.09E-12	1.82	1.8198	1.2636	0.3375	yes	up	B(i)	-	-	-	-
D-Lactose	2.05E-18	5.10E-16	1.8194	1.8192	0.7929	-0.3348	yes	down	B(i)	-	-	-	-
Dl-Leu-Dl	5.49E-14	2.02E-12	1.8174	1.817	0.8065	-0.3102	yes	down	B(i)	-	-	-	-
(R)-Piperic	1.75E-21	2.04E-18	1.8172	1.8171	1.2151	0.2811	yes	up	B(i)	-	-	-	-
Glu-Leu-S	1.09E-11	1.56E-10	1.808	1.8076	0.8069	-0.3095	yes	down	B(i)	-	-	-	-
Sabeluzole	1.36E-11	1.87E-10	1.8074	1.8075	0.7901	-0.3398	yes	down	B(ii)	-	-	-	-
Levonorde	2.21E-06	5.92E-06	1.8061	1.8068	0.7493	-0.4165	yes	down	B(i)	C11768	-	-	-
Peg Mono	6.32E-10	4.70E-09	1.8054	1.8051	0.7907	-0.3388	yes	down	B(i)	-	-	-	-

L-Isoleucy	1.39E-12	2.86E-11	1.8035	1.803	0.8064	-0.3104	yes	down	B(i)	-	-	-	-
Glu-Gln-T	1.90E-09	1.20E-08	1.8032	1.8026	0.8029	-0.3167	yes	down	B(i)	-	-	-	-
Cytochalas	1.49E-06	4.13E-06	1.8021	1.803	0.7785	-0.3612	yes	down	B(i)	-	-	-	-
Ps(6 Keto-	1.31E-05	2.98E-05	1.8021	1.8021	0.7948	-0.3313	yes	down	B(ii)	-	-	-	-
Asp-Gln-Il	1.28E-07	4.63E-07	1.8019	1.8027	0.7924	-0.3357	yes	down	B(i)	-	-	-	-
Gly-Ala	4.60E-13	1.15E-11	1.7994	1.7989	0.799	-0.3238	yes	down	B(i)	-	-	-	-
4-Benzofu	4.72E-10	3.67E-09	1.7969	1.7969	0.784	-0.351	yes	down	B(ii)	-	-	-	-
(2S)-2-[[(2	8.12E-16	6.42E-14	1.7956	1.7954	0.8335	-0.2627	yes	down	B(i)	-	-	-	-
(10Z,12E)-	0.01169	0.01626	1.7949	1.7931	1.4535	0.5395	yes	up	B(i)	-	-	-	-
Thr-Glu-G	1.08E-07	3.99E-07	1.7925	1.7926	0.7929	-0.3347	yes	down	B(i)	-	-	-	-
Glabrin D	3.13E-12	5.54E-11	1.7914	1.7914	0.8028	-0.3169	yes	down	B(ii)	-	-	-	-
Glycyl-D-L	7.43E-15	3.89E-13	1.7892	1.789	0.8191	-0.288	yes	down	B(i)	-	-	-	-
Asp Ile Phe	2.16E-11	2.74E-10	1.7854	1.7855	0.8114	-0.3016	yes	down	B(i)	-	-	-	-
Tfllr-Nh2	7.28E-09	3.78E-08	1.785	1.7844	0.7971	-0.3271	yes	down	B(ii)	-	-	-	-
N-Lactoyll	1.52E-12	3.07E-11	1.7848	1.7849	1.2679	0.3425	yes	up	B(ii)	-	-	-	-
Ketotrilost	9.18E-06	2.16E-05	1.7848	1.7846	0.7839	-0.3513	yes	down	B(ii)	-	-	-	-
Traumatic	2.12E-11	2.69E-10	1.7835	1.7833	1.2883	0.3655	yes	up	B(i)	C16308	-	-	map00592
Glu-Ile-Ile	6.00E-15	3.25E-13	1.7834	1.7831	0.8232	-0.2808	yes	down	B(i)	-	-	-	-
1-(4-Iodo	1.82E-08	8.45E-08	1.7829	1.7825	1.2506	0.3226	yes	up	B(i)	-	-	-	-
D,L-Valyl-	4.50E-16	3.92E-14	1.78	1.7797	0.8321	-0.2653	yes	down	B(i)	-	-	-	-
Val-Ser-Ile	2.33E-09	1.42E-08	1.7784	1.7786	0.774	-0.3697	yes	down	B(i)	-	-	-	-
19-Hydrox	5.22E-07	1.62E-06	1.7725	1.7728	0.7919	-0.3367	yes	down	B(ii)	-	-	-	-
Thr-Ala	1.16E-09	7.88E-09	1.7722	1.7713	0.7698	-0.3775	yes	down	B(i)	-	-	-	-
Floionolic	6.08E-17	7.83E-15	1.7708	1.7707	1.215	0.281	yes	up	B(ii)	C19621	-	-	-
Saucerneo	1.13E-10	1.10E-09	1.7704	1.7704	1.2363	0.306	yes	up	B(i)	-	-	-	-
Gln Val As	5.34E-08	2.15E-07	1.7696	1.7691	0.8166	-0.2922	yes	down	B(i)	-	-	-	-
Heptaethy	3.23E-08	1.39E-07	1.7687	1.7681	1.2677	0.3423	yes	up	B(ii)	-	-	-	-
Tyrosyl-Gl	8.58E-10	6.10E-09	1.7626	1.7621	0.7925	-0.3355	yes	down	B(i)	-	-	-	-
N5-Acetyl	1.79E-12	3.52E-11	1.7621	1.7619	0.8161	-0.2931	yes	down	B(ii)	-	-	-	-
Peg Mono	5.71E-10	4.32E-09	1.7602	1.7598	0.8236	-0.2801	yes	down	B(i)	-	-	-	-
Methyl 2-(	3.77E-18	8.45E-16	1.7586	1.7584	1.1783	0.2368	yes	up	B(i)	-	-	-	-
4-Phenylb	2.65E-07	8.85E-07	1.7582	1.7581	1.3874	0.4724	yes	up	B(i)	-	-	-	-
5-(4-Iodo	2.64E-08	1.16E-07	1.7569	1.7565	1.3464	0.4291	yes	up	B(i)	-	-	-	-
4-Hydroxy	1.34E-16	1.49E-14	1.7559	1.7558	0.8329	-0.2638	yes	down	B(i)	-	-	-	-
Glycyllysin	1.70E-07	5.97E-07	1.7522	1.7514	0.7504	-0.4142	yes	down	B(i)	-	-	-	-
N-Benzoyl	8.38E-10	5.99E-09	1.7511	1.7512	1.2735	0.3489	yes	up	B(i)	-	-	-	-
Arg-Phe	5.43E-11	5.92E-10	1.7456	1.7455	0.7957	-0.3297	yes	down	B(i)	-	-	-	-
Chenodeo	6.49E-07	1.96E-06	1.7449	1.744	0.7905	-0.3392	yes	down	B(ii)	-	-	-	-

Gln-Pro-Ly	3.89E-15	2.29E-13	1.7442	1.7439	1.2249	0.2927	yes	up	B(i)	-	-	-	-
Ile-Tyr	3.80E-11	4.37E-10	1.743	1.7424	0.7993	-0.3233	yes	down	B(i)	-	-	-	-
Fahfa(18:1	1.68E-13	5.03E-12	1.7426	1.7426	1.2696	0.3444	yes	up	B(i)	-	-	-	-
Leu-Val	3.10E-15	1.91E-13	1.7311	1.7308	0.8331	-0.2635	yes	down	B(i)	-	-	-	-
N-[[(2R,3S	6.10E-14	2.19E-12	1.7307	1.7306	1.2747	0.3501	yes	up	B(i)	-	-	-	-
Gamma-G	7.25E-07	2.16E-06	1.7284	1.7288	0.7938	-0.3331	yes	down	B(i)	C05283	-	-	-
2-Naphthy	1.36E-16	1.51E-14	1.7273	1.7271	0.8044	-0.3141	yes	down	B(ii)	-	-	-	-
Octapropy	2.32E-09	1.42E-08	1.7254	1.7249	1.2387	0.3088	yes	up	B(i)	-	-	-	-
Prosapoge	9.55E-10	6.65E-09	1.7238	1.7238	0.8239	-0.2795	yes	down	B(ii)	-	-	-	-
Lys-Leu	2.75E-14	1.16E-12	1.7232	1.723	0.8276	-0.273	yes	down	B(i)	-	-	-	-
Deoxyurid	2.92E-13	7.94E-12	1.7214	1.7213	1.2529	0.3252	yes	up	B(i)	-	-	-	-
Pro-Lys-Ile	5.56E-06	1.37E-05	1.721	1.7218	0.7967	-0.3278	yes	down	B(ii)	-	-	-	-
Apigenin	6.50E-13	1.52E-11	1.7193	1.7193	1.2286	0.2971	yes	up	B(i)	C01477	-	-	map01100
(+/-)9-Ho	1.01E-13	3.34E-12	1.7184	1.7183	1.2427	0.3134	yes	up	B(i)	-	-	-	-
Fluticasone	1.83E-05	4.04E-05	1.7182	1.718	1.2802	0.3563	yes	up	B(ii)	-	-	-	-
1-(2,4-Dih	9.36E-13	2.06E-11	1.7178	1.7176	1.2351	0.3047	yes	up	B(i)	-	-	-	-
Coq6	8.48E-10	6.04E-09	1.7175	1.7173	0.8213	-0.2841	yes	down	B(i)	C17568	-	-	-
Cholestane	2.60E-07	8.69E-07	1.7172	1.717	0.8022	-0.3179	yes	down	B(ii)	C03033;_	-	-	map01100
Flemiphilip	3.89E-12	6.62E-11	1.7148	1.7148	0.8139	-0.2971	yes	down	B(i)	-	-	-	-
2-Acetami	1.04E-09	7.15E-09	1.7081	1.7078	0.8192	-0.2878	yes	down	B(i)	-	-	-	-
Dl-Leucylg	6.19E-17	7.89E-15	1.704	1.7038	0.8414	-0.2492	yes	down	B(i)	-	-	-	-
Phe-Val-L	1.28E-07	4.63E-07	1.703	1.7032	0.7897	-0.3406	yes	down	B(i)	-	-	-	-
Val-Ile-Leu	4.86E-10	3.76E-09	1.6997	1.6994	0.818	-0.2899	yes	down	B(i)	-	-	-	-
Glu-Glu-Le	6.18E-16	5.02E-14	1.6994	1.6993	0.8385	-0.254	yes	down	B(i)	-	-	-	-
L-Leucyl-L	2.11E-16	2.04E-14	1.6987	1.6985	0.8463	-0.2407	yes	down	B(i)	-	-	-	-
(2R)-2-[[(2	3.47E-10	2.82E-09	1.6982	1.6982	0.7983	-0.325	yes	down	B(i)	-	-	-	-
Leu-Thr-Il	1.10E-15	8.19E-14	1.6974	1.6972	0.7989	-0.3239	yes	down	B(i)	-	-	-	-
4-(Trifluor	2.57E-14	1.10E-12	1.696	1.6957	0.8069	-0.3096	yes	down	B(i)	-	-	-	-
3-Methyl-	1.80E-16	1.82E-14	1.6949	1.6948	1.2073	0.2718	yes	up	B(i)	-	-	-	-
His Leu	4.10E-15	2.38E-13	1.6903	1.69	0.8303	-0.2683	yes	down	B(i)	-	-	-	-
Leukotrien	5.86E-13	1.40E-11	1.6897	1.6895	0.8266	-0.2747	yes	down	B(ii)	-	-	-	-
Gibberellin	3.52E-08	1.49E-07	1.6894	1.6891	0.8017	-0.3188	yes	down	B(ii)	C11863;_	-	-	map01100
1-(4-Ethyl	8.94E-10	6.31E-09	1.6885	1.6883	1.2954	0.3734	yes	up	B(i)	-	-	-	-
(E)-Zeatin	5.19E-12	8.37E-11	1.6838	1.6834	0.8077	-0.3082	yes	down	B(ii)	-	-	-	-
2,5-Dimetl	2.47E-10	2.11E-09	1.6834	1.6833	0.7876	-0.3445	yes	down	B(ii)	-	-	-	-
N-Cyclope	5.38E-10	4.09E-09	1.6822	1.6819	1.2167	0.283	yes	up	B(i)	-	-	-	-
11-(Dansy	1.46E-07	5.22E-07	1.6804	1.6799	1.2595	0.3329	yes	up	B(i)	-	-	-	-
5-Hydroxy	9.07E-13	2.01E-11	1.6794	1.679	0.8465	-0.2405	yes	down	B(i)	-	-	-	-

Pyrrolidine	8.83E-16	6.91E-14	1.6783	1.6782	1.2266	0.2947	yes	up	B(i)	-	-	-	-
Glu-Gly-H	1.59E-14	7.47E-13	1.6776	1.6773	0.8498	-0.2348	yes	down	B(i)	-	-	-	-
9-Formyl-	7.60E-18	1.50E-15	1.6747	1.6745	0.8043	-0.3142	yes	down	B(i)	-	-	-	-
L-Acetylca	3.36E-13	8.92E-12	1.6735	1.6735	1.2368	0.3066	yes	up	B(ii)	C02571;_	-	-	-
Ile-Gln-As	2.57E-11	3.16E-10	1.6719	1.6714	0.8347	-0.2608	yes	down	B(i)	-	-	-	-
N-(2-Hydı	1.72E-09	1.10E-08	1.6692	1.6687	1.2391	0.3093	yes	up	B(i)	-	-	-	-
Glu Asp Tl	7.83E-07	2.32E-06	1.6691	1.6683	0.8246	-0.2783	yes	down	B(i)	-	-	-	-
(2S)-2-[[(2	4.87E-14	1.83E-12	1.6672	1.667	0.8288	-0.271	yes	down	B(i)	-	-	-	-
(Z)-Narcei	1.32E-08	6.38E-08	1.6666	1.6669	1.2256	0.2935	yes	up	B(ii)	-	-	-	-
Ala-Leu	9.86E-15	4.94E-13	1.6661	1.6661	0.8356	-0.2591	yes	down	B(i)	-	-	-	-
Neomycin	8.06E-12	1.22E-10	1.6637	1.6634	1.1988	0.2616	yes	up	B(i)	C00384;CC	-;Antibioti	-;Aminogl	map01110
Leu-Gly	9.60E-17	1.14E-14	1.6621	1.6619	0.8474	-0.2388	yes	down	B(i)	C02155	-	-	-
L-Gamma-	1.39E-09	9.15E-09	1.6603	1.6601	0.8205	-0.2855	yes	down	B(i)	C05282	-	-	-
Destruxin I	3.42E-10	2.78E-09	1.6575	1.6575	0.8319	-0.2654	yes	down	B(ii)	-	-	-	-
L-Glutamiı	4.28E-15	2.46E-13	1.6566	1.6564	0.8349	-0.2603	yes	down	B(i)	C00064	Peptides	Amino aci	map01100
Phe-Pro	2.82E-17	4.36E-15	1.6535	1.6532	0.851	-0.2328	yes	down	B(i)	-	-	-	-
Fumaric Ac	3.86E-13	9.99E-12	1.6523	1.652	1.2258	0.2937	yes	up	B(i)	C00122	-	-	map01100
L-Prolinan	1.32E-13	4.17E-12	1.6498	1.6495	0.8413	-0.2494	yes	down	B(ii)	-	-	-	-
Gamma-G	2.16E-11	2.73E-10	1.6456	1.6453	1.2136	0.2793	yes	up	B(ii)	-	-	-	-
Hydroxypr	5.73E-18	1.20E-15	1.6427	1.6426	1.1775	0.2358	yes	up	B(ii)	-	-	-	-
Physangul	9.87E-08	3.69E-07	1.6424	1.6427	1.223	0.2904	yes	up	B(ii)	-	-	-	-
Proglumid	1.23E-17	2.24E-15	1.6414	1.6413	0.8498	-0.2349	yes	down	B(ii)	-	-	-	-
Dg(6 Keto-	4.52E-07	1.43E-06	1.6401	1.639	0.8193	-0.2876	yes	down	B(ii)	-	-	-	-
9(10)-Epoı	6.88E-13	1.60E-11	1.6393	1.6391	1.2017	0.265	yes	up	B(i)	C14825	-	-	map01100
Malic Acid	5.71E-16	4.70E-14	1.6385	1.6383	1.1689	0.2252	yes	up	B(i)	C00149;CC	Organic ac	Carboxylic	map01100
(2S)-2-[[(2	5.46E-15	2.99E-13	1.6383	1.6383	0.8516	-0.2318	yes	down	B(i)	-	-	-	-
Leucylprol	5.39E-17	7.12E-15	1.637	1.6367	0.8615	-0.2151	yes	down	B(i)	-	-	-	-
5-Bromop	7.23E-10	5.27E-09	1.6359	1.6359	1.2092	0.2741	yes	up	B(i)	-	-	-	-
4-(Glutam	1.60E-07	5.66E-07	1.6338	1.6339	0.8147	-0.2957	yes	down	B(ii)	C15767	-	-	map01100
2-((3-Hyd	1.86E-06	5.04E-06	1.628	1.6278	0.7981	-0.3254	yes	down	B(i)	-	-	-	-
Glu-Val-Le	1.42E-14	6.81E-13	1.6277	1.6275	0.8595	-0.2185	yes	down	B(i)	-	-	-	-
Linatine	1.98E-16	1.98E-14	1.627	1.6269	1.1578	0.2114	yes	up	B(ii)	C05939	-	-	map00470
Val-Gly	1.76E-13	5.23E-12	1.6238	1.6235	0.8489	-0.2364	yes	down	B(i)	-	-	-	-
Ser-Asp-L	2.12E-08	9.63E-08	1.6187	1.6185	0.8138	-0.2973	yes	down	B(i)	-	-	-	-
Isocitric Ac	2.64E-17	4.13E-15	1.6157	1.6156	1.1914	0.2526	yes	up	B(i)	C00311	Organic ac	Carboxylic	map01100
(8As)-7-(C	4.81E-10	3.73E-09	1.6156	1.6155	1.232	0.301	yes	up	B(i)	-	-	-	-
Tyr-Leu	3.92E-10	3.14E-09	1.6144	1.6144	0.8199	-0.2865	yes	down	B(i)	-	-	-	-
Pc(6 Keto-	2.04E-08	9.29E-08	1.6137	1.6129	0.8617	-0.2148	yes	down	B(ii)	-	-	-	-

Hydroxypr	1.50E-12	3.06E-11	1.6135	1.6131	1.1833	0.2428	yes	up	B(ii)	-	-	-	-
Tyr-Pro	4.11E-17	5.82E-15	1.6128	1.6126	0.8535	-0.2286	yes	down	B(i)	-	-	-	-
Genistein	8.95E-17	1.09E-14	1.6123	1.612	1.1456	0.1961	yes	up	B(i)	C06563	-	-	map01100
N-(1-Deo:	1.06E-10	1.03E-09	1.6115	1.6114	1.2152	0.2812	yes	up	B(ii)	-	-	-	-
Asp-Phe-I	1.23E-15	9.03E-14	1.6113	1.611	0.8629	-0.2127	yes	down	B(i)	-	-	-	-
Fructosylv	1.32E-11	1.82E-10	1.6106	1.6102	0.8551	-0.2258	yes	down	B(ii)	-	-	-	-
Ajugasterc	1.65E-07	5.81E-07	1.6084	1.608	1.2029	0.2666	yes	up	B(i)	C08811	-	-	-
2,3-Dihydr	4.21E-13	1.07E-11	1.6058	1.6058	1.2228	0.2902	yes	up	B(i)	C00196	-	-	map01053
Pro-Asp-S	7.89E-08	3.03E-07	1.6052	1.6049	1.2176	0.2841	yes	up	B(i)	-	-	-	-
Physapruir	5.45E-10	4.14E-09	1.6014	1.6015	0.8379	-0.2551	yes	down	B(ii)	-	-	-	-
Isovalerylg	2.69E-09	1.61E-08	1.5988	1.5981	1.199	0.2618	yes	up	B(ii)	-	-	-	-
3-Iodober	8.99E-09	4.55E-08	1.5975	1.5975	1.2069	0.2713	yes	up	B(i)	-	-	-	-
Loxistatin /	4.02E-06	1.02E-05	1.597	1.5963	0.7767	-0.3645	yes	down	B(i)	-	-	-	-
D-Valyl-A:	5.36E-19	1.67E-16	1.5953	1.5951	0.8392	-0.253	yes	down	B(i)	-	-	-	-
N6-Galact	1.96E-12	3.80E-11	1.5953	1.5951	1.1942	0.2561	yes	up	B(ii)	-	-	-	-
1-O-All-T	3.63E-14	1.44E-12	1.5945	1.5944	0.8385	-0.2541	yes	down	B(ii)	C11061;_	-	-	-
Fludrocort	3.70E-12	6.38E-11	1.5891	1.5891	0.8574	-0.222	yes	down	B(ii)	C07004	-	-	-
N-Isobuty	5.06E-16	4.31E-14	1.5855	1.5852	0.8241	-0.2791	yes	down	B(ii)	-	-	-	-
(2E)-2-(Cy	4.50E-13	1.13E-11	1.5848	1.5846	0.832	-0.2653	yes	down	B(i)	-	-	-	-
Histidylisol	5.59E-07	1.72E-06	1.5846	1.5841	0.8294	-0.2699	yes	down	B(i)	-	-	-	-
Acetyl Pro	7.60E-17	9.32E-15	1.5834	1.5832	0.8725	-0.1967	yes	down	B(ii)	-	-	-	-
2,3,4,5-Tet	7.86E-08	3.02E-07	1.5834	1.5837	1.2324	0.3015	yes	up	B(ii)	C00450	-	-	map01100
Dexameth	2.61E-09	1.57E-08	1.5817	1.5818	0.8393	-0.2527	yes	down	B(ii)	C15643	-	-	-
1-(2-Deox	5.60E-11	6.08E-10	1.5781	1.5782	1.1865	0.2468	yes	up	B(i)	-	-	-	-
(R)-6'-O-(	7.40E-12	1.13E-10	1.5766	1.5768	0.8555	-0.2251	yes	down	B(ii)	-	-	-	-
Hericene E	9.98E-09	4.99E-08	1.5757	1.5753	1.1907	0.2519	yes	up	B(ii)	-	-	-	-
Triferuloyl	3.96E-07	1.27E-06	1.5739	1.5746	0.8395	-0.2525	yes	down	B(ii)	-	-	-	-
Hexobarbi	5.26E-11	5.78E-10	1.5718	1.5718	1.1976	0.2602	yes	up	B(ii)	C11723	-	-	-
2-Aminoo	4.09E-17	5.82E-15	1.5696	1.5695	1.1597	0.2137	yes	up	B(i)	-	-	-	-
Timegadin	1.50E-11	2.01E-10	1.5692	1.5691	1.1938	0.2556	yes	up	B(ii)	-	-	-	-
Arginyl-Pr	9.40E-08	3.54E-07	1.5676	1.5672	0.8102	-0.3036	yes	down	B(ii)	-	-	-	-
Lavendusti	3.42E-12	5.96E-11	1.5676	1.5675	1.1928	0.2544	yes	up	B(ii)	-	-	-	-
Val-Ala	2.22E-17	3.61E-15	1.5633	1.5631	0.8584	-0.2202	yes	down	B(i)	-	-	-	-
1-(1-Phen	2.57E-15	1.63E-13	1.5632	1.5631	1.1737	0.231	yes	up	B(i)	-	-	-	-
1H-Indol-:	3.57E-08	1.51E-07	1.5618	1.5619	1.2402	0.3105	yes	up	B(i)	-	-	-	-
Leu-Phe	3.92E-12	6.64E-11	1.5601	1.5602	1.1873	0.2477	yes	up	B(i)	-	-	-	-
S-(11-Oh-	2.74E-11	3.33E-10	1.5596	1.5595	0.8584	-0.2202	yes	down	B(ii)	-	-	-	-
N-(Metho:	1.82E-12	3.59E-11	1.5592	1.5592	1.183	0.2425	yes	up	B(i)	-	-	-	-

Ile-Val	3.88E-14	1.54E-12	1.5586	1.5582	0.8595	-0.2185	yes	down	B(i)	-	-	-	-
Gly-Phe	4.02E-18	8.92E-16	1.5577	1.5575	0.853	-0.2295	yes	down	B(i)	-	-	-	-
Mepenzola	2.96E-15	1.85E-13	1.5574	1.5571	0.8755	-0.1918	yes	down	B(ii)	C07818	-	-	-
Ethyl Galla	3.89E-13	1.01E-11	1.5556	1.5556	1.2156	0.2817	yes	up	B(i)	-	-	-	-
Isobutyran	1.92E-15	1.31E-13	1.5527	1.5525	1.2282	0.2965	yes	up	B(ii)	-	-	-	-
Glucitol-Ly	1.66E-13	4.98E-12	1.5516	1.5513	1.1583	0.2121	yes	up	B(ii)	-	-	-	-
Pc(Pgf2Alp	1.05E-07	3.90E-07	1.5492	1.5484	0.8661	-0.2073	yes	down	B(ii)	-	-	-	-
Leu Gly Ala	3.92E-12	6.64E-11	1.5478	1.5478	0.8521	-0.2309	yes	down	B(i)	-	-	-	-
Neurotens	7.20E-15	3.78E-13	1.5452	1.5452	1.1731	0.2303	yes	up	B(ii)	-	-	-	-
Pyroglu-V	2.21E-17	3.61E-15	1.545	1.5447	0.8793	-0.1856	yes	down	B(i)	-	-	-	-
Cyclo(Leu-	4.55E-07	1.44E-06	1.5438	1.5433	1.2716	0.3466	yes	up	B(ii)	C20519	-	-	-
4-Hydroxy	3.66E-17	5.37E-15	1.5432	1.543	0.8672	-0.2056	yes	down	B(i)	-	-	-	-
Dictyoquir	1.51E-07	5.37E-07	1.5432	1.5426	0.8023	-0.3177	yes	down	B(ii)	-	-	-	-
1,7-Bis(4-I	1.42E-07	5.10E-07	1.5429	1.5421	0.8308	-0.2674	yes	down	B(i)	-	-	-	-
Pretyrosine	8.52E-08	3.24E-07	1.5427	1.543	1.2241	0.2917	yes	up	B(ii)	C00826	-	-	map01100
31-Hydrox	8.29E-10	5.93E-09	1.5406	1.5406	0.8407	-0.2504	yes	down	B(ii)	-	-	-	-
Dl-Malic A	1.49E-14	7.09E-13	1.5396	1.5396	1.1651	0.2205	yes	up	B(i)	-	-	-	-
His-Pro	3.89E-07	1.25E-06	1.5366	1.5368	0.8464	-0.2405	yes	down	B(i)	-	-	-	-
Ile-Ile-Asp	5.82E-11	6.26E-10	1.5337	1.5332	1.1971	0.2596	yes	up	B(i)	-	-	-	-
9Alpha-Flu	4.05E-11	4.61E-10	1.5331	1.5331	1.1846	0.2444	yes	up	B(i)	-	-	-	-
O-Desmet	4.24E-10	3.35E-09	1.5301	1.53	0.859	-0.2193	yes	down	B(ii)	-	-	-	-
Threoninyl	3.64E-16	3.25E-14	1.528	1.5278	1.1287	0.1747	yes	up	B(ii)	-	-	-	-
Thr-Pro-Ty	3.63E-08	1.53E-07	1.527	1.5268	0.8474	-0.2389	yes	down	B(i)	-	-	-	-
Trp-Ile-Le	2.52E-11	3.12E-10	1.5262	1.5262	0.8349	-0.2602	yes	down	B(i)	-	-	-	-
L-1,2,3,4-T	1.46E-14	6.98E-13	1.5253	1.5252	1.1647	0.22	yes	up	B(i)	-	-	-	-
D-Glucosa	4.13E-13	1.06E-11	1.525	1.5249	0.862	-0.2143	yes	down	B(ii)	C06023	-	-	map01100
Met-Tyr	7.29E-07	2.18E-06	1.5249	1.5247	0.8266	-0.2747	yes	down	B(i)	-	-	-	-
Formyl-5-	9.73E-15	4.91E-13	1.5239	1.5238	1.1628	0.2176	yes	up	B(ii)	C05647	-	-	map00380
Glu-Gln-Ile	9.61E-08	3.60E-07	1.5224	1.522	0.8425	-0.2473	yes	down	B(i)	-	-	-	-
Sl(19:2_O/	1.32E-06	3.72E-06	1.5219	1.5211	0.8337	-0.2624	yes	down	B(i)	-	-	-	-
Golotimod	3.56E-10	2.89E-09	1.5205	1.5202	0.8456	-0.2419	yes	down	B(ii)	-	-	-	-
Scorzoside	2.59E-10	2.19E-09	1.5204	1.5202	1.1878	0.2483	yes	up	B(ii)	-	-	-	-
Pe(O-26:7	2.62E-07	8.76E-07	1.5195	1.5187	0.8569	-0.2227	yes	down	B(i)	-	-	-	-
Leu-Glu-L	2.45E-05	5.27E-05	1.5179	1.5171	0.8363	-0.2579	yes	down	B(i)	-	-	-	-
Taurocholi	2.61E-08	1.15E-07	1.5173	1.5174	0.8416	-0.2487	yes	down	B(i)	C05122	Steroids	24-Carbor	map01100
Glu-Thr-Ile	1.39E-13	4.32E-12	1.5168	1.5168	0.8579	-0.2212	yes	down	B(i)	-	-	-	-
D-Glucohe	5.50E-07	1.70E-06	1.514	1.5139	0.8763	-0.1905	yes	down	B(i)	-	-	-	-
(1Xi,3Xi)-1	3.25E-13	8.68E-12	1.5138	1.5135	1.1673	0.2232	yes	up	B(ii)	-	-	-	-

L-Isoleucir	2.28E-12	4.26E-11	1.5137	1.5135	1.174	0.2315	yes	up	B(i)	-	-	-	-
N-Acetyl-3	5.18E-16	4.36E-14	1.5135	1.5135	1.1592	0.2131	yes	up	B(ii)	-	-	-	-
D-Desthio	1.25E-10	1.19E-09	1.5134	1.5131	0.8584	-0.2203	yes	down	B(i)	-	-	-	-
D-Mannos	8.22E-13	1.86E-11	1.5132	1.5132	1.1685	0.2247	yes	up	B(i)	C00275	-	-	map01100
6-Hydroxy	4.78E-08	1.95E-07	1.5129	1.5132	1.1717	0.2286	yes	up	B(i)	C14314	-	-	map01110
(S)-4-Metl	6.76E-13	1.58E-11	1.5116	1.5117	1.2009	0.2642	yes	up	B(i)	-	-	-	-
4-(Aminor	4.22E-13	1.07E-11	1.5114	1.5112	1.1675	0.2234	yes	up	B(i)	-	-	-	-
Gingerglyc	9.21E-10	6.47E-09	1.5075	1.5076	0.865	-0.2093	yes	down	B(ii)	-	-	-	-
Argininic A	3.09E-06	8.01E-06	1.5074	1.5082	1.2076	0.2722	yes	up	B(ii)	-	-	-	-
N-Acetylai	1.43E-15	1.02E-13	1.5056	1.5055	0.8407	-0.2503	yes	down	B(ii)	-	-	-	-
Ala-Pro-A	5.62E-08	2.25E-07	1.5035	1.504	1.227	0.2951	yes	up	B(i)	-	-	-	-
N-Acetyltr	6.92E-16	5.55E-14	1.5034	1.5034	1.1687	0.2249	yes	up	B(i)	-	-	-	-
Ergocristin	9.18E-11	9.18E-10	1.4999	1.4999	1.1669	0.2226	yes	up	B(ii)	C09164;_	-	-	-
Glaucarub	9.65E-12	1.41E-10	1.4987	1.4984	0.8664	-0.2069	yes	down	B(ii)	-	-	-	-
N,N-Dime	1.49E-13	4.54E-12	1.4981	1.4979	1.1688	0.225	yes	up	B(i)	C04259	-	-	-
Draflazine	3.16E-10	2.60E-09	1.4972	1.4966	0.8733	-0.1954	yes	down	B(ii)	-	-	-	-
(3,4,5-Trih	1.01E-09	7.01E-09	1.495	1.4952	1.1787	0.2372	yes	up	B(i)	-	-	-	-
Dodeca-3,	1.70E-08	7.94E-08	1.4896	1.4902	0.8634	-0.2119	yes	down	B(ii)	-	-	-	-
3-Hydroxy	1.14E-09	7.76E-09	1.4895	1.4897	0.8578	-0.2212	yes	down	B(i)	-	-	-	-
Thr-Pro-H	1.16E-14	5.74E-13	1.4883	1.4881	1.1577	0.2112	yes	up	B(i)	-	-	-	-
4-Megasti	3.92E-07	1.26E-06	1.4879	1.4881	0.8462	-0.241	yes	down	B(ii)	-	-	-	-
Melezitose	4.76E-12	7.81E-11	1.4867	1.4863	1.1553	0.2083	yes	up	B(i)	C08243	-	-	-
Ala Gly Va	1.30E-16	1.46E-14	1.4854	1.4854	0.8682	-0.2039	yes	down	B(i)	-	-	-	-
5-Benzylac	1.03E-15	7.84E-14	1.4848	1.4846	0.869	-0.2026	yes	down	B(ii)	-	-	-	-
Cis-Aconit	7.53E-11	7.83E-10	1.4843	1.4843	1.1965	0.2588	yes	up	B(i)	C00417	Organic ac	Carboxylic	map01100
5-Hydroxy	4.29E-12	7.15E-11	1.4839	1.484	1.1801	0.239	yes	up	B(i)	-	-	-	-
N-(Metho:	1.50E-09	9.78E-09	1.4827	1.4822	1.2036	0.2674	yes	up	B(i)	-	-	-	-
3B,6A-Dih	2.29E-07	7.78E-07	1.4822	1.4816	0.8604	-0.2169	yes	down	B(ii)	-	-	-	-
2-{[1-(2-A	1.99E-16	1.98E-14	1.4822	1.4821	1.1266	0.172	yes	up	B(i)	-	-	-	-
Afn911	3.50E-09	2.02E-08	1.4818	1.4813	0.8573	-0.2221	yes	down	B(ii)	-	-	-	-
D-Malate	3.06E-15	1.89E-13	1.4817	1.4816	1.1278	0.1735	yes	up	B(i)	C00497	-	-	map01100
Phenazopy	1.05E-10	1.02E-09	1.4809	1.4812	0.8664	-0.207	yes	down	B(ii)	C07429	-	-	-
3-(Aminor	1.41E-06	3.94E-06	1.4807	1.481	1.1995	0.2624	yes	up	B(i)	-	-	-	-
Glu-Ile-As	5.20E-16	4.36E-14	1.4798	1.4797	0.8773	-0.1889	yes	down	B(i)	-	-	-	-
2-(1-{2-[(3	1.23E-11	1.72E-10	1.4793	1.479	1.1749	0.2325	yes	up	B(i)	-	-	-	-
(3S,7E,9R)-	7.03E-08	2.73E-07	1.4769	1.4763	0.8397	-0.2521	yes	down	B(ii)	-	-	-	-
Sacubitrila	7.02E-10	5.14E-09	1.4768	1.477	1.1736	0.2309	yes	up	B(ii)	-	-	-	-
Glu-Thr-A	2.52E-17	3.97E-15	1.476	1.4759	0.8794	-0.1854	yes	down	B(i)	-	-	-	-

Aminodeo	1.29E-08	6.24E-08	1.4755	1.4756	0.8603	-0.2171	yes	down	B(ii)	-	-	-	-
3-Aminoa	7.18E-15	3.78E-13	1.4754	1.4753	1.1622	0.2169	yes	up	B(i)	-	-	-	-
Filgotinib	1.29E-11	1.79E-10	1.4738	1.4735	0.8815	-0.182	yes	down	B(ii)	-	-	-	-
Asp Phe V	5.21E-09	2.83E-08	1.473	1.4729	0.8419	-0.2482	yes	down	B(i)	-	-	-	-
(3S,5R,6R)-	5.31E-10	4.05E-09	1.4728	1.4721	1.1544	0.2071	yes	up	B(ii)	C14044	-	-	-
Tyr-Asp-L	2.23E-15	1.48E-13	1.4718	1.4717	1.1422	0.1918	yes	up	B(i)	-	-	-	-
4-(Piperidi	5.14E-05	0.000105	1.4699	1.4707	0.8405	-0.2506	yes	down	B(i)	-	-	-	-
Naorn(22:6	1.71E-08	7.99E-08	1.4696	1.4692	1.1588	0.2126	yes	up	B(i)	-	-	-	-
L-Trans-A	2.93E-10	2.43E-09	1.4689	1.4687	1.1851	0.245	yes	up	B(ii)	-	-	-	-
2-Isopenty	1.62E-09	1.05E-08	1.4678	1.4674	1.2097	0.2746	yes	up	B(i)	-	-	-	-
3-(5-Isopr	1.23E-11	1.73E-10	1.466	1.4657	0.883	-0.1795	yes	down	B(i)	-	-	-	-
5-Hydroxy	1.84E-10	1.62E-09	1.4643	1.4644	1.172	0.229	yes	up	B(ii)	C05634	-	-	map01100
Trilostane	7.51E-13	1.72E-11	1.4629	1.4626	1.1521	0.2043	yes	up	B(ii)	C12580	-	-	-
Zeatin	3.38E-09	1.96E-08	1.4622	1.4619	0.8633	-0.2121	yes	down	B(i)	C00371	-	-	map01100
Tyr Ile Glu	8.75E-07	2.57E-06	1.4621	1.4614	0.8341	-0.2617	yes	down	B(i)	-	-	-	-
Bisphenol	4.06E-10	3.23E-09	1.4597	1.4593	0.8624	-0.2136	yes	down	B(ii)	C14347	-	-	-
6-Oxohexa	1.17E-13	3.75E-12	1.4596	1.4597	1.1598	0.2138	yes	up	B(i)	C06102	-	-	map01100
1-Kestose	1.29E-11	1.78E-10	1.4592	1.4591	1.142	0.1916	yes	up	B(i)	C03661	-	-	-
N-[1-(4-M	2.33E-12	4.33E-11	1.4576	1.4573	1.169	0.2253	yes	up	B(i)	-	-	-	-
D-Galacto	8.78E-16	6.90E-14	1.4561	1.4561	1.1302	0.1765	yes	up	B(i)	C00880	-	-	map01100
3-(3,4-Dih	5.91E-13	1.41E-11	1.455	1.4551	1.1493	0.2008	yes	up	B(i)	C01207	-	-	-
3-Aminon	2.21E-09	1.36E-08	1.452	1.4519	1.1951	0.2571	yes	up	B(i)	-	-	-	-
Ser-Trp-Le	1.09E-08	5.40E-08	1.4474	1.4467	0.8541	-0.2275	yes	down	B(i)	-	-	-	-
Ser-Asp-Il	1.11E-06	3.17E-06	1.447	1.4478	0.8494	-0.2356	yes	down	B(i)	-	-	-	-
Citraconic	1.51E-16	1.64E-14	1.4464	1.4463	1.1327	0.1798	yes	up	B(ii)	C02226	-	-	map01100
Ile-Met(O)	1.62E-08	7.62E-08	1.4457	1.4451	0.8627	-0.213	yes	down	B(i)	-	-	-	-
Lysylglutar	1.42E-10	1.32E-09	1.4455	1.4455	1.188	0.2485	yes	up	B(ii)	-	-	-	-
Ile-Asn	4.55E-13	1.14E-11	1.4441	1.4438	0.8849	-0.1763	yes	down	B(i)	-	-	-	-
N-(2-Hydr	1.30E-06	3.65E-06	1.4412	1.4404	1.156	0.2092	yes	up	B(ii)	-	-	-	-
N-Delta-B	7.07E-18	1.43E-15	1.4402	1.4401	0.8837	-0.1784	yes	down	B(i)	-	-	-	-
(2S,3R)-2-	4.69E-13	1.16E-11	1.4384	1.4382	0.8309	-0.2673	yes	down	B(i)	-	-	-	-
Glu-Glu	1.49E-17	2.61E-15	1.4363	1.4362	0.8953	-0.1596	yes	down	B(i)	C01425	-	-	-
Gly-Val	3.85E-14	1.53E-12	1.4354	1.4353	0.8835	-0.1787	yes	down	B(i)	-	-	-	-
Ile-Asp-Se	1.68E-12	3.35E-11	1.4343	1.4339	0.8771	-0.1892	yes	down	B(i)	-	-	-	-
Echinocyst	1.48E-09	9.69E-09	1.4343	1.434	0.8805	-0.1836	yes	down	B(i)	-	-	-	-
Phe-Val	3.85E-12	6.58E-11	1.4327	1.4324	0.8592	-0.2189	yes	down	B(i)	-	-	-	-
Gluconate	4.76E-11	5.32E-10	1.4326	1.4328	1.142	0.1915	yes	up	B(i)	C00257	Carbohydr	Monosacc	map01100
2-Hydroxy	1.62E-19	7.01E-17	1.4313	1.4311	1.1185	0.1615	yes	up	B(i)	C02630	-	-	map01100

Aminophy	5.73E-13	1.37E-11	1.4281	1.4278	0.873	-0.196	yes	down	B(i)	C07130	-	-	map01100
Methyl 6-(	1.45E-13	4.48E-12	1.4281	1.4281	1.1329	0.18	yes	up	B(i)	-	-	-	-
15-Hydrox	8.91E-13	1.98E-11	1.4279	1.4275	0.8792	-0.1858	yes	down	B(ii)	C03033;_	-	-	map01100
Ala Lys Leu	2.29E-15	1.51E-13	1.4266	1.4265	1.1361	0.1841	yes	up	B(i)	-	-	-	-
N-Lactoyl-	2.20E-17	3.61E-15	1.4265	1.4263	1.1183	0.1613	yes	up	B(i)	-	-	-	-
Ile Asn Val	1.00E-08	5.01E-08	1.4261	1.4256	0.8539	-0.2279	yes	down	B(i)	-	-	-	-
Dopamine	6.35E-11	6.77E-10	1.426	1.4261	1.151	0.2029	yes	up	B(i)	C03758	Peptides;H	Amines;Ot	map01100
Thr-Leu-C	1.69E-09	1.08E-08	1.4258	1.4258	1.1907	0.2518	yes	up	B(i)	-	-	-	-
Gln-Glu-G	3.50E-11	4.08E-10	1.4245	1.4243	0.8687	-0.2031	yes	down	B(i)	-	-	-	-
Leu-Glu-Il	7.63E-09	3.94E-08	1.4236	1.4237	0.8652	-0.2089	yes	down	B(i)	-	-	-	-
(8Ar)-2-M	6.13E-12	9.63E-11	1.4225	1.4222	0.8897	-0.1685	yes	down	B(i)	-	-	-	-
L-Argininc	1.04E-08	5.16E-08	1.4219	1.422	1.1721	0.2291	yes	up	B(ii)	-	-	-	-
3-Chloro-	3.02E-12	5.39E-11	1.4217	1.4215	0.8782	-0.1873	yes	down	B(i)	-	-	-	-
Gluconola	4.35E-12	7.24E-11	1.42	1.42	1.1466	0.1973	yes	up	B(i)	C00198	-	-	map01100
Lys Ile Asp	3.55E-09	2.04E-08	1.419	1.4184	0.8786	-0.1867	yes	down	B(i)	-	-	-	-
(2S)-2-[[(2	1.41E-10	1.31E-09	1.4183	1.418	0.8655	-0.2083	yes	down	B(i)	-	-	-	-
D-Gluconi	5.82E-12	9.24E-11	1.4179	1.4179	1.1211	0.165	yes	up	B(i)	C00257	Carbohydr	Monosacc	map01100
N-(1-Deox	6.49E-13	1.52E-11	1.4179	1.4177	0.892	-0.1648	yes	down	B(ii)	-	-	-	-
D-Galacto	8.07E-14	2.79E-12	1.4167	1.4166	0.8888	-0.1701	yes	down	B(ii)	-	-	-	-
Pc(Pgf1Alp	1.81E-07	6.31E-07	1.4163	1.4157	0.8878	-0.1716	yes	down	B(ii)	-	-	-	-
Gly-Thr-A	1.06E-15	7.98E-14	1.4161	1.416	1.1103	0.151	yes	up	B(i)	-	-	-	-
Asn Leu	1.62E-16	1.71E-14	1.4156	1.4155	0.8773	-0.1889	yes	down	B(i)	-	-	-	-
Beta-D-Gl	1.02E-13	3.35E-12	1.4148	1.4145	0.8824	-0.1805	yes	down	B(i)	C08349	-	-	-
Tezampan	1.07E-12	2.30E-11	1.4146	1.4142	0.8844	-0.1773	yes	down	B(ii)	-	-	-	-
4,8-Dimetl	2.56E-10	2.17E-09	1.4138	1.4139	1.1792	0.2378	yes	up	B(i)	-	-	-	-
2,6-Dioxo-	4.88E-07	1.53E-06	1.4135	1.4133	1.1821	0.2414	yes	up	B(i)	-	-	-	-
Zearalanol	4.08E-13	1.05E-11	1.4135	1.4134	0.89	-0.1682	yes	down	B(ii)	_;C14754	-	-	-
Norerythrc	0.000109	0.00021	1.4131	1.4122	0.8585	-0.2201	yes	down	B(ii)	-	-	-	-
Cyclosqua	8.39E-11	8.51E-10	1.4111	1.4112	0.8843	-0.1774	yes	down	B(ii)	-	-	-	-
Phe-Asp-/	1.50E-12	3.05E-11	1.4109	1.4106	1.1366	0.1847	yes	up	B(i)	-	-	-	-
Prolylgluta	1.87E-12	3.65E-11	1.4109	1.4109	1.1569	0.2102	yes	up	B(i)	-	-	-	-
Ser-Glu-Le	4.46E-17	6.15E-15	1.4104	1.4102	0.8912	-0.1662	yes	down	B(i)	-	-	-	-
Phenylalar	1.82E-15	1.27E-13	1.4103	1.4103	0.8769	-0.1895	yes	down	B(i)	-	-	-	-
Leu-Glu-A	4.02E-07	1.29E-06	1.4096	1.4091	0.8678	-0.2045	yes	down	B(i)	-	-	-	-
Momordic	1.15E-10	1.11E-09	1.4079	1.4075	0.8848	-0.1766	yes	down	B(ii)	-	-	-	-
Ile Glu Val	9.27E-12	1.36E-10	1.4071	1.4069	0.8619	-0.2144	yes	down	B(i)	-	-	-	-
5-(3-Phen	1.71E-13	5.10E-12	1.4057	1.4056	0.8754	-0.1919	yes	down	B(i)	-	-	-	-
SI(14:3_O/.	3.63E-08	1.53E-07	1.404	1.4038	0.858	-0.2209	yes	down	B(i)	-	-	-	-

Dihydroerg	0.00022	0.000399	1.4018	1.4004	0.8526	-0.23	yes	down	B(ii)	-	-	-	-
L-Asparag	2.10E-12	4.00E-11	1.4017	1.4015	0.8673	-0.2053	yes	down	B(i)	-	-	-	-
N-(Phenox	2.44E-12	4.50E-11	1.4015	1.4014	0.8709	-0.1994	yes	down	B(i)	-	-	-	-
Glycyl-L-H	1.04E-10	1.02E-09	1.4013	1.4013	1.1527	0.2051	yes	up	B(ii)	-	-	-	-
Histidylglu	1.43E-13	4.41E-12	1.4008	1.4007	1.1217	0.1656	yes	up	B(ii)	-	-	-	-
Ser-Asn-A	2.36E-11	2.95E-10	1.3993	1.3993	1.144	0.1941	yes	up	B(i)	-	-	-	-
N'-Nitrosc	7.95E-11	8.18E-10	1.3983	1.3979	1.1464	0.1971	yes	up	B(ii)	C16452	-	-	-
N2-Acetyl	6.72E-16	5.43E-14	1.3983	1.3981	1.1188	0.1619	yes	up	B(i)	C00437	-	-	map01100
2-(3-Indol	6.36E-08	2.51E-07	1.3975	1.3972	1.1721	0.2291	yes	up	B(i)	-	-	-	-
Gly-Glu-Ile	3.42E-09	1.98E-08	1.3966	1.3972	0.8667	-0.2065	yes	down	B(i)	-	-	-	-
Guanine	2.25E-16	2.16E-14	1.3965	1.3964	0.8788	-0.1864	yes	down	B(i)	C00242	Nucleic ac	Bases	map01100
5-{[(5-Met	1.05E-08	5.21E-08	1.3954	1.3952	1.1661	0.2217	yes	up	B(i)	-	-	-	-
2-Phenyla	8.85E-13	1.97E-11	1.3951	1.3951	1.1394	0.1883	yes	up	B(i)	C02505	-	-	map01100
5-Hydroxy	2.30E-08	1.03E-07	1.3942	1.3946	1.153	0.2054	yes	up	B(ii)	-	-	-	-
Migrastatir	3.31E-12	5.81E-11	1.3933	1.3931	0.8842	-0.1775	yes	down	B(i)	-	-	-	-
Isoguanos	1.81E-13	5.32E-12	1.3927	1.3924	0.8825	-0.1803	yes	down	B(i)	C08432	-	-	-
Arctiopicri	4.26E-14	1.65E-12	1.3924	1.3923	0.8893	-0.1693	yes	down	B(ii)	C09297	-	-	-
Sedoheptu	1.95E-07	6.76E-07	1.3904	1.3902	0.9002	-0.1517	yes	down	B(i)	C02076;CC	Carbohydr	Monosacc	map01100
Diosbulbir	2.17E-06	5.81E-06	1.3901	1.3906	1.1716	0.2285	yes	up	B(ii)	-	-	-	-
Leu Gln Al	4.94E-15	2.74E-13	1.3896	1.3894	0.8958	-0.1587	yes	down	B(i)	-	-	-	-
Guanosine	4.55E-12	7.54E-11	1.3893	1.3892	0.8858	-0.1749	yes	down	B(i)	C00387	Nucleic ac	Nucleoside	map01100
3-Chloro-	8.75E-05	0.000171	1.3873	1.3866	1.2028	0.2664	yes	up	B(i)	-	-	-	-
N-Acetyl-	1.32E-17	2.33E-15	1.387	1.3868	1.1154	0.1575	yes	up	B(ii)	-	-	-	-
2-Amino-	3.51E-11	4.08E-10	1.3866	1.3865	0.8845	-0.177	yes	down	B(i)	-	-	-	-
Endothion	2.99E-07	9.86E-07	1.3854	1.3862	1.1689	0.2252	yes	up	B(ii)	C18982	-	-	-
2-[(9-Benz	2.28E-11	2.86E-10	1.3845	1.3841	1.1448	0.1951	yes	up	B(i)	-	-	-	-
4-Hydroxy	5.92E-10	4.45E-09	1.3843	1.3845	1.1526	0.2049	yes	up	B(ii)	C06317	-	-	map00627
Milbemyci	9.67E-16	7.45E-14	1.3827	1.3826	0.9027	-0.1476	yes	down	B(ii)	-	-	-	-
2-Naphthy	3.26E-14	1.33E-12	1.3794	1.3793	1.1314	0.1782	yes	up	B(i)	C02227	-	-	-
2-Acetami	7.97E-12	1.21E-10	1.3793	1.3791	1.1654	0.2209	yes	up	B(i)	-	-	-	-
N-Acetyl-l	1.01E-17	1.95E-15	1.3785	1.3785	1.126	0.1712	yes	up	B(i)	-	-	-	-
Gamma-G	1.38E-15	1.00E-13	1.3785	1.3784	0.904	-0.1457	yes	down	B(i)	-	-	-	-
3-Hydroxy	1.89E-14	8.58E-13	1.3781	1.378	1.1421	0.1916	yes	up	B(i)	-	-	-	-
2-[(2-Ethy	3.39E-12	5.93E-11	1.3779	1.3778	1.1522	0.2044	yes	up	B(i)	-	-	-	-
2-[[2-(2-A	6.91E-09	3.61E-08	1.3748	1.375	0.8689	-0.2027	yes	down	B(i)	-	-	-	-
Phenylpyru	6.80E-10	5.00E-09	1.3748	1.3751	1.1528	0.2051	yes	up	B(i)	C00166	-	-	map01100
S-(Pga1)-(	7.94E-13	1.81E-11	1.3738	1.3736	0.8941	-0.1615	yes	down	B(ii)	C11304;_	-	-	-
Val-Glu	6.81E-15	3.60E-13	1.3724	1.3722	0.892	-0.1648	yes	down	B(i)	-	-	-	-

Ceanothin	1.32E-11	1.82E-10	1.3723	1.3721	0.8876	-0.172	yes	down	B(ii)	_;C10001	-	-	-
1-(2-Meth	5.07E-13	1.25E-11	1.3703	1.3703	1.1439	0.194	yes	up	B(i)	-	-	-	-
Metaxalon	5.94E-12	9.38E-11	1.3692	1.3694	1.1418	0.1913	yes	up	B(ii)	C07934	-	-	-
(4-Acetam	5.55E-09	2.99E-08	1.3678	1.368	1.1597	0.2138	yes	up	B(i)	-	-	-	-
4-Oxo-4,5	1.37E-06	3.83E-06	1.3666	1.366	1.1751	0.2328	yes	up	B(i)	-	-	-	-
(3B,16A,20	5.44E-14	2.01E-12	1.3657	1.3655	0.892	-0.1649	yes	down	B(ii)	-	-	-	-
(2S)-2-[[(2	1.64E-17	2.81E-15	1.3646	1.3645	0.8895	-0.1689	yes	down	B(i)	-	-	-	-
Perospiror	6.70E-13	1.57E-11	1.3636	1.3634	0.8823	-0.1807	yes	down	B(i)	-	-	-	-
Furaneol 4	2.04E-11	2.61E-10	1.3628	1.3629	1.1426	0.1923	yes	up	B(ii)	-	-	-	-
Goshonosi	2.45E-07	8.27E-07	1.3614	1.3605	0.8748	-0.1929	yes	down	B(ii)	-	-	-	-
2,4-Dihydr	6.95E-08	2.71E-07	1.3609	1.3602	1.1689	0.2251	yes	up	B(i)	-	-	-	-
Gln-Ile-As	3.13E-14	1.28E-12	1.3605	1.3604	0.8878	-0.1717	yes	down	B(i)	-	-	-	-
5-Acetami	3.14E-10	2.58E-09	1.3599	1.3597	1.1481	0.1992	yes	up	B(i)	-	-	-	-
Uzarigenir	5.72E-15	3.11E-13	1.3595	1.3595	0.9002	-0.1517	yes	down	B(ii)	-	-	-	-
Delta-Vale	1.00E-09	6.95E-09	1.3553	1.355	1.1454	0.1959	yes	up	B(i)	-	-	-	-
Cyclo(Glyc	1.32E-08	6.39E-08	1.3552	1.3549	0.8838	-0.1782	yes	down	B(ii)	-	-	-	-
N-Lactoyl-	1.74E-14	8.10E-13	1.3548	1.3547	1.1279	0.1736	yes	up	B(ii)	-	-	-	-
Leu-Asn	1.82E-15	1.27E-13	1.3499	1.3498	0.8963	-0.1579	yes	down	B(i)	-	-	-	-
1,3-Dimetl	4.40E-12	7.31E-11	1.3454	1.3453	1.1571	0.2106	yes	up	B(i)	-	-	-	-
His Pro His	1.66E-09	1.07E-08	1.3451	1.345	0.8908	-0.1669	yes	down	B(i)	-	-	-	-
Gamma-G	1.58E-12	3.18E-11	1.3438	1.3438	1.1355	0.1833	yes	up	B(ii)	C06114	-	-	map00460
N-Carboxy	0.000421	0.00073	1.3435	1.3423	0.869	-0.2025	yes	down	B(ii)	-	-	-	-
N(Alpha)-I	1.04E-14	5.17E-13	1.3422	1.342	0.8786	-0.1867	yes	down	B(ii)	-	-	-	-
Phe Asn Ile	2.45E-12	4.51E-11	1.3415	1.3415	0.8854	-0.1755	yes	down	B(i)	-	-	-	-
2-Dehydro	3.36E-14	1.36E-12	1.337	1.337	0.871	-0.1992	yes	down	B(ii)	C00966	-	-	map01100
3-Hexened	1.22E-07	4.43E-07	1.3368	1.3366	0.8874	-0.1723	yes	down	B(i)	-	-	-	-
N-Cyclope	4.73E-14	1.79E-12	1.3364	1.3364	0.8704	-0.2002	yes	down	B(i)	-	-	-	-
Austalide I	6.17E-09	3.27E-08	1.3362	1.3358	0.8842	-0.1775	yes	down	B(ii)	-	-	-	-
L-Phenylal	9.40E-12	1.37E-10	1.336	1.336	1.1346	0.1822	yes	up	B(i)	-	-	-	-
Leu Ser Ile	3.10E-12	5.50E-11	1.3358	1.3355	0.8803	-0.1839	yes	down	B(i)	-	-	-	-
(2S,6S)-6-	1.71E-10	1.53E-09	1.3356	1.3357	0.8825	-0.1803	yes	down	B(ii)	-	-	-	-
4,5-Seco-I	3.47E-12	6.03E-11	1.3337	1.3337	1.1388	0.1875	yes	up	B(ii)	_;C17758	-	-	map01100
Tiglylglycir	0.000123	0.000234	1.3335	1.3352	0.8535	-0.2286	yes	down	B(i)	-	-	-	-
Val-Gly-Le	2.39E-13	6.75E-12	1.3297	1.3296	0.8902	-0.1677	yes	down	B(i)	-	-	-	-
1-Methyla	2.81E-09	1.67E-08	1.3297	1.3296	0.8841	-0.1778	yes	down	B(ii)	C02494	-	-	-
N-(Phenox	3.27E-10	2.68E-09	1.3273	1.327	0.8634	-0.2118	yes	down	B(i)	-	-	-	-
17-Phenyl	1.92E-16	1.93E-14	1.3266	1.3266	0.9033	-0.1467	yes	down	B(i)	-	-	-	-
Fraxidin	1.23E-10	1.18E-09	1.3261	1.3263	1.1454	0.1958	yes	up	B(i)	C17479	-	-	-

Asp-Leu	6.55E-14	2.33E-12	1.3249	1.3245	0.8898	-0.1685	yes	down	B(i)	-	-	-	-
Petromyzo	1.15E-08	5.65E-08	1.3249	1.3248	0.8874	-0.1723	yes	down	B(ii)	-	-	-	-
(E)-Ethenc	2.44E-15	1.57E-13	1.3235	1.3234	1.0961	0.1324	yes	up	B(i)	-	-	-	-
2-{2-Oxo-	2.12E-16	2.04E-14	1.3176	1.3174	0.8873	-0.1726	yes	down	B(i)	-	-	-	-
6-(Alpha-I	3.33E-12	5.84E-11	1.3173	1.317	0.8902	-0.1678	yes	down	B(ii)	C15658	-	-	map01100
Salvianic A	9.16E-11	9.16E-10	1.3155	1.3156	1.1313	0.178	yes	up	B(i)	-	-	-	-
8-Acetyleg	5.94E-09	3.17E-08	1.3137	1.3142	1.1391	0.1879	yes	up	B(ii)	-	-	-	-
7,9-Dihydr	1.13E-12	2.42E-11	1.3125	1.3124	0.8585	-0.2201	yes	down	B(i)	-	-	-	-
Dihydroxy-	1.13E-11	1.60E-10	1.3114	1.3114	0.8996	-0.1526	yes	down	B(i)	-	-	-	-
N-(Hydrox	4.65E-10	3.63E-09	1.3105	1.3105	0.8769	-0.1895	yes	down	B(ii)	C19456	-	-	-
N-AcetyIn	1.39E-10	1.30E-09	1.31	1.3098	0.8888	-0.1701	yes	down	B(i)	C19910;CC	-;Carbohy	-;Monosac	map01100
Ile Pro Ile	1.63E-13	4.93E-12	1.3087	1.3086	0.8989	-0.1537	yes	down	B(i)	-	-	-	-
(+/-)-Fura	9.99E-13	2.18E-11	1.3087	1.3085	1.1346	0.1822	yes	up	B(i)	-	-	-	-
2-Amino-3	3.34E-13	8.89E-12	1.3084	1.3081	1.1249	0.1697	yes	up	B(i)	-	-	-	-
Glu-Ser-Tl	3.41E-12	5.95E-11	1.3083	1.308	0.8966	-0.1575	yes	down	B(i)	-	-	-	-
Leu Gly Va	7.50E-15	3.91E-13	1.308	1.3079	0.9031	-0.147	yes	down	B(i)	-	-	-	-
2-Hydroxy	2.79E-12	5.04E-11	1.3078	1.3078	1.1415	0.1909	yes	up	B(i)	-	-	-	-
Arg Asn H	1.19E-06	3.39E-06	1.3072	1.3069	0.884	-0.178	yes	down	B(i)	-	-	-	-
4-(4-Meth	1.13E-05	2.61E-05	1.3056	1.3048	0.8741	-0.1941	yes	down	B(i)	-	-	-	-
4'-Hydroxy	5.32E-14	1.97E-12	1.3044	1.3042	0.9061	-0.1423	yes	down	B(ii)	-	-	-	-
Glutaminyl	2.08E-13	6.03E-12	1.3031	1.303	1.1233	0.1677	yes	up	B(ii)	-	-	-	-
Ala-Gly-Le	4.51E-13	1.13E-11	1.303	1.3029	0.8877	-0.1718	yes	down	B(i)	-	-	-	-
Imidazolea	8.23E-14	2.84E-12	1.3026	1.3024	0.9046	-0.1446	yes	down	B(ii)	C05131	-	-	map00340
Dimethadi	3.77E-12	6.46E-11	1.3024	1.3021	0.8791	-0.1859	yes	down	B(ii)	-	-	-	-
Asp Leu Se	1.14E-13	3.66E-12	1.3024	1.3024	0.8988	-0.154	yes	down	B(i)	-	-	-	-
1-[5-(Triflu	7.41E-12	1.14E-10	1.302	1.3021	1.1199	0.1634	yes	up	B(i)	-	-	-	-
Glu-Ile-Ty	3.05E-05	6.44E-05	1.3019	1.3023	0.8768	-0.1897	yes	down	B(i)	-	-	-	-
(1-Naphth	1.57E-07	5.55E-07	1.3016	1.3017	0.8478	-0.2383	yes	down	B(i)	-	-	-	-
Casomorp	9.29E-12	1.36E-10	1.3008	1.3007	0.8882	-0.171	yes	down	B(ii)	-	-	-	-
2-(Azepan	1.40E-12	2.87E-11	1.3001	1.3	0.8843	-0.1774	yes	down	B(i)	-	-	-	-
7-Diethyla	1.11E-11	1.59E-10	1.2995	1.2991	1.1305	0.1769	yes	up	B(i)	-	-	-	-
6Alpha/Be	3.14E-13	8.40E-12	1.2985	1.2983	1.1147	0.1566	yes	up	B(i)	-	-	-	-
Ile Gln Val	6.19E-13	1.46E-11	1.2973	1.2971	0.8888	-0.1701	yes	down	B(i)	-	-	-	-
Estradiol-1	6.35E-09	3.35E-08	1.2966	1.297	1.1238	0.1684	yes	up	B(ii)	C11237;_	-	-	-
1-Methyl-	7.77E-11	8.02E-10	1.2957	1.2957	1.1372	0.1855	yes	up	B(i)	-	-	-	-
Spermidin	0.000328	0.00058	1.2949	1.2944	0.8389	-0.2534	yes	down	B(i)	C00315	-	-	map01100
3-Hydroxy	9.26E-10	6.49E-09	1.2942	1.2941	1.132	0.1789	yes	up	B(i)	-	-	-	-
Leu Asn Th	1.63E-13	4.93E-12	1.2933	1.2932	0.9012	-0.1501	yes	down	B(i)	-	-	-	-

Methyl 3-I	1.52E-10	1.40E-09	1.2906	1.2906	1.129	0.1751	yes	up	B(i)	-	-	-	-
1-Ethyl-2-	1.56E-12	3.15E-11	1.2904	1.2902	1.1266	0.1719	yes	up	B(ii)	-	-	-	-
Dihydroerg	2.47E-08	1.10E-07	1.2902	1.29	0.8953	-0.1596	yes	down	B(ii)	-	-	-	-
Glycylproly	8.88E-14	3.02E-12	1.2901	1.2897	0.903	-0.1472	yes	down	B(i)	-	-	-	-
4,6-Diamir	4.15E-18	9.04E-16	1.2879	1.2877	0.9134	-0.1307	yes	down	B(i)	C06502	-	-	-
Mammeisi	6.07E-14	2.18E-12	1.2849	1.2848	0.8953	-0.1596	yes	down	B(ii)	C09275	-	-	-
2,6-Dihydr	3.16E-12	5.60E-11	1.2844	1.2841	1.1215	0.1654	yes	up	B(i)	-	-	-	-
2-Amino-:	0.000979	0.001603	1.2836	1.2844	0.8653	-0.2087	yes	down	B(ii)	C05520	-	-	-
Ala Ile Val	2.28E-13	6.48E-12	1.2834	1.2834	0.8833	-0.1791	yes	down	B(i)	-	-	-	-
Lotaustrali	4.04E-13	1.04E-11	1.2809	1.2807	0.9194	-0.1212	yes	down	B(i)	C08334	-	-	map00460
Ser-Ile-Leu	4.12E-11	4.69E-10	1.2792	1.2792	0.8892	-0.1694	yes	down	B(i)	-	-	-	-
4'-Aminoa	2.31E-15	1.51E-13	1.2789	1.2788	1.1181	0.1611	yes	up	B(i)	-	-	-	-
Thr-Asp-L	2.94E-09	1.74E-08	1.278	1.2778	0.8861	-0.1745	yes	down	B(i)	-	-	-	-
N-Acetyl-I	2.35E-09	1.43E-08	1.278	1.2778	1.1329	0.1801	yes	up	B(i)	C03137;_	-	-	-
Gln Pro As	9.01E-13	2.00E-11	1.2777	1.2775	0.9003	-0.1515	yes	down	B(i)	-	-	-	-
Ser-Trp-Ile	1.20E-10	1.15E-09	1.2759	1.2761	0.8956	-0.1591	yes	down	B(i)	-	-	-	-
Phorbolol	4.04E-08	1.68E-07	1.2752	1.2749	0.8796	-0.185	yes	down	B(ii)	-	-	-	-
Gly Val Ala	5.33E-14	1.97E-12	1.2744	1.2744	0.9009	-0.1506	yes	down	B(i)	-	-	-	-
Trp Asn Le	7.16E-12	1.10E-10	1.274	1.2738	0.8849	-0.1763	yes	down	B(i)	-	-	-	-
N2-Fructo	2.04E-14	9.11E-13	1.2737	1.2734	0.9197	-0.1207	yes	down	B(ii)	-	-	-	-
Glu-Gly-Ile	1.06E-11	1.52E-10	1.2729	1.2727	0.899	-0.1535	yes	down	B(i)	-	-	-	-
(3-Ethyl-3	4.01E-13	1.03E-11	1.2723	1.2721	0.9214	-0.1181	yes	down	B(i)	-	-	-	-
Trigoneosi	2.42E-12	4.47E-11	1.2668	1.2667	0.9216	-0.1178	yes	down	B(ii)	-	-	-	-
Leucyl-Arg	2.49E-10	2.12E-09	1.2659	1.266	1.1169	0.1596	yes	up	B(ii)	-	-	-	-
Ile Ile Leu	3.54E-08	1.50E-07	1.2658	1.2659	0.881	-0.1828	yes	down	B(i)	-	-	-	-
Val Leu Ty	2.41E-11	2.99E-10	1.2657	1.2655	0.8844	-0.1772	yes	down	B(i)	-	-	-	-
Metaneph	1.88E-09	1.18E-08	1.2638	1.2635	1.1295	0.1756	yes	up	B(ii)	-	-	-	-
Val Tyr	6.77E-15	3.60E-13	1.2626	1.2625	0.8997	-0.1524	yes	down	B(i)	-	-	-	-
Val Asn Gl	1.80E-13	5.32E-12	1.2623	1.2622	0.9084	-0.1385	yes	down	B(i)	-	-	-	-
Glutaminyl	4.05E-14	1.59E-12	1.2619	1.2617	0.917	-0.125	yes	down	B(ii)	-	-	-	-
N-(1-Deox	9.02E-05	0.000176	1.2599	1.2589	1.1386	0.1872	yes	up	B(ii)	-	-	-	-
2-[(2-Hyd	1.08E-10	1.05E-09	1.2595	1.2595	0.8968	-0.1571	yes	down	B(i)	-	-	-	-
5-Isoxazol	1.40E-12	2.88E-11	1.2585	1.2584	1.1187	0.1619	yes	up	B(i)	-	-	-	-
(-)-N-Ace	4.57E-13	1.14E-11	1.2573	1.2572	1.1031	0.1416	yes	up	B(ii)	-	-	-	-
Ethyl Beta-	6.08E-11	6.52E-10	1.2573	1.2573	1.1244	0.1692	yes	up	B(i)	-	-	-	-
Dihydroka	1.17E-12	2.48E-11	1.2566	1.2565	1.1114	0.1524	yes	up	B(ii)	-	-	-	-
Diethyl 2-(	2.04E-12	3.91E-11	1.2561	1.2558	0.9112	-0.1341	yes	down	B(i)	-	-	-	-
1-(2-Meth	1.15E-12	2.44E-11	1.2553	1.255	1.104	0.1428	yes	up	B(i)	-	-	-	-

Vidarabine	2.62E-12	4.76E-11	1.2542	1.2539	0.9013	-0.1499	yes	down	B(i)	C07195	-	-	-
Dimethyl 4	1.62E-10	1.47E-09	1.253	1.2526	0.8998	-0.1523	yes	down	B(i)	-	-	-	-
Gallacetop	1.26E-10	1.20E-09	1.2526	1.2528	1.1249	0.1698	yes	up	B(i)	-	-	-	-
Gly-Gly-Le	4.24E-14	1.65E-12	1.2511	1.251	0.9037	-0.146	yes	down	B(i)	-	-	-	-
N1,N10-D	1.83E-11	2.38E-10	1.2505	1.2507	0.9082	-0.1389	yes	down	B(ii)	-	-	-	-
Stevioside	3.24E-06	8.35E-06	1.2499	1.2503	0.8972	-0.1566	yes	down	B(i)	C09189	-	-	map01110
Val Glu Trp	3.65E-11	4.22E-10	1.2487	1.2487	0.8886	-0.1704	yes	down	B(i)	-	-	-	-
Leucopela	5.63E-07	1.73E-06	1.2478	1.2478	0.8943	-0.1612	yes	down	B(ii)	C03648	-	-	map01100
Lenperone	8.50E-10	6.05E-09	1.2472	1.2466	0.904	-0.1456	yes	down	B(ii)	-	-	-	-
Asn-Phe	3.56E-06	9.09E-06	1.2461	1.2458	0.8906	-0.1671	yes	down	B(i)	-	-	-	-
Gly-Leu-Il	1.29E-08	6.26E-08	1.2448	1.2442	0.8898	-0.1684	yes	down	B(i)	-	-	-	-
Thunbergi	3.25E-09	1.89E-08	1.2437	1.2439	1.1188	0.162	yes	up	B(i)	-	-	-	-
Val Asp Le	3.14E-12	5.56E-11	1.2429	1.2427	0.8989	-0.1538	yes	down	B(i)	-	-	-	-
Nicotinic A	1.11E-16	1.29E-14	1.2418	1.2417	1.0894	0.1236	yes	up	B(i)	C00253	Vitamins a	Vitamins	map01100
Methoxam	1.12E-13	3.61E-12	1.2412	1.241	1.0978	0.1346	yes	up	B(ii)	C07513	-	-	-
Glu-Lys-Ile	1.28E-11	1.77E-10	1.241	1.2407	0.8944	-0.161	yes	down	B(i)	-	-	-	-
3'-Amp	3.40E-11	3.99E-10	1.2408	1.2408	0.9135	-0.1305	yes	down	B(i)	C01367	-	-	map01100
N-Arachid	2.61E-11	3.20E-10	1.2408	1.2408	1.0984	0.1355	yes	up	B(ii)	-	-	-	-
Trypanoth	3.03E-14	1.25E-12	1.2408	1.2407	0.9184	-0.1229	yes	down	B(ii)	C03170;CC	-	-	map01100
Val-Asn-S	8.30E-13	1.87E-11	1.2399	1.2397	1.1117	0.1527	yes	up	B(i)	-	-	-	-
Gly-Trp	1.69E-05	3.75E-05	1.2398	1.2402	0.8849	-0.1764	yes	down	B(i)	-	-	-	-
(-)-Alpha-	8.78E-13	1.96E-11	1.2395	1.2395	1.1199	0.1633	yes	up	B(i)	-	-	-	-
Asn-Pro	4.45E-13	1.13E-11	1.2371	1.2369	0.8899	-0.1683	yes	down	B(i)	-	-	-	-
3-{[2-(Difl	4.53E-13	1.14E-11	1.2363	1.2362	0.89	-0.1682	yes	down	B(i)	-	-	-	-
3A,4,5,9B-	3.06E-11	3.64E-10	1.2333	1.2333	1.123	0.1674	yes	up	B(i)	-	-	-	-
N2-Galact	4.22E-11	4.79E-10	1.2313	1.2311	1.1149	0.1569	yes	up	B(ii)	-	-	-	-
Cysteinylcy	5.20E-13	1.27E-11	1.2288	1.2284	0.9047	-0.1444	yes	down	B(i)	-	-	-	-
Nodularin	3.20E-10	2.62E-09	1.2282	1.2278	0.9054	-0.1433	yes	down	B(ii)	_;C15713	-	-	-
4,4'-Methy	1.59E-05	3.56E-05	1.2257	1.2258	0.8745	-0.1935	yes	down	B(i)	C14298	-	-	map00363
2-Acetyl-1	5.01E-14	1.88E-12	1.2247	1.2246	1.0917	0.1266	yes	up	B(ii)	-	-	-	-
Solasodine	1.24E-14	6.11E-13	1.2237	1.2236	0.9145	-0.1289	yes	down	B(ii)	-	-	-	-
Ile Asn Gln	1.35E-07	4.86E-07	1.2193	1.2185	0.9009	-0.1505	yes	down	B(i)	-	-	-	-
Enbucrilate	3.99E-12	6.73E-11	1.2191	1.2191	1.11	0.1505	yes	up	B(ii)	C13415	-	-	-
Val Ile Val	3.38E-11	3.97E-10	1.2189	1.2186	0.8995	-0.1528	yes	down	B(i)	-	-	-	-
Thr-Gly	3.14E-09	1.84E-08	1.2187	1.2186	0.8968	-0.1572	yes	down	B(i)	-	-	-	-
Corchorus	8.23E-09	4.21E-08	1.2182	1.218	0.9131	-0.1312	yes	down	B(ii)	-	-	-	-
2-(Benzoy	2.70E-11	3.30E-10	1.2167	1.2167	1.114	0.1558	yes	up	B(i)	-	-	-	-
Berbamine	5.46E-13	1.33E-11	1.2167	1.2164	0.9123	-0.1324	yes	down	B(ii)	_;C09357	-	-	-

Val-Ala-Ile	1.02E-13	3.37E-12	1.2166	1.2166	0.9016	-0.1494	yes	down	B(i)	-	-	-	-
Cicaprost	2.03E-09	1.26E-08	1.2166	1.2168	1.1257	0.1708	yes	up	B(ii)	-	-	-	-
Prolyl-Asp	8.66E-14	2.96E-12	1.2148	1.2146	1.0872	0.1207	yes	up	B(ii)	-	-	-	-
Asp-Leu-L	6.53E-13	1.53E-11	1.2146	1.2146	0.9168	-0.1253	yes	down	B(i)	-	-	-	-
Ile-Ile	1.50E-08	7.12E-08	1.2138	1.2139	0.8931	-0.1631	yes	down	B(i)	-	-	-	-
2-(Acetyla	2.64E-14	1.13E-12	1.2137	1.2135	1.0979	0.1347	yes	up	B(i)	-	-	-	-
3-Methyld	3.19E-13	8.54E-12	1.2129	1.2127	1.1006	0.1383	yes	up	B(ii)	C05834	-	-	map00380
O-Acetylca	1.98E-12	3.81E-11	1.2126	1.2125	1.1067	0.1463	yes	up	B(i)	C02571	-	-	-
Alpha-Ace	5.33E-09	2.88E-08	1.2125	1.212	0.9055	-0.1431	yes	down	B(ii)	-	-	-	-
Heliotridin	1.85E-14	8.44E-13	1.2125	1.2123	1.0952	0.1312	yes	up	B(ii)	_;C10321	-	-	-
Trans-2-B	6.94E-08	2.71E-07	1.2124	1.2122	0.922	-0.1171	yes	down	B(i)	-	-	-	-
Tyrosylhyc	1.30E-06	3.66E-06	1.2118	1.2117	0.8973	-0.1563	yes	down	B(ii)	-	-	-	-
D-1-[(3-C	4.45E-11	5.00E-10	1.2108	1.2109	1.1058	0.145	yes	up	B(ii)	-	-	-	-
Osmaronir	2.63E-13	7.31E-12	1.2093	1.2092	1.0826	0.1145	yes	up	B(ii)	-	-	-	-
Dl-M-Tyrc	2.12E-14	9.41E-13	1.2086	1.2085	0.9233	-0.1151	yes	down	B(i)	-	-	-	-
1-(Hexame	1.85E-08	8.54E-08	1.2077	1.2076	0.891	-0.1665	yes	down	B(i)	-	-	-	-
3,4-Dihydr	1.92E-11	2.48E-10	1.2075	1.2076	1.1069	0.1466	yes	up	B(i)	C10675	-	-	-
Pa(Lte4/I-1	5.52E-14	2.02E-12	1.2074	1.2074	0.9246	-0.113	yes	down	B(ii)	-	-	-	-
Ser-Leu-Il	1.59E-11	2.12E-10	1.2074	1.2074	0.9139	-0.1299	yes	down	B(i)	-	-	-	-
N-Cyclohe	8.40E-14	2.89E-12	1.2071	1.2071	0.9116	-0.1335	yes	down	B(i)	-	-	-	-
Ile-Leu-Ile	2.93E-11	3.52E-10	1.2069	1.2071	0.9099	-0.1362	yes	down	B(i)	-	-	-	-
Asp-Val-Il	1.67E-09	1.08E-08	1.2057	1.2053	0.9073	-0.1403	yes	down	B(i)	-	-	-	-
L-Furosine	3.39E-14	1.37E-12	1.2055	1.2056	1.1022	0.1404	yes	up	B(ii)	-	-	-	-
26-Glucos	2.25E-10	1.95E-09	1.2049	1.2047	0.9137	-0.1303	yes	down	B(ii)	-	-	-	-
Gamma-L-	5.81E-16	4.73E-14	1.2045	1.2044	0.9229	-0.1157	yes	down	B(i)	C03740	-	-	map01100
7-(4-Meth	7.13E-11	7.46E-10	1.2042	1.204	1.0881	0.1218	yes	up	B(i)	-	-	-	-
Gynocardi	4.57E-08	1.87E-07	1.2027	1.2023	0.9001	-0.1518	yes	down	B(ii)	C08331	-	-	-
Pravastatir	1.15E-10	1.11E-09	1.2024	1.2019	0.8993	-0.1531	yes	down	B(ii)	C01844;_	-	-	-
Coproporp	4.16E-09	2.34E-08	1.2023	1.2022	0.9118	-0.1333	yes	down	B(ii)	C03263	-	-	map01100
Ala-Ile-Ile	1.14E-12	2.43E-11	1.2	1.1999	0.9106	-0.1351	yes	down	B(i)	-	-	-	-
Ser-Val-Ile	2.49E-14	1.08E-12	1.1999	1.1999	0.9065	-0.1417	yes	down	B(i)	-	-	-	-
Midodrine	2.66E-09	1.59E-08	1.1992	1.1995	1.1061	0.1455	yes	up	B(ii)	C07890	-	-	-
Taurodeox	1.81E-09	1.15E-08	1.1985	1.1984	1.1021	0.1402	yes	up	B(ii)	-	-	-	-
Ser-Ser-Ile	4.82E-10	3.73E-09	1.1982	1.198	0.9097	-0.1366	yes	down	B(i)	-	-	-	-
3-Polyprer	3.49E-12	6.06E-11	1.198	1.1979	1.1037	0.1424	yes	up	B(ii)	C17559	-	-	map01100
Isoprotere	3.80E-13	9.85E-12	1.1965	1.1962	1.0872	0.1206	yes	up	B(ii)	C07056	-	-	-
7-Hydroxy	3.04E-09	1.79E-08	1.1964	1.1961	0.9096	-0.1368	yes	down	B(ii)	-	-	-	-
20-Trifluor	1.38E-11	1.88E-10	1.1963	1.1962	0.9168	-0.1253	yes	down	B(i)	-	-	-	-

Rebaudios	4.96E-11	5.50E-10	1.1962	1.1962	0.92	-0.1203	yes	down	B(ii)	-	-	-	-
4,6-Dimetl	1.66E-10	1.50E-09	1.195	1.195	1.1199	0.1634	yes	up	B(i)	-	-	-	-
Eglumegad	2.00E-14	8.97E-13	1.194	1.1939	1.1029	0.1413	yes	up	B(i)	-	-	-	-
Indole-3-/	5.52E-12	8.82E-11	1.1935	1.1936	1.1134	0.155	yes	up	B(ii)	-	-	-	-
D-(+)-Raf	6.73E-12	1.05E-10	1.1934	1.1935	1.0814	0.1129	yes	up	B(i)	C00492	-	-	map01100
L-Isoleucir	1.71E-10	1.53E-09	1.1932	1.1933	1.1193	0.1626	yes	up	B(i)	-	-	-	-
4-Pyridoxi	9.16E-12	1.35E-10	1.193	1.1928	1.1063	0.1458	yes	up	B(i)	C00847	-	-	map01100
6-Hydroxy	3.61E-08	1.52E-07	1.1928	1.1923	1.111	0.1518	yes	up	B(ii)	-	-	-	-
4-Cyclopro	3.15E-09	1.84E-08	1.1923	1.1927	1.1252	0.1702	yes	up	B(i)	-	-	-	-
2-Hydroxy	3.02E-08	1.31E-07	1.1921	1.192	1.1067	0.1463	yes	up	B(ii)	-	-	-	-
D-Valyl-L-	9.35E-16	7.27E-14	1.1913	1.1911	1.087	0.1204	yes	up	B(i)	-	-	-	-
D-(-)-Citra	4.22E-15	2.44E-13	1.191	1.1909	1.0875	0.121	yes	up	B(i)	-	-	-	-
Pectenoto:	2.48E-09	1.50E-08	1.1897	1.1895	0.9117	-0.1333	yes	down	B(ii)	-	-	-	-
3-O-Meth	2.17E-11	2.75E-10	1.1878	1.1878	1.1001	0.1376	yes	up	B(ii)	-	-	-	-
Thr Val Va	6.86E-10	5.04E-09	1.1869	1.1869	0.8989	-0.1538	yes	down	B(i)	-	-	-	-
1-Methyl-	1.15E-13	3.68E-12	1.1866	1.1865	1.1106	0.1513	yes	up	B(i)	-	-	-	-
Asp-Val-P	2.56E-13	7.15E-12	1.1864	1.1862	0.917	-0.125	yes	down	B(i)	-	-	-	-
Trp-Thr	5.30E-12	8.53E-11	1.1849	1.1846	0.9013	-0.15	yes	down	B(i)	-	-	-	-
Ile-Gln	1.20E-11	1.69E-10	1.1849	1.1848	0.9152	-0.1279	yes	down	B(i)	-	-	-	-
Hypochoe	3.14E-06	8.13E-06	1.1841	1.1848	0.9004	-0.1514	yes	down	B(ii)	-	-	-	-
2,6-Dihydr	2.58E-14	1.11E-12	1.1836	1.1835	0.9223	-0.1168	yes	down	B(i)	C00385	-	-	map01100
S-(5'-Ader	2.03E-15	1.37E-13	1.1829	1.1828	0.9085	-0.1384	yes	down	B(i)	C00021	-	-	map01100
Adenosine	6.78E-11	7.15E-10	1.1828	1.1827	0.926	-0.1109	yes	down	B(i)	C00020	Nucleic ac	Nucleotide	map01100
Befloxaton	1.28E-07	4.64E-07	1.1814	1.1812	0.911	-0.1345	yes	down	B(ii)	-	-	-	-
Phe-Phe	1.77E-11	2.32E-10	1.1809	1.1809	1.1156	0.1578	yes	up	B(i)	-	-	-	-
Pyroglutar	8.88E-13	1.98E-11	1.1796	1.1797	1.1043	0.1431	yes	up	B(ii)	-	-	-	-
Phe-Leu-I	0.000207	0.000378	1.1788	1.1774	0.8917	-0.1654	yes	down	B(i)	-	-	-	-
Lersivirine	2.58E-15	1.64E-13	1.1786	1.1786	0.9089	-0.1379	yes	down	B(ii)	-	-	-	-
Ser Leu Ala	1.35E-12	2.79E-11	1.1781	1.1781	0.91	-0.1361	yes	down	B(i)	-	-	-	-
(2E)-3-Me	1.00E-10	9.88E-10	1.1766	1.1764	0.8999	-0.1522	yes	down	B(ii)	-	-	-	-
Phe-Pro-L	1.36E-10	1.27E-09	1.1766	1.1765	0.9154	-0.1275	yes	down	B(i)	-	-	-	-
Leu Asp Va	4.59E-12	7.59E-11	1.1762	1.1762	0.9071	-0.1406	yes	down	B(i)	-	-	-	-
Melanosta	7.90E-15	4.09E-13	1.176	1.1759	0.9246	-0.113	yes	down	B(ii)	-	-	-	-
Carambox	1.91E-12	3.72E-11	1.1732	1.1733	1.0959	0.1321	yes	up	B(i)	-	-	-	-
Ptychodisc	1.75E-10	1.56E-09	1.1723	1.172	0.9184	-0.1229	yes	down	B(ii)	-	-	-	-
Histamine	2.52E-13	7.04E-12	1.1709	1.1708	1.1016	0.1396	yes	up	B(i)	C00388	Peptides;H	Amines;Ot	map01100
9-O-Acety	5.86E-14	2.12E-12	1.1704	1.1704	1.086	0.119	yes	up	B(ii)	-	-	-	-
Prostaglan	6.39E-14	2.28E-12	1.1704	1.1703	1.0897	0.124	yes	up	B(ii)	C05957	Lipids	Eicosanoid	map00590

Demethylc	9.20E-08	3.47E-07	1.1697	1.1701	1.1005	0.1382	yes	up	B(ii)	-	-	-	-
Kinetensin	5.19E-11	5.72E-10	1.169	1.1689	1.093	0.1283	yes	up	B(ii)	-	-	-	-
Phe-Ser-Il	0.000502	0.00086	1.1687	1.1699	0.8881	-0.1713	yes	down	B(i)	-	-	-	-
D-Lyxose	6.25E-11	6.67E-10	1.1678	1.1676	1.094	0.1297	yes	up	B(i)	C00476	Carbohydr	Monosacc	map00040
1-(Beta-D	1.60E-08	7.54E-08	1.1674	1.1675	1.1009	0.1387	yes	up	B(ii)	C15497	-	-	-
Schisandri	8.17E-07	2.41E-06	1.1671	1.167	0.9058	-0.1427	yes	down	B(i)	-	-	-	-
Ile-Val-Ph	1.54E-08	7.27E-08	1.1667	1.1663	0.9064	-0.1418	yes	down	B(i)	-	-	-	-
Fructosyl-l	1.51E-13	4.58E-12	1.1664	1.1661	0.929	-0.1063	yes	down	B(i)	C16488	-	-	map02060
Dl-Leucylg	4.50E-13	1.13E-11	1.1662	1.166	0.9187	-0.1223	yes	down	B(i)	-	-	-	-
5,7-Dihydr	2.37E-11	2.95E-10	1.1658	1.1657	0.9055	-0.1433	yes	down	B(i)	C10028	-	-	-
2,6-Dimetl	1.25E-11	1.75E-10	1.1648	1.1647	0.9186	-0.1226	yes	down	B(i)	-	-	-	-
Thr-Glu-Il	2.26E-11	2.84E-10	1.1638	1.1638	0.9126	-0.132	yes	down	B(i)	-	-	-	-
4,6-Dihydr	6.81E-11	7.17E-10	1.1629	1.1631	1.1129	0.1543	yes	up	B(i)	-	-	-	-
Ala-Val-Ile	1.50E-09	9.78E-09	1.1627	1.1625	0.91	-0.1361	yes	down	B(i)	-	-	-	-
Asn-Thr-L	5.05E-14	1.89E-12	1.1626	1.1623	0.9265	-0.1102	yes	down	B(i)	-	-	-	-
Metaneph	1.30E-13	4.11E-12	1.161	1.161	1.0908	0.1254	yes	up	B(ii)	C05588	-	-	map01100
3-[(3,5-Di	5.69E-14	2.08E-12	1.1602	1.1602	1.0885	0.1223	yes	up	B(i)	-	-	-	-
S-(11-Hyd	2.22E-13	6.36E-12	1.1585	1.1585	0.9257	-0.1114	yes	down	B(ii)	-	-	-	-
Acf-(Opdc	3.11E-11	3.71E-10	1.1584	1.1586	0.9237	-0.1146	yes	down	B(ii)	-	-	-	-
Ile Ile Val	2.80E-14	1.17E-12	1.1568	1.1567	0.9303	-0.1043	yes	down	B(i)	-	-	-	-
Xanthine A	0.000242	0.000436	1.156	1.1559	1.124	0.1687	yes	up	B(ii)	-	-	-	-
15-Keto La	2.46E-15	1.58E-13	1.1559	1.1559	1.0861	0.1192	yes	up	B(i)	-	-	-	-
Guanosine	8.72E-06	2.06E-05	1.1556	1.1563	0.9096	-0.1367	yes	down	B(i)	-	-	-	-
(2S)-2-Hy	1.58E-08	7.47E-08	1.1554	1.1549	1.0915	0.1263	yes	up	B(ii)	-	-	-	-
Aristoic Ac	1.16E-08	5.71E-08	1.1554	1.1554	1.108	0.148	yes	up	B(i)	-	-	-	-
Capromor	2.00E-09	1.25E-08	1.1551	1.1548	0.9123	-0.1324	yes	down	B(ii)	-	-	-	-
2-Hydroxy	5.14E-11	5.68E-10	1.1549	1.1548	1.0957	0.1318	yes	up	B(i)	-	-	-	-
N-Cyclohe	1.11E-08	5.46E-08	1.1548	1.1542	0.9123	-0.1323	yes	down	B(i)	-	-	-	-
Cyclocalop	9.60E-07	2.79E-06	1.1534	1.1529	0.9035	-0.1464	yes	down	B(ii)	-	-	-	-
2-Methyl-	1.33E-11	1.84E-10	1.1522	1.152	1.1114	0.1524	yes	up	B(ii)	-	-	-	-
Islatravir	3.51E-13	9.24E-12	1.1521	1.1519	0.9311	-0.103	yes	down	B(ii)	-	-	-	-
Ehretioside	7.14E-14	2.51E-12	1.1514	1.1512	0.9337	-0.099	yes	down	B(i)	-	-	-	-
5-(Tert-Bu	1.78E-16	1.82E-14	1.1513	1.1512	1.0813	0.1127	yes	up	B(i)	-	-	-	-
3,7-Octadi	6.07E-13	1.44E-11	1.1504	1.1505	1.09	0.1243	yes	up	B(ii)	-	-	-	-
Ala-Asp-Il	2.00E-08	9.15E-08	1.1497	1.1503	0.9089	-0.1379	yes	down	B(i)	-	-	-	-
Gly-Val-Pl	3.89E-07	1.25E-06	1.1497	1.1494	0.9109	-0.1346	yes	down	B(i)	-	-	-	-
Guanidylic	2.55E-10	2.16E-09	1.1489	1.149	0.9148	-0.1284	yes	down	B(i)	-	-	-	-
(2R,3S)-4-	2.23E-11	2.80E-10	1.1484	1.1482	1.0881	0.1218	yes	up	B(i)	-	-	-	-

Arg-Val-Il	2.31E-12	4.29E-11	1.1479	1.1477	0.9151	-0.1279	yes	down	B(i)	-	-	-	-
Indoxyl Glu	7.59E-11	7.87E-10	1.1474	1.1474	1.0913	0.1261	yes	up	B(ii)	-	-	-	-
Medicosid	2.65E-06	6.98E-06	1.1467	1.1466	0.9207	-0.1193	yes	down	B(ii)	-	-	-	-
Asp-Ile-As	2.95E-12	5.28E-11	1.1466	1.1466	0.9225	-0.1164	yes	down	B(i)	-	-	-	-
Ps(Pgf2Alp	7.71E-06	1.84E-05	1.1461	1.1455	1.1055	0.1447	yes	up	B(ii)	-	-	-	-
Cytidine 3'	4.42E-11	4.97E-10	1.1459	1.146	0.9189	-0.1219	yes	down	B(i)	C05822	-	-	map00240
Demethyla	7.65E-11	7.93E-10	1.1457	1.1456	1.0873	0.1208	yes	up	B(ii)	-	-	-	-
Milbemyci	2.91E-11	3.51E-10	1.1455	1.1452	0.9248	-0.1129	yes	down	B(ii)	-	-	-	-
L-Agarido	3.87E-08	1.62E-07	1.1454	1.1458	1.0992	0.1365	yes	up	B(ii)	-	-	-	-
Crepidiasi	4.96E-09	2.71E-08	1.1454	1.1455	1.0999	0.1373	yes	up	B(ii)	-	-	-	-
Dolichyl B-	7.30E-10	5.32E-09	1.1452	1.1451	1.0962	0.1325	yes	up	B(ii)	C01246	-	-	map01100
Galdosol	6.34E-10	4.70E-09	1.1452	1.1452	1.0931	0.1284	yes	up	B(i)	-	-	-	-
(2S)-2-[[(2	1.25E-09	8.36E-09	1.1451	1.1446	0.9106	-0.1351	yes	down	B(i)	-	-	-	-
Lys Ile Ile	5.23E-12	8.42E-11	1.1442	1.1441	0.9143	-0.1293	yes	down	B(i)	-	-	-	-
Methyl 2-I	1.53E-08	7.26E-08	1.1435	1.1433	0.9017	-0.1492	yes	down	B(i)	-	-	-	-
Glu-Val-A	1.28E-05	2.91E-05	1.1435	1.1437	0.9094	-0.137	yes	down	B(i)	-	-	-	-
Agaritinal	3.31E-09	1.93E-08	1.1434	1.1433	1.1024	0.1407	yes	up	B(ii)	-	-	-	-
His-Val	6.80E-11	7.17E-10	1.143	1.1429	1.0961	0.1324	yes	up	B(i)	-	-	-	-
L-Pyridosi	9.85E-10	6.84E-09	1.1425	1.1426	1.1005	0.1382	yes	up	B(ii)	-	-	-	-
1-Butyl-5-	1.74E-09	1.11E-08	1.1417	1.1412	1.1132	0.1547	yes	up	B(i)	-	-	-	-
Dienestrol	5.86E-06	1.43E-05	1.1414	1.1421	1.1016	0.1396	yes	up	B(i)	-	-	-	-
2-Hydroxy	2.68E-09	1.60E-08	1.1413	1.1414	0.9117	-0.1334	yes	down	B(ii)	-	-	-	-
3-(3-Fluor	1.30E-14	6.33E-13	1.1409	1.1408	1.0813	0.1128	yes	up	B(i)	-	-	-	-
3,5-Dihydr	4.08E-14	1.60E-12	1.1405	1.1404	1.0871	0.1204	yes	up	B(i)	-	-	-	-
Myricanen	2.02E-11	2.59E-10	1.1404	1.1404	0.9258	-0.1112	yes	down	B(ii)	-	-	-	-
(15A,20R)-	7.99E-10	5.74E-09	1.1394	1.1391	0.912	-0.1329	yes	down	B(ii)	-	-	-	-
5,6,7,8-Tet	5.43E-12	8.71E-11	1.1387	1.1386	1.0908	0.1254	yes	up	B(i)	-	-	-	-
2-Hydroxy	9.32E-09	4.70E-08	1.1378	1.1373	0.904	-0.1456	yes	down	B(i)	-	-	-	-
1-Vinylaze	2.92E-11	3.52E-10	1.1365	1.1364	0.9092	-0.1373	yes	down	B(i)	-	-	-	-
Gibberellir	3.94E-12	6.67E-11	1.1364	1.1365	1.0851	0.1178	yes	up	B(ii)	-	-	-	-
4-(3-Form	6.46E-12	1.01E-10	1.1358	1.1357	1.0933	0.1287	yes	up	B(i)	-	-	-	-
Cinncassio	1.79E-09	1.14E-08	1.1357	1.1355	1.087	0.1204	yes	up	B(ii)	C17641;_	-	-	-
2,4,6-Trim	0.001621	0.002564	1.1336	1.1341	0.9058	-0.1427	yes	down	B(i)	-	-	-	-
Gln-Val-Le	1.45E-09	9.53E-09	1.1333	1.1333	0.9173	-0.1245	yes	down	B(i)	-	-	-	-
Asp Val	3.84E-11	4.40E-10	1.1318	1.1319	0.9225	-0.1164	yes	down	B(i)	-	-	-	-
Ile Asn Glu	1.05E-12	2.26E-11	1.1317	1.1315	0.9209	-0.1189	yes	down	B(i)	-	-	-	-
Ile-Glu-Ile	2.68E-07	8.93E-07	1.1302	1.1307	0.9157	-0.1271	yes	down	B(i)	-	-	-	-
Tafluprost	6.35E-08	2.50E-07	1.1302	1.1299	0.9144	-0.129	yes	down	B(ii)	-	-	-	-

Opiorphin	3.58E-08	1.51E-07	1.1281	1.128	0.9167	-0.1255	yes	down	B(ii)	-	-	-	-
Asn-Ile-As	3.22E-12	5.67E-11	1.1265	1.1264	0.9299	-0.1048	yes	down	B(i)	-	-	-	-
Tyr-His-Gl	8.08E-09	4.14E-08	1.1242	1.1239	1.0908	0.1254	yes	up	B(i)	-	-	-	-
Propofol G	1.60E-10	1.46E-09	1.1221	1.122	1.0951	0.1311	yes	up	B(ii)	-	-	-	-
2-Furanme	7.22E-08	2.80E-07	1.1218	1.1217	0.9076	-0.1399	yes	down	B(i)	C20441	-	-	map00365
Exepanol	7.45E-10	5.41E-09	1.1217	1.1214	1.0934	0.1288	yes	up	B(i)	-	-	-	-
Thymidine	2.70E-10	2.27E-09	1.1211	1.1211	1.091	0.1256	yes	up	B(ii)	C00214	Nucleic ac	Nucleoside	map00240
Gln-Asn-L	2.32E-08	1.04E-07	1.1199	1.1195	0.9155	-0.1274	yes	down	B(i)	-	-	-	-
Alinamin	3.66E-08	1.54E-07	1.1196	1.1196	0.9172	-0.1246	yes	down	B(ii)	-	-	-	-
Glycyltrypt	3.85E-11	4.42E-10	1.1196	1.1193	0.9107	-0.135	yes	down	B(i)	-	-	-	-
Daidzein	1.60E-12	3.22E-11	1.1195	1.1193	1.072	0.1003	yes	up	B(i)	C10208	-	-	map01100
12,13-Dihc	1.96E-12	3.80E-11	1.1193	1.1191	1.0726	0.1011	yes	up	B(i)	C14829	-	-	map00591
Arginylthre	1.25E-08	6.07E-08	1.1191	1.1193	0.9142	-0.1294	yes	down	B(ii)	-	-	-	-
Val Ser Ph	5.03E-12	8.17E-11	1.1186	1.1183	0.9148	-0.1285	yes	down	B(i)	-	-	-	-
Leu-Glu	7.44E-12	1.14E-10	1.1185	1.1184	0.9131	-0.1312	yes	down	B(i)	-	-	-	-
Val Pro Ile	5.76E-14	2.09E-12	1.1171	1.117	0.9274	-0.1087	yes	down	B(i)	-	-	-	-
Cannabinc	9.90E-11	9.78E-10	1.1147	1.1144	1.0857	0.1186	yes	up	B(ii)	C07580	-	-	-
Ala-Val-Le	1.80E-10	1.60E-09	1.1146	1.1147	0.924	-0.114	yes	down	B(i)	-	-	-	-
Gibberellir	9.67E-11	9.58E-10	1.1143	1.114	1.083	0.115	yes	up	B(ii)	C11871;_	-	-	map01110
N-Fructos	1.30E-11	1.79E-10	1.1142	1.114	0.9392	-0.0905	yes	down	B(i)	-	-	-	-
Val Pro Va	1.24E-10	1.18E-09	1.1135	1.1135	0.9141	-0.1296	yes	down	B(i)	-	-	-	-
Alpha-Me	1.06E-10	1.03E-09	1.1135	1.1133	1.1031	0.1415	yes	up	B(ii)	C11820	-	-	-
Danshensu	1.86E-14	8.49E-13	1.1134	1.1133	1.0778	0.1081	yes	up	B(i)	C01207	-	-	-
Rhizoxin	5.66E-13	1.36E-11	1.1133	1.113	1.075	0.1044	yes	up	B(ii)	-	-	-	-
5-(4-Meth	1.93E-14	8.74E-13	1.1125	1.1123	0.9241	-0.1139	yes	down	B(i)	-	-	-	-
Leu-His-Il	1.76E-10	1.57E-09	1.1123	1.1122	0.9121	-0.1327	yes	down	B(i)	-	-	-	-
2-Benzylsu	1.48E-08	7.04E-08	1.112	1.1117	0.9108	-0.1348	yes	down	B(i)	-	-	-	-
Isoachifolic	7.23E-12	1.11E-10	1.1113	1.1111	1.0825	0.1144	yes	up	B(ii)	-	-	-	-
2,2,4,4-Tet	1.13E-11	1.60E-10	1.1111	1.1109	1.0871	0.1205	yes	up	B(i)	-	-	-	-
6,3'-Dihyd	1.83E-08	8.49E-08	1.1109	1.1106	0.9151	-0.128	yes	down	B(i)	-	-	-	-
Tyr-Tyr-Ly	1.35E-13	4.23E-12	1.1107	1.1107	0.9298	-0.105	yes	down	B(i)	-	-	-	-
9,10-Meth	7.67E-10	5.56E-09	1.1101	1.1098	0.9225	-0.1164	yes	down	B(i)	-	-	-	-
Ile Lys Ile	5.91E-11	6.34E-10	1.1096	1.1095	0.9161	-0.1264	yes	down	B(i)	-	-	-	-
3-Hydroxy	2.06E-10	1.80E-09	1.1095	1.1093	0.9153	-0.1277	yes	down	B(i)	C03351	-	-	map01220
Coagulin F	3.69E-10	2.98E-09	1.1094	1.1094	1.0844	0.1169	yes	up	B(ii)	-	-	-	-
Glu-Asn	1.31E-06	3.70E-06	1.1093	1.1091	0.9196	-0.1209	yes	down	B(i)	-	-	-	-
2-[(Dibuty	1.24E-11	1.74E-10	1.1093	1.1092	1.0884	0.1222	yes	up	B(i)	-	-	-	-
Calophym	2.01E-11	2.58E-10	1.1091	1.1092	0.925	-0.1124	yes	down	B(i)	-	-	-	-

Gamma-A	4.59E-11	5.15E-10	1.1091	1.1092	1.0885	0.1223	yes	up	B(ii)	-	-	-	-
4-Carboxy	7.06E-09	3.69E-08	1.1087	1.1088	1.1018	0.1398	yes	up	B(i)	-	-	-	-
Tyr-His-Hi	3.70E-08	1.55E-07	1.1085	1.1085	1.0947	0.1306	yes	up	B(i)	-	-	-	-
Propoxyph	2.87E-11	3.47E-10	1.1085	1.1083	0.9222	-0.1169	yes	down	B(i)	-	-	-	-
Piriprost	1.27E-10	1.21E-09	1.1083	1.1084	0.9118	-0.1332	yes	down	B(ii)	-	-	-	-
2-Keto-3-	1.91E-12	3.72E-11	1.1078	1.1078	1.068	0.0949	yes	up	B(i)	C01216	-	-	map01100
Val-Pro-Le	1.02E-09	7.03E-09	1.1072	1.1072	0.924	-0.114	yes	down	B(i)	-	-	-	-
Cichoriosic	7.44E-12	1.14E-10	1.1064	1.1064	0.932	-0.1016	yes	down	B(ii)	-	-	-	-
N-Methyl-	1.60E-11	2.13E-10	1.1064	1.1064	1.0949	0.1307	yes	up	B(i)	-	-	-	-
(S)-2-Ami	8.00E-10	5.75E-09	1.1057	1.1056	0.9136	-0.1304	yes	down	B(i)	-	-	-	-
4-Methoxy	1.38E-06	3.86E-06	1.1048	1.1044	0.9172	-0.1247	yes	down	B(i)	-	-	-	-
Olitoriusin	9.04E-11	9.07E-10	1.1048	1.1047	0.9281	-0.1077	yes	down	B(ii)	-	-	-	-
3-(1,5-Din	2.41E-10	2.06E-09	1.1048	1.1045	1.0894	0.1235	yes	up	B(i)	-	-	-	-
Validamyc	9.27E-08	3.49E-07	1.1047	1.1045	0.9221	-0.1171	yes	down	B(ii)	C12112	-	-	map01100
(2,5-Dioxo	8.24E-13	1.86E-11	1.1045	1.1045	1.074	0.103	yes	up	B(ii)	-	-	-	-
Leukotrien	3.59E-10	2.90E-09	1.1044	1.104	0.9177	-0.1239	yes	down	B(ii)	-	-	-	-
3,4-Methy	4.69E-15	2.64E-13	1.1043	1.1042	1.072	0.1003	yes	up	B(ii)	C07577	-	-	-
Leu Glu Va	1.28E-10	1.21E-09	1.1042	1.1039	0.9173	-0.1245	yes	down	B(i)	-	-	-	-
Pa(Pgf1Alp	1.53E-09	9.98E-09	1.1038	1.1034	0.9256	-0.1116	yes	down	B(ii)	-	-	-	-
2-Amino-4	2.54E-10	2.15E-09	1.1035	1.1035	1.1018	0.1398	yes	up	B(i)	-	-	-	-
Veratramir	3.28E-13	8.74E-12	1.1026	1.1026	0.9128	-0.1316	yes	down	B(ii)	C10829;_	-	-	-
Arg-Leu-Il	1.16E-10	1.11E-09	1.1025	1.1024	0.9197	-0.1208	yes	down	B(i)	-	-	-	-
N-(3,5-Dir	5.59E-11	6.07E-10	1.1024	1.1022	0.9398	-0.0895	yes	down	B(i)	-	-	-	-
Physagulir	1.09E-11	1.55E-10	1.102	1.1021	0.9286	-0.1069	yes	down	B(ii)	-	-	-	-
2-[[2-[(2-/	3.61E-08	1.52E-07	1.1019	1.1023	0.9204	-0.1196	yes	down	B(i)	-	-	-	-
4-[[Tetrahy	2.49E-13	6.98E-12	1.1015	1.1013	1.076	0.1057	yes	up	B(i)	-	-	-	-
N-Methac	1.31E-10	1.24E-09	1.1012	1.101	1.0884	0.1222	yes	up	B(ii)	-	-	-	-
N2-Succin	1.85E-09	1.17E-08	1.1007	1.1006	0.9261	-0.1108	yes	down	B(ii)	C03415	-	-	map01100
Pencolide	2.24E-13	6.40E-12	1.1003	1.1002	1.0833	0.1154	yes	up	B(i)	-	-	-	-
Lnaps(9:0/	2.82E-11	3.42E-10	1.0985	1.0986	1.0831	0.1151	yes	up	B(i)	-	-	-	-
Altiloxin B	1.19E-10	1.14E-09	1.0973	1.0973	1.0942	0.1299	yes	up	B(i)	-	-	-	-
Talinumos	5.27E-11	5.79E-10	1.0968	1.0968	0.9452	-0.0813	yes	down	B(ii)	-	-	-	-
L-2-Amino	1.63E-08	7.66E-08	1.0963	1.0967	1.1007	0.1384	yes	up	B(ii)	-	-	-	-
Riddelliine	2.95E-06	7.69E-06	1.0962	1.0955	0.9109	-0.1347	yes	down	B(ii)	-	-	-	-
N-(1-Benz	7.63E-12	1.16E-10	1.0962	1.0963	1.0738	0.1027	yes	up	B(i)	-	-	-	-
2-(1-Hydr	6.83E-08	2.67E-07	1.0948	1.0948	1.1032	0.1417	yes	up	B(i)	-	-	-	-
2,4-Diamir	3.32E-09	1.93E-08	1.0944	1.0945	1.0958	0.1319	yes	up	B(i)	C14401	-	-	map00633
Glu-Lys-Le	6.22E-12	9.76E-11	1.0943	1.0942	0.9251	-0.1123	yes	down	B(i)	-	-	-	-

Leu-Ser	1.15E-12	2.44E-11	1.0936	1.0935	0.9275	-0.1086	yes	down	B(i)	-	-	-	-
Val-Gly-Ile	1.64E-13	4.94E-12	1.0936	1.0934	0.9345	-0.0977	yes	down	B(i)	-	-	-	-
5-Formimi	2.40E-10	2.05E-09	1.0924	1.0924	1.0832	0.1153	yes	up	B(ii)	C00664	-	-	map01100
N-Acetyl-l	4.88E-15	2.71E-13	1.092	1.092	1.0792	0.1099	yes	up	B(i)	C02712	-	-	-
Tyr-Pro-Pl	5.00E-11	5.55E-10	1.0915	1.0915	0.9353	-0.0965	yes	down	B(ii)	-	-	-	-
7-Hydroxy	2.56E-12	4.68E-11	1.0909	1.0907	1.0809	0.1123	yes	up	B(ii)	-	-	-	-
Gly-Val-Le	4.65E-12	7.66E-11	1.0908	1.0908	0.9309	-0.1033	yes	down	B(i)	-	-	-	-
(S)-2-Hydr	6.29E-17	7.96E-15	1.0905	1.0905	1.0812	0.1127	yes	up	B(i)	-	-	-	-
Cadabicine	0.000372	0.000651	1.0898	1.0886	0.9163	-0.1261	yes	down	B(ii)	-	-	-	-
Isocolumb	2.01E-10	1.75E-09	1.0893	1.089	0.917	-0.1249	yes	down	B(ii)	C17508	-	-	-
6-Benzyl-1	1.46E-07	5.22E-07	1.0892	1.0887	1.0986	0.1357	yes	up	B(i)	-	-	-	-
Pro-Met	2.71E-10	2.27E-09	1.0888	1.0886	0.9176	-0.124	yes	down	B(i)	-	-	-	-
Periandrin	4.61E-12	7.61E-11	1.0879	1.0877	0.9324	-0.1009	yes	down	B(ii)	-	-	-	-
4-(2,4-Dih	2.31E-11	2.90E-10	1.0877	1.0876	0.9233	-0.1151	yes	down	B(i)	-	-	-	-
Dactimicin	1.88E-11	2.43E-10	1.0874	1.0872	0.9276	-0.1084	yes	down	B(ii)	_;C17979	-	-	-
Mannitol	5.58E-13	1.35E-11	1.0867	1.0865	1.0833	0.1155	yes	up	B(i)	C00392	Carbohydr	Monosacc	map01100
Cerebrocra	1.05E-11	1.52E-10	1.0864	1.0863	1.0749	0.1043	yes	up	B(ii)	-	-	-	-
Asn-Val-Le	5.46E-14	2.01E-12	1.086	1.0859	0.9258	-0.1113	yes	down	B(i)	-	-	-	-
(2S)-2-(Ca	5.14E-10	3.94E-09	1.0858	1.0856	1.0774	0.1076	yes	up	B(ii)	-	-	-	-
N-(Furan-	9.27E-14	3.13E-12	1.0857	1.0856	0.9184	-0.1228	yes	down	B(i)	-	-	-	-
Abacavir	5.60E-07	1.72E-06	1.0855	1.0848	1.0988	0.136	yes	up	B(ii)	_;C07624	-	-	-
2-(4-Tert-	1.42E-11	1.91E-10	1.0843	1.0844	0.9209	-0.1189	yes	down	B(i)	-	-	-	-
N-3-Hydro	8.17E-12	1.23E-10	1.0835	1.0833	1.0695	0.0969	yes	up	B(ii)	C21200	-	-	map02024
Leu Thr Va	4.46E-11	5.02E-10	1.0816	1.0815	0.9196	-0.121	yes	down	B(i)	-	-	-	-
Pe(Dime(1	9.73E-06	2.27E-05	1.0809	1.0809	0.9178	-0.1238	yes	down	B(ii)	-	-	-	-
Gly Lys Ile	3.77E-13	9.79E-12	1.0807	1.0807	0.9275	-0.1086	yes	down	B(i)	-	-	-	-
3-Chloro-	3.71E-12	6.38E-11	1.079	1.0787	0.9232	-0.1153	yes	down	B(i)	-	-	-	-
4-(2-Oxo-	7.31E-09	3.79E-08	1.0786	1.0789	1.0867	0.12	yes	up	B(i)	-	-	-	-
N-Acetylar	1.44E-11	1.94E-10	1.0776	1.0774	1.0753	0.1048	yes	up	B(ii)	C07565	-	-	-
Gibberellin	5.76E-14	2.09E-12	1.0775	1.0776	1.0708	0.0987	yes	up	B(ii)	C11862;_	-	-	map01100
D(+)-Treha	9.05E-13	2.00E-11	1.0773	1.0769	0.9385	-0.0915	yes	down	B(i)	-	-	-	-
Daidzin	1.78E-11	2.33E-10	1.0772	1.0772	0.9286	-0.1069	yes	down	B(i)	C10216;_	-	-	map01100
Arecaidine	2.62E-09	1.57E-08	1.0768	1.0763	1.0932	0.1285	yes	up	B(ii)	C10128	-	-	-
Valnemulin	6.04E-08	2.39E-07	1.076	1.0762	0.9305	-0.1039	yes	down	B(ii)	C12066;_	-	-	-
Gly Pro Ala	5.29E-10	4.04E-09	1.0735	1.0736	1.0764	0.1063	yes	up	B(i)	-	-	-	-
1-[5-(4-Fl	2.10E-07	7.22E-07	1.0733	1.0736	0.9142	-0.1295	yes	down	B(i)	-	-	-	-
Lucyoside	7.29E-11	7.61E-10	1.0729	1.0728	0.9412	-0.0874	yes	down	B(ii)	-	-	-	-
Pantoyllac	3.47E-10	2.82E-09	1.0721	1.0718	0.9325	-0.1009	yes	down	B(ii)	-	-	-	-

3-Pyridine	1.51E-12	3.06E-11	1.0711	1.0709	1.0845	0.117	yes	up	B(i)	-	-	-	-
N-[2-(Aze	8.79E-11	8.87E-10	1.0701	1.0703	0.9274	-0.1088	yes	down	B(i)	-	-	-	-
Beta-D-Fr	4.59E-12	7.59E-11	1.0701	1.0701	1.0683	0.0953	yes	up	B(i)	C02336	-	-	-
Ile-Thr-Le	4.16E-10	3.30E-09	1.07	1.0698	0.9223	-0.1167	yes	down	B(i)	-	-	-	-
Pro-Leu	2.40E-13	6.78E-12	1.0697	1.0695	0.931	-0.1032	yes	down	B(i)	-	-	-	-
Befunolol	1.15E-09	7.83E-09	1.0695	1.0692	1.0826	0.1145	yes	up	B(ii)	-	-	-	-
Vanillic Ac	1.37E-05	3.11E-05	1.0694	1.0691	0.9186	-0.1225	yes	down	B(i)	C06672	-	-	map00627
Tetraphylli	1.67E-10	1.51E-09	1.0693	1.0689	0.93	-0.1047	yes	down	B(ii)	-	-	-	-
Gibberellir	5.86E-07	1.80E-06	1.0686	1.0684	0.9253	-0.1119	yes	down	B(ii)	C11867;_	-	-	map01110
Leu-Pro-L	2.68E-10	2.25E-09	1.0682	1.0682	0.9288	-0.1066	yes	down	B(i)	-	-	-	-
Omadacyc	3.21E-09	1.88E-08	1.068	1.0681	1.0797	0.1106	yes	up	B(ii)	-	-	-	-
Dihydrocy	1.36E-11	1.86E-10	1.0678	1.0676	1.0681	0.0951	yes	up	B(ii)	-	-	-	-
Salmefamo	9.41E-11	9.37E-10	1.067	1.0672	1.0739	0.1028	yes	up	B(ii)	C11771	-	-	-
Maltoxazir	3.72E-13	9.70E-12	1.0669	1.0669	1.0816	0.1131	yes	up	B(ii)	-	-	-	-
Phenylbuty	1.40E-09	9.25E-09	1.0652	1.0654	0.9252	-0.1122	yes	down	B(ii)	-	-	-	-
1-Acetyl-E	1.73E-13	5.16E-12	1.0645	1.0645	1.0721	0.1005	yes	up	B(i)	-	-	-	-
Val-Asp-Il	8.58E-14	2.94E-12	1.0645	1.0644	0.9338	-0.0988	yes	down	B(i)	-	-	-	-
Asp-Val-A	1.18E-13	3.75E-12	1.0644	1.0642	0.9272	-0.1091	yes	down	B(i)	-	-	-	-
Glutaminyl	1.84E-08	8.53E-08	1.0641	1.0643	1.0849	0.1176	yes	up	B(ii)	-	-	-	-
Asp-Leu-S	8.59E-13	1.92E-11	1.0636	1.0635	0.938	-0.0923	yes	down	B(i)	-	-	-	-
Jenamidine	6.38E-16	5.16E-14	1.0623	1.0622	1.0709	0.0988	yes	up	B(ii)	-	-	-	-
Eicosadien	1.05E-06	3.02E-06	1.062	1.0618	0.9229	-0.1157	yes	down	B(i)	C16525	Lipids	Fatty acids	map01040
7Alpha-Hy	8.98E-07	2.63E-06	1.062	1.0619	1.0885	0.1224	yes	up	B(ii)	-	-	-	-
Glycyl-Glu	1.88E-10	1.66E-09	1.0615	1.0611	0.9228	-0.116	yes	down	B(i)	-	-	-	-
N-Acetyl-I	8.42E-11	8.53E-10	1.0609	1.0608	1.0732	0.1019	yes	up	B(i)	-	-	-	-
3,4-Dihydr	1.95E-09	1.22E-08	1.0609	1.0607	1.0984	0.1354	yes	up	B(i)	C01161	-	-	map01100
Val-Phe-S	1.62E-10	1.47E-09	1.0604	1.0603	0.9272	-0.1091	yes	down	B(i)	-	-	-	-
Pro-Ile	2.27E-07	7.72E-07	1.06	1.0603	1.084	0.1164	yes	up	B(ii)	-	-	-	-
Octopine	1.83E-10	1.62E-09	1.0596	1.0597	1.0725	0.101	yes	up	B(ii)	C04137;_	-	-	map01100
Notoginse	4.16E-09	2.34E-08	1.0593	1.0595	0.9375	-0.0932	yes	down	B(ii)	-	-	-	-
Cyclo(-L-S	2.31E-10	1.99E-09	1.058	1.0576	0.9248	-0.1129	yes	down	B(i)	-	-	-	-
3-Hydroxy	8.93E-12	1.32E-10	1.0578	1.0577	1.0826	0.1146	yes	up	B(i)	-	-	-	-
Glu-Trp-Ile	2.82E-09	1.68E-08	1.0576	1.0576	0.9305	-0.104	yes	down	B(i)	-	-	-	-
Val-Val-Le	2.18E-11	2.75E-10	1.0569	1.0567	0.9263	-0.1105	yes	down	B(i)	-	-	-	-
L-Beta-Hc	6.29E-11	6.72E-10	1.0555	1.0555	1.0777	0.108	yes	up	B(i)	-	-	-	-
Gallacetop	2.94E-13	7.98E-12	1.0543	1.0543	1.0721	0.1005	yes	up	B(i)	-	-	-	-
L-Alanyl-L	6.55E-10	4.85E-09	1.0543	1.0542	0.9184	-0.1228	yes	down	B(i)	-	-	-	-
Dalargin	1.19E-13	3.79E-12	1.0538	1.0537	0.9418	-0.0864	yes	down	B(ii)	-	-	-	-

Coutaric A	2.99E-13	8.06E-12	1.0538	1.0537	1.0702	0.0979	yes	up	B(ii)	C01327	-	-	-
2-Hydroxy	1.31E-12	2.73E-11	1.0537	1.0535	1.0705	0.0982	yes	up	B(ii)	-	-	-	-
Clomipran	5.77E-08	2.30E-07	1.0537	1.0535	1.0829	0.1149	yes	up	B(i)	C06918	-	-	-
16-Deacet	2.36E-06	6.28E-06	1.0531	1.0521	0.9252	-0.1122	yes	down	B(ii)	-	-	-	-
Met-Phe-(	1.75E-11	2.29E-10	1.0519	1.052	1.0781	0.1085	yes	up	B(i)	-	-	-	-
Ala-Ile-Leı	4.72E-14	1.79E-12	1.0514	1.0513	0.9386	-0.0914	yes	down	B(i)	-	-	-	-
Lys-Tyrme	2.93E-07	9.69E-07	1.0506	1.0501	0.9229	-0.1157	yes	down	B(i)	-	-	-	-
Chamissor	1.65E-11	2.18E-10	1.0496	1.0496	1.0782	0.1086	yes	up	B(ii)	-	-	-	-
Natamycir	1.41E-10	1.31E-09	1.049	1.0489	0.9332	-0.0998	yes	down	B(ii)	C08073;_	-	-	map01052
Monogala	1.28E-09	8.58E-09	1.0484	1.0477	1.0898	0.1241	yes	up	B(i)	C03692	Lipids	Glycolipids	map01100
(2R,4R)-1-	0.01066	0.0149	1.0477	1.0482	0.8956	-0.1592	yes	down	B(i)	-	-	-	-
Gly-Ala-Le	2.59E-12	4.71E-11	1.0476	1.0476	0.9299	-0.1048	yes	down	B(i)	-	-	-	-
N-Formyl-	4.02E-12	6.76E-11	1.0474	1.0472	1.082	0.1137	yes	up	B(i)	C03145	Peptides	Amino aci	map00270
Gly Leu Ph	2.31E-07	7.84E-07	1.047	1.0471	0.9267	-0.1099	yes	down	B(i)	-	-	-	-
N-(1-Deo	9.72E-12	1.42E-10	1.0468	1.0466	0.9319	-0.1017	yes	down	B(ii)	-	-	-	-
Tyr-Glu-Ly	1.18E-10	1.13E-09	1.0464	1.0462	1.0745	0.1037	yes	up	B(i)	-	-	-	-
Ile-Val-Va	8.65E-14	2.96E-12	1.046	1.0458	0.918	-0.1235	yes	down	B(ii)	-	-	-	-
Idarubicin	9.91E-09	4.96E-08	1.0459	1.0454	1.0768	0.1067	yes	up	B(ii)	-	-	-	-
Tryptopha	7.56E-12	1.15E-10	1.045	1.0448	0.9423	-0.0857	yes	down	B(ii)	-	-	-	-
2-Methoxy	3.04E-15	1.89E-13	1.0449	1.0447	1.0671	0.0937	yes	up	B(ii)	-	-	-	-
N-(2-Furo	7.98E-09	4.10E-08	1.0444	1.0443	1.0896	0.1238	yes	up	B(i)	-	-	-	-
(S)-2-(Nap	1.32E-07	4.75E-07	1.0443	1.044	1.0826	0.1145	yes	up	B(i)	-	-	-	-
Ala-Val-Pl	1.35E-06	3.79E-06	1.0405	1.0395	0.922	-0.1171	yes	down	B(i)	-	-	-	-
Simulanoq	1.05E-10	1.03E-09	1.04	1.04	1.0697	0.0972	yes	up	B(ii)	-	-	-	-
2,5-Dihydı	5.59E-10	4.24E-09	1.0395	1.0394	1.0836	0.1158	yes	up	B(i)	C05585	-	-	map01100
2-Hydroxy	1.91E-07	6.61E-07	1.0382	1.0389	1.0851	0.1179	yes	up	B(ii)	-	-	-	-
Epinephrir	9.02E-08	3.41E-07	1.0382	1.0383	1.093	0.1282	yes	up	B(i)	C00788	Hormones	Other horr	map01100
Trp-Ile	8.05E-08	3.09E-07	1.0382	1.0384	0.919	-0.1219	yes	down	B(i)	-	-	-	-
Uridine Mc	1.16E-09	7.88E-09	1.038	1.0379	0.9354	-0.0963	yes	down	B(i)	-	-	-	-
Leu Glu Pł	2.29E-09	1.40E-08	1.0377	1.0376	0.9301	-0.1046	yes	down	B(i)	-	-	-	-
Pyroglutar	9.01E-10	6.35E-09	1.0374	1.0372	1.0829	0.1149	yes	up	B(i)	-	-	-	-
Protoporp	2.01E-10	1.75E-09	1.0374	1.0373	0.9401	-0.0892	yes	down	B(ii)	C01079	-	-	map01100
Xanthosine	8.07E-13	1.83E-11	1.0373	1.0372	1.0709	0.0988	yes	up	B(i)	C01762	-	-	map01100
Gibberellir	1.29E-12	2.70E-11	1.0369	1.0369	1.0706	0.0984	yes	up	B(ii)	C11861;_	-	-	map01100
Phe-Ser-L	1.48E-06	4.12E-06	1.0367	1.0369	0.9242	-0.1138	yes	down	B(i)	-	-	-	-
(+)-Galeoı	4.22E-12	7.06E-11	1.0363	1.0361	1.0724	0.1009	yes	up	B(ii)	-	-	-	-
L-Erythror	1.50E-16	1.63E-14	1.0353	1.0352	0.9387	-0.0913	yes	down	B(i)	-	-	-	-
5-Methyl-	1.63E-05	3.64E-05	1.0352	1.0345	0.9174	-0.1244	yes	down	B(i)	-	-	-	-

1-(2,3-Dih	1.13E-09	7.69E-09	1.0335	1.0332	1.0857	0.1187	yes	up	B(ii)	-	-	-	-
3-Amino-:	1.76E-07	6.16E-07	1.0333	1.0334	1.082	0.1137	yes	up	B(ii)	C12110	-	-	-
Tomaymyc	5.08E-10	3.91E-09	1.0331	1.0331	1.0808	0.1121	yes	up	B(i)	-	-	-	-
Arg Cys Ty	1.28E-09	8.53E-09	1.0328	1.0328	0.9359	-0.0956	yes	down	B(i)	-	-	-	-
N-Phenyla	1.81E-12	3.57E-11	1.0328	1.0327	1.0674	0.0941	yes	up	B(ii)	-	-	-	-
Cay10585	2.64E-14	1.13E-12	1.0313	1.0312	1.0643	0.0898	yes	up	B(i)	-	-	-	-
5-Oxo-1-I	7.60E-10	5.52E-09	1.0312	1.0311	1.0849	0.1176	yes	up	B(i)	-	-	-	-
5-Aminois	2.41E-10	2.06E-09	1.0302	1.0301	1.0845	0.1171	yes	up	B(i)	-	-	-	-
Asn Thr As	1.27E-11	1.77E-10	1.0281	1.0279	0.9402	-0.089	yes	down	B(i)	-	-	-	-
Deoxypyric	5.06E-08	2.05E-07	1.0279	1.0275	1.077	0.107	yes	up	B(ii)	-	-	-	-
Asparaginy	6.48E-14	2.31E-12	1.0275	1.0273	1.07	0.0976	yes	up	B(ii)	-	-	-	-
2-[Methyl(	3.67E-12	6.34E-11	1.0274	1.0275	1.0743	0.1035	yes	up	B(i)	-	-	-	-
N(6)-Mon(	6.20E-07	1.89E-06	1.0273	1.027	0.9321	-0.1014	yes	down	B(ii)	-	-	-	-
1-Methylh	2.61E-13	7.26E-12	1.0265	1.0264	1.073	0.1017	yes	up	B(i)	-	-	-	-
Ala-Glu-Ile	4.28E-12	7.15E-11	1.0264	1.0264	0.939	-0.0908	yes	down	B(i)	-	-	-	-
4-[5-(3,4-	1.41E-13	4.38E-12	1.0264	1.0263	1.0628	0.0878	yes	up	B(i)	-	-	-	-
1,6-Anhyd	4.48E-12	7.43E-11	1.0256	1.0256	1.0725	0.101	yes	up	B(i)	-	-	-	-
3,6,9-Triox	1.10E-10	1.07E-09	1.0256	1.0253	1.0714	0.0995	yes	up	B(i)	-	-	-	-
Glu-His-G	2.50E-14	1.08E-12	1.0254	1.0253	0.9419	-0.0864	yes	down	B(i)	-	-	-	-
5-O-Desm	1.78E-12	3.52E-11	1.025	1.0248	1.0695	0.0969	yes	up	B(ii)	-	-	-	-
Toyocamy	6.59E-15	3.52E-13	1.0246	1.0246	1.0612	0.0857	yes	up	B(i)	-	-	-	-
Carbetami	7.23E-09	3.76E-08	1.0235	1.023	1.0837	0.1159	yes	up	B(ii)	C11075	-	-	-
Acevaltrate	2.94E-07	9.70E-07	1.0231	1.0231	0.9281	-0.1077	yes	down	B(ii)	C16752	-	-	-
Leu Glu Al	1.69E-11	2.22E-10	1.0228	1.0225	0.937	-0.0939	yes	down	B(i)	-	-	-	-
4-Hydroxy	2.41E-07	8.13E-07	1.0223	1.0226	1.0809	0.1122	yes	up	B(i)	-	-	-	-
Pro-Phe	9.56E-12	1.39E-10	1.0219	1.0219	0.9315	-0.1024	yes	down	B(i)	-	-	-	-
Alpha-Bur	3.80E-11	4.36E-10	1.0217	1.0218	0.9391	-0.0907	yes	down	B(ii)	_;C20037	-	-	-
Hophe-Gly	3.43E-10	2.79E-09	1.0217	1.0215	1.0731	0.1017	yes	up	B(i)	-	-	-	-
Pglu-Glu-	2.52E-07	8.47E-07	1.0214	1.0208	0.9321	-0.1014	yes	down	B(i)	-	-	-	-
Pyroglutar	3.92E-13	1.01E-11	1.0206	1.0205	0.9296	-0.1053	yes	down	B(ii)	-	-	-	-
Ketologan	5.27E-09	2.85E-08	1.0205	1.0204	0.9287	-0.1067	yes	down	B(i)	-	-	-	-
2-(2,5-Dia	8.20E-13	1.85E-11	1.0196	1.0195	1.0688	0.096	yes	up	B(i)	-	-	-	-
Tyr-His	1.81E-11	2.35E-10	1.0196	1.0193	1.0732	0.1019	yes	up	B(i)	-	-	-	-
S-Adenosy	6.71E-11	7.08E-10	1.0188	1.0187	0.9293	-0.1058	yes	down	B(i)	C00021	-	-	map01100
Supinidine	7.01E-12	1.08E-10	1.0183	1.0182	1.0745	0.1036	yes	up	B(ii)	C10400	-	-	-
L-4-Hydro	1.03E-12	2.23E-11	1.0179	1.0179	1.0691	0.0964	yes	up	B(ii)	C05938	-	-	map01100
N-Acetyl-I	2.37E-10	2.03E-09	1.0172	1.0174	1.0709	0.0988	yes	up	B(i)	-	-	-	-
(S)-2-Hydr	3.08E-11	3.67E-10	1.0161	1.0162	1.0636	0.0889	yes	up	B(ii)	-	-	-	-

Tetrahydro	7.01E-06	1.69E-05	1.0161	1.0157	0.9264	-0.1103	yes	down	B(ii)	-	-	-	-
Asp-Glu-L	1.70E-12	3.38E-11	1.016	1.0161	1.069	0.0963	yes	up	B(i)	-	-	-	-
Senecioic /	1.46E-10	1.35E-09	1.016	1.0162	1.0783	0.1087	yes	up	B(i)	-	-	-	-
Scopolami	4.34E-07	1.38E-06	1.0157	1.0156	1.0896	0.1239	yes	up	B(i)	C01851	-	-	map01100
2-Phenoxy	3.49E-10	2.83E-09	1.0152	1.0152	0.9279	-0.1079	yes	down	B(i)	-	-	-	-
Icaridin	2.96E-05	6.27E-05	1.0148	1.0145	0.9163	-0.1261	yes	down	B(ii)	-	-	-	-
Pregnanet	2.17E-12	4.10E-11	1.0135	1.0132	0.9356	-0.096	yes	down	B(ii)	-	-	-	-
Dihydroerg	1.50E-13	4.58E-12	1.0132	1.0131	1.0649	0.0907	yes	up	B(ii)	-	-	-	-
Goshonosi	2.33E-09	1.42E-08	1.0124	1.0123	0.9344	-0.0979	yes	down	B(ii)	-	-	-	-
5,6,7,8-Tet	2.91E-10	2.42E-09	1.0116	1.0113	1.0811	0.1125	yes	up	B(i)	-	-	-	-
2-[[(2E)-2-	4.45E-09	2.47E-08	1.0114	1.0111	1.0786	0.1091	yes	up	B(i)	-	-	-	-
Beclometh	8.68E-08	3.30E-07	1.0112	1.0112	0.9374	-0.0932	yes	down	B(i)	_;C06842	-	-	-
4-(3-Amir	4.20E-12	7.03E-11	1.0111	1.0112	1.0831	0.1152	yes	up	B(i)	-	-	-	-
Pyridoxal	5.09E-10	3.91E-09	1.0109	1.0105	1.0725	0.101	yes	up	B(ii)	C00250	Vitamins a	Vitamins	map01100
2-Methoxy	7.82E-07	2.32E-06	1.0108	1.0109	0.9238	-0.1143	yes	down	B(ii)	C11132	-	-	-
Asp-Trp-L	1.76E-12	3.48E-11	1.0097	1.0096	1.0661	0.0923	yes	up	B(i)	-	-	-	-
4-Hydroxy	4.23E-09	2.37E-08	1.0083	1.0079	0.9354	-0.0964	yes	down	B(ii)	C19565	-	-	-
Glucosyl (2	6.37E-11	6.79E-10	1.0079	1.0078	1.0692	0.0965	yes	up	B(ii)	-	-	-	-
Carteolol	4.01E-11	4.57E-10	1.0075	1.0077	1.0704	0.0981	yes	up	B(ii)	C06874	-	-	-
Janthitrem	2.37E-10	2.03E-09	1.0073	1.0074	1.0669	0.0934	yes	up	B(ii)	C20601;_	-	-	-
3-Amino-4	1.42E-09	9.36E-09	1.0066	1.0065	0.9138	-0.13	yes	down	B(i)	C12115	-	-	map01100
Silodosin	3.87E-11	4.44E-10	1.0063	1.006	1.0669	0.0934	yes	up	B(ii)	-	-	-	-
Adipic Acid	4.49E-13	1.13E-11	1.0054	1.0055	1.0642	0.0898	yes	up	B(i)	C06104	Organic ac	Carboxylic	map01100
6-(4-Morp	6.52E-11	6.91E-10	1.0051	1.0047	0.9343	-0.0981	yes	down	B(i)	-	-	-	-
Ethyl 1-An	1.86E-15	1.28E-13	1.0046	1.0045	1.0564	0.0792	yes	up	B(i)	-	-	-	-
2-[1-(2-O	1.49E-12	3.04E-11	1.0046	1.0043	0.9424	-0.0856	yes	down	B(i)	-	-	-	-
Ala-Glu	2.77E-13	7.62E-12	1.0036	1.0036	1.0667	0.0931	yes	up	B(i)	-	-	-	-
Neplanocin	3.06E-12	5.44E-11	1.0034	1.0034	1.0678	0.0946	yes	up	B(ii)	-	-	-	-
Glu Ile Arg	7.67E-11	7.94E-10	1.0026	1.0026	0.9471	-0.0784	yes	down	B(i)	-	-	-	-
Val-Leu-Ile	1.05E-13	3.43E-12	1.0008	1.0006	0.9445	-0.0823	yes	down	B(i)	-	-	-	-
4-O-Meth	1.00E-08	5.02E-08	1.0006	1.0008	1.0715	0.0997	yes	up	B(ii)	-	-	-	-

KEGG Path	KEGG Path	KEGG Path	Library ID	HMDB Sup	HMDB Cla	HMDB Sub	CAS ID	M/Z	Retention	Adducts	Formula	Fragmenta	Theoretica
-	-	-	-	-	-	-		316.1288	4.1718	M+H	C16H17N3	93.9	0
-	-	-	HMDB002	Organic ac	Carboxylic	Amino acids, peptides		635.2612	4.8436	2M-H	C15H18N4	0	59.1
-	-	-	HMDB002	Organic ac	Carboxylic	Amino acids, peptides		318.1809	3.9657	M+H	C17H23N3	90.6	0
-	-	-	HMDB001	Organic ac	Carboxylic	Amino acid	126222-34	651.3255	5.414	M+Na-2H	C33H50N4	0	70.7
-	-	-	-;HMDB02	Phenylprop	Flavonoids	Flavans	-;_;	480.2338	5.5282	M+ACN+H	C26H30O6	35.1	0
-	-	-	HMDB030	Lipids and	Prenol lipid	Terpene lactones		393.1883	3.4189	M-H2O-H	C21H32O8	0	77.8
-	-	-	HMDB003	Organohet	Pyrans	Pyranones	74145-79-	480.2342	5.0596	M+ACN+N	C24H32O6	0	41.7
-	-	-	HMDB003	Organic ox	Organooxy	Alcohols a	100288-36	305.1704	3.4008	M+CH3OH	C15H22O3	0	74.5
-	-	-	HMDB002	Lipids and	Prenol lipid	Terpene gl	69809-29-	377.1932	4.0751	M-H2O-H	C21H32O7	0	69.9
-	-	-	-	-	-	-		229.0886	4.6149	M-H	C14H14O3	54.3	0
-	-	-	HMDB025	Organohet	Naphthopy	Not Availa	78213-64-	602.305	4.6683	M+Cl	C37H45NO	0	55
-	-	-	-	-	-	-	508-65-6	532.286	3.6238	M+Na	C27H43NO	47.2	0
-	-	-	HMDB003	Organic ox	Organooxy	Carbohydr	29774-74-	379.1725	3.4036	M-H	C20H28O7	0	83.5
-	-	-	HMDB003	Organohet	Benzofuran	Not Availa	271-89-6	117.0335	4.7677	M-H	C8H6O	44.7	0
-	-	-	HMDB001	Phenylprop	Tetracyclin	Not Availa	992-21-2	637.2266	4.8513	M+Cl	C29H38N4	0	84.8
-	-	-	HMDB000	Lipids and	Steroids ar	Bile acids,	71977-73-	604.2842	3.4576	M+Hac-H	C26H43NO	0	67.9
-	-	-	HMDB002	Organic ac	Carboxylic	Amino acid	5969-52-8	243.1337	3.6953	M+H-H2O	C11H20N2	96.9	0
-	-	-	HMDB002	Organic ac	Carboxylic	Amino acid	39537-24-	292.1305	2.3413	M-H	C14H19N3	74.8	0
-	-	-	HMDB030	Lipids and	Steroids ar	Estrane ste	15183-37-	363.1777	3.3648	M+Hac-H	C18H24O4	0	67.9
-	-	-	-	-	-	-		240.1228	1.8325	M+H	C12H17NO	66.6	0
-	-	-	HMDB025	Benzenoid	Naphthale	Naphthoq	481-42-5;_	187.0413	1.0328	M-H	C11H8O3	0	48.7
-	-	-	HMDB024	Organohet	Azolines	Oxazolines		197.129	4.2379	2M-H	C5H9NO	0	55.9
-	-	-	HMDB002	Organic ac	Carboxylic	Amino acids, peptides		288.2027	2.1404	M+H	C12H25N5	60.4	0
-	-	-	-	-	-	-	629-22-1	299.2592	6.0565	M-H	C18H36O3	85.2	0
-	-	-	HMDB000	Lipids and	Steroids ar	Pregnane s	641-77-0;	405.2244	5.4449	M+Hac-H	C21H30O4	0	73.8
-	-	-	-	-	-	-		362.1361	4.1836	M-H2O-H	C17H23N3	76.3	0
Metabolic	Metabolism	Global and	HMDB000	Organic ox	Organooxy	Alcohols a	79-83-4;_	218.1031	2.833	M-H	C9H17NO	87.8	0
-	-	-	HMDB030	Organohet	Imidazopy	Not Available		533.2105	2.9253	M-H	C28H28F2	0	63.1
-	-	-	HMDB030	Organic ac	Carboxylic	Amino acids, peptides		480.2205	3.1104	M+Na-2H	C18H33N7	0	54.3
-	-	-	HMDB003	Organohet	Benzopyra	1-benzopy	491-38-3	147.0439	1.605	M+H	C9H6O2	52.4	0
-	-	-	HMDB000	Lipids and	Fatty Acyls	Eicosanoid	71953-80-	389.1934	4.5309	M+Na-2H	C20H32O6	0	60.9
-	-	-	-	-	-	-		393.2231	4.3788	M+H	C21H32N2	48.5	0
-	-	-	HMDB002	Organic ac	Carboxylic	Amino acids, peptides		217.119	2.3186	M-H	C9H18N2O	0	41.4
-	-	-	-	-	-	-		220.1179	2.2357	M+H	C9H17NO	86.5	0
-	-	-	-	-	-	-		277.118	3.9813	M+H-H2O	C14H18N2	98	0
-	-	-	HMDB005	Lipids and	Fatty Acyls	Fatty acids and conju		254.1385	5.4715	M+ACN+H	C11H16O4	0	43.3
-	-	-	HMDB025	Phenylprop	Macrolides	Not Available		588.2654	5.0819	M+FA-H	C30H41NO	0	48.6

-	-	-	HMDB001	Benzenoid	Benzene a	Trifluorom	54739-18-	373.1732	5.6729	M+CH3OH	C15H21F3	0	40.9
-	-	-	-	-	-	-		657.311	2.9485	2M-H	C14H23N3	57.7	0
-	-	-	-	-	-	-		623.3013	4.4187	M+H	C28H42N6	35.7	0
-	-	-	LMFA0103	-	-	-		223.17	5.9319	M-H	C14H24O2	86.8	0
-	-	-	HMDB001	Organohet	Diazanaph	Benzodiaz	52128-35-	368.1715	4.8744	M-H	C19H23N5	0	55.5
-	-	-	-	-	-	-		287.1963	5.1323	M+CH3OH	C13H22N2	62.4	0
-	-	-	HMDB002	Organic ac	Carboxylic	Amino aci	90729-28-	249.0914	2.0431	M-H	C9H18N2O	93.7	0
-	-	-	-	-	-	-		312.1567	3.7661	M-H2O-H	C14H25N3	80.7	0
-	-	-	HMDB025	Organohet	Benzofura	Not Available		298.0698	2.1278	M+FA-H	C15H11NO	38.1	0
-	-	-	HMDB002	Organic ac	Carboxylic	Amino aci	2896-20-0	274.187	1.5891	M+H	C11H23N5	65.1	0
-	-	-	HMDB025	Organic ac	Carboxylic	Amino acids, peptides		626.3003	4.2196	M+H	C35H39N5	0	54
-	-	-	-	-	-	-		274.1871	0.9643	M+H	C11H23N5	55.6	0
-	-	-	HMDB003	Phenylpro	Coumarins	Furanocoumarins		317.1177	4.2688	M+Na-2H	C19H20O3	0	40.4
-	-	-	HMDB003	Phenylpro	Macrolides	Zearaleno	17924-92-	637.3045	6.0955	2M+H	C18H22O5	0	94.9
-	-	-	-	-	-	-		269.0457	4.8053	M-H2O-H	C15H12O6	75.8	0
-	-	-	HMDB003	Lipids and	Prenol lipi	Monoterpe	18296-44-	233.0429	4.7824	M+Na-2H	C10H12O5	0	49.7
-	-	-	HMDB030	Lipids and	Prenol lipi	Sesquiterpenoids		477.1042	4.8129	M-H	C22H22O1	0	80.2
-	-	-	HMDB003	Organic ac	Carboxylic	Tricarboxy	99-14-9	175.0241	1.7713	M-H	C6H8O6	97.8	0
-	-	-	HMDB002	Organic ac	Carboxylic	Amino acids, peptides		217.119	1.501	M-H	C9H18N2O	81.4	0
-	-	-	-	-	-	-		251.1037	2.174	M-H	C12H16N2	40.4	0
-	-	-	HMDB004	Lipids and	Prenol lipi	Triterpeno	172659-13	1030.571	5.4955	M+	C52H86O2	0	43.2
-	-	-	-	-	-	-		262.1449	5.8316	M-H	C15H21NO	68.6	0
-	-	-	-	-	-	-		282.1556	1.8797	M+H	C12H19N5	47.1	0
Degradatio	Metabolism	Biosynthes	-;HMDB00	Phenylpro	Isoflavono	Isoflavono	529-59-9	431.0986	4.8129	M-H	C21H20O1	95.6	0
-	-	-	-	-	-	-		468.389	6.148	M+NH4	C24H50O7	72.1	0
-	-	-	-	-	-	-		308.1466	5.4715	M+CH3OH	C15H17NO	65.4	0
-	-	-	-	-	-	-		483.2464	4.2303	2M-H	C11H18N2	86.1	0
-	-	-	HMDB002	Organic ac	Carboxylic	Amino aci	5879-22-1	243.1337	3.4565	M+H-H2O	C11H20N2	87.2	0
-	-	-	HMDB030	Lipids and	Steroids ar	Stigmastanes and der		533.3841	7.3247	M+CH3OH	C29H50O5	0	43.3
-	-	-	HMDB006	Organic ac	Carboxylic	Amino acids, peptides		609.2451	3.5884	2M+FA-H	C13H18N2	0	45.1
-	-	-	HMDB003	Organic ac	Keto acids	Beta-keto	3249-68-1	203.092	5.638	M+FA-H	C8H14O3	0	42.2
Metabolic	Metabolism	Global and	HMDB000	Organic ox	Organooxy	Carbohydr	-;190079-	213.0167	0.8148	M-H	C5H11O7P	46.9	0
-	-	-	HMDB003	Lipids and	Prenol lipi	Sesquiterp	97653-95-	475.2073	5.5362	M+Na-H2	C27H34O7	0	60.6
-	-	-	HMDB030	Organic ac	Carboxylic	Amino acids, peptides		834.4499	5.1725	M+H-H2O	C39H65N9	0	57.2
-	-	-	HMDB024	Benzenoid	Benzene a	Benzoic acids and der		397.1616	3.6428	M-H	C19H22N6	0	65.8
-	-	-	HMDB025	Organic ac	Carboxylic	Amino acids, peptides		279.17	3.9258	M+H	C15H22N2	86.3	0
-	-	-	HMDB025	Organic ac	Carboxylic	Amino acids, peptides		175.1189	0.7352	M+H	C6H14N4O	95.9	0
-	-	-	-	-	-	-		301.177	5.4758	M+FA-H	C13H24N2	43.3	0

Metabolic	Metabolisr	Global anc	-;LMPK12:	-	-	-	5989-16-2	271.0598	4.3629	M+H-H2C	C15H12O6	86	0
-	-	-	HMDB034	-	-	-		258.0596	0.8531	M+Na-2H	C8H15NO	0	44.9
Metabolic	Metabolisr	Global anc	HMDB000:	Phenylpro	Cinnamic a	Hydroxycir	614-60-8	165.0546	1.605	M+H	C9H8O3	79.8	0
Polycyclic	Metabolisr	Xenobiotic	-	-	-	-	4385-35-7	147.0442	4.7677	M-H	C9H8O2	85.4	0
-	-	-	HMDB030:	Phenylpro	Macrolides	Not Availa	-;_	601.3201	5.2209	M+Hac-H	C28H46O1	0	91.1
-	-	-	-	-	-	-		213.1492	5.9166	M-H	C12H22O3	41.7	0
-	-	-	-	-	-	-		504.2318	2.4253	M-H	C20H35N5	39.5	0
-	-	-	-	-	-	-		343.1973	3.3529	M+H	C15H26N4	54.2	0
-	-	-	HMDB003	Phenylpro	Flavonoids	Flavans	55051-77-	305.1204	4.7449	M-H2O-H	C20H20O4	0	62.3
-	-	-	-	-	-	-		194.1175	1.9347	M+H	C11H15NC	56.1	0
-	-	-	-	-	-	-		277.1558	4.1915	M-H	C15H22N2	92.1	0
-	-	-	HMDB025:	Organohe	Benzodiox	Not Available		387.1147	1.5681	M-H	C20H21Cl	0	54.1
-	-	-	-	-	-	-		146.0964	2.2357	M+H	C10H11N	49	0
-	-	-	HMDB025:	Benzenoid	Benzene a	Anilides		577.2369	3.1026	M-H2O-H	C33H36N6	68.7	0
Metabolic	Metabolisr	Global anc	HMDB001	Organic o	Organooxy	Carbonyl c	123-08-0	123.0442	1.605	M+H	C7H6O2	86.1	0
-	-	-	-	-	-	-		528.3755	5.4315	M+H	C26H49N5	46.1	0
-	-	-	HMDB025	Organohe	Naphthop	Not Availa	78213-66-	634.2951	4.145	M+Cl	C37H45NC	0	75
-	-	-	HMDB030:	Lipids and	Prenol lipi	Diterpenoi	32630-92-	407.1677	3.5194	M+Hac-H	C19H24O6	0	56.3
-	-	-	HMDB024:	Organic ac	Carboxylic	Amino acids, peptides		482.2148	4.7602	M+Cl	C22H33N5	0	61.6
-	-	-	HMDB000	Lipids and	Fatty Acyls	Fatty acids	498-36-2	131.0703	4.5845	M-H	C6H12O3	97.5	0
-	-	-	-	-	-	-		473.1931	5.0589	2M-H	C12H15NC	51	0
-	-	-	HMDB002	Organic ac	Carboxylic	Amino acid	16875-28-	251.1037	3.018	M-H	C12H16N2	96.2	0
-	-	-	HMDB024	Organic ac	Carboxylic	Amino acids, peptides		491.2001	2.4253	M-H	C19H32N4	93.2	0
-	-	-	HMDB002	Organic ac	Carboxylic	Amino acids, peptides		217.119	2.5177	M-H	C9H18N2C	77.8	0
-	-	-	HMDB025:	Organohe	Diazinanes	Piperazines		434.2243	1.8011	M+NH4	C23H29Cl	0	44.6
-	-	-	-	-	-	-		241.1192	4.2303	M-H	C11H18N2	98.3	0
-	-	-	HMDB003	Lipids and	Prenol lipi	Terpene glycosides		565.237	2.5484	M+Cl	C26H42O1	0	77.2
-	-	-	-	-	-	-		281.0892	2.5639	M-H	C11H14N4	88.1	0
Propanoat	Metabolisr	Carbohydr	HMDB000	Organic ac	Hydroxy a	Alpha hyd	-;3347-90-	253.0929	0.8458	2M+FA-H	C4H8O3	0	53.4
-	-	-	-	-	-	-		277.1558	4.423	M-H	C15H22N2	61	0
-	-	-	-	-	-	-		404.2137	1.7852	M+H	C16H29N5	39.5	0
-	-	-	HMDB030	Phenylpro	Cinnamic a	Hydroxycir	_;	413.1566	3.6117	M+Hac-H	C21H22O5	0	49.1
Metabolic	Metabolisr	Global anc	HMDB000:	Lipids and	Steroids ar	Steroidal g	72504-58-	603.2891	4.8974	M+Cl	C30H48O1	0	58.2
Phosphotr	Environme	Membrane	HMDB024	Organic o	Organooxy	Carbohydr	3615-39-2	198.0971	0.6484	M+NH4	C6H12O6	68.2	0
-	-	-	-	-	-	-		476.2617	5.1976	M+Hac-H	C15H31N9	41.1	0
-	-	-	-	-	-	-		343.1974	3.1776	M+H	C15H26N4	50.6	0
-	-	-	-	-	-	-		298.1409	4.2069	M-H2O-H	C13H23N3	90.9	0
Metabolic	Metabolisr	Global anc	HMDB000	Organohe	Pteridines	Pterins anc	134-35-0	440.1675	3.4885	M-H2O-H	C20H25N7	0	59.1

-	-	-	HMDB025	Lipids and	Steroids ar	Hydroxyst	53-34-9;_	423.1798	4.8436	M+FA-H	C21H27FC	0	63.5
-	-	-	HMDB025	Organic ac	Carboxylic	Amino acids, peptides		247.1121	2.5947	M-H	C10H20N2	89.4	0
-	-	-	HMDB024	Organohe	Biotin and	Not Available		328.1963	3.0099	M+	C15H28N4	0	51.6
-	-	-	-	-	-	-		122.0966	2.2357	M+H	C8H11N	80.9	0
-	-	-	-	-	-	-		185.0561	1.9141	M-H2O-H	C7H12N2C	90.1	0
-	-	-	-	-	-	-		93.0703	1.7381	M+H-H2C	C7H10O	63	0
-	-	-	HMDB002	Organic ac	Carboxylic	Amino aci	3303-45-5	189.1232	1.5021	M+H	C8H16N2C	87.5	0
Metabolic	Metabolisr	Global anc	HMDB030	Organic ac	Keto acids	Gamma-k	-;_	299.0776	2.1048	M+Hac-H	C11H12O6	0	61.4
-	-	-	HMDB002	Organic ac	Carboxylic	Amino aci	3918-92-1	263.1402	3.6195	M-H	C14H20N2	89.7	0
Glyceroph	Metabolisr	Lipid meta	HMDB003	Organic ac	Carboxylic	Amino aci	1186-34-1	261.0868	3.3131	M+CH3OH	C5H13N2C	0	49.4
-	-	-	HMDB002	Organic ac	Carboxylic	Amino acids, peptides		316.1668	4.4695	M-H	C17H23N3	82.5	0
-	-	-	HMDB025	Benzenoid	Benzene a	Benzoic ac	91-40-7	214.0861	5.4229	M+H	C13H11NC	68.7	0
Metabolic	Metabolisr	Global anc	HMDB000	Organic o	Organooxy	Carbonyl c	-;_	303.1563	4.3616	2M+FA-H	C6H11NO	0	55.5
-	-	-	-	-	-	-		374.1724	5.1435	M-H2O-H	C19H27N3	48.2	0
-	-	-	HMDB025	Organic ac	Carboxylic	Amino acids, peptides		662.3589	3.6634	M+H-H2C	C30H49N9	0	46.9
Biosynthes	Metabolisr	Global anc	HMDB000	Phenylpro	Phenylpro	Not Availa	828-01-3	165.0549	4.7677	M-H	C9H10O3	99.2	0
-	-	-	-	-	-	-		284.1702	2.7782	M+NH4	C11H22O7	45.4	0
-	-	-	-	-	-	-		483.2464	4.0213	2M-H	C11H18N2	78.2	0
-	-	-	HMDB000	Organic o	Organooxy	Alcohols a	-;103302-3	77.0392	1.7381	M+H-2H2	C6H8O2	0	56.7
-	-	-	HMDB002	Organic ac	Carboxylic	Amino acids, peptides		293.1509	3.5733	M-H	C15H22N2	58.4	0
-	-	-	HMDB002	Organic ac	Carboxylic	Amino acids, peptides		231.1348	2.0125	M-H	C10H20N2	92.2	0
-	-	-	HMDB024	Organic ac	Peptidomi	Hybrid pe	67655-94-	533.2835	5.0436	M+Hac-H	C21H38N4	0	40.3
-	-	-	-	-	-	-		505.3528	5.8504	M+H	C30H48O6	52.6	0
Metabolic	Metabolisr	Global anc	HMDB000	Organic o	Organooxy	Carbohydr	80875-93-	469.2781	2.2808	M+FA-H	C24H40O6	0	52.7
-	-	-	-	-	-	-		416.2249	1.5891	M+NH4	C16H26N6	61.3	0
-	-	-	HMDB025	Organic ac	Carboxylic	Amino acids, peptides		201.1233	4.4507	M+H-H2C	C9H18N2C	0	70.5
-	-	-	HMDB006	Lipids and	Fatty Acyls	Eicosanoids		501.269	4.2919	M+Hac-H	C23H38O8	0	61.3
-	-	-	HMDB002	Organic ac	Carboxylic	Amino acids, peptides		279.17	3.6555	M+H	C15H22N2	78.4	0
-	-	-	HMDB025	Lipids and	Steroids ar	Steroid lactones		645.3109	3.1026	M+Hac-H	C29H46O1	0	59.7
-	-	-	HMDB006	Benzenoid	Phenanthr	Not Available		288.1228	5.5035	M+H	C16H17NC	66.7	0
-	-	-	-	-	-	-		488.2256	3.9748	M-H	C22H31N7	41.7	0
-	-	-	-	-	-	-		405.2245	5.5373	M+Hac-H	C21H30O4	43.3	0
-	-	-	HMDB002	Organic ac	Carboxylic	Amino aci	3918-94-3	215.1398	2.2123	M-H	C10H20N2	78.4	0
-	-	-	HMDB002	Organic ac	Carboxylic	Amino aci	1188-24-5	288.2027	1.8247	M+H	C12H25N5	80.6	0
-	-	-	-	-	-	-		284.1505	5.6611	M+FA-H	C13H21NC	57.2	0
-	-	-	HMDB001	Benzenoid	Benzene a	Benzophe	22071-15-	219.0825	0.609	M+H-2H2	C16H14O3	0	56.8
-	-	-	-	-	-	-		125	0.9203	M-H	C2H7O4P	61.4	0
Biosynthes	Metabolisr	Global anc	HMDB030	Lipids and	Fatty Acyls	Fatty acyl glycosides		423.1973	2.5708	M+ACN+H	C16H23N5	0	56.3

-	-	-	-	-	-	-		187.1082	0.8677	M-H	C8H16N2C		43	0
Metabolic	Metabolisr	Global anc	HMDB000	Organohe	Pyridines a	Pyridineca	98-92-0	123.0554	1.2488	M+H	C6H6N2O		76.5	0
-	-	-	HMDB002	Organic ac	Carboxylic	Amino acids, peptide:		189.1233	2.1801	M+H	C8H16N2C		71.6	0
Metabolic	Metabolisr	Global anc	HMDB000	Organohe	Indoles an	Indoles	2591-98-2	158.0603	4.9512	M-H	C10H9NO		80.4	0
-	-	-	HMDB025	Organic ac	Carboxylic	Amino acids, peptide:		308.0988	0.8755	M-H	C11H19NC		0	47
-	-	-	HMDB024	Benzenoid	Benzene a	Benzoic acids and de		607.2841	4.5386	M+Hac-H	C28H40N2		0	45.6
-	-	-	HMDB003	Phenylpro	Flavonoids	Flavonoid	578-74-5	433.1125	4.3629	M+H	C21H20O1		96	0
-	-	-	HMDB000	Organohe	Indoles an	Indolyl car	7417-65-4	204.0661	4.9512	M-H	C11H11NC		97.5	0
-	-	-	HMDB002	Organic ac	Carboxylic	Amino aci	126590-89	303.177	0.8456	M+H	C11H22N6		59	0
-	-	-	HMDB024	Organic o	Organooxy	Carbohydrates and ca		407.168	3.4111	M-H2O-H	C21H30O9		0	66.1
-	-	-	-	-	-	-		374.157	2.6332	M-H	C15H25N3		56.6	0
-	-	-	HMDB004	Lipids and	Steroids ar	Steroidal g	120015-16	783.424	4.8359	M+FA-H	C39H62O1		0	46.3
-	-	-	HMDB025	Organic o	Organooxy	Carbohydrates and ca		429.2857	6.0959	M+Hac-H	C21H38O5		0	46.2
-	-	-	HMDB002	Organic ac	Carboxylic	Amino aci	5875-38-7	215.0669	1.0545	M-H2O-H	C8H14N2C		76.7	0
Metabolic	Metabolisr	Global anc	HMDB000	Benzenoid	Benzene a	Phenethyla	51-67-2	138.0912	1.7303	M+H	C8H11NO		88.6	0
-	-	-	HMDB001	Organic ac	Carboxylic	Amino aci	159989-64	588.2894	3.5425	M+Na-2H	C32H45N3		0	56.9
-	-	-	HMDB000	Organohe	Lactones	Delta valer	823-22-3	79.0549	2.2357	M+H-2H2	C6H10O2		47	0
-	-	-	HMDB003	Organohe	Pyrrolizine	Not Availa	315-22-0	308.1466	5.5522	M+H-H2C	C16H23NC		0	58
-	-	-	HMDB025	Phenylpro	Macrolides	Epothilone	189453-10	526.2401	4.3459	M+Cl	C27H41NC		0	68.1
-	-	-	HMDB024	Lipids and	Fatty Acyls	Fatty acid esters		540.5344	5.9711	M+NH4	C34H66O3		0	56.2
-	-	-	-	-	-	-		372.1592	5.0436	M-H2O-H	C16H29N3		71.1	0
-	-	-	HMDB002	Organic ac	Carboxylic	Amino aci	16875-27-	265.1195	3.1259	M-H	C13H18N2		96.1	0
-	-	-	HMDB025	Organic o	Organooxy	Carbohydrates and ca		225.0613	0.6396	M-H	C7H14O8		0	49.4
-	-	-	HMDB002	Organic ac	Carboxylic	Amino acids, peptide:		221.0929	3.0799	M-H	C11H14N2		0	40.4
-	-	-	-	-	-	-		150.076	0.6247	M+H	C5H11NO		41.9	0
-	-	-	HMDB000	Organic o	Organooxy	Carbohydr	470-15-5	201.0375	0.8985	M+Na-2H	C6H12O6		40.8	0
-	-	-	HMDB000	Benzenoid	Benzene a	Benzoic ac	495-69-2	178.0503	3.7122	M-H	C9H9NO3		89.8	0
-	-	-	-	-	-	-		376.1646	4.9282	M+Cl	C14H32NC		86.2	0
-	-	-	HMDB004	Lipids and	Prenol lipi	Terpene la	117804-06	445.208	4.4028	M+H	C21H32O1		0	42.9
-	-	-	-	-	-	-		288.2027	1.526	M+H	C12H25N5		81.2	0
-	-	-	HMDB000	Lipids and	Fatty Acyls	Fatty acids	617-31-2	117.0546	3.2028	M-H	C5H10O3		87.7	0
-	-	-	HMDB024	Organic ac	Carboxylic	Amino acids, peptide:		706.2799	3.1721	M-H2O-H	C38H47NC		0	78.2
-	-	-	-	-	-	-		279.1351	2.7562	M-H	C14H20N2		71.6	0
-	-	-	HMDB002	Organic ac	Carboxylic	Amino acids, peptide:		309.1094	2.0431	M-H	C14H18N2		91.6	0
-	-	-	HMDB002	Organic ac	Carboxylic	Amino aci	3617-45-6	293.1145	2.6332	M-H	C14H18N2		86.7	0
-	-	-	-	-	-	-		363.1629	2.2597	M+H	C17H22N4		38.9	0
-	-	-	-	-	-	-		231.1702	2.4191	M+H	C11H22N2		58.7	0
-	-	-	HMDB025	Benzenoid	Benzene a	Benzoic acids and de		421.2042	3.4087	M+H	C20H28N4		0	45.2

-	-	-	HMDB003	Lipids and	Prenol lipi	Diterpenoi	6983-79-5	399.1911	5.7941	M+Na-H2	C25H30O4	0	74.8
-	-	-	-	-	-	-		340.1878	4.7067	M-H2O-H	C16H29N3	63.4	0
-	-	-	-	-	-	-		203.139	1.9347	M+H	C9H18N2(	59.4	0
Metabolic	Metabolisr	Global and	HMDB000	Lipids and	Fatty Acyls	Fatty acids	533-48-2	273.1456	4.4075	M+Hac-H	C10H18N2	0	43.6
-	-	-	HMDB002	Organic ac	Carboxylic	Amino aci	20556-11-	246.181	0.9484	M+H	C11H23N3	37.8	0
-	-	-	HMDB000	Organic ac	Carboxylic	Amino aci	869-19-2	189.1233	2.2915	M+H	C8H16N2(	90	0
Metabolic	Metabolisr	Global and	HMDB000	Organic ac	Carboxylic	Amino aci	60-18-4	182.0811	1.605	M+H	C9H11NO	99.1	0
-	-	-	-	-	-	-		211.0608	3.2256	M-H	C10H12O5	52.3	0
-	-	-	-	-	-	-		164.107	2.443	M+NH4	C10H10O	70.5	0
-	-	-	-	-	-	-		237.0614	0.8531	M-H	C9H10N4(	42.6	0
-	-	-	-	-	-	-		388.1187	3.0564	M-H2O-H	C15H25N3	52.2	0
-	-	-	HMDB000	Organohe	Imidazopy	Purines an	2465-59-(	151.0252	2.174	M-H	C5H4N4O	71.7	0
-	-	-	HMDB002	Organic ac	Carboxylic	Amino acids, peptides		231.1348	2.3186	M-H	C10H20N2	92.9	0
-	-	-	HMDB002	Organic ac	Carboxylic	Amino aci	23926-98-	632.3204	5.3182	M+ACN+I	C34H40N4	0	84
-	-	-	HMDB003	Phenylpro	Isoflavono	O-methyla	139906-02	331.1189	4.7677	M-H	C18H20O6	0	42.9
-	-	-	-	-	-	-		393.2228	4.2434	M+H	C21H32N2	52	0
-	-	-	HMDB000	Organic ac	Carboxylic	Amino aci	144964-56	559.2737	2.4869	M+Cl	C23H40N8	0	72
-	-	-	HMDB002	Organic ac	Carboxylic	Amino aci	20556-13-	260.1966	1.5101	M+H	C12H25N3	37.8	0
-	-	-	HMDB002	Organic ac	Carboxylic	Amino acids, peptides		304.1653	3.1936	M+H	C16H21N3	92.1	0
-	-	-	-	-	-	-		231.1702	2.6819	M+H	C11H22N2	59.4	0
Metabolic	Metabolisr	Global and	HMDB000	Nucleoside	Purine nuc	Not Availa	58-61-7;_	268.1038	1.864	M+H	C10H13N5	84.9	0
-	-	-	-	-	-	-		433.2081	2.5786	M+H	C21H28N4	41.6	0
-	-	-	HMDB001	Organic ac	Carboxylic	Amino aci	10148-81-	256.0939	0.7093	M-H2O-H	C10H17N3	79.7	0
-	-	-	HMDB002	Organic ac	Carboxylic	Amino acids, peptides		228.0987	2.1048	M-H	C9H15N3(	82.5	0
-	-	-	-	-	-	-		247.1287	2.2437	M+H	C10H18N2	62.7	0
-	-	-	-	-	-	-		245.1858	3.2174	M+H	C12H24N2	62.8	0
-	-	-	HMDB025	Organohe	Imidazopy	Purines and purine de		334.1146	2.9869	M+Hac-H	C12H13N5	0	41.7
Metabolic	Metabolisr	Global and	HMDB000	Benzenoid	Benzene a	Benzoic ac	118-90-1	137.0596	1.2567	M+H	C8H8O2	70.7	0
-	-	-	HMDB003	Lipids and	Fatty Acyls	Fatty acid	13323-48-	421.1832	3.7045	M+Hac-H	C20H26O6	0	57.4
-	-	-	HMDB000	Benzenoid	Phenols	1-hydroxy	104-14-3	136.0756	1.605	M+H-H2C	C8H11NO	89.1	0
-	-	-	-	-	-	-		264.1606	5.8547	M-H	C15H23N(	75	0
-	-	-	-	-	-	-		328.1515	2.9485	M-H2O-H	C14H25N3	78.7	0
-	-	-	HMDB003	Lipids and	Fatty Acyls	Fatty acyl	177261-68	559.2722	2.0062	M+CH3OH	C24H40O1	0	63.5
-	-	-	-	-	-	-		377.1784	3.2254	M+H	C18H24N4	53.2	0
-	-	-	-	-	-	-		121.0649	1.7303	M+H	C8H8O	96.3	0
-	-	-	-	-	-	-		233.1494	1.817	M+H	C10H20N2	66	0
-	-	-	HMDB026	Not Availa	Not Availa	Not Available		812.4427	5.3986	M+Cl	C39H72N(	0	80.4
-	-	-	HMDB002	Organic ac	Carboxylic	Amino aci	5879/6/1	247.1286	1.6361	M+H	C10H18N2	88.5	0

-	-	-	HMDB025	Organic ac	Carboxylic	Amino acids, peptides		521.2108	2.4947	M+Hac-H	C19H26N8	0	54.7
-	-	-	HMDB002	Organic ac	Carboxylic	Amino aci	13716-89-	203.0668	0.966	M-H	C7H12N2C	42.7	0
-	-	-	-	-	-	-		275.1039	4.4847	M-H2O-H	C14H18N2	39.8	0
-	-	-	HMDB002	Organic ac	Carboxylic	Amino aci	22951-94-	279.17	3.4883	M+H	C15H22N2	88	0
-	-	-	HMDB024	Organic ac	Carboxylic	Amino acids, peptides		446.1965	4.0521	M-H	C16H29N7	0	46.1
-	-	-	HMDB002	Organic ac	Carboxylic	Amino aci	51782-06-	205.1182	1.4306	M+H	C8H16N2C	92.6	0
-	-	-	HMDB003	Organic ac	Carboxylic	Amino aci	125310-0C	1001.516	4.6664	M+K	C48H82O1	0	63.1
-	-	-	HMDB025	Organic ni	Organonit	Amines		505.2521	4.145	M+Hac-H	C25H30N6	0	48.1
-	-	-	-	-	-	-		520.4202	6.2368	M+NH4	C28H54O7	90.9	0
-	-	-	HMDB029	Not Availa	Not Availa	Not Available		780.5457	6.1781	M+H	C44H77NC	0	42.6
-	-	-	-	-	-	-		182.118	5.6846	M-H2O-H	C10H19NC	78	0
-	-	-	-	-	-	-		203.139	2.2677	M+H	C9H18N2C	70	0
-	-	-	HMDB003	Organohe	Isoquinoli	Benzylisoq	61478-53-	814.4556	4.9872	M+ACN+	C39H64O1	0	41.8
-	-	-	-	-	-	-		245.1858	3.1056	M+H	C12H24N2	61	0
-	-	-	HMDB003	Lipids and	Prenol lipi	Terpene la	15291-76-	485.1326	2.2656	M+FA-H	C20H24O1	0	53.1
-	-	-	HMDB003	Lipids and	Fatty Acyls	Fatty acyl glycosides		491.2151	4.7824	M+Na-2H	C20H38O1	0	56.2
-	-	-	-	-	-	-		372.1597	4.8282	M-H2O-H	C16H29N3	50.7	0
-	-	-	HMDB003	Alkaloids a	Harmala a	Not Availa	59132-30-	259.0725	3.8744	M-H	C13H12N2	0	44.9
-	-	-	HMDB003	Lignans, n	Furanoid li	Tetrahydr	96917-11-	519.206	1.967	M-H	C30H32O8	0	51.2
-	-	-	-	-	-	-		216.0986	0.9509	M-H	C8H15N3C	61.3	0
-	-	-	HMDB034	-	-	-	508-44-1	237.0403	4.0056	M+FA-H	C10H8O4	0	56.3
Metabolic	Metabolisr	Global anc	HMDB000	Organic ac	Carboxylic	Amino aci	2387-71-5	345.14	1.3593	M+CH3OH	C10H18N4	52.5	0
-	-	-	HMDB003	Organic o	Organooxy	Carbohydr	35943-37-	375.1776	3.7738	M-H2O-H	C21H30O7	0	47.8
-	-	-	HMDB002	Organic ac	Carboxylic	Amino acids, peptides		288.2027	1.1933	M+H	C12H25N5	79	0
-	-	-	HMDB002	Organic ac	Carboxylic	Amino aci	6875-80-5	229.0827	1.9141	M-H2O-H	C9H16N2C	95.7	0
-	-	-	HMDB004	Organohe	Lactones	Gamma bu	5027-63-4	213.0013	1.4712	M+Na-2H	C6H8O7	0	45.4
-	-	-	HMDB002	Organic ac	Carboxylic	Amino aci	3422-39-7	291.0987	3.2566	M-H2O-H	C14H18N2	65.6	0
-	-	-	HMDB025	Organic ac	Carboxylic	Amino acids, peptides		229.1181	2.5786	M+H	C10H16N2	0	44.9
-	-	-	HMDB001	Organic ac	Carboxylic	Amino aci	2566-39-4	241.1193	4.0135	M-H2O-H	C11H20N2	96.5	0
-	-	-	HMDB024	Organohe	Diazines	Pyrimidines and pyrin		201.0869	1.872	M+H	C8H12N2C	0	42.3
-	-	-	HMDB000	Phenylpro	Phenylpro	Not Availa	306-23-0	181.05	3.2642	M-H	C9H10O4	97.9	0
-	-	-	HMDB025	Benzenoid	Benzene a	Not Available		263.1402	3.3337	M+Hac-H	C12H16N2	0	41.4
Metabolic	Metabolisr	Global anc	HMDB000	Benzenoid	Benzene a	Not Availa	103-82-2	119.0493	1.605	M+H-H2C	C8H8O2	93.9	0
-	-	-	HMDB025	Organic ac	Carboxylic	Amino acids, peptides		273.1457	4.2919	M+FA-H	C11H20N2	0	51.6
-	-	-	HMDB004	Lipids and	Prenol lipi	Terpene gl	143775-68	379.2073	3.5202	M+Na-H2	C19H34O7	0	55.2
-	-	-	-	-	-	-		430.2088	5.1052	M-H	C21H29N5	87.3	0
-	-	-	-	-	-	-		217.119	1.0106	M-H	C9H18N2C	52.8	0
-	-	-	-	-	-	-		105.0702	2.2357	M+H-2H2	C8H12O2	95.9	0

-	-	-	-	-	-	-		371.1923	2.7861	M+CH3OH	C13H24N4	38.3	0
-	-	-	-	-	-	-		261.1444	1.9113	M+H	C11H20N2	77.4	0
-	-	-	-	-	-	-		419.1787	2.4636	M-H	C17H24N8	45.3	0
-	-	-	HMDB025	Lipids and	Steroids ar	Steroid lactones		669.3469	4.9359	M+Cl	C35H54O1	0	47.7
-	-	-	-	-	-	-		348.1236	2.4328	M-H	C13H23N3	40.9	0
-	-	-	-	-	-	-		397.2191	1.6913	M+ACN+H	C15H25N5	81.1	0
-	-	-	-	-	-	-		327.1306	2.3032	M+Hac-H	C11H16N4	36.7	0
-	-	-	HMDB002	Organic ac	Carboxylic	Amino acids, peptides		243.1713	3.6812	M-H	C12H24N2	77	0
-	-	-	HMDB002	Organic ac	Carboxylic	Amino acids, peptides		243.135	3.357	M-H	C11H20N2	0	42.2
-	-	-	-	-	-	-		181.05	3.9672	M-H	C9H10O4	89.2	0
-	-	-	-	-	-	-		362.1378	2.4112	M+CH3OH	C11H21N3	38.3	0
-	-	-	-	-	-	-		187.1082	2.6716	M-H	C8H16N2O	85.5	0
-	-	-	-	-	-	-		245.1858	2.9219	M+H	C12H24N2	81.1	0
-	-	-	-	-	-	-		345.178	2.4026	M-H	C14H26N4	65.4	0
-	-	-	HMDB002	Organic ac	Carboxylic	Amino aci	26848-14-	274.1046	0.9586	M-H	C10H17N3	57.3	0
-	-	-	HMDB002	Organic ac	Carboxylic	Amino aci	7298-84-2	201.124	2.4483	M-H	C9H18N2O	77.1	0
-	-	-	HMDB024	Not Availa	Not Availa	Not Available		582.3511	4.2534	M+Hac-H	C29H49NO	0	85.2
-	-	-	HMDB002	Organic ac	Carboxylic	Amino aci	3061-90-3	237.1233	2.5389	M+H	C12H16N2	93.8	0
-	-	-	-	-	-	-		233.1495	2.2835	M+H	C10H20N2	88.1	0
-	-	-	HMDB002	Organic ac	Carboxylic	Amino acids, peptides		258.146	1.6507	M-H	C11H21N3	77.8	0
Caprolacta	Metabolisr	Xenobiotic	LMFA0000	-	-	-		243.1713	3.9748	M-H	C12H24N2	50.2	0
-	-	-	HMDB024	Organic ac	Carboxylic	Amino acids, peptides		220.0646	3.5194	M+Hac-H	C6H11NO	0	52.8
-	-	-	HMDB002	Organic ac	Carboxylic	Amino aci	3918-92-1	265.1544	3.0416	M+H	C14H20N2	90.9	0
-	-	-	-	-	-	-		103.0546	1.7381	M+H-2H2	C8H10O2	82.8	0
-	-	-	-	-	-	-		217.1546	1.9665	M+H	C10H20N2	75.9	0
-	-	-	-	-	-	-		297.1553	2.2517	M+H-2H2	C13H24N4	45	0
-	-	-	-	-	-	-		355.1625	2.7177	M-H2O-H	C15H26N4	41	0
Nicotinate	Metabolisr	Metabolisr	HMDB000	Organic ac	Carboxylic	Amino aci	583-08-4	239.0672	2.9253	M+Hac-H	C8H8N2O	0	42.3
-	-	-	-	-	-	-		422.2134	2.2597	M+H	C21H31N3	48.4	0
-	-	-	HMDB002	Organic ac	Carboxylic	Amino acids, peptides		260.1239	1.7932	M+H	C10H17N3	0	54.7
-	-	-	HMDB006	Organohe	Pyrrolidine	N-alkylpyr	106692-36	183.1133	3.1642	M-H	C9H16N2O	0	55.5
-	-	-	-	-	-	-		224.128	2.2517	M+NH4	C12H14O3	50.4	0
-	-	-	-	-	-	-		312.1567	4.1605	M-H2O-H	C14H25N3	88.2	0
-	-	-	HMDB002	Organic ac	Carboxylic	Amino acids, peptides		261.1264	3.2102	M-H	C11H22N2	71	0
Biosynthes	Metabolisr	Global anc	HMDB024	Organohe	Imidazopy	Purines and purine de		405.2244	4.8895	2M-H	C10H13N5	65.6	0
-	-	-	-	-	-	-		195.0293	3.4885	M-H	C9H8O5	35.4	0
-	-	-	HMDB027	Not Availa	Not Availa	Not Available		989.493	4.8747	M+H	C45H82O1	0	69.4
-	-	-	HMDB025	Organohe	Oxepanes	Not Availa	38647-10-	409.1821	2.3712	M+CH3OH	C20H24O7	0	46.9

-	-	-	HMDB030	Organic ac	Carboxylic	Amino acids, peptides		294.1803	1.9269	M+H	C15H23N3	0	66.5
-	-	-	HMDB002	Organic ac	Carboxylic	Amino aci	99032-17-	219.1338	1.4942	M+H	C9H18N2C	51.1	0
-	-	-	-	-	-	-		260.1967	0.98	M+H	C12H25N3	38.9	0
-	-	-	-	-	-	-		342.1671	4.1991	M-H2O-H	C15H27N3	93.9	0
-	-	-	-	-	-	-		279.0988	3.2028	M-H	C13H16N2	84.1	0
-	-	-	HMDB003	Organic ac	Carboxylic	Amino aci	38495-84-	317.1353	3.3958	M+Hac-H	C11H18N2	0	77.7
-	-	-	-	-	-	-		224.1037	2.7562	M-H	C10H15N3	64.5	0
-	-	-	HMDB002	Organic ac	Carboxylic	Amino aci	13588-94-	203.1033	1.4489	M-H	C8H16N2C	90.1	0
-	-	-	-	-	-	-		244.1189	5.414	M+Hac-H	C9H15NO	36.6	0
-	-	-	HMDB025	Organic ac	Carboxylic	Amino acids, peptides		571.3466	5.3368	M-H	C26H48N6	0	71.8
-	-	-	-	-	-	-		330.1658	2.3234	M+H	C14H23N3	36.2	0
-	-	-	HMDB002	Organic ac	Carboxylic	Amino aci	22828-05-	279.0987	2.4869	M-H	C13H16N2	91.1	0
-	-	-	HMDB002	Organic ac	Carboxylic	Amino aci	74863-12-	338.1828	1.4386	M+H	C15H23N5	58.5	0
-	-	-	-	-	-	-		314.1149	4.6528	M-H	C13H21N3	67.5	0
-	-	-	-	-	-	-		325.1866	3.1216	M+H-2H2	C15H28N4	36.4	0
-	-	-	-	-	-	-		329.1822	2.4669	M+H	C14H24N4	44.5	0
-	-	-	HMDB024	Organic o	Organooxy	Carbohydrates and ca		199.0582	4.5691	M+Na-2H	C7H14O5	0	55.6
-	-	-	HMDB025	Organohe	Naphthop	Not Availa	11076-67-	642.3478	4.3769	M+Hac-H	C37H45NC	0	62
-	-	-	-	-	-	-		202.1437	2.1484	M+H	C10H19NC	77.8	0
-	-	-	HMDB002	Organic ac	Carboxylic	Amino aci	2370-39-C	289.1406	3.9284	2M-H	C6H11NO	0	56.5
-	-	-	-	-	-	-		346.197	2.4191	M+H	C15H27N3	69.3	0
-	-	-	-	-	-	-		442.1733	3.998	M-H2O-H	C21H27N5	71.6	0
-	-	-	-	-	-	-		359.2303	2.3951	M-H	C16H32N4	51.9	0
-	-	-	-	-	-	-		240.0488	1.4788	M-H	C11H9F2N	45.7	0
-	-	-	HMDB004	Lipids and	Fatty Acyls	Fatty acyl	177261-75	395.2024	3.1376	M+Na-H2	C19H34O8	0	41
-	-	-	-	-	-	-		208.1331	5.7055	M+H	C12H17NC	56.4	0
-	-	-	-	-	-	-		173.0924	0.7014	M-H	C7H14N2C	75.9	0
-	-	-	HMDB002	Organic ac	Carboxylic	Amino aci	129050-49	231.1348	2.8251	M-H	C10H20N2	93.9	0
-	-	-	-	-	-	-		433.1754	5.7782	M+H	C22H28N2	61.2	0
-	-	-	HMDB030	Lipids and	Fatty Acyls	Fatty acids	120-87-6;	315.254	5.9631	M-H	C18H36O4	0	51.7
-	-	-	HMDB002	Organic o	Organooxy	Carbohydrates and ca		275.0541	0.6474	M+Cl	C8H16O8	0	52.3
-	-	-	HMDB002	Organic ac	Carboxylic	Amino acids, peptides		258.1823	0.988	M-H	C12H25N3	66.2	0
-	-	-	-	-	-	-		654.5139	6.2778	M+H-H2C	C38H73NC	35.6	0
Metabolic	Metabolisr	Global anc	HMDB000	Organic o	Organooxy	Carbohydr	6556/12/3	253.0564	0.7014	M+Hac-H	C6H10O7	0	44.8
-	-	-	HMDB003	Benzenoid	Benzene a	Benzoic ac	308811-92	720.4024	4.1402	M+K	C44H59NC	0	60.3
-	-	-	HMDB002	Organic ac	Carboxylic	Amino aci	34027-65-	240.1353	3.4422	M-H2O-H	C11H21N3	42.6	0
-	-	-	LMFA0200	-	-	-		297.2435	6.0255	M-H	C18H34O3	52.2	0
-	-	-	-;HMDB02	Organic ac	Carboxylic	Amino aci	7531-52-4	115.0868	0.5219	M+H	C5H10N2C	89.3	0

-	-	-	-	-	-	-	135.0441	3.2642	M-H	C8H8O2	48.8	0
-	-	-	HMDB026	Not Availa	Not Availa	Not Available	873.5527	7.3167	M+H	C46H81O1	0	41.3
-	-	-	HMDB025	Organohe	Indoles an	Indolecarboxylic acids	354.1309	2.9253	M+Na-2H	C18H23N(	0	62.3
-	-	-	-	-	-	-	208.061	2.6408	M-H	C10H11N(	42.1	0
-	-	-	HMDB002	Organic ac	Carboxylic	Amino acids, peptide	277.1194	2.833	M-H	C14H18N2	51.2	0
-	-	-	HMDB002	Organic ac	Carboxylic	Amino aci	91086-51-	219.1339	2.1881	M+H	C9H18N2(	90.4	0
Metabolic	Metabolisr	Global anc	-;HMDB00	Organic ac	Carboxylic	Amino aci	70-26-8;3	133.0972	0.5219	M+H	C5H12N2(	91.7	0
-	-	-	HMDB028	Not Availa	Not Availa	Not Available	868.5981	6.1562	M+H	C48H86N(	0	52.8
-	-	-	HMDB025	Organic ac	Carboxylic	Amino acids, peptide	217.119	2.6716	M-H	C9H18N2(	93.9	0
-	-	-	HMDB003	Organic ac	Carboxylic	Amino aci 3081-61-6	155.0817	2.074	M-H2O-H	C7H14N2(	78.8	0
-	-	-	-	-	-	-	205.0349	2.3032	M-H	C7H10O7	96.7	0
-	-	-	-	-	-	-	326.1816	2.0934	M+H	C14H23N5	37.6	0
-	-	-	HMDB002	Organic ac	Carboxylic	Amino aci 29727-65-	201.124	2.7946	M-H	C9H18N2(	92.2	0
Metabolic	Metabolisr	Global anc	HMDB000	Organohe	Imidazopy	Purines an 73-24-5	134.0462	2.0819	M-H	C5H5N5	90.2	0
-	-	-	-	-	-	-	293.1129	2.6659	M+H	C14H16N2	76.3	0
-	-	-	-	-	-	-	298.1409	3.4885	M-H2O-H	C13H23N3	67.1	0
-	-	-	HMDB030	Lipids and	Steroids ar	Bile acids, alcohols ar	489.3577	6.139	M+Na	C28H50O5	0	89.8
-	-	-	HMDB003	Phenylpro	Coumarins	Not Available	455.2034	4.6989	M+Hac-H	C24H28O5	0	63.2
-	-	-	HMDB001	Benzenoid	Phenanthr	Not Availa 52485-79-	502.2771	4.2146	M+Cl	C29H41N(	0	41.4
-	-	-	HMDB004	Phenylpro	Isoflavono	Pyranoisof 156250-69	441.1523	3.4809	M+Hac-H	C22H22O6	0	76.4
Metabolic	Metabolisr	Global anc	HMDB025	Organic o:	Organooxy	Alcohols a 108102-51	303.1563	4.145	M+Na-2H	C16H26O4	0	48.8
-	-	-	HMDB002	Organic ac	Carboxylic	Amino aci 3061-88-9	253.1182	2.435	M+H	C12H16N2	48.4	0
-	-	-	HMDB002	Organic ac	Carboxylic	Amino aci 6665-19-6	234.1446	0.5219	M+H	C9H19N3(	66.8	0
Metabolic	Metabolisr	Global anc	-;HMDB02	Organic ac	Carboxylic	Amino aci 1492-23-5	218.0666	2.074	M-H	C8H13NO(	57.3	0
-	-	-	-	-	-	-	354.2037	5.5218	M-H2O-H	C17H31N3	89.6	0
-	-	-	HMDB002	Organic ac	Carboxylic	Amino acids, peptide	295.165	2.9939	M+H	C15H22N2	66.8	0
-	-	-	-	-	-	-	429.2509	5.6225	M-H	C23H34N4	65.1	0
-	-	-	HMDB002	Organic ac	Carboxylic	Amino aci 3303-31-9	243.1713	3.7893	M-H	C12H24N2	95.7	0
-	-	-	HMDB003	Lipids and	Fatty Acyls	Fatty acyl (347852-04	561.2881	2.0301	M+CH3OH	C24H42O1	0	51.4
-	-	-	HMDB002	Organic ac	Carboxylic	Amino aci 4423-22-7	259.0758	3.1642	M-H2O-H	C10H18N2	56.7	0
-	-	-	-	-	-	-	317.1354	4.0368	M+Na-2H	C16H24O5	45.1	0
-	-	-	-	-	-	-	341.1089	1.5754	M-H	C12H22O1	68.6	0
-	-	-	-	-	-	-	229.1555	3.0408	M-H	C11H22N2	80.6	0
-	-	-	HMDB000	Organic ac	Carboxylic	Amino aci 1723-00-8	174.0765	1.9141	M+FA-H	C6H11NO:	48.9	0
-	-	-	-	-	-	-	328.1516	3.2566	M-H2O-H	C14H25N3	89.3	0
-	-	-	HMDB025	Organohe	Benzothia	Not Available	474.1845	1.9518	M+Hac-H	C22H26FN	0	77.6
-	-	-	HMDB001	Benzenoid	Benzene a	Phenylpro 829-74-3	242.1033	5.329	M+Hac-H	C9H13NO:	36.2	0
-	-	-	-	-	-	-	564.4463	6.2252	M+NH4	C30H58O8	88	0

-	-	-	HMDB002	Organic ac	Carboxylic	Amino acids, peptide:		243.1713	3.4962	M-H	C12H24N2	86.7	0
-	-	-	-	-	-	-		357.1417	2.296	M-H2O-H	C14H24N4	85.5	0
-	-	-	LMPK1100	-	-	-		552.2927	5.7387	M+Hac-H	C30H39N(	77.2	0
-	-	-	HMDB028	Not Availa	Not Availa	Not Available		894.4678	4.3948	M+Na	C44H74N(	0	42.9
-	-	-	-	-	-	-		373.173	2.1586	M-H	C15H26N4	90.9	0
-	-	-	HMDB025	Organic ac	Carboxylic	Amino acids, peptide:		145.0609	0.6703	M-H	C5H10N2(	74	0
-	-	-	HMDB024	Organohe	Benzofura	Not Available		441.236	4.5921	M+FA-H	C24H32N2	0	69.6
-	-	-	-	-	-	-		259.1299	2.0819	M-H	C11H20N2	93	0
-	-	-	-	-	-	-		470.2355	1.7694	M+NH4	C23H32O9	41.6	0
-	-	-	-	-	-	-		358.1258	2.1125	M-H2O-H	C14H23N3	73.2	0
-	-	-	HMDB003	Organic ac	Carboxylic	Amino acids, peptide:		704.3443	3.5362	M+H-2H2	C36H49N7	0	91.4
-	-	-	-	-	-	-		187.1082	2.8251	M-H	C8H16N2(	87.7	0
-	-	-	-	-	-	-		394.1969	4.4507	M+H	C19H27N3	78.2	0
-	-	-	HMDB025	Organic ac	Carboxylic	Amino acids, peptide:		682.3787	4.477	M+Cl	C31H53N9	0	43.5
-	-	-	HMDB006	Organic ac	Carboxylic	Amino aci	210769-82	202.108	4.3149	M-H	C9H17NO	0	49.4
-	-	-	HMDB025	Lipids and	Steroids ar	Estrane steroids		699.3684	3.3107	2M+FA-H	C20H25N(	0	59.1
alpha-Linc	Metabolisr	Lipid meta	HMDB000	Lipids and	Fatty Acyls	Fatty acids	6402-36-4	227.1286	5.8857	M-H	C12H20O4	77.6	0
-	-	-	-	-	-	-		354.2037	5.5992	M-H2O-H	C17H31N3	91.8	0
-	-	-	-	-	-	-		332.9629	0.5006	M+FA-H	C10H9IO2	87.8	0
-	-	-	-	-	-	-		229.1556	3.2642	M-H	C11H22N2	76.6	0
-	-	-	-	-	-	-		318.2021	3.3848	M+H	C14H27N3	66.3	0
-	-	-	HMDB003	Lipids and	Prenol lipi	Terpene glycosides		595.2954	3.6953	M+CH3OH	C26H42O1	0	48.4
-	-	-	HMDB002	Organic ac	Carboxylic	Amino aci	56217-50-	189.0875	0.6936	M-H	C7H14N2(	87.9	0
-	-	-	HMDB003	Lipids and	Fatty Acyls	Fatty acids	17705-68-	331.2488	5.8547	M-H	C18H36O5	0	91.4
-	-	-	-	-	-	-		539.2672	3.0656	M+H	C31H38O8	46.5	0
-	-	-	-	-	-	-		488.2003	2.3874	M-H	C19H31N5	69.3	0
-	-	-	HMDB025	Organic o	Organooxy	Ethers		512.4153	6.1355	M+NH4, M	C26H54O8	0	44.1
-	-	-	HMDB002	Organic ac	Carboxylic	Amino acids, peptide:		310.1394	1.518	M+H	C14H19N3	81.2	0
-	-	-	HMDB003	Organic ac	Carboxylic	Amino aci	102148-92	284.1253	2.9485	M-H2O-H	C12H21N3	0	58.2
-	-	-	-	-	-	-		652.4986	6.2047	M+NH4	C34H66O1	93	0
-	-	-	-	-	-	-		202.108	4.8895	M-H	C9H17NO	45.6	0
-	-	-	HMDB003	Benzenoid	Benzene a	Not Availa	2344-70-9	133.1011	6.0821	M+H-H2O	C10H14O	53.8	0
-	-	-	-	-	-	-		305.0007	0.5612	M+H	C11H13IO	44.7	0
-	-	-	-	-	-	-		455.2151	3.1642	2M-H	C10H16N2	76.7	0
-	-	-	HMDB002	Organic ac	Carboxylic	Amino acids, peptide:		204.1342	0.5219	M+H	C8H17N3(	66.5	0
-	-	-	-	-	-	-		236.128	2.5389	M+H	C13H17N(	69.8	0
-	-	-	HMDB002	Organic ac	Carboxylic	Amino aci	2047-13-4	322.1868	2.054	M+H	C15H23N5	79.1	0
-	-	-	HMDB024	Lipids and	Steroids ar	Bile acids, alcohols ar		540.3045	5.4064	M+FA-H	C27H45N(	0	64.6

-	-	-	-	-	-	-		372.2222	3.2254	M+H	C16H29N5	47.2	0
-	-	-	HMDB002	Organic ac	Carboxylic	Amino acids, peptide:		295.165	2.5708	M+H	C15H22N2	82.8	0
-	-	-	-	-	-	-		353.2699	6.2254	M-H	C21H38O4	95.8	0
-	-	-	HMDB002	Organic ac	Carboxylic	Amino acids, peptide:		231.1702	2.2757	M+H	C11H22N2	89.9	0
-	-	-	-	-	-	-		265.1545	4.9791	M+H	C14H20N2	63.8	0
-	-	-	HMDB001	Organic ac	Carboxylic	Amino aci	10148-81-	256.0938	2.0896	M-H2O-H	C10H17N3	43	0
-	-	-	HMDB024	Organic ac	Carboxylic	Amino acids, peptide:		515.2658	2.4328	2M-H	C15H18N2	0	44.4
-	-	-	-	-	-	-		556.4411	6.1269	M+NH4	C28H58O9	76.5	0
-	-	-	HMDB004	Lipids and	Prenol lipi	Terpene g	24404-56-	717.3815	4.2276	M+CH3OH	C36H54O1	0	55.9
-	-	-	HMDB002	Organic ac	Carboxylic	Amino aci	7369-79-1	260.1966	1.4069	M+H	C12H25N3	50.8	0
-	-	-	-	-	-	-		307.0347	1.0403	M-H	C9H13N2O	60.9	0
-	-	-	HMDB034	-	-	-		415.2562	4.5309	M+Hac-H	C17H32N4	0	43.6
Metabolic	Metabolisr	Global anc	HMDB000	Phenylpro	Flavonoids	Flavones	520-36-5;	269.0457	7.2425	M-H	C15H10O5	40.3	0
-	-	-	-	-	-	-		295.2279	5.9784	M-H	C18H32O3	84.7	0
-	-	-	HMDB006	Lipids and	Steroids ar	Androstane steroids		431.1409	1.7414	M+Cl	C21H26F2	0	42.3
-	-	-	-	-	-	-		243.0663	5.7001	M-H	C14H12O4	87.3	0
-	-	-	HMDB003	Lipids and	Prenol lipi	Quinone a	1065-31-2	608.4725	6.2116	M+NH4	C39H58O4	36.1	0
Metabolic	Metabolisr	Global anc	HMDB001	Lipids and	Steroids ar	Steroidal g	77172-80-	611.3778	4.5386	M-H	C33H56O1	0	61.9
-	-	-	-	-	-	-		489.2302	1.4306	M+H	C30H32O6	60	0
-	-	-	-	-	-	-		310.1761	1.3277	M+NH4	C15H20N2	68.7	0
-	-	-	-	-	-	-		187.1082	2.1125	M-H	C8H16N2O	57.9	0
-	-	-	-	-	-	-		376.2243	5.2824	M-H	C20H31N3	60.5	0
-	-	-	-	-	-	-		366.2361	4.1082	M+Na	C17H33N3	58.5	0
-	-	-	-	-	-	-		370.1621	3.5117	M-H2O-H	C16H27N3	67.8	0
-	-	-	HMDB002	Organic ac	Carboxylic	Amino acids, peptide:		246.1446	1.3277	M+H	C10H19N3	67	0
-	-	-	-	-	-	-		276.1342	2.9699	M+H	C14H17N3	96.1	0
-	-	-	-	-	-	-		344.2192	4.7526	M-H	C16H31N3	97.1	0
-	-	-	-	-	-	-		248.0539	1.7262	M+FA-H	C9H8F3NO	42.8	0
-	-	-	-	-	-	-		116.0708	0.5219	M+H-H2O	C5H11NO3	89.1	0
-	-	-	-	-	-	-		269.1606	1.312	M+H	C12H20N4	90.2	0
-	-	-	HMDB001	Organic ac	Carboxylic	Amino aci	75207-09-	624.2907	1.9191	M+H	C30H45N3	0	68
Metabolic	Metabolisr	Global anc	HMDB030	Lipids and	Prenol lipi	Diterpenoi	427-77-0;	375.1776	2.5639	M+Hac-H	C19H24O4	0	59.7
-	-	-	-	-	-	-		216.1018	5.2702	M+H-H2O	C13H15NO	45.5	0
-	-	-	HMDB030	Lipids and	Fatty Acyls	Fatty acyl glycosides		423.1975	3.1296	M+ACN+H	C16H23N5	0	63.4
-	-	-	HMDB003	Organohet	Dihydrofu	Furanones	80873-59-	226.1082	5.3754	M+FA-H	C10H15NO	0	88.8
-	-	-	-	-	-	-		385.2069	2.4669	M+H	C21H25FN	49.7	0
-	-	-	HMDB025	Benzenoid	Naphthale	Naphthalene sulfonic		435.2342	3.2015	M+H	C23H34N2	35.3	0
-	-	-	-	-	-	-		113.0233	0.6396	M-H	C5H6O3	40.7	0

-	-	-	-	-	-	-		114.0549	2.5177	M-H	C5H9NO2	37.8	0
-	-	-	-	-	-	-		342.1391	0.7588	M+H	C13H19N5	73.5	0
-	-	-	-	-	-	-		216.1026	5.4604	M-H	C13H15N(	38.5	0
-	-	-	HMDB000	Lipids and	Fatty Acyls	Fatty acid	3040-38-8	204.1229	4.4187	M+H	C9H17NO	0	44.8
-	-	-	-	-	-	-		432.2101	2.0204	M+Hac-H	C15H27N5	35.9	0
-	-	-	-	-	-	-		218.1385	1.9507	M+H	C10H19N(	74.1	0
-	-	-	-	-	-	-		491.1634	1.2954	M-H	C18H28N4	56.7	0
-	-	-	-	-	-	-		215.0669	0.7093	M-H2O-H	C8H14N2(	54.3	0
-	-	-	HMDB003	Organohe	Isoindoles	Isoindoline	55968-77-	471.1735	3.7893	M+FA-H	C23H26N2	0	73.5
-	-	-	HMDB002	Organic ac	Carboxylic	Amino aci	3303-34-2	201.1239	2.0896	M-H	C9H18N2(	76.4	0
Biosynthes	Metabolisr	Global anc	-;HMDB00	Organic o:	Organooxy	Carbohydr	1404-04-2	632.3496	3.9179	M+NH4	C23H46N6	40.6	0
-	-	-	HMDB000	Organic ac	Carboxylic	Amino aci	869-19-2	189.1233	1.9427	M+H	C8H16N2(	67.1	0
-	-	-	HMDB001	Organic ac	Carboxylic	Amino aci	1116-22-9	259.0923	1.8247	M+H-H2C	C10H16N2	71.5	0
-	-	-	HMDB025	Organic ac	Peptidomi	Depsipeptides		558.3287	5.1243	M+H-2H2	C29H47N5	0	51.6
Metabolic	Metabolisr	Global anc	HMDB000	Organic ac	Carboxylic	Amino aci	56-85-9	147.0763	0.68	M+H	C5H10N2(	43	0
-	-	-	HMDB001	Organic ac	Carboxylic	Amino aci	7669-65-(	263.1388	2.6899	M+H	C14H18N2	74.6	0
Metabolic	Metabolisr	Global anc	HMDB000	Organic ac	Carboxylic	Dicarboxyl	110-17-8	115.0026	0.7093	M-H	C4H4O4	92.4	0
-	-	-	HMDB025	Organic ac	Carboxylic	Amino acids, peptide:		369.178	3.3727	M+FA-H	C15H24N4	0	51.8
-	-	-	HMDB002	Organic ac	Carboxylic	Amino aci	53411-63-	533.2105	2.6638	2M+FA-H	C10H16N2	0	62.8
-	-	-	HMDB002	Organic ac	Carboxylic	Amino acids, peptide:		240.0988	1.9594	M-H2O-H	C10H17N3	0	58.1
-	-	-	HMDB003	Lipids and	Steroids ar	Steroid lac	131749-58	555.3141	2.6899	M+CH3OH	C28H42O9	0	46.2
-	-	-	HMDB025	Organic ac	Carboxylic	Amino acids, peptide:		713.3739	5.2746	2M+FA-H	C18H26N2	0	53
-	-	-	HMDB029	Not Availa	Not Availa	Not Available		710.5399	6.2176	M+	C41H74O9	0	50.5
Metabolic	Metabolisr	Global anc	HMDB000	Lipids and	Fatty Acyls	Fatty acids	-;	355.2492	5.9396	M+Hac-H	C18H32O3	85.2	0
Metabolic	Metabolisr	Global anc	HMDB000	Organic ac	Hydroxy a	Beta hydrc	97-67-6;6	133.0132	0.7093	M-H	C4H6O5	98.8	0
-	-	-	-	-	-	-		243.1713	5.1976	M-H	C12H24N2	65.6	0
-	-	-	HMDB001	Organic ac	Carboxylic	Amino aci	6403-35-6	229.1546	2.4191	M+H	C11H20N2	53.4	0
-	-	-	-	-	-	-		216.9505	0.5061	M+H	C7H5BrO3	35.3	0
Metabolic	Metabolisr	Global anc	HMDB001	Organic ac	Carboxylic	Amino aci	5105-96-4	213.0877	1.9895	M-H2O-H	C9H16N2(	0	63.3
-	-	-	-	-	-	-		267.0512	4.3381	M+Hac-H	C10H8O5	37.1	0
-	-	-	-	-	-	-		340.1879	4.9282	M-H2O-H	C16H29N3	83.2	0
D-Amino	Metabolisr	Metabolisr	HMDB003	Organic ac	Carboxylic	Amino aci	10139-06-	242.1132	1.7773	M+H-H2C	C10H17N3	0	71.3
-	-	-	HMDB025	Organic ac	Carboxylic	Amino acids, peptide:		173.0924	1.553	M-H	C7H14N2(	48.2	0
-	-	-	-	-	-	-		349.1751	5.7702	M+H	C13H24N4	35.7	0
Metabolic	Metabolisr	Global anc	HMDB000	Organic ac	Carboxylic	Tricarboxy	320-77-4	173.0085	2.4947	M-H2O-H	C6H8O7	94.4	0
-	-	-	-	-	-	-		271.1762	2.9219	M+NH4	C12H19N3	42.5	0
-	-	-	HMDB002	Organic ac	Carboxylic	Amino acids, peptide:		295.1651	2.7459	M+H	C15H22N2	78.3	0
-	-	-	HMDB028	Not Availa	Not Availa	Not Available		960.682	6.139	M+H	C52H98N(	0	59.1

-	-	-	HMDB001	Lipids and	Fatty Acyls	Fatty acid	_;	234.1334	1.8877	M+H	C10H19N(	0	42.4
-	-	-	HMDB002	Organic ac	Carboxylic	Amino acids, peptides		279.1338	2.1722	M+H	C14H18N2	91.1	0
Metabolic	Metabolisr	Global and	HMDB000	Phenylpro	Isoflavono	Isoflav-2-e	446-72-0;	269.0457	5.7387	M-H	C15H10O5	98.1	0
-	-	-	HMDB003	Organic ac	Carboxylic	Amino aci	25020-13-	298.1046	2.3491	M-H2O-H	C12H19N3	0	55.1
-	-	-	-	-	-	-		392.1831	4.8974	M-H	C19H27N3	85.3	0
-	-	-	HMDB025	Organic ac	Carboxylic	Amino acids, peptides		278.1245	1.0545	M-H	C11H21N(	0	56.9
-	-	-	-	-	-	-	23044-80-	485.2893	6.0821	M+Na-H2	C27H44O7	38.1	0
Biosynthes	Metabolisr	Metabolisr	HMDB000	Benzenoid	Benzene a	Benzoic ac	303-38-8	153.0185	3.0408	M-H	C7H6O4	98.3	0
-	-	-	-	-	-	-		300.1187	1.9665	M+H-H2O	C12H19N3	64.9	0
-	-	-	HMDB004	Organohet	Benzopyra	1-benzopyrans		607.3204	4.5306	M+Na-H2	C34H50O9	0	41.8
-	-	-	HMDB000	Organic ac	Carboxylic	Amino aci	80154-63-	264.1439	1.9269	M+CH3OH	C10H17N(	0	58.6
-	-	-	-	-	-	-		234.9609	0.5061	M+H	C7H7IO	83.6	0
-	-	-	-	-	-	-		315.1912	5.7455	M+H	C15H26N2	62.8	0
-	-	-	-	-	-	-		231.0984	0.9953	M-H	C9H16N2(	89.9	0
-	-	-	HMDB003	Organic ox	Organooxy	Carbohydr	220718-57	303.1199	3.2642	M-H2O-H	C12H22N2	0	62.2
-	-	-	HMDB006	Lipids and	Prenol lipi	Terpene gl	_;	441.2246	5.5682	M+H-2H2	C26H36O8	0	43.9
-	-	-	HMDB001	Lipids and	Steroids ar	Hydroxyste	127-31-1;	439.2095	5.6611	M+Hac-H	C21H29FC	0	51.6
-	-	-	HMDB025	Organic ac	Carboxylic	Amino acids, peptides		381.1163	4.7824	2M-H	C7H13NO	0	48.7
-	-	-	-	-	-	-		263.1423	2.4749	M+Na	C17H20O	45.6	0
-	-	-	HMDB002	Organic ac	Carboxylic	Amino aci	129050-48	267.1463	0.9735	M-H	C12H20N4	64.9	0
-	-	-	HMDB024	Organic ac	Carboxylic	Amino acids, peptides		370.1621	3.6579	M+Hac-H	C14H21N3	0	50.8
Metabolic	Metabolisr	Global and	HMDB001	Organic ac	Carboxylic	Amino aci	73980-78-	299.1248	4.1295	2M+FA-H	C6H9NO2	0	58.7
-	-	-	HMDB001	Lipids and	Steroids ar	Hydroxyste	1950/2/2;5	427.1717	4.0056	M+Cl	C22H29FC	0	59.2
-	-	-	-	-	-	-		268.1401	1.4069	M+NH4	C11H14N4	40.7	0
-	-	-	HMDB003	Lipids and	Prenol lipi	Terpene la	142628-36	675.321	3.5348	M+FA-H	C38H46O8	0	57.5
-	-	-	HMDB004	Lipids and	Prenol lipi	Monoterp	157207-55	600.4673	6.1126	M+NH4	C37H58O5	0	42.4
-	-	-	HMDB030	Phenylpro	Cinnamic a	Hydroxycinnamic acid		654.2863	3.0873	M-H2O-H	C37H43N3	0	41.4
-	-	-	HMDB001	Organohet	Diazines	Pyrimidine	56-29-1	281.1144	4.4463	M+FA-H	C12H16N2	0	40.6
-	-	-	-	-	-	-		188.0922	3.8667	M-H	C8H15NO	50.5	0
-	-	-	HMDB025	Organohet	Quinolines	Not Available		400.1362	4.2842	M+Cl	C20H23N5	0	47.7
-	-	-	HMDB024	Organic ac	Carboxylic	Amino acids, peptides		403.184	4.9823	M+Cl	C16H28N6	0	72.4
-	-	-	HMDB025	Benzenoid	Benzene a	Phenylmethylamines		320.0777	5.1744	M+FA-H	C14H13N(	0	50.5
-	-	-	HMDB002	Organic ac	Carboxylic	Amino aci	27493-61-	189.1232	1.1933	M+H	C8H16N2(	50.4	0
-	-	-	-	-	-	-		215.0823	3.8744	M-H	C12H12N2	67.9	0
-	-	-	-	-	-	-		225.1019	5.939	M+H	C14H12N2	71.9	0
-	-	-	HMDB030	Organic ac	Carboxylic	Amino acids, peptides		279.1701	5.7135	M+H	C15H22N2	83.9	0
-	-	-	HMDB001	Organic ac	Carboxylic	Amino acids, peptides		702.332	4.0829	M+Hac-H	C30H49N3	0	75.7
-	-	-	-	-	-	-		176.0916	1.7773	M+H	C7H13NO	65.8	0

-	-	-	HMDB002	Organic ac	Carboxylic	Amino acids, peptides		229.1555	2.8869	M-H	C11H22N2	94.6	0
-	-	-	HMDB002	Organic ac	Carboxylic	Amino acids, peptides		223.1076	2.4989	M+H	C11H14N2	93.8	0
-	-	-	HMDB001	Benzenoid	Benzene a	Diphenylm	25990-43-	725.3852	4.4154	2M+FA-H	C21H26N(	0	51.1
-	-	-	HMDB003	Benzenoid	Benzene a	Benzoic ac	831-61-8	197.045	3.6658	M-H	C9H10O5	78.8	0
-	-	-	HMDB025	Organic ac	Carboxylic	Carboxylic acid deriva		70.0658	0.5219	M+H-H2C	C4H9NO	0	48.8
-	-	-	HMDB025	Organic ac	Carboxylic	Amino acids, peptides		331.1513	4.1761	M+Na-2H	C12H26N2	0	45.7
-	-	-	HMDB028	Not Availa	Not Availa	Not Available		912.624	6.1562	M+H	C50H90N(	0	44.4
-	-	-	-	-	-	-		260.1603	2.1247	M+H	C11H21N3	36	0
-	-	-	HMDB001	Organic ac	Carboxylic	Amino acids, peptides		452.2403	5.2901	M+FA-H	C21H33N3	0	50.4
-	-	-	-	-	-	-		227.1035	3.1642	M-H	C10H16N2	93.9	0
-	-	-	HMDB009	Organic ac	Carboxylic	Amino acids, peptides		261.1596	5.7055	M+H	C15H20N2	0	58.8
-	-	-	-	-	-	-		383.1226	1.5754	M+FA-H	C19H18N2	82.2	0
-	-	-	HMDB004	Organohe	Diazanaph	Benzodiazines		313.118	5.5922	M+H	C17H16N2	0	53.7
-	-	-	-	-	-	-		380.1811	3.7511	M+ACN+N	C19H24O4	52.1	0
Metabolic	Metabolisr	Global anc	HMDB030	Organic ac	Carboxylic	Amino aci	53078-86-	286.0934	4.2225	M+Hac-H	C10H13N(	0	50.4
-	-	-	HMDB006	Organohe	Naphthofu	Not Available		863.4508	5.5282	M+H	C46H62N4	0	59.8
-	-	-	-	-	-	-		133.0132	1.5981	M-H	C4H6O5	89.9	0
-	-	-	HMDB002	Organic ac	Carboxylic	Amino aci	20930-58-	233.1042	1.5153	M-H2O-H	C11H16N4	73.8	0
-	-	-	-	-	-	-		358.1986	5.3831	M-H	C16H29N3	66.1	0
-	-	-	-	-	-	-		393.1869	2.4989	M+K	C20H31FC	55.9	0
-	-	-	HMDB006	Organic ac	Carboxylic	Amino acids, peptides		517.2306	5.3986	2M+FA-H	C12H16N2	0	43.1
-	-	-	HMDB002	Organic ac	Carboxylic	Amino acids, peptides		212.1028	1.9269	M+H-2H2	C9H17N3(	0	71.5
-	-	-	-	-	-	-		378.1673	4.0829	M-H	C18H25N3	58.1	0
-	-	-	-	-	-	-		431.2651	5.4875	M+H	C23H34N4	57.9	0
-	-	-	HMDB003	Organohe	Indoles an	Pyridoinde	42438-90-	217.097	3.2094	M+H	C12H12N2	95.9	0
Metabolic	Metabolisr	Global anc	HMDB000	Organic o>	Organooxy	Carbohydr	-;78341-3	522.1946	2.6332	M+Na-2H	C18H35N3	0	54.8
-	-	-	HMDB002	Organic ac	Carboxylic	Amino acids, peptides		311.104	5.6768	M-H	C14H20N2	48.1	0
Tryptopha	Metabolisr	Amino aci	HMDB001	Organic o>	Organooxy	Carbonyl c	-;958733-	226.1185	2.1563	M+NH4	C10H12N2	0	48.9
-	-	-	-	-	-	-		387.1888	2.4328	M-H	C16H28N4	88	0
-	-	-	-	-	-	-		666.5139	6.2176	M+H-H2C	C39H73N(	35	0
-	-	-	HMDB025	Organic ac	Carboxylic	Amino acids, peptides		332.1254	4.0751	M-H	C16H19N3	0	44.4
-	-	-	HMDB004	Lipids and	Prenol lipi	Terpene la	106009-45	391.1724	3.7971	M-H2O-H	C21H30O8	0	50.2
-	-	-	-	-	-	-		684.3834	5.6458	M+Cl	C36H60N(	41.8	0
-	-	-	-	-	-	-		372.2141	4.7602	M-H	C17H31N3	90.9	0
Metabolic	Metabolisr	Global anc	HMDB000	Lipids and	Steroids ar	Bile acids,	81-24-3;	514.2881	4.8436	M-H	C26H45N(	62	0
-	-	-	-	-	-	-		342.1672	3.504	M-H2O-H	C15H27N3	84.7	0
-	-	-	-	-	-	-		269.0879	0.6396	M+Hac-H	C7H14O7	53.1	0
-	-	-	HMDB003	Alkaloids a	Harmala a	Not Available		275.1039	5.1357	M+FA-H	C13H14N2	0	63.7

-	-	-	-	-	-	-		146.1175	2.3872	M+H	C7H15NO	47.9	0
-	-	-	HMDB024	Organic ac	Carboxylic	Amino aci	37841-04-	210.088	2.4103	M-H	C9H13N3	0	48.9
-	-	-	-	-	-	-		213.1241	2.5102	M-H	C10H18N2	45.1	0
Metabolic	Metabolisr	Global anc	HMDB000	Organic ox	Organooxy	Carbohydr	-;_	259.0225	0.5623	M-H	C6H13O9	65.8	0
Biosynthes	Metabolisr	Global anc	HMDB003	Phenylpro	Isoflavono	Isoflav-2-e	17817-31-	271.0598	5.7215	M+H	C15H10O5	84.7	0
-	-	-	-	-	-	-		182.1175	5.1725	M+H	C10H15N	35.8	0
-	-	-	-	-	-	-		184.0609	0.6781	M-H	C6H10F3N	58.4	0
-	-	-	HMDB004	Lipids and	Glycerolipi	Glycosylgly	35949-86-	715.3682	4.6683	M+Cl	C33H60O1	0	54.5
-	-	-	HMDB000	Organic ac	Carboxylic	Amino aci	157-07-3	156.0769	1.3898	M-H2O-H	C6H13N3	0	45
-	-	-	HMDB005	Organic ac	Carboxylic	Amino acids, peptides		182.118	5.3831	M-H2O-H	C10H19N	0	43.2
-	-	-	-	-	-	-		301.1522	5.7135	M+H	C12H20N4	97.8	0
-	-	-	HMDB001	Organic ac	Carboxylic	Amino aci	1218-34-4	245.093	3.6195	M-H	C13H14N2	36	0
-	-	-	HMDB025	Alkaloids a	Ergoline ar	Lysergic ac	511-08-0;	610.2977	4.3948	M+H	C35H39N5	0	44.9
-	-	-	HMDB003	Lipids and	Prenol lipi	Terpene la	89202-76-	601.2115	2.1356	M+FA-H	C26H36O1	0	57.1
-	-	-	-	-	-	-	24940-57-	166.0974	1.9269	M+H-H2O	C8H13N3	76.3	0
-	-	-	HMDB025	Benzenoid	Benzene a	Diphenylmethanes		602.1958	2.5333	M-H	C30H33Cl	0	52.1
-	-	-	-	-	-	-		298.1284	2.1563	M+H	C14H19N	45.4	0
-	-	-	HMDB024	Lipids and	Fatty Acyls	Fatty acid esters		783.4272	5.0201	2M+FA-H	C19H31N	0	55
-	-	-	-	-	-	-		278.1035	5.2131	M+Hac-H	C12H13N	78.9	0
-	-	-	-	-	-	-		336.165	2.5469	M+H-H2O	C15H23N5	67.5	0
-	-	-	HMDB004	Lipids and	Fatty Acyls	Fatty acyl g	177261-74	548.3075	4.4667	M+ACN+H	C24H42O1	0	40.3
-	-	-	HMDB001	Organic ox	Organooxy	Carbohydr	597-12-6	503.162	0.9355	M-H	C18H32O1	88.1	0
-	-	-	-	-	-	-		246.1447	1.9585	M+H	C10H19N3	55.6	0
-	-	-	HMDB024	Organohet	Diazines	Pyrimidines and pyrin		551.2154	4.4847	2M-H	C14H16N2	0	46.8
Metabolic	Metabolisr	Global anc	HMDB000	Organic ac	Carboxylic	Tricarboxy	585-84-2	173.0084	2.7023	M-H	C6H6O6	85.8	0
-	-	-	-	-	-	-		166.0502	3.5194	M-H	C8H9NO3	65.6	0
-	-	-	-	-	-	-		190.1073	3.3131	M+H	C8H15NO	80.9	0
-	-	-	HMDB004	Lipids and	Fatty Acyls	Fatty acyl g	177261-72	519.2429	2.456	M-H	C24H40O1	0	42.2
-	-	-	HMDB025	Organic ac	Carboxylic	Amino acids, peptides		302.1343	1.2012	M+H	C12H19N3	45	0
-	-	-	HMDB001	Organohet	Diazines	Pyrimidines and pyrin		532.2419	5.6148	M+Na-2H	C29H33N7	0	66.3
Metabolic	Metabolisr	Global anc	HMDB003	Organic ac	Hydroxy ac	Beta hydrc	636-61-3	133.0132	0.966	M-H	C4H6O5	96.8	0
-	-	-	HMDB001	Organohet	Pyridines a	Aminopyri	94-78-0	425.1933	5.005	2M-H	C11H11N5	0	53.1
-	-	-	-	-	-	-		198.0766	0.8458	M+Hac-H	C7H9NO2	56	0
-	-	-	-	-	-	-		356.1465	3.5425	M-H2O-H	C15H25N3	77.4	0
-	-	-	-	-	-	-		278.1399	5.8006	M-H	C15H21N	41.6	0
-	-	-	HMDB002	Lipids and	Fatty Acyls	Fatty acyl glycosides		505.2627	5.8022	M+H	C24H40O1	0	53.1
-	-	-	HMDB025	Benzenoid	Benzene a	Biphenyls and derivat		442.1834	5.2131	M+Hac-H	C22H25N	0	76.1
-	-	-	-	-	-	-		320.1448	1.2962	M+H	C12H21N3	70.9	0

-	-	-	HMDB024	Organic o	Organooxy	Carbohydrates and ca		504.2317	3.0254	M+Na-2H	C18H37N5	0	81.9
-	-	-	HMDB024	Organic ac	Carboxylic	Amino acids, peptides		160.0607	2.1125	M-H	C6H11NO	54.2	0
-	-	-	HMDB025	Organohe	Pyridines a	Phenylpyridines		470.1516	0.891	M+FA-H	C21H23N5	0	43.2
-	-	-	-	-	-	-		380.1811	3.5282	M+H	C18H25N3	59.6	0
-	-	-	HMDB030	Lipids and	Prenol lipi	Diterpenoi	-;_;	427.2507	5.8083	M+FA-H	C25H34O3	0	45.5
-	-	-	-	-	-	-		407.1893	2.4669	M+H-H2C	C19H28N4	63.2	0
-	-	-	-	-	-	-		280.1177	1.746	M+	C15H15F3	42.2	0
-	-	-	-	-	-	-		644.4935	6.109	M+NH4	C38H62N2	56.8	0
-	-	-	HMDB003	Organic ac	Carboxylic	Amino aci	117857-93	142.0499	1.526	M+H-H2C	C6H9NO4	0	41.5
-	-	-	-	-	-	-		262.1072	5.2702	M+H	C14H15N(	36.9	0
-	-	-	-	-	-	-		653.2571	2.5947	2M-H	C19H21N(	36.2	0
Metabolic	Metabolisr	Global anc	HMDB000	Organohe	Indoles an	Hydroxyin	-;1892-21	234.077	3.072	M+Hac-H	C10H9NO	0	55.7
-	-	-	HMDB001	Lipids and	Steroids ar	Estrane ste	13647-35-	388.2091	4.6683	M+Hac-H	C20H27N(	0	53.1
Metabolic	Metabolisr	Global anc	HMDB001	Organohe	Imidazopy	Purines an	1637-39-4	278.1246	0.7248	M+Hac-H	C10H13N5	38.4	0
-	-	-	-	-	-	-		580.3131	4.8906	M+H	C26H41N7	62.8	0
-	-	-	HMDB024	Benzenoid	Benzene a	Diphenylm	901-44-0;_	375.1776	2.8407	M+Hac-H	C19H24O4	0	44.5
Metabolic	Metabolisr	Global anc	HMDB001	Lipids and	Fatty Acyls	Fatty acids	-;928-81-	95.0496	2.2357	M+H-2H2	C6H10O3	39	0
-	-	-	HMDB001	Organic o	Organooxy	Carbohydr	470-69-9	503.1621	0.7014	M-H	C18H32O1	97.4	0
-	-	-	-	-	-	-		224.0925	5.0201	M-H	C11H15N(	52.1	0
Metabolic	Metabolisr	Global anc	HMDB000	Organic ac	Hydroxy a	Medium-c	576-36-3	195.0504	0.8381	M-H	C6H12O7	56.2	0
-	-	-	HMDB000	Phenylpro	Phenylpro	Not Availa	23028-17-	257.0668	2.5563	M+Hac-H	C9H10O5	90.2	0
-	-	-	-	-	-	-		170.0599	3.3211	M+H-H2C	C11H9NO	70.5	0
-	-	-	-	-	-	-		405.2129	4.6744	M+H	C20H28N4	84.7	0
-	-	-	-	-	-	-		314.1359	3.195	M-H2O-H	C13H23N3	58.1	0
Metabolic	Metabolisr	Global anc	HMDB000	Lipids and	Fatty Acyls	Fatty acids	498-23-7;	111.0076	2.4947	M-H2O-H	C5H6O4	0	50.4
-	-	-	-	-	-	-		279.1368	1.864	M+H	C11H22N2	78.4	0
-	-	-	HMDB002	Organic ac	Carboxylic	Amino aci	92352-82-	239.1487	2.7702	M+H-2H2	C11H22N4	0	43.7
-	-	-	HMDB002	Organic ac	Carboxylic	Amino acids, peptides		244.1302	2.1969	M-H	C10H19N3	75.1	0
-	-	-	HMDB025	Organic 1,	Allyl-type	Organic nitro compo		303.0334	0.7923	M+Na-2H	C8H9F3N4	0	45.1
-	-	-	-	-	-	-		215.1389	2.0381	M+H-H2C	C10H20N2	60.9	0
-	-	-	-	-	-	-		242.1384	5.3508	M+CH3OH	C11H15N(	58.8	0
-	-	-	HMDB002	Organic ac	Carboxylic	Amino aci	3929-61-1	257.0779	1.9895	M-H2O-H	C10H16N2	95.7	0
-	-	-	HMDB002	Organic ac	Carboxylic	Amino aci	1963-21-9	175.1076	1.5496	M+H	C7H14N2(	78	0
-	-	-	-	-	-	-		314.136	2.7023	M-H2O-H	C13H23N3	70.2	0
-	-	-	-	-	-	-		635.4145	5.8263	M+H	C36H58O9	52.3	0
-	-	-	HMDB002	Organic ac	Carboxylic	Amino aci	3918-90-9	265.1545	2.7379	M+H	C14H20N2	90.7	0
Metabolic	Metabolisr	Global anc	HMDB000	Organic o	Organooxy	Carbohydr	526-95-4	195.0504	1.5754	M-H	C6H12O7	88.5	0
Metabolic	Metabolisr	Global anc	HMDB005	Organic ac	Hydroxy a	Short-chai	2889-31-8	147.0289	1.0994	M-H	C5H8O5	94.8	0

Metabolic	Metabolisr	Global anc	HMDB000	Organohe	Imidazopy	Purines an	58-55-9	244.0789	0.6879	M+ACN+I	C7H8N4O	50.3	0
-	-	-	-	-	-	-		435.1988	3.018	M-H	C23H32O8	40.1	0
Metabolic	Metabolisr	Global anc	HMDB001	Lipids and	Steroids ar	Steroidal g	131749-23	482.2348	2.4909	M+NH4	C24H32O9	0	62.5
-	-	-	-	-	-	-		446.2596	2.6659	M+H	C19H35N5	40.4	0
-	-	-	HMDB006	Organic ac	Carboxylic	Amino acid	183241-73	236.0926	5.0666	M-H	C12H15NC	94.2	0
-	-	-	-	-	-	-		345.2129	3.0896	M+H	C15H28N4	68.2	0
Metabolic	Metabolisr	Global anc	HMDB000	Benzenoid	Phenols	Benzenedi	51-61-6;6	154.0862	1.2567	M+H	C8H11NO	90.4	0
-	-	-	-	-	-	-		355.1501	5.5842	M+H	C16H22N2	53.8	0
-	-	-	-	-	-	-		384.1525	1.7262	M-H2O-H	C15H25N5	45.7	0
-	-	-	-	-	-	-		372.2142	4.5156	M-H	C17H31N3	67.6	0
-	-	-	-	-	-	-		262.1283	0.7509	M+H	C12H15N5	49.5	0
-	-	-	HMDB030	Organic ac	Carboxylic	Amino acids, peptides		288.109	4.4618	M-H	C10H17N4	0	42.9
-	-	-	-	-	-	-		318.0946	0.7274	M+H	C14H20Cl	58.7	0
Metabolic	Metabolisr	Global anc	HMDB000	Organic o	Organooxy	Carbohydr	90-80-2	223.0456	0.7014	M+FA-H	C6H10O6	70.2	0
-	-	-	-	-	-	-		618.3089	1.8011	M+H	C25H43N7	58.5	0
-	-	-	-	-	-	-		243.1338	2.3394	M+H-H2C	C11H20N2	90.8	0
Metabolic	Metabolisr	Global anc	HMDB000	Organic o	Organooxy	Carbohydr	526-95-4	195.0504	0.624	M-H	C6H12O7	93.7	0
-	-	-	HMDB003	Organic ac	Carboxylic	Amino acids, peptides		280.1389	0.7588	M+H	C11H21NC	0	61.8
-	-	-	HMDB003	Organic o	Organooxy	Carbohydr	79043-15-	473.1527	2.12	M-H	C17H30O1	0	42.8
-	-	-	HMDB028	Not Availa	Not Availa	Not Available		956.6503	6.139	M+H	C52H94NC	0	53.9
-	-	-	-	-	-	-		212.1028	1.518	M+H-2H2	C9H17N3C	45.2	0
-	-	-	-	-	-	-		246.1447	2.2039	M+H	C10H19N3	81	0
-	-	-	HMDB003	Organic o	Organooxy	Carbohydr	14257-69-	180.0865	0.6564	M+H	C6H13NO	72.6	0
-	-	-	HMDB025	Organic ac	Carboxylic	Amino acids, peptides		338.1836	2.4253	M+Hac-H	C13H21N5	0	44.3
-	-	-	-	-	-	-		156.0808	3.0656	M+H-H2C	C11H11NC	73.9	0
-	-	-	-	-	-	-		156.0293	0.966	M-H	C6H7NO4	88.2	0
-	-	-	HMDB025	Phenylpro	Macrolides	Zearalenol	5975-78-C	379.1725	2.8791	M+Hac-H	C18H24O5	0	70.8
-	-	-	HMDB006	Lipids and	Steroids ar	Estrane steroids		726.4039	5.1052	M+Na-2H	C35H63NC	0	96.2
-	-	-	HMDB030	Organic ac	Carboxylic	Amino acids, peptides		836.4038	3.3371	M+	C41H56N8	0	80.4
-	-	-	-	-	-	-		437.2134	2.3712	M+H	C19H28N6	38.6	0
-	-	-	-	-	-	-		243.0985	2.6332	M-H	C10H16N2	60.7	0
-	-	-	-	-	-	-		328.1515	3.3881	M-H2O-H	C14H25N3	70.5	0
-	-	-	HMDB002	Organic ac	Carboxylic	Amino acid	721-90-4	223.1076	2.1642	M+H	C11H14N2	84.7	0
-	-	-	-	-	-	-		374.1574	3.0873	M-H	C15H25N3	61.8	0
-	-	-	HMDB003	Lipids and	Steroids ar	Steroidal g	78887-74-	735.3662	3.5918	M+K	C37H60O1	0	53.9
-	-	-	-	-	-	-		360.212	3.2573	M+H	C16H29N3	44.8	0
-	-	-	-	-	-	-		252.1229	2.9299	M+ACN+I	C10H12N4	39.7	0
-	-	-	-	-	-	-		554.3466	5.8182	M+H	C30H51NC	64.6	0

-	-	-	HMDB025	Alkaloids a	Ergoline ar	Lysergic acids and de		594.3117	4.8105	M+H-H2C	C35H41N5	0	57.4
-	-	-	HMDB002	Organic ac	Carboxylic	Amino acids, peptides		280.129	2.435	M+H	C13H17N3	58.8	0
-	-	-	-	-	-	-		252.1229	2.7539	M+H	C13H17NC	42	0
-	-	-	HMDB025	Organic ac	Carboxylic	Amino acids, peptides		361.1618	3.882	M+Na-2H	C14H24N6	0	52.9
-	-	-	HMDB002	Organic ac	Carboxylic	Amino acio	224638-13	304.1041	4.2146	M+Na-2H	C11H17N5	0	47.4
-	-	-	-	-	-	-		354.1057	2.5639	M+Na-2H	C11H19N5	41.1	0
-	-	-	HMDB004	Organohet	Pyridines a	Pyrrolidiny	16543-55-	399.1776	5.7309	2M+FA-H	C9H11N3C	0	41.5
Metabolic	Metabolisr	Global anc	HMDB000	Organic ac	Carboxylic	Amino acio	6205/8/9	175.1077	0.7037	M+H	C7H14N2C	55.5	0
-	-	-	-	-	-	-		234.0769	3.6736	M+FA-H	C11H11NC	38.6	0
-	-	-	-	-	-	-		298.1409	3.3805	M-H2O-H	C13H23N3	69.8	0
Metabolic	Metabolisr	Global anc	HMDB000	Organohet	Imidazopy	Purines an	73-40-5	152.0566	1.9035	M+H	C5H5N5O	90.5	0
-	-	-	-	-	-	-		224.0925	4.2225	M-H	C11H15NC	62.8	0
Metabolic	Metabolisr	Global anc	HMDB001	Benzenoid	Benzene a	Phenylacet	103-81-1	118.0653	1.7773	M+H-H2C	C8H9NO	77.9	0
-	-	-	HMDB001	Organohet	Pyrroles	Substituted pyrroles		472.1761	4.2225	M+FA-H	C24H26FN	0	40.7
-	-	-	-	-	-	-		490.2755	3.1616	M+H	C27H39NC	41.5	0
-	-	-	-	-	-	-	1818-71-9	284.0987	1.9035	M+H	C10H13N5	75.1	0
-	-	-	HMDB030	Lipids and	Prenol lipi	Terpene la	19889-01-	699.3331	3.6117	2M-H	C19H26O6	0	48.3
Metabolic	Metabolisr	Global anc	-;HMDB00	Organic ox	Organooxy	Carbohydr	3019-74-7	269.0879	0.8602	M+Hac-H	C7H14O7	35	0
-	-	-	HMDB003	Lipids and	Prenol lipi	Terpene la	67567-13-	417.1883	5.4295	M-H	C23H30O7	0	68.8
-	-	-	-	-	-	-		331.1972	2.1247	M+H	C14H26N4	89.2	0
Metabolic	Metabolisr	Global anc	HMDB000	Nucleoside	Purine nuc	Not Availa	118-00-3	282.0845	2.1278	M-H	C10H13N5	95.7	0
-	-	-	-	-	-	-		216.0179	0.5061	M+ACN+H	C5H3ClN2	40.5	0
-	-	-	HMDB024	Organic ac	Carboxylic	Amino acio	84285-33-	210.088	2.1048	M-H	C9H13N3C	0	49.6
-	-	-	-	-	-	-		377.1918	2.078	M+Na	C20H26N4	47.8	0
-	-	-	HMDB025	Organohet	Pyrans	Pyranones	2778/4/3;_	325.0154	1.0623	M+FA-H	C9H13O6P	0	45.7
-	-	-	-	-	-	-		270.1334	1.9113	M+H	C14H15N5	64.2	0
Aminoben	Metabolisr	Xenobiotic	HMDB003	Benzenoid	Phenols	Methoxypl	498-00-0	199.0607	3.4189	M+FA-H	C8H10O3	0	59.6
-	-	-	HMDB025	Phenylpro	Macrolides	Milbemycins		686.3977	4.2196	M+	C39H58O1	0	50.3
-	-	-	HMDB004	Benzenoid	Naphthale	Not Availa	91-59-8	144.0807	3.2094	M+H	C10H9N	83.5	0
-	-	-	-	-	-	-		250.1073	2.8021	M+H-H2C	C13H17NC	64.1	0
-	-	-	-	-	-	-		172.0972	4.3692	M-H	C8H15NO3	96	0
-	-	-	HMDB001	Organic ac	Carboxylic	Amino acio	2566-39-4	259.13	2.4406	M-H	C11H20N2	56.8	0
-	-	-	-	-	-	-		166.0502	2.1048	M-H	C8H9NO3	61.9	0
-	-	-	-	-	-	-		294.1351	5.785	M-H	C15H21NC	72.4	0
-	-	-	-	-	-	-		216.0986	0.7014	M-H	C8H15N3C	65.5	0
Metabolic	Metabolisr	Global anc	HMDB000	Benzenoid	Benzene a	Phenylpyru	156-06-9	163.0393	5.7619	M-H	C9H8O3	78.3	0
-	-	-	HMDB001	Organic ac	Carboxylic	Amino acio	-;_	688.3169	2.9485	M+FA-H	C30H49N3	0	81.2
-	-	-	HMDB002	Organic ac	Carboxylic	Amino acio	3062/7/5	245.1142	2.9177	M-H	C10H18N2	73.5	0

-	-	-	HMDB002!	Organic ac	Carboxylic	Amino acid	19471-43-	559.2903	3.1056	M+CH3OH	C29H36N4	0	58.3
-	-	-	-	-	-	-		247.1075	3.0656	M+NH4	C13H11NC	58.2	0
-	-	-	HMDB001	Benzenoid	Phenol eth	Not Availa	1665-48-1	280.1191	5.7697	M+Hac-H	C12H15NC	0	40.1
-	-	-	-	-	-	-		238.1071	4.6026	M+H	C12H15NC	39.6	0
-	-	-	-	-	-	-		179.0343	4.5691	M-H	C9H8O4	70.8	0
-	-	-	HMDB003	Lipids and	Steroids ar	Steroidal g	178062-95	675.4173	4.3072	M+H-2H2	C38H62O1	0	75.6
-	-	-	-	-	-	-		244.1302	2.7023	M-H	C10H19N3	90.3	0
-	-	-	-	-	-	-		427.2186	3.3848	M+H	C23H30N4	40.7	0
-	-	-	HMDB003	Organic ox	Organooxy	Carbohydr	121063-56	271.0825	3.1336	M-H2O-H	C12H18O8	0	44
-	-	-	HMDB003	Lipids and	Prenol lipi	Terpene gl	90851-26-	679.3067	2.6638	M+Cl	C32H52O1	0	83.8
-	-	-	-	-	-	-		161.0255	5.8394	M-H2O-H	C9H8O4	35.3	0
-	-	-	-	-	-	-		355.1626	3.1336	M-H2O-H	C15H26N4	35.2	0
-	-	-	-	-	-	-		259.1439	2.0934	M+H-2H2	C15H22N2	70.8	0
-	-	-	HMDB003	Lipids and	Steroids ar	Steroid lac	255861-29	657.3274	2.5865	M+Na-H2	C34H52O1	0	47.7
-	-	-	-	-	-	-		160.1331	0.9484	M+H	C8H17NO	45	0
-	-	-	HMDB030	Organic ac	Carboxylic	Amino acids, peptides		625.3579	3.4962	M+Hac-H	C27H46N6	0	85.1
-	-	-	HMDB006	Organic ac	Carboxylic	Amino acids, peptides		252.0877	3.5961	M-H	C12H15NC	0	47.6
-	-	-	HMDB002	Organic ac	Carboxylic	Amino acids, peptides		244.1301	2.0356	M-H	C10H19N3	76.5	0
-	-	-	-	-	-	-		111.0919	1.9113	M+H-H2O	C6H12N2C	39.7	0
-	-	-	-	-	-	-		390.1865	1.8091	M+H	C17H23N7	45.8	0
Cyanoamir	Metabolisr	Metabolisr	HMDB006	Organic ac	Carboxylic	Amino acid	-;_	200.1029	1.864	M+H	C8H13N3C	0	41.7
-	-	-	HMDB000	Organic ac	Carboxylic	Amino acid	4386/3/2	349.1617	1.4634	2M-H	C7H13NO	0	49.5
-	-	-	HMDB025	Organic ac	Carboxylic	Amino acids, peptides		299.1001	2.451	M+CH3OH	C8H15N2C	0	41.6
-	-	-	-	-	-	-		522.2553	3.6874	M+H	C24H35N5	50.1	0
Metabolic	Metabolisr	Global anc	HMDB030	Organic ac	Keto acids	Short-chai	-;_	291.1101	5.2209	2M-H	C6H10O4	0	47.1
-	-	-	HMDB000	Lipids and	Fatty Acyls	Fatty acids	29311-53-	145.0495	7.9845	M+H	C6H8O4	51.3	0
-	-	-	-	-	-	-		364.184	4.4667	M+H	C18H25N3	65.3	0
-	-	-	HMDB003	Organohet	Benzopyra	1-benzopy	96817-10-	511.2152	3.4422	M+Cl	C26H36O8	0	54.7
-	-	-	-	-	-	-		180.1018	2.6342	M+H	C10H13NC	85.6	0
-	-	-	-	-	-	-		332.2177	4.0845	M+H	C15H29N3	79.8	0
-	-	-	HMDB034	-	-	-		373.1619	3.8202	2M-H	C8H13NO	0	51.2
Metabolic	Metabolisr	Global anc	HMDB030	Organic ac	Carboxylic	Amino acid	-;_	228.0511	2.1048	M-H	C9H11NO	0	49.3
-	-	-	HMDB000	Organic ac	Carboxylic	Amino acid	35842-45-	216.0874	5.0974	M+Hac-H	C7H11NO	60.9	0
-	-	-	-	-	-	-		288.1915	3.2413	M+H	C13H25N3	69	0
-	-	-	HMDB000	Nucleoside	Purine nuc	Not Availa	15763-06-	282.1179	0.7667	M+H	C11H15N5	0	58.8
-	-	-	-	-	-	-		332.1491	5.2862	M+CH3OH	C17H17NC	46	0
-	-	-	-	-	-	-		442.2561	5.8239	M-H2O-H	C26H39NC	41.3	0
-	-	-	-	-	-	-	525-21-3	221.0451	4.3303	M-H	C11H10O5	62.1	0

-	-	-	HMDB002	Organic ac	Carboxylic	Amino acids, peptides		247.1287	2.3792	M+H	C10H18N2	87.1	0
-	-	-	HMDB025	Lipids and	Steroids ar	Bile acids, alcohols ar		519.2673	4.1915	M+FA-H	C24H42O7	0	61.9
-	-	-	-	-	-	-		158.0923	1.3673	M+H	C6H11N3C	45.1	0
-	-	-	-	-	-	-		267.1338	2.5389	M+H	C13H18N2	59.6	0
Metabolic	Metabolisn	Global anc	HMDB001	Organic ox	Organooxy	Carbohydr	-;_	340.1249	0.6626	M-H	C12H23NC	0	87
-	-	-	-	-	-	-		197.045	2.5563	M-H	C9H10O5	55.4	0
-	-	-	HMDB003	Organohe	Cyclohepta	Not Availa	142449-62	329.0991	3.2952	M+Na-2H	C16H20O6	0	50.9
-	-	-	-	-	-	-		269.1743	5.7862	M+H	C15H24O4	36.9	0
-	-	-	-	-	-	-		133.0496	1.0031	M-H	C5H10O4	84.4	0
-	-	-	HMDB025	Organic ac	Carboxylic	Acrylic acic	924-42-5;_	247.0934	0.7014	2M+FA-H	C4H7NO2	0	41
Metabolic	Metabolisn	Global anc	-;HMDB00	Organic ox	Organooxy	Carbohydr	131-48-6;	310.1129	0.7588	M+H	C11H19NC	73.9	0
-	-	-	-	-	-	-		342.2384	3.7272	M+H	C17H31N3	97.5	0
-	-	-	HMDB004	Organohe	Dihydrofur	Furanones		129.0547	2.6342	M+H	C6H8O3	45.7	0
-	-	-	-	-	-	-		221.0919	2.1563	M+H	C11H12N2	95.9	0
-	-	-	-	-	-	-		318.1291	0.7588	M+H-H2C	C12H21N3	50.7	0
-	-	-	-	-	-	-		288.1915	2.9539	M+H	C13H25N3	87.4	0
-	-	-	HMDB000	Lipids and	Fatty Acyls	Fatty acids	617-73-2	159.1018	5.8006	M-H	C8H16O3	70.9	0
-	-	-	-	-	-	-		426.2221	1.5813	M+H	C16H27N9	73.3	0
-	-	-	-	-	-	-		311.1232	1.8403	M+H	C18H18N2	88.1	0
-	-	-	HMDB024	Organohe	Quinolines	Quinolones and deriv		406.1832	2.5563	M+Na-2H	C20H27N5	0	54.7
-	-	-	HMDB002	Organic ac	Carboxylic	Amino acic	114659-59	336.1244	5.8083	M+Hac-H	C10H19N3	0	45.2
-	-	-	-	-	-	-		260.1603	2.459	M+H	C11H21N3	45.4	0
Histidine n	Metabolisn	Amino acic	HMDB000	Nucleoside	Imidazole	Not Availa	29605-99-	515.1634	2.4869	2M-H	C10H14N2	0	67
-	-	-	HMDB006	Organohe	Azolidines	Oxazolidines		110.0236	1.809	M-H2O-H	C5H7NO3	0	46.3
-	-	-	-	-	-	-		461.1895	2.4636	M-H	C18H30N4	49.8	0
-	-	-	-	-	-	-		273.0882	4.3074	M-H	C12H13F3	79	0
-	-	-	-	-	-	-		404.1829	4.4847	M-H2O-H	C20H29N3	67.3	0
-	-	-	-	-	-	-		276.1228	5.3989	M+CH3OH	C14H13NC	79.2	0
-	-	-	HMDB005	Organic ac	Carboxylic	Amino acids, peptides		905.4766	5.3262	M+ACN+H	C44H61N7	0	59.3
-	-	-	-	-	-	-		382.2083	2.7539	M+H	C22H27N3	58.2	0
-	-	-	-	-	-	-		260.0929	5.6071	M-H	C14H15NC	79.2	0
-	-	-	-	-	-	-		302.14	5.8239	M-H	C17H21NC	56.6	0
-	-	-	-	-	-	-		359.2284	3.1536	M+H	C16H30N4	83	0
-	-	-	HMDB025	Lipids and	Steroids ar	Steroidal g	1806-98-0	490.2402	3.4167	M+ACN+H	C24H32O8	0	44.8
-	-	-	-	-	-	-		229.098	4.3074	M-H	C13H14N2	71.7	0
Metabolic	Metabolisn	Global anc	HMDB000	Organic ni	Organonit	Amines	124-20-9	146.1651	0.5141	M+H	C7H19N3	67.3	0
-	-	-	-	-	-	-		181.0611	2.2047	M-H	C8H10N2C	59.3	0
-	-	-	-	-	-	-		347.1922	2.078	M+H	C14H26N4	80.9	0

-	-	-	-	-	-	-		284.0778	2.9099	M+FA-H	C11H13N(	47	0
-	-	-	HMDB024:	Organohe	Pyrrolidine	N-alkylpyrrolidines		271.1665	5.0974	2M+FA-H	C6H11NO	0	41
-	-	-	HMDB025	Lipids and	Steroids ar	Steroid lactones		598.2997	5.7309	M+Na-2H	C32H43N5	0	45.7
-	-	-	HMDB025:	Organic ac	Carboxylic	Amino acids, peptides		329.1928	0.8377	M+H	C13H24N6	78.3	0
-	-	-	HMDB000	Organohe	Diazines	Pyrimidine	5122-36-1	136.0617	1.0116	M+H-H2C	C5H7N5O	64.5	0
-	-	-	HMDB003	Phenylpro	Neoflavon	Prenylated	18483-64-	405.1668	5.7232	M-H	C25H26O5	0	61.9
-	-	-	-	-	-	-		197.045	3.3337	M+Hac-H	C7H6O3	92	0
-	-	-	HMDB006	Organic ac	Carboxylic	Amino aci	-;_	174.04	1.3824	M-H	C6H9NO5	0	54
-	-	-	-	-	-	-		302.2071	3.0179	M+H	C14H27N3	60.1	0
Cyanoami	Metabolisr	Metabolisr	HMDB003:	Organic o	Organooxy	Carbohydr	534-67-8	262.1283	1.0747	M+H	C11H19N(	56.1	0
-	-	-	-	-	-	-		332.2177	3.8781	M+H	C15H29N3	78.7	0
-	-	-	HMDB024	Organic o	Organooxy	Carbonyl compounds		118.0653	2.2357	M+H-H2C	C8H9NO	66.1	0
-	-	-	-	-	-	-		330.1658	2.8101	M+H-H2C	C14H25N3	67.8	0
-	-	-	-;HMDB02	Organic ac	Carboxylic	Amino aci	2280/1/5;_	229.0969	3.3689	M+H-H2C	C13H14N2	53.6	0
-	-	-	-	-	-	-		359.1535	0.6327	M+H	C14H22N4	64	0
-	-	-	-	-	-	-		403.1988	5.1205	M-H	C20H28N4	81.8	0
-	-	-	HMDB025	Lipids and	Prenol lipi	Diterpenoids		657.3765	5.6249	M+K	C36H58O8	0	86.4
-	-	-	-	-	-	-		246.1446	1.8482	M+H	C10H19N3	49.3	0
-	-	-	-	-	-	-		432.2238	4.6824	M+H	C21H29N5	42.5	0
-	-	-	HMDB004	Organic ac	Carboxylic	Amino aci	162966-07	337.1714	0.7509	M+H	C12H24N4	0	55
-	-	-	-	-	-	-		298.1409	4.0598	M-H2O-H	C13H23N3	88.5	0
-	-	-	-	-	-	-		280.1388	1.0904	M+H	C15H21N(	73.1	0
-	-	-	HMDB003	Lipids and	Steroids ar	Steroidal g	425644-95	889.4817	5.3909	M+H	C44H72O1	0	44.4
-	-	-	HMDB002	Organic ac	Carboxylic	Amino acids, peptides		342.2132	1.817	M+CH3OH	C12H25N5	0	40.2
-	-	-	-	-	-	-		358.2696	4.5946	M+H	C18H35N3	75.5	0
-	-	-	-	-	-	-		394.2314	5.6649	M+H	C20H31N3	38.9	0
-	-	-	HMDB024	Organic ac	Organic su	Arylsulfates		322.0603	3.1721	M+FA-H	C10H15N(	0	52.4
-	-	-	-	-	-	-		281.1493	2.1563	M+H	C14H20N2	66	0
-	-	-	-	-	-	-		361.1714	1.7618	M+H	C14H24N4	78.9	0
-	-	-	HMDB002	Organic ac	Carboxylic	Amino aci	104018-08	266.1232	0.7588	M+H-H2C	C11H17N5	0	65.3
-	-	-	HMDB003	Organic o	Organooxy	Carbohydr	60644-20-	258.0596	0.6165	M+Na-2H	C8H15NO	0	59.2
-	-	-	-	-	-	-		218.1386	2.3633	M+H	C10H19N(	86.2	0
-	-	-	HMDB025:	Benzenoid	Phenols	1-hydroxy-2-unsubst		236.0915	3.0896	M+H	C12H13N(	63.4	0
-	-	-	HMDB034	-	-	-		290.0884	3.2256	M-H2O-H	C11H19N(	0	57.2
-	-	-	HMDB002	Organic o	Organooxy	Carbohydr	3198-49-(	207.087	3.2102	M-H	C8H16O6	49.7	0
-	-	-	HMDB025	Organic ac	Carboxylic	Amino acids, peptides		260.114	4.2379	M+FA-H	C10H17N(	0	61.8
-	-	-	-	-	-	-		248.1127	0.7588	M+H	C10H17N(	74.1	0
-	-	-	-	-	-	-		188.128	1.9665	M+H	C9H17NO:	84.6	0

-	-	-	HMDB001	Nucleoside	Purine nuc	Not Availa	24356-66-	312.0951	2.3032	M+FA-H	C10H13N5	57.2	0
-	-	-	-	-	-	-		410.107	1.8386	M+FA-H	C17H19N	38.2	0
-	-	-	-	-	-	-		181.05	5.5604	M-H	C9H10O4	55	0
-	-	-	-	-	-	-		244.1302	2.9792	M-H	C10H19N3	81.5	0
-	-	-	HMDB003	Phenylpro	Cinnamic a	Hydroxycir	70185-61-	556.2626	4.7677	M+Hac-H	C27H35N3	0	51
Biosynthes	Metabolisr	Global anc	HMDB003	Lipids and	Prenol lipi	Terpene gl	57817-89-	827.3718	3.4883	M+Na	C38H60O1	85.6	0
-	-	-	-	-	-	-		415.1973	4.9146	M+H-H2C	C21H28N4	36.4	0
Metabolic	Metabolisr	Global anc	HMDB003	Phenylpro	Flavonoids	Flavans	98919-66-	308.1125	1.7381	M+NH4	C15H14O6	0	44.4
-	-	-	HMDB025	Organic o	Organoox	Carbonyl compounds		416.1678	3.1566	M+FA-H	C22H23F2	0	45.1
-	-	-	HMDB002	Organic ac	Carboxylic	Amino acids, peptides		278.1148	3.0254	M-H	C13H17N3	86	0
-	-	-	-	-	-	-		302.2072	3.9497	M+H	C14H27N3	57.7	0
-	-	-	-	-	-	-		253.0506	7.2425	M-H2O-H	C15H12O5	40.3	0
-	-	-	-	-	-	-		346.197	3.3769	M+H	C15H27N3	74.4	0
Metabolic	Metabolisr	Global anc	HMDB000	Organohe	Pyridines a	Pyridineca	59-67-6	124.0394	1.0904	M+H	C6H5NO2	79.7	0
-	-	-	HMDB001	Benzenoid	Benzene a	Methoxyb	390-28-3	256.1191	4.7297	M+FA-H	C11H17N	0	55.9
-	-	-	-	-	-	-		371.2287	2.7702	M+H-H2C	C17H32N4	72.9	0
Metabolic	Metabolisr	Global anc	HMDB000	Nucleoside	Ribonucle	Not Availa	84-21-9	348.07	1.6283	M+H	C10H14N5	80.1	0
-	-	-	HMDB024	Organic ac	Carboxylic	Amino aci	_;	338.0885	4.3459	M-H2O-H	C15H19N	0	50.6
Metabolic	Metabolisr	Global anc	HMDB006	Organic ac	Carboxylic	Amino aci	-;_	722.3015	3.6195	M-H	C27H49N	0	62
-	-	-	-	-	-	-		317.1466	0.6318	M-H	C12H22N4	49.9	0
-	-	-	HMDB002	Organic ac	Carboxylic	Amino acids, peptides		260.1041	3.5117	M-H	C13H15N3	72.5	0
-	-	-	-	-	-	-		194.0817	5.7387	M-H2O-H	C10H15N	43.3	0
-	-	-	HMDB002	Organic ac	Carboxylic	Amino acids, peptides		228.0987	2.4178	M-H	C9H15N3	82.5	0
-	-	-	-	-	-	-		293.0632	0.6165	M-H	C15H12F2	36.3	0
-	-	-	-	-	-	-		258.0771	4.4463	M-H	C14H13N	61.3	0
-	-	-	HMDB003	Organic o	Organoox	Carbohydr	220718-56	343.1148	4.1683	M+Na-2H	C12H22N2	0	67.2
-	-	-	HMDB002	Organic ac	Carboxylic	Amino acids, peptides		225.0344	0.6404	M+H	C6H12N2	42.9	0
-	-	-	HMDB025	Organic ac	Carboxylic	Amino aci	118399-22	866.4721	2.8739	M+ACN+	C41H60N8	0	83.4
Bisphenol	Metabolisr	Xenobiotic	HMDB024	Benzenoid	Benzene a	Diphenylmethanes		199.0759	5.8316	M-H	C13H12O2	39.5	0
-	-	-	HMDB003	Organohe	Azepines	Not Available		180.0659	2.9947	M-H2O-H	C9H13NO	0	63.1
-	-	-	HMDB030	Lipids and	Steroids ar	Steroidal glycosides		581.3652	4.1638	M+Na-H2	C33H54N	0	47.3
-	-	-	-	-	-	-		374.203	1.7932	M+H	C15H27N5	71.5	0
-	-	-	HMDB024	Organic ac	Carboxylic	Carboxylic acid deriva		198.0767	2.956	M+FA-H	C8H11NO	0	44.1
-	-	-	-	-	-	-		330.2383	3.4486	M+H	C16H31N3	84.9	0
-	-	-	HMDB002	Organic ac	Carboxylic	Amino aci	686-44-2	175.0718	0.6552	M-H	C6H12N2	76.6	0
-	-	-	HMDB003	Lipids and	Steroids ar	Steroid lac	210637-17	715.3864	3.7828	M+CH3O	C35H54O1	0	41.8
-	-	-	-	-	-	-		236.0562	2.9177	M-H	C11H11N	89.6	0
-	-	-	HMDB024	Lignans, n	Not Availa	Not Availa	478-61-5;_	607.284	3.3492	M-H	C37H40N2	0	42.1

-	-	-	-	-	-	-		302.2071	3.2254	M+H	C14H27N3	43.1	0
-	-	-	HMDB025	Lipids and	Prenol lipi	Monoterpenoids		433.2191	3.7893	M+Hac-H	C22H30O5	0	53.5
-	-	-	HMDB002	Organic ac	Carboxylic	Amino acids, peptides		230.1134	1.4942	M+H	C9H15N3O	0	63.1
-	-	-	-	-	-	-		358.1986	4.5618	M-H	C16H29N3	87.7	0
-	-	-	HMDB002	Organic ac	Carboxylic	Amino acids, peptides		227.1752	3.7988	M+H-H2O	C12H24N2	85.1	0
-	-	-	-	-	-	-		232.1188	2.074	M+Hac-H	C8H15NO	74.7	0
Tryptopha	Metabolism	Amino aci	HMDB000	Organohe	Indoles an	Indolines	-;3040-34	162.0552	4.0751	M-H	C9H9NO2	0	51.8
-	-	-	-	-	-	-		204.123	1.0588	M+H	C9H17NO	55	0
-	-	-	HMDB024	Lipids and	Steroids ar	Steroid lactones		787.4345	3.902	M+H-2H2	C43H66O1	0	51.7
-	-	-	HMDB025	Alkaloids a	Not Availa	Not Availa	520-63-8;	355.1877	5.0358	2M+FA-H	C8H13NO	0	73.5
-	-	-	HMDB000	Lipids and	Fatty Acyls	Fatty acids	29311-53-	145.0495	1.0904	M+H	C6H8O4	76.5	0
-	-	-	HMDB002	Organic ac	Carboxylic	Amino aci	936346-34	293.1145	3.2028	M-H	C14H18N2	0	59.2
-	-	-	HMDB003	Organic ac	Carboxylic	Amino aci	10003-63-	286.0934	4.0291	M+Na-2H	C10H19NO	0	50.3
-	-	-	HMDB003	Lipids and	Fatty Acyls	Fatty acyl	160551-60	304.1039	4.0984	M+FA-H	C11H17NO	0	60.1
-	-	-	-	-	-	-		180.0659	1.8539	M-H	C9H11NO	95.9	0
-	-	-	-	-	-	-		199.1803	3.7988	M+ACN+H	C9H19NO	51.3	0
-	-	-	HMDB002	Organic ox	Organooxy	Carbonyl c	1197-09-7	151.0392	3.8281	M-H	C8H8O3	90.3	0
-	-	-	HMDB026	Not Availa	Not Availa	Not Available		756.395	5.2052	M-H2O-H	C38H66NO	0	66.3
-	-	-	-	-	-	-		330.2036	4.5618	M-H	C15H29N3	89.2	0
-	-	-	-	-	-	-		433.2445	3.7112	M+H	C26H29FN	54.5	0
-	-	-	-	-	-	-		356.2557	5.2285	M-H	C18H35N3	69.9	0
-	-	-	-	-	-	-		344.1829	3.743	M-H	C15H27N3	87.7	0
-	-	-	HMDB002	Organic ac	Carboxylic	Amino aci	19746-33-	253.1193	2.9792	M-H	C12H18N2	0	43.1
-	-	-	HMDB004	Lipids and	Steroids ar	Bile acids,	155510-75	677.3336	2.9539	M+K	C34H54O1	0	50.8
Metabolic	Metabolism	Global and	HMDB000	Organic ac	Carboxylic	Amino aci	5875-41-2	199.0719	2.074	M-H2O-H	C8H14N2O	92	0
-	-	-	-	-	-	-		338.0885	4.4385	M+Na-2H	C19H15N3	67.2	0
-	-	-	HMDB002	Organic ox	Organooxy	Carbohydr	14332-17-	304.0998	0.6564	M+H	C12H17NO	0	53.1
-	-	-	HMDB000	Organic ac	Hydroxy ac	Medium-c	81093-37-	463.2096	5.5682	M+K	C23H36O7	0	69.5
Metabolic	Metabolism	Global and	HMDB000	Organohe	Tetrapyrro	Porphyrins	2624-63-7	643.311	4.7864	M+H-H2O	C36H44N4	0	41
-	-	-	-	-	-	-		316.2227	3.8701	M+H	C15H29N3	79.7	0
-	-	-	-	-	-	-		318.202	3.1936	M+H	C14H27N3	50.4	0
-	-	-	HMDB001	Benzenoid	Benzene a	Methoxyb	42794-76-	553.252	5.7156	2M+FA-H	C12H18N2	0	81.8
-	-	-	HMDB024	Lipids and	Steroids ar	Bile acids, alcohols ar		656.3158	5.6225	M-H2O-H	C32H53NO	0	85.3
-	-	-	-	-	-	-		304.1514	2.4483	M-H	C12H23N3	63.1	0
Metabolic	Metabolism	Global and	HMDB006	Benzenoid	Benzene a	Benzoic ac	-;_	325.1405	4.7903	M+Na-2H	C18H24O4	0	43.5
-	-	-	HMDB001	Benzenoid	Phenols	Benzenedi	7683-59-2	256.1191	4.5541	M+FA-H	C11H17NO	0	54.5
-	-	-	HMDB009	Organic ac	Carboxylic	Amino acids, peptides		274.1298	4.477	M+Hac-H	C10H17NO	0	57.8
-	-	-	-	-	-	-		391.2073	2.3394	M+H	C20H29F3	77.6	0

-	-	-	HMDB003	Lipids and	Prenol lipi	Terpene gl	58543-17-	825.3538	3.4652	M+Na-2H	C38H60O1	0	85.3
-	-	-	-	-	-	-		176.071	5.1052	M-H	C10H11NC	79.6	0
-	-	-	-	-	-	-		184.0609	2.1048	M-H	C8H11NO	75.6	0
-	-	-	HMDB030	Organic ac	Carboxylic	Amino acids, peptide		254.1034	5.6071	M-H2O-H	C15H17N2	0	44.9
Metabolic	Metabolisr	Global anc	HMDB000	Organic ox	Organooxy	Carbohydr	512-69-6	527.1581	0.9089	M+Na	C18H32O1	96.6	0
-	-	-	HMDB025	Organic ac	Carboxylic	Amino acids, peptide		131.1179	1.4942	M+H	C6H14N2C	42.3	0
Metabolic	Metabolisr	Global anc	HMDB000	Organohe	Pyridines a	Pyridineca	82-82-6	182.0452	2.0587	M-H	C8H9NO4	93.1	0
-	-	-	HMDB001	Organic ox	Organooxy	Carbohydr	77463-72-	320.0777	4.4463	M-H2O-H	C15H17NC	0	41.1
-	-	-	-	-	-	-		150.1025	2.5548	M+H	C8H11N3	46.9	0
-	-	-	HMDB006	Organohe	Benzazepi	Dibenzazepines		503.1998	3.7738	M+FA-H	C24H30N2	0	73.8
-	-	-	-	-	-	-		245.1142	3.2566	M-H	C10H18N2	73.7	0
-	-	-	-	-	-	-		147.0289	0.7998	M-H	C5H8O5	92.4	0
-	-	-	HMDB003	Phenylpro	Macrolides	Pectenotoxins and de		888.4558	4.9391	M+	C47H68O1	0	48
-	-	-	HMDB003	Lipids and	Prenol lipi	Terpene lactones		429.1514	3.8744	M+Na-2H	C21H28O8	0	58.3
-	-	-	-	-	-	-		318.2021	2.9859	M+H	C14H27N3	55.7	0
-	-	-	-	-	-	-		163.0866	2.2279	M+H	C9H10N2C	61.5	0
-	-	-	-	-	-	-		378.1673	4.2688	M-H	C18H25N3	89.7	0
-	-	-	HMDB002	Organic ac	Carboxylic	Amino acids, peptide		306.1476	2.427	M+H	C15H19N3	43	0
-	-	-	HMDB002	Organic ac	Carboxylic	Amino acids, peptide		258.1459	2.6486	M-H	C11H21N3	64.8	0
-	-	-	HMDB030	Lipids and	Prenol lipi	Terpene lactones		411.1996	3.7272	M+H-H2C	C21H32O9	0	70
Metabolic	Metabolisr	Global anc	HMDB000	Organohe	Imidazopy	Purines an	69-89-6	151.0252	1.5904	M-H	C5H4N4O	96.8	0
Metabolic	Metabolisr	Global anc	HMDB000	Organohe	Lactones	Gamma bu	979-92-0	385.1285	1.7694	M+H	C14H20N6	80.4	0
Metabolic	Metabolisr	Global anc	HMDB000	Nucleoside	Purine nuc	Purine ribc	61-19-8	346.0559	1.673	M-H	C10H14N5	95.3	0
-	-	-	HMDB024	Benzenoid	Phenol eth	Not Available		394.112	2.5947	M+FA-H	C15H18F3	0	51.5
-	-	-	HMDB001	Organic ac	Carboxylic	Amino acic	2577-40-4	313.1543	5.6649	M+H	C18H20N2	86.8	0
-	-	-	HMDB024	Organic ac	Carboxylic	Amino acids, peptide		357.1671	4.1991	M-H2O-H	C17H24N6	0	49.3
-	-	-	-	-	-	-		390.2402	5.2209	M-H	C21H33N3	36.5	0
-	-	-	HMDB025	Organic ox	Organooxy	Ethers		619.2744	4.0907	2M-H	C17H18N4	0	81.2
-	-	-	-	-	-	-		290.1706	2.1722	M+H	C12H23N3	41	0
-	-	-	HMDB024	Lipids and	Fatty Acyls	Fatty acid esters		305.1704	3.5282	M+NH4	C13H21NC	0	55.1
-	-	-	-	-	-	-		376.2228	4.4187	M+H	C20H29N3	91.1	0
-	-	-	-	-	-	-		346.1967	2.658	M+H	C15H27N3	68	0
-	-	-	HMDB000	Organic ac	Carboxylic	Amino acic	9083-38-9	329.1831	2.7486	M+FA-H	C13H24N4	0	50.3
-	-	-	-	-	-	-		238.0706	1.3277	M+H-H2C	C11H13NC	40.3	0
-	-	-	HMDB024	Phenylpro	Brevetoxin	Not Available		887.4377	4.3537	M+Cl	C48H68O1	0	59.6
Metabolic	Metabolisr	Global anc	HMDB000	Organic ni	Organonit	Amines	51-45-6	112.0872	1.3673	M+H	C5H9N3	37.9	0
-	-	-	HMDB025	Organic ox	Organooxy	Carbohydrates and ca		290.0884	3.1412	M-H2O-H	C11H19NC	0	65.2
Arachidon	Metabolisr	Lipid meta	HMDB000	Lipids and	Fatty Acyls	Eicosanoic	60203-57-	355.1876	5.2052	M+Na-2H	C20H30O4	0	62.8

-	-	-	HMDB003	Lipids and	Prenol lipi	Terpene gl	52077-55-	571.1719	4.2303	M+FA-H	C24H30O1	0	42.1
-	-	-	HMDB001	Organic ac	Carboxylic	Amino aci	138482-56	592.263	5.4835	M+Na-2H	C26H37N9	0	72.3
-	-	-	-	-	-	-		364.1881	4.9282	M-H	C18H27N3	69.6	0
Pentose ar	Metabolisr	Carbohydr	-	-	-	-	1114-34-7	209.0663	0.6936	M+Hac-H	C5H10O5	37.8	0
-	-	-	HMDB001	Organic o	Organooxy	Carbohydr	-;_	257.1129	1.872	M+H	C11H16N2	0	61.7
-	-	-	-	-	-	-		433.2184	3.0416	M+H	C24H32O7	75.2	0
-	-	-	-	-	-	-		376.2244	5.0742	M-H	C20H31N3	81.2	0
Phosphotr	Environme	Membrane	HMDB025	Organic ac	Carboxylic	Amino acids, peptides		309.1652	0.7588	M+H	C12H24N2	86.8	0
-	-	-	-	-	-	-		244.1302	2.5563	M-H	C10H19N3	76.3	0
-	-	-	HMDB003	Phenylpro	Flavonoids	Flavones	480-40-0;	255.0649	3.7351	M+H	C15H10O4	92.8	0
-	-	-	HMDB003	Organohe	Diazines	Pyrazines	108-50-9	109.0763	3.0416	M+H	C6H8N2	78.2	0
-	-	-	-	-	-	-		342.1671	3.6658	M-H2O-H	C15H27N3	83.4	0
-	-	-	-	-	-	-		178.0503	4.2688	M-H	C9H9NO3	85.9	0
-	-	-	-	-	-	-		300.193	3.5961	M-H	C14H27N3	73	0
-	-	-	-	-	-	-		345.178	2.5408	M-H	C14H26N4	40.6	0
Metabolic	Metabolisr	Global and	HMDB000	Benzenoid	Phenols	Methoxypl	5001-33-2	242.1033	3.4344	M+FA-H	C10H15NC	0	48.1
-	-	-	-	-	-	-		254.067	3.1181	M-H2O-H	C12H11N5	45	0
-	-	-	HMDB001	Organic ac	Carboxylic	Amino acids, peptides		626.316	3.9672	M-H2O-H	C30H51N3	0	75.4
-	-	-	HMDB024	Organic ac	Carboxylic	Amino acids, peptides		937.5347	3.8224	M+ACN+H	C47H65N1	0	74.8
-	-	-	-	-	-	-		344.254	3.7988	M+H	C17H33N3	91.1	0
-	-	-	HMDB025	Organohe	Imidazopy	Purines and purine de		446.2532	5.9147	M+NH4	C21H28N6	0	44.3
-	-	-	-	-	-	-		369.2033	5.638	M-H2O-H	C23H32O5	68.2	0
-	-	-	-	-	-	-		344.0402	1.9895	M-H	C10H12N5	49.7	0
-	-	-	HMDB024	Lipids and	Fatty Acyls	Fatty acid esters		683.3264	5.1666	2M+FA-H	C14H25NC	0	71.9
-	-	-	-	-	-	-		303.1185	1.4069	M+Na	C15H20O5	50	0
-	-	-	HMDB024	Organic ac	Carboxylic	Amino acids, peptides		488.2707	3.5121	M+H-H2C	C28H35N5	0	46.7
-	-	-	-	-	-	-		255.0874	4.7602	M+Hac-H	C10H12O4	36.7	0
-	-	-	-	-	-	-		447.2597	4.7224	M+H	C27H31FN	49	0
-	-	-	HMDB003	Organohe	Furopyran	Not Available		415.195	2.435	M+CH3OH	C19H26O8	0	48.6
-	-	-	HMDB003	Organohe	Diazines	Pyrazines	55138-63-	135.0916	3.2174	M+H	C8H10N2	0	40
-	-	-	HMDB025	Organohe	Imidazopy	Purines and purine de		294.1003	1.2803	M+H	C12H12FN	0	74.9
-	-	-	-	-	-	-		312.1108	1.2803	M+H	C14H17NC	82.7	0
-	-	-	-	-	-	-		184.108	1.3198	M+H	C8H13N3C	63.1	0
-	-	-	HMDB009	Organic ac	Carboxylic	Amino acids, peptides		242.1033	3.7277	M+FA-H	C10H15NC	0	48.8
-	-	-	-	-	-	-		298.1409	3.9437	M-H2O-H	C13H23N3	47.9	0
-	-	-	-	-	-	-		320.1616	4.1372	M-H	C16H23N3	53	0
-	-	-	-	-	-	-		364.0648	1.7223	M+H	C10H14N5	85.8	0
-	-	-	-	-	-	-		222.1124	2.2757	M+H	C12H15NC	44.6	0

-	-	-	-	-	-	-		387.2713	2.2757	M+H	C17H34N6	73.4	0
-	-	-	HMDB001(	Organic o>	Organooxy	Carbohydr	35804-66-	354.0833	3.2566	M+FA-H	C14H15NC	0	64
-	-	-	HMDB003:	Lipids and	Prenol lipi	Terpene gl	106074-96	865.4038	5.0436	M+K	C42H66O1	0	45.1
-	-	-	-	-	-	-		360.1412	2.0509	M-H	C14H23N3	80.5	0
-	-	-	HMDB028:	Not Availa	Not Availa	Not Available		950.6378	6.2116	M+CH3OH	C48H88NC	0	41.2
Pyrimidine	Metabolisr	Nucleotide	HMDB024(	Organic o>	Organooxy	Carbohydr	84-52-6	322.0445	1.0254	M-H	C9H14N3C	91.9	0
-	-	-	HMDB025(	Lipids and	Prenol lipi	Quinone and hydroqu		527.2	4.0907	M+Na-2H	C32H30N2	0	40.6
-	-	-	HMDB025	Phenylpro	Macrolides	Milbemycins		695.3775	4.1797	M+Na	C38H56O1	0	45.6
-	-	-	HMDB002	Organic ac	Carboxylic	Amino acids, peptide:		299.0883	2.1048	M+FA-H	C11H14N2	0	58.7
-	-	-	HMDB030:	Organic o>	Organooxy	Carbohydrates and ca		405.1518	3.8744	M-H2O-H	C21H28O9	0	42.6
Metabolic	Metabolisr	Global and	HMDB000	Lipids and	Prenol lipi	Sesquiterp	220496-27	466.2303	3.1856	M+	C21H39O9	0	56.8
-	-	-	-	-	-	-		403.1726	5.1976	M+Hac-H	C20H24O5	41.4	0
-	-	-	-	-	-	-		249.1262	2.1014	M+H	C10H20N2	74.2	0
-	-	-	-	-	-	-		373.2807	2.9219	M+H	C18H36N4	37.4	0
-	-	-	-	-	-	-		260.0928	5.7156	M+FA-H	C13H13NC	52.3	0
-	-	-	-	-	-	-		341.1466	2.5639	M-H2O-H	C14H24N4	72.5	0
-	-	-	HMDB004	Organic ac	Carboxylic	Amino aci	114847-20	310.1045	2.9253	M+FA-H	C12H15N3	0	68.3
-	-	-	HMDB002	Organic ac	Carboxylic	Amino aci	76019-15-	255.1449	1.8011	M+H	C11H18N4	57.6	0
-	-	-	HMDB002	Organic ac	Carboxylic	Amino aci	31489-08-	313.1408	3.7045	M+Hac-H	C12H18N2	0	73.9
-	-	-	-	-	-	-		184.0972	5.0358	M-H	C9H15NO:	70	0
-	-	-	HMDB025	Phenylpro	Stilbenes	Not Available		333.1475	1.605	M+H-H2C	C22H22O4	51.8	0
-	-	-	HMDB006	Benzenoid	Phenol eth	Not Available		240.124	5.6303	M+FA-H	C11H17NC	0	50.9
-	-	-	-	-	-	-		348.0701	1.0747	M+H	C18H10FN	77.3	0
-	-	-	-	-	-	-		167.0342	2.7562	M-H	C8H8O4	83.4	0
-	-	-	HMDB003	Phenylpro	Diarylhept	Cyclic diarylheptanoic		657.251	4.1718	M+Na	C32H42O1	0	60.3
-	-	-	HMDB003:	Lipids and	Steroids ar	Steroidal glycosides		699.3527	5.6329	M+H	C35H54O1	0	48.1
-	-	-	HMDB003:	Organohe	Diazines	Pyrazines	34413-35-	135.0917	0.901	M+H	C8H10N2	55.1	0
-	-	-	-	-	-	-		137.0597	1.526	M+H-H2C	C8H10O3	47.4	0
-	-	-	-	-	-	-		172.1331	2.3633	M+CH3OH	C8H13NO	76.8	0
-	-	-	HMDB003	Lipids and	Prenol lipi	Diterpenoids		389.1569	2.7177	M+Hac-H	C19H22O5	0	48.9
-	-	-	-	-	-	-		242.0821	5.7619	M-H	C14H13NC	58.6	0
-	-	-	HMDB003	Lipids and	Prenol lipi	Sesquiterp	-;64979-94	403.1724	5.0125	M+Na-2H	C20H30O7	0	60.9
-	-	-	-	-	-	-		259.0631	7.4974	M+Hac-H	C9H12O3S	75	0
-	-	-	-	-	-	-		357.2145	3.7199	M-H	C16H30N4	41	0
-	-	-	-	-	-	-		233.113	1.6283	M+H	C9H16N2C	64.4	0
-	-	-	-	-	-	-		375.1874	1.9743	M+H	C15H26N4	74.9	0
-	-	-	-	-	-	-		372.2142	4.6149	M-H	C17H31N3	57.8	0
-	-	-	HMDB001	Lipids and	Fatty Acyls	Eicosanoids		469.2058	2.956	M+Hac-H	C22H28F2	0	42

-	-	-	HMDB005	Organic ac	Carboxylic	Amino acids, peptides		657.3555	3.7272	M+H-2H2	C29H48N1	0	73.3
-	-	-	-	-	-	-		359.1574	1.9895	M-H	C14H24N4	36.6	0
-	-	-	-	-	-	-		445.1841	5.3213	M-H	C20H26N6	76.4	0
-	-	-	HMDB006	Organic o	Organooxy	Carbohydrates and ca		375.141	4.2842	M+Na-2H	C18H26O7	0	67.9
Furfural de	Metabolism	Xenobiotic	HMDB001	Organohet	Heteroaro	Not Availa	98-00-0	99.0445	7.9845	M+H	C5H6O2	52.9	0
-	-	-	-	-	-	-		176.1069	2.2757	M+H-H2O	C11H15NO	83.7	0
Pyrimidine	Metabolism	Nucleotide	HMDB000	Nucleoside	Pyrimidine	Pyrimidine	50-89-5	287.0885	2.8485	M+FA-H	C10H14N2	0	51.2
-	-	-	-	-	-	-		372.189	2.0125	M-H	C15H27N5	36.7	0
-	-	-	HMDB025	Organohet	Diazines	Pyrimidines and pyrin		415.1474	2.1125	M+Hac-H	C15H24N4	0	63.4
-	-	-	HMDB002	Organic ac	Carboxylic	Amino acids, peptides		262.1188	2.9459	M+H	C13H15N3	87	0
Metabolic	Metabolism	Global and	HMDB000	Phenylprop	Isoflavono	Isoflav-2-e	486-66-8;	253.0507	5.6611	M-H	C15H10O4	88.3	0
Linoleic ac	Metabolism	Lipid meta	LMFA0200	-	-	-		313.2386	5.9166	M-H	C18H34O4	82.5	0
-	-	-	HMDB002	Organic ac	Carboxylic	Amino acid	106326-78	330.1732	3.8383	M+CH3OH	C10H21N5	0	52.8
-	-	-	-	-	-	-		352.1859	3.4883	M+H	C17H25N3	73.2	0
-	-	-	HMDB002	Organic ac	Carboxylic	Amino acids, peptides		243.1338	3.2094	M+H-H2O	C11H20N2	95	0
-	-	-	-	-	-	-		328.2228	3.2094	M+H	C16H29N3	87.6	0
-	-	-	HMDB024	Organohet	Benzopyra	1-benzopy	521-35-7;	369.2032	5.8316	M+Hac-H	C21H26O2	0	57.1
-	-	-	-	-	-	-		300.193	3.7971	M-H	C14H27N3	45.5	0
Biosynthes	Metabolism	Global and	HMDB030	Lipids and	Prenol lipi	Diterpenoi	561-56-8;	389.1569	2.8869	M+Hac-H	C19H22O5	0	50.4
-	-	-	-	-	-	-		328.1388	2.117	M+H	C15H21NO	74.7	0
-	-	-	-	-	-	-		314.2072	2.4829	M+H	C15H27N3	37.6	0
-	-	-	HMDB024	Phenylprop	Phenylprop	Not Availa	305-96-4;	228.123	5.1725	M+CH3OH	C10H13NO	0	40.8
-	-	-	HMDB000	Phenylprop	Phenylprop	Not Availa	23028-17-	257.0667	2.7407	M+Hac-H	C9H10O5	77.7	0
-	-	-	HMDB025	Lipids and	Prenol lipi	Terpene lactones		624.3158	5.7619	M-H	C35H47NO	0	78.4
-	-	-	-	-	-	-		215.139	2.2995	M+H	C10H18N2	58.3	0
-	-	-	-	-	-	-		382.2446	3.1376	M+H	C18H31N5	79	0
-	-	-	-	-	-	-		357.1766	3.3371	M+H	C19H24N4	57.8	0
-	-	-	HMDB003	Lipids and	Fatty Acyls	Fatty acid esters		403.1725	3.5348	M+Hac-H	C20H24O5	0	67.7
-	-	-	-	-	-	-		142.1226	1.9665	M+H	C8H15NO	48.9	0
-	-	-	-	-	-	-		253.0506	4.2688	M-H	C15H10O4	72.7	0
-	-	-	-	-	-	-		473.2357	1.9585	M+H	C24H32N4	62.6	0
-	-	-	LMFA0114	-	-	-		323.2576	5.8423	M+CH3OH	C17H32O2	59	0
-	-	-	-	-	-	-		373.2806	3.1616	M+H	C18H36N4	70.4	0
Degradatio	Metabolism	Global and	HMDB003	Organic o	Organooxy	Carbohydr	620-24-6	107.0495	1.6205	M+H-H2O	C7H8O2	83.3	0
-	-	-	HMDB003	Lipids and	Steroids ar	Steroid lac	221695-66	691.3318	5.6689	M+Hac-H	C34H48O1	0	75.1
-	-	-	HMDB002	Organic ac	Carboxylic	Amino acid	36314-37-	242.0782	0.9953	M-H2O-H	C9H15N3O	71.9	0
-	-	-	-	-	-	-		258.15	5.7774	M-H2O-H	C16H23NO	39	0
-	-	-	-	-	-	-		461.1681	4.3381	M-H	C20H30O1	43.3	0

-	-	-	HMDB030	Organic ac	Carboxylic	Amino acids, peptides		341.0991	2.9099	M+FA-H	C13H16N2	0	45.7
-	-	-	HMDB000	Organic ac	Carboxylic	Amino acid	7292-81-1	194.0453	3.8744	M-H	C9H9NO4	54.2	0
-	-	-	-	-	-	-		454.1838	5.5604	M-H	C21H25N7	54.5	0
-	-	-	-	-	-	-		519.2416	2.427	M+H	C24H34N6	50.5	0
-	-	-	HMDB025	Organohe	Pyrroles	Substituted pyrroles		489.2695	5.6815	M+ACN+N	C26H35NO	0	53.4
Metabolic	Metabolism	Global and	HMDB000	Organic ac	Keto acids	Medium-c	17510-99-	177.0398	1.5305	M-H	C6H10O6	49.5	0
-	-	-	-	-	-	-		326.2086	3.7893	M-H	C16H29N3	60	0
-	-	-	HMDB030	Lipids and	Prenol lipi	Terpene glycosides		471.2211	2.3796	M+FA-H	C22H34O8	0	67.3
-	-	-	-	-	-	-		178.0715	0.6086	M+Hac-H	C4H9NO3	40	0
-	-	-	-	-	-	-		181.097	3.894	M+H	C9H12N2O	51.5	0
-	-	-	-	-	-	-		350.1209	2.1325	M+H	C16H19N3	81.1	0
-	-	-	HMDB003	Lipids and	Steroids ar	Steroid lac	125708-07	859.392	5.0277	M+H	C41H62O1	0	59.6
-	-	-	-	-	-	-		206.0566	2.1048	M-H	C9H9N3O	74.7	0
Metabolic	Metabolism	Global and	HMDB003	Organic o	Organooxy	Carbohydr	37248-47-	556.2267	3.5579	M+Hac-H	C20H35NO	0	79.3
-	-	-	HMDB024	Organic ac	Keto acids	Gamma-keto acids ar		274.0918	1.3277	M+H	C11H15NO	0	47.8
-	-	-	HMDB030	Lipids and	Fatty Acyls	Fatty acids and conju		516.2312	1.501	M+Na-2H	C25H39N2	0	84.9
-	-	-	HMDB025	Organohe	Benzodiox	Not Availa	42542-10-	238.1084	5.6689	M+FA-H	C11H15NO	0	43.1
-	-	-	-	-	-	-		360.2122	3.3848	M+H	C16H29N3	49.3	0
-	-	-	HMDB026	Not Availa	Not Availa	Not Available		570.2992	1.7381	M+NH4	C25H45O1	0	41.9
-	-	-	-	-	-	-		148.112	2.3633	M+H-H2O	C10H15NO	73.7	0
-	-	-	HMDB025	Benzenoid	Fluorenes	Not Availa	60-70-8;_	454.2925	5.9013	M+FA-H	C27H39NO	0	40.5
-	-	-	-	-	-	-		401.287	2.7459	M+H	C18H36N6	81.7	0
-	-	-	-	-	-	-		310.1282	2.117	M+H	C16H15N5	70.8	0
-	-	-	HMDB003	Lipids and	Steroids ar	Steroid lac	148076-22	777.3324	2.4032	M+CH3OH	C36H50O1	0	91
-	-	-	-	-	-	-		300.193	4.6149	M-H	C14H27N3	89.8	0
-	-	-	-	-	-	-		290.1036	4.859	M-H	C15H17NO	44.8	0
-	-	-	HMDB025	Organic ac	Carboxylic	Amino acids, peptides		214.0717	2.5025	M-H	C9H13NO	0	51.4
Metabolic	Metabolism	Global and	HMDB000	Organic ac	Carboxylic	Amino acid	-;899816-9	231.0984	1.6355	M-H	C9H16N2O	0	67.2
-	-	-	-	-	-	-		240.0513	2.8251	M+FA-H	C9H9NO4	43.6	0
-	-	-	-	-	-	-		440.1676	4.0984	M-H	C17H32NO	55.6	0
-	-	-	-	-	-	-		303.1337	2.9939	M+H	C15H23ClO	76.7	0
-	-	-	HMDB004	Lipids and	Prenol lipi	Terpene gl	145826-48	821.4402	5.0277	M+H-H2O	C43H66O1	0	47.1
-	-	-	HMDB003	Organic ac	Carboxylic	Amino acids, peptides		114.0549	0.6936	M-H2O-H	C5H11NO	0	42.4
-	-	-	HMDB025	Alkaloids a	Not Availa	Not Available		330.1351	5.5992	M-H2O-H	C18H23NO	0	52.9
-	-	-	-	-	-	-		314.0843	1.3198	M+H	C17H15NO	79.5	0
-	-	-	-	-	-	-		240.1229	2.834	M+H	C12H17NO	39.4	0
Nitrotolue	Metabolism	Xenobiotic	HMDB004	Benzenoid	Benzene a	Toluenes	95-80-7	123.0918	4.1322	M+H	C7H10N2	48.5	0
-	-	-	-	-	-	-		371.2285	2.866	M+H-H2O	C17H32N4	66.5	0

-	-	-	HMDB002	Organic ac	Carboxylic	Amino acids, peptides		217.119	2.9485	M-H	C9H18N2C	92.1	0
-	-	-	-	-	-	-		286.1774	3.5425	M-H	C13H25N3	77.5	0
Metabolic	Metabolisr	Global anc	HMDB000	Organohe	Pteridines	Pterins anc	2311-81-1	471.1741	3.2874	M-H	C20H24N8	0	76.1
-	-	-	HMDB001	Organic ac	Carboxylic	Amino acic	65-82-7	190.0538	3.2797	M-H	C7H13NO	94.5	0
-	-	-	HMDB025	Organic ac	Carboxylic	Amino acids, peptides		790.4133	5.1083	M+H	C41H55N7	0	91.6
-	-	-	HMDB009	Organic ac	Carboxylic	Amino acids, peptides		260.114	3.7045	M+FA-H	C10H17NC	0	61.5
-	-	-	-	-	-	-		286.1774	3.8124	M-H	C13H25N3	88.6	0
-	-	-	-	-	-	-		129.0183	2.5718	M-H2O-H	C5H8O5	58.9	0
-	-	-	HMDB003	Organic o:	Organoox	Ethers	99964-83-	915.4293	4.5921	2M+FA-H	C25H29N3	0	93
-	-	-	HMDB003	Organohe	Naphthop	Not Availa	-;471-54-!	376.1757	5.6249	M+NH4	C20H22O6	0	60.2
-	-	-	-	-	-	-		214.087	4.4385	M-H	C13H13NC	51.5	0
-	-	-	HMDB002	Organic ac	Carboxylic	Amino acids, peptides		247.1108	3.5918	M+H	C10H18N2	79.8	0
-	-	-	HMDB003	Lipids and	Prenol lipi	Terpene gl	73565-59-	886.4124	2.9619	M+ACN+I	C42H62O1	0	66.7
-	-	-	HMDB024	Organic ac	Carboxylic	Amino acids, peptides		251.1599	0.5141	M+NH4	C10H19NC	50.7	0
-	-	-	HMDB025	Organic o:	Organoox	Carbohydr	73196-97-	487.2876	3.0896	M+CH3OI	C18H36N6	0	45.6
Metabolic	Metabolisr	Global anc	HMDB000	Organic o:	Organoox	Carbohydr	69-65-8	181.071	0.6318	M-H	C6H14O6	54	0
-	-	-	HMDB024	Organohe	Pyridines a	Hydropyridines		532.2148	3.195	M+Na-2H	C26H35F2	0	49.8
-	-	-	-	-	-	-		343.1987	3.8359	M-H	C15H28N4	41.1	0
-	-	-	HMDB026	Organic ac	Carboxylic	Amino acids, peptides		227.1147	0.8677	M-H2O-H	C9H18N4C	0	48.4
-	-	-	-	-	-	-		410.1593	5.6009	M+H	C24H19N5	70.3	0
-	-	-	HMDB001	Nucleoside	Nucleoside	Cyclopenty	136470-78	331.151	4.4075	M+FA-H	C14H18N6	0	59.6
-	-	-	-	-	-	-		304.1337	3.0408	M-H	C20H19NC	49.2	0
Quorum s	Cellular Pr	Cellular co	HMDB034	-	-	-	192883-14	242.1397	5.8624	M-H	C12H21NC	0	51.5
-	-	-	-	-	-	-		332.2177	3.2493	M+H	C15H29N3	77	0
-	-	-	HMDB028	Not Availa	Not Availa	Not Available		854.5394	5.8905	M+H-2H2	C49H80NC	0	72.7
-	-	-	-	-	-	-		317.218	1.8403	M+H	C14H28N4	43.6	0
-	-	-	-	-	-	-		241.0082	0.6404	M+H	C11H9ClO	67.5	0
-	-	-	-	-	-	-		221.0928	1.5101	M+H	C11H12N2	59	0
-	-	-	HMDB000	Benzenoid	Benzene a	Not Availa	103-84-4;!	180.0659	5.8472	M+FA-H	C8H9NO	0	52.2
Metabolic	Metabolisr	Global anc	HMDB030	Lipids and	Prenol lipi	Diterpenoi	38076-57-	421.1835	2.956	M+Hac-H	C20H26O6	0	62.2
-	-	-	-	-	-	-		341.109	0.6936	M-H	C12H22O1	94.5	0
Metabolic	Metabolisr	Global anc	HMDB003	Phenylpro	Isoflavono	Isoflavono	552-66-9;	417.1176	3.7351	M+H	C21H20O9	66.3	0
-	-	-	HMDB003	Alkaloids a	Not Availa	Not Availa	499-04-7	327.1564	3.9437	2M+FA-H	C7H11NO	0	50.2
-	-	-	HMDB025	Lipids and	Prenol lipi	Diterpenoi	101312-92	599.33	4.7067	M+Cl	C31H52N2	0	59.7
-	-	-	-	-	-	-		244.129	2.0934	M+H	C10H17N3	37.1	0
-	-	-	-	-	-	-		385.2442	5.0277	M+H	C22H29FN	42	0
-	-	-	HMDB003	Lipids and	Prenol lipi	Terpene gl	100156-32	841.4426	4.4747	M+H-H2C	C44H68O1	0	55.4
-	-	-	HMDB004	Lipids and	Fatty Acyls	Fatty acyl glycosides		292.1177	2.117	M+	C12H20O8	0	65.9

-	-	-	HMDB000	Organohe	Pyridines a	Not Availa	501-81-5	182.0452	2.8714	M+FA-H	C7H7NO2	43	0
-	-	-	-	-	-	-		447.2451	3.1936	M+H	C23H34N4	46.6	0
-	-	-	HMDB000	Organic ox	Organooxy	Carbohydr	53188-23-	161.0447	2.8485	M-H2O-H	C6H12O6	81.8	0
-	-	-	-	-	-	-		346.2333	4.1322	M+H	C16H31N3	91.7	0
-	-	-	HMDB025	Organic ac	Carboxylic	Amino acids, peptides		229.1544	4.053	M+H	C11H20N2	76.2	0
-	-	-	HMDB024	Organohe	Benzofura	Not Available		312.1201	2.5102	M+Na-2H	C16H21NO	0	51.1
Aminoben	Metabolism	Xenobiotic	HMDB000	Benzenoid	Benzene a	Benzoic ac	121-34-6	167.0344	7.3739	M-H	C8H8O4	42.4	0
-	-	-	HMDB002	Organic ox	Organooxy	Carbohydr	34323-07-	288.1055	0.6879	M+H	C12H17NO	0	53.2
Biosynthes	Metabolism	Global and	HMDB030	Lipids and	Prenol lipi	Diterpenoi	510-75-8;	389.1572	3.6351	M+Hac-H	C19H22O5	0	64.4
-	-	-	-	-	-	-		340.2242	4.2611	M-H	C17H31N3	87.2	0
-	-	-	HMDB025	Phenylpro	Tetracyclin	Not Available		615.3044	5.6148	M+Hac-H	C29H40N4	0	81.2
-	-	-	HMDB025	Alkaloids a	Cytochalas	Cytochalasins		516.2563	5.6689	M+Cl	C29H39NO	0	73.1
-	-	-	HMDB025	Benzenoid	Benzene a	Phenethyla	18910-65-	390.1881	2.9715	M+Hac-H	C19H25NO	0	41.7
-	-	-	HMDB003	Organohe	Pyrrolidine	Not Availa	80933-73-	224.0926	5.1435	M+FA-H	C10H13NO	0	40.4
-	-	-	HMDB034	-	-	-		441.1994	3.5811	2M-H	C12H15NO	0	40.5
-	-	-	-	-	-	-		211.0864	5.8423	M+H	C13H10N2	94.7	0
-	-	-	-	-	-	-		344.1828	3.9359	M-H	C15H27N3	69.1	0
-	-	-	-	-	-	-		327.131	2.028	M-H2O-H	C13H22N4	48.9	0
-	-	-	HMDB002	Organic ac	Carboxylic	Amino acids, peptides		240.0988	2.3109	M-H2O-H	C10H17N3	0	48.3
-	-	-	-	-	-	-		332.1464	2.5947	M-H	C13H23N3	83.2	0
-	-	-	HMDB034	-	-	-		325.1406	5.491	M+Hac-H	C13H18N2	0	56.1
Biosynthes	Metabolism	Lipid meta	HMDB000	Lipids and	Fatty Acyls	Fatty acids	-;5598-38	309.2784	7.3247	M+H	C20H36O2	54.8	0
-	-	-	HMDB006	Lipids and	Steroids ar	Bile acids,	14772-95-	452.2751	5.9147	M+ACN+N	C24H36O4	0	42.1
-	-	-	HMDB002	Organic ac	Carboxylic	Amino acids, peptides		203.0668	0.7014	M-H	C7H12N2O	80.9	0
-	-	-	-	-	-	-		174.1124	1.1933	M+H	C8H15NO3	76.1	0
Metabolic	Metabolism	Global and	HMDB000	Benzenoid	Phenols	Benzenedi	102-32-9	167.0343	5.3135	M-H	C8H8O4	87.7	0
-	-	-	-	-	-	-		350.1722	4.0444	M-H	C17H25N3	79.1	0
-	-	-	HMDB030	Organic ac	Carboxylic	Amino acids, peptides		501.2931	4.2688	2M+FA-H	C11H20N2	0	95.5
Metabolic	Metabolism	Global and	HMDB025	Organic ac	Carboxylic	Amino acid	34522-32-	227.1147	0.6936	M-H2O-H	C9H18N4O	0	54.7
-	-	-	HMDB003	Benzenoid	Benzene a	Toluenes	193976-63	978.5618	4.9872	M+NH4	C48H80O1	0	44.5
-	-	-	-	-	-	-		499.1798	1.967	2M-H	C12H14N2	38.3	0
-	-	-	-	-	-	-		219.0507	2.9099	M-H	C8H12O7	40.5	0
-	-	-	-	-	-	-		447.2199	1.8403	M+H	C22H30N4	49.7	0
-	-	-	-	-	-	-		330.2383	3.7033	M+H	C16H31N3	87.1	0
-	-	-	-	-	-	-		134.0812	0.7037	M+H	C5H11NO3	50.3	0
-	-	-	-	-	-	-		167.0342	4.2534	M-H	C8H8O4	80.3	0
-	-	-	HMDB002	Organic ac	Carboxylic	Amino acid	14486-05-	219.0806	1.9518	M-H	C8H16N2O	76.3	0
-	-	-	HMDB025	Organic ac	Carboxylic	Amino acids, peptides		748.3686	3.3131	M+Na	C35H51N9	0	54.4

-	-	-	HMDB002	Benzenoid	Benzene a	Benzoic acids and de		394.1984	5.7619	M+FA-H	C18H27N3	0	41.7
-	-	-	HMDB006	Lipids and	Steroids ar	Estrane steroids		453.1994	5.3368	M-H2O-H	C25H32N2	0	72.5
-	-	-	HMDB001	Organohet	Benzazepi	Dibenzaze	303-49-1	315.1656	1.4704	M+H	C19H23ClN	70.6	0
-	-	-	HMDB024	Lipids and	Prenol lipi	Diterpenoids		689.3004	3.1259	M+Na-2H	C36H48N2	0	84.6
-	-	-	-	-	-	-		403.0995	2.7486	M-H	C19H20N2	64.3	0
-	-	-	-	-	-	-		314.2088	4.3769	M-H	C15H29N3	42.6	0
-	-	-	-	-	-	-		430.1624	5.6458	M-H	C21H25N3	57.5	0
-	-	-	HMDB034	-	-	-		359.1262	5.5373	M+Cl	C17H24O6	0	45.9
Type I poly	Metabolisr	Metabolisr	HMDB001	Organic ox	Organooxy	Carbohydr	7681-93-8	724.3171	4.1062	M+Hac-H	C33H47NO	0	56.3
Metabolic	Metabolisr	Global anc	-	-	-	-		334.1046	3.9901	M-H	C12H21N3	68	0
-	-	-	-	-	-	-		154.0501	7.3971	M-H2O-H	C7H11NO4	42	0
-	-	-	-	-	-	-		258.146	3.0254	M-H	C11H21N3	85.3	0
Cysteine a	Metabolisr	Amino aci	HMDB000	Organic ac	Carboxylic	Amino aci	4289-98-9	176.038	3.0799	M-H	C6H11NO3	95.2	0
-	-	-	-	-	-	-		334.1775	4.7677	M-H	C17H25N3	89.5	0
-	-	-	HMDB003	Organic ox	Organooxy	Carbohydr	70954-04-	280.1039	0.6552	M-H	C10H19NO	0	44.4
-	-	-	-	-	-	-		437.2047	5.6303	M-H	C20H30N4	71.3	0
-	-	-	HMDB030	Organic ac	Carboxylic	Amino acids, peptide		310.2139	5.5992	M-H2O-H	C16H31N3	0	47
-	-	-	HMDB001	Benzenoid	Benzene a	Diphenylm	58957-92-	542.163	2.4791	M+FA-H	C26H27NO	0	41.8
-	-	-	HMDB034	-	-	-		367.1499	2.4829	M+H	C17H22N2	0	74.2
-	-	-	HMDB024	Lipids and	Steroids ar	Estrane steroids		339.1563	5.6458	M+Na-2H	C19H26O4	0	46
-	-	-	HMDB000	Organic ac	Carboxylic	Amino aci	5657-19-2	168.0294	2.61	M-H	C7H7NO4	75.5	0
-	-	-	-	-	-	-		215.0728	3.2028	M-H	C13H12O3	76.4	0
-	-	-	-	-	-	-		334.1775	4.9745	M-H	C17H25N3	70.9	0
-	-	-	HMDB004	Lipids and	Prenol lipi	Monoterp	155416-22	617.2289	5.6846	M-H	C37H34N2	0	40.1
Metabolic	Metabolisr	Global anc	HMDB000	Organic ox	Organooxy	Carbonyl c	-;1194-98	137.0234	3.3337	M-H	C7H6O3	75.2	0
-	-	-	HMDB034	-	-	-		410.2282	5.8022	M+ACN+N	C21H30O4	0	44.5
Metabolic	Metabolisr	Global anc	HMDB000	Benzenoid	Phenols	Benzenedi	51-43-4	184.0969	2.5469	M+H	C9H13NO3	81.8	0
-	-	-	HMDB002	Organic ac	Carboxylic	Amino acids, peptide		300.1704	5.3262	M+H-H2O	C17H23N3	67.9	0
-	-	-	-	-	-	-		323.0286	1.2087	M-H	C9H13N2O	51.4	0
-	-	-	-	-	-	-		537.2618	5.2213	M+H	C25H36N4	39	0
-	-	-	-	-	-	-		301.1406	4.2225	M+Hac-H	C11H18N2	40.7	0
Metabolic	Metabolisr	Global anc	HMDB000	Organohet	Tetrapyrro	Porphyrins	7412-77-3	601.3433	4.2754	M+CH3OH	C34H40N4	0	53.1
Metabolic	Metabolisr	Global anc	HMDB000	Nucleoside	Purine nuc	Not Availa	146-80-5	283.0686	2.4947	M-H	C10H12N4	96.6	0
Metabolic	Metabolisr	Global anc	HMDB030	Lipids and	Prenol lipi	Diterpenoi	19427-32-	367.1512	5.6846	M+Na-2H	C20H26O5	0	42.3
-	-	-	-	-	-	-		364.1881	4.7219	M-H	C18H27N3	74.8	0
-	-	-	HMDB003	Phenylpro	Diarylhept	Cyclic diar	191999-64	385.1618	3.9901	M+Hac-H	C20H22O4	0	62.9
-	-	-	-	-	-	-		137.0458	1.2962	M+H	C4H8O5	43.2	0
-	-	-	-	-	-	-		144.0293	1.786	M-H	C5H7NO4	42.7	0

-	-	-	HMDB004	Organohe	Pyrrolizine	Not Availa	97073-10-	250.1085	5.7774	M+FA-H	C12H15N(	0	40.6
-	-	-	HMDB025	Organic ac	Carboxylic	Amino aci	87980-11-	323.1249	3.5271	2M+FA-H	C7H9NO2	0	51.6
-	-	-	-	-	-	-		303.1337	2.8899	M+H	C16H18N2	53.4	0
-	-	-	-	-	-	-		441.1862	2.1404	M+H	C18H28N6	39.6	0
-	-	-	HMDB005	Benzenoid	Benzene a	Phenylacetamides		290.1036	4.5618	M+FA-H	C14H15N(	0	48.9
-	-	-	-	-	-	-		456.1816	5.6303	M+Na-2H	C26H29N(	41.8	0
-	-	-	-	-	-	-		184.0973	5.1976	M-H	C9H15NO:	74.7	0
-	-	-	-	-	-	-		180.0295	3.6967	M-H	C8H7NO4	83	0
-	-	-	-	-	-	-		349.1393	2.4829	M+H	C12H20N4	52.9	0
-	-	-	HMDB000	Organic ac	Carboxylic	Amino aci	83462-55-	393.1781	4.6378	M-H2O-H	C18H28N4	0	43
-	-	-	HMDB002	Organic ac	Carboxylic	Amino acids, peptide:		214.1185	1.1694	M+H-H2C	C9H17N3(	0	62.2
-	-	-	-	-	-	-		153.1021	3.2174	M+H	C8H12N2(	86.3	0
-	-	-	HMDB025	Nucleoside	Purine nuc	Not Available		673.2694	2.956	2M-H	C14H19N5	0	48.9
-	-	-	-	-	-	-		128.1071	1.1854	M+H	C7H13NO	42.2	0
-	-	-	-	-	-	-		330.1672	2.8099	M-H	C14H25N3	78.2	0
-	-	-	-	-	-	-		375.1411	3.2566	M+Hac-H	C18H20O5	87.8	0
-	-	-	HMDB000	Organohe	Oxepanes	Not Availa	498-07-7	221.0663	0.7014	M+Hac-H	C6H10O5	60.1	0
-	-	-	-	-	-	-		267.0723	0.7014	M+FA-H	C8H14O7	53	0
-	-	-	-	-	-	-		395.1667	1.1854	M+H-H2C	C16H24N6	65.8	0
-	-	-	HMDB001	Organohe	Piperidines	Benzylpiperidines		424.2093	5.6458	M+Hac-H	C23H27N(	0	58
-	-	-	-	-	-	-		290.0883	1.4045	M-H	C12H13N5	47.1	0
-	-	-	HMDB034	-	-	-	16118-49-	281.1144	4.2146	M+FA-H	C12H16N2	0	54.2
-	-	-	HMDB003	Organic ac	Carboxylic	Tetracarbc	25161-41-	513.2318	3.7272	M+CH3OH	C24H32O1	0	43.6
-	-	-	-	-	-	-		332.1814	2.1722	M+H	C14H25N3	54.7	0
-	-	-	HMDB000	Lipids and	Fatty Acyls	Fatty acids	77220-86-	163.0604	0.7093	M+FA-H	C5H10O3	52.4	0
-	-	-	HMDB001	Organic ac	Carboxylic	Amino aci	13589-02-	263.1387	3.7192	M+H	C14H18N2	89.2	0
-	-	-	HMDB024	Phenylpro	Brevetoxin	Not Availa	11032-79-	877.4654	4.9391	M+H-H2C	C50H70O1	0	43.5
-	-	-	-	-	-	-		343.097	4.6837	M-H	C17H16N2	52.6	0
-	-	-	-	-	-	-		389.1205	2.5947	M+Cl	C15H22N4	53.9	0
-	-	-	HMDB034	-	-	-		239.0671	1.9972	M-H2O-H	C10H14N2	0	47.3
-	-	-	-	-	-	-		375.1274	0.6327	M+H	C16H22O1	72.4	0
-	-	-	-	-	-	-		153.1022	2.8021	M+H	C8H12N2(	80.7	0
-	-	-	HMDB002	Organic ac	Carboxylic	Amino acids, peptide:		317.1256	5.7001	M-H	C15H18N4	61.7	0
Metabolic	Metabolisr	Global anc	HMDB000	Organohe	Lactones	Gamma bu	979-92-0	383.1144	1.9972	M-H	C14H20N6	81	0
-	-	-	HMDB030	Alkaloids a	Not Availa	Not Availa	551-59-7;_	184.0973	4.7903	M+FA-H	C8H13NO	0	49.2
Metabolic	Metabolisr	Global anc	HMDB000	Organic ac	Carboxylic	Amino aci	-;_	168.027	0.6165	M+Na-2H	C5H9NO4	0	45.3
-	-	-	-	-	-	-		156.1019	2.5786	M+H-H2C	C8H15NO:	65.3	0
-	-	-	HMDB003	Organic o	Organooxy	Carbohydrates and ca		462.1192	3.6504	M+Cl	C19H25N(	0	47.8

-	-	-	HMDB025	Nucleoside	Pyrimidine	Not Available		229.0827	1.0183	M-H2O-H	C9H16N2O	0	49.2
-	-	-	-	-	-	-		374.1571	3.357	M-H	C15H25N3	86.6	0
-	-	-	HMDB000	Lipids and	Fatty Acyls	Fatty acids	541-47-9	145.0497	2.1048	M+FA-H	C5H8O2	69	0
Metabolic	Metabolism	Global and	HMDB000	Organic ac	Hydroxy ac	Beta hydro	51-34-3	304.154	5.7782	M+H	C17H21NO	59.3	0
-	-	-	-	-	-	-		215.0877	0.5219	M+H	C12H11BO	36.1	0
-	-	-	HMDB025	Organohet	Piperidines	Piperidinecarboxylic a		228.1602	5.8316	M-H	C12H23NO	0	49.9
-	-	-	HMDB024	Lipids and	Steroids ar	Steroidal glycosides		557.2944	2.8563	M+FA-H	C27H44O9	0	67.1
-	-	-	HMDB025	Alkaloids a	Ergoline ar	Lysergic acids and de		627.3231	3.2413	M+ACN+N	C31H41N5	0	51.7
-	-	-	HMDB003	Lipids and	Prenol lipi	Terpene gl	90851-24-	523.2623	2.642	M+K	C26H44O8	0	53.7
-	-	-	-	-	-	-		151.0965	1.9665	M+H	C7H10N4	46.6	0
-	-	-	-	-	-	-		257.1507	5.6611	M-H	C12H22N2	38.6	0
-	-	-	-;HMDB02	Lipids and	Steroids ar	Hydroxyste	4419-39-0	409.1814	1.4863	M+H	C22H29ClO	50.1	0
-	-	-	-	-	-	-		158.0964	3.3211	M+H-H2O	C11H13NO	86.9	0
Metabolic	Metabolism	Global and	HMDB000	Organohet	Pyridines a	Pyridine ca	66-72-8	166.0502	5.7156	M-H	C8H9NO3	0	44
-	-	-	HMDB000	Lipids and	Steroids ar	Steroidal g	25577-70-	509.2374	5.5282	M+CH3OH	C25H32O9	0	46.2
-	-	-	-	-	-	-		446.2048	5.0436	M-H	C21H29N5	57.9	0
-	-	-	HMDB006	Organic ox	Organooxy	Carbonyl c	59578-62-	375.156	5.7001	2M+FA-H	C9H11NO	0	41.4
-	-	-	HMDB003	Lipids and	Prenol lipi	Terpene gl	204927-92	491.2518	5.7619	M+Hac-H	C21H36O9	0	51.7
-	-	-	HMDB001	Organohet	Quinolines	Hydroquin	51781-06-	337.1771	5.7697	M+FA-H	C16H24N2	0	46.3
-	-	-	HMDB004	Organic ac	Peptidomi	Depsipept	73561-91-	590.3201	5.7542	M+Na-2H	C37H47NO	0	41.7
Metabolic	Metabolism	Global and	HMDB030	Benzenoid	Benzene a	Benzoic ac	1571-72-8	154.0498	5.3422	M+H	C7H7NO3	66.7	0
-	-	-	HMDB025	Organohet	Indoles an	Indolecarboxylic acids		540.2318	5.1512	M+FA-H	C25H32F3	0	85.4
Metabolic	Metabolism	Global and	HMDB000	Lipids and	Fatty Acyls	Fatty acids	124-04-9;	111.0443	0.9643	M+H-2H2	C6H10O4	52	0
-	-	-	-	-	-	-		264.109	0.6858	M+Hac-H	C9H11N5O	46.9	0
-	-	-	-	-	-	-		130.0863	0.972	M+H	C6H11NO	65.8	0
-	-	-	-	-	-	-		356.1465	3.3881	M-H	C18H22F3	46.5	0
-	-	-	HMDB002	Organic ac	Carboxylic	Amino acid	13187-90-	217.0826	1.8613	M-H	C8H14N2O	41.6	0
-	-	-	HMDB025	Nucleoside	Nucleoside	Cyclopentyl nucleosid		262.0933	2.7639	M-H	C11H13N5	0	60.7
-	-	-	-	-	-	-		546.2882	2.0301	M+H	C22H39N7	43	0
-	-	-	-	-	-	-		685.4877	4.3149	2M-H	C17H33N3	72.7	0
-	-	-	HMDB003	Organic ox	Organooxy	Carbohydr	75589-40-	355.1249	0.6552	M-H	C13H24O1	0	43.6

Metab ID	ID	Mode	RSD	Score	Mass Error	mcs1903_r	mcs1903_S	mrs_mean	mrs_SD
metab_113	pos_11500	pos		57.5		1.81	0.207	6.171	0.01148
metab_210	neg_4594	neg		48.3		2.795	0.3712	6.913	0.01981
metab_114	pos_11699	pos		56		2.084	0.004933	5.8	0.01229
metab_207	neg_4352	neg		50.4		3.519	0.01216	7.038	0.01561
metab_100	pos_10252	pos		42.4		1.752	0.8163	5.409	0.02649
metab_182	neg_1839	neg		52.4		3.962	0.3083	7.225	0.01068
metab_104	pos_10601	pos		45.5		2.071	1.133	5.544	0.04635
metab_430	pos_4413	pos		50.9		1.966	0.555	5.082	0.04074
metab_214	neg_5075	neg		50.4		3.436	0.06271	6.469	0.009216
metab_211	neg_4727	neg		47		6.462	0.05283	3.416	0.2377
metab_211	neg_4695	neg		48.6		4.328	0.05266	7.291	0.01629
metab_118	pos_12022	pos		45.8		6.35	0.01963	3.32	0.5803
metab_219	neg_5582	neg		52.7		3.516	0.01041	6.444	0.006361
metab_192	neg_2778	neg		45.7		5.755	0.02045	2.833	0.1584
metab_192	neg_2834	neg		51.6		5.514	0.05183	2.469	0.9548
metab_183	neg_1879	neg		49.6		3.828	0.3056	6.649	0.01871
metab_458	pos_4700	pos		58.5		3.787	0.3578	6.606	0.01033
metab_173	neg_912	neg		51.9		2.42	0.5595	5.281	0.04162
metab_182	neg_1796	neg		50.3		4.038	0.1124	6.761	0.01627
metab_144	pos_1511	pos		52.1		6.291	0.008996	3.272	1.203
metab_170	neg_553	neg		47.1		7.902	0.008332	5.234	0.02365
metab_213	neg_4967	neg		49.7		3.895	0.3456	6.59	0.003698
metab_228	pos_2372	pos		49.6		3.405	0.1682	6.028	0.01916
metab_203	neg_3889	neg		56.4		6.658	0.02772	4.053	0.05099
metab_195	neg_3077	neg		51.8		3.924	0.01041	6.501	0.01959
metab_188	neg_2405	neg		53.2		2.972	0.0104	5.518	0.009324
metab_224	neg_6100	neg		56.6		3.613	0.05467	6.158	0.01482
metab_178	neg_1381	neg		49.3		6.47	0.006933	3.964	0.1705
metab_180	neg_1543	neg		49.2		4.313	0.1244	6.775	0.01197
metab_150	pos_15284	pos		49.6		4.049	0.3232	6.528	0.00813
metab_190	neg_2636	neg		50.2		3.558	0.01041	6.002	0.01533
metab_519	pos_5328	pos		45.9		4.142	0.1269	6.583	0.02041
metab_173	neg_908	neg		47.2		4.098	0.1915	6.53	0.01587
metab_137	pos_14017	pos		55.8		2.919	0.4009	5.391	0.01209
metab_114	pos_11678	pos		58.3		3.689	0.218	6.119	0.01193
metab_621	pos_6367	pos		48		5.145	0.01595	2.762	0.2107
metab_193	neg_2943	neg		44.4		5.59	0.04682	3.24	0.2044

metab_648	pos_6640	pos	44.9	4.864	0.03571	2.309	0.88
metab_223	neg_5999	neg	49.2	3.716	0.01041	6.034	0.03299
metab_111	pos_11306	pos	43.3	3.797	0.4231	6.152	0.0288
metab_199	neg_3513	neg	55.5	5.348	0.05132	3.087	0.05699
metab_210	neg_4582	neg	48.2	6.192	0.02708	3.935	0.1609
metab_103	pos_10536	pos	51.5	2.989	0.2333	5.245	0.01728
metab_230	neg_6709	neg	56	2.919	0.4207	5.215	0.0383
metab_185	neg_2106	neg	54.7	3.81	0.01041	6.029	0.01763
metab_173	neg_844	neg	42.4	6.077	0.02344	3.848	0.2165
metab_130	pos_1347	pos	50.4	3.792	0.004974	5.994	0.02834
metab_112	pos_11456	pos	46.7	4.297	0.321	6.525	0.03907
metab_839	pos_865	pos	49.6	4.562	0.117	6.758	0.006803
metab_213	neg_4945	neg	44.5	6.086	0.01449	3.885	0.2131
metab_910	pos_9265	pos	55.5	5.17	0.1282	2.984	0.005727
metab_192	neg_2804	neg	54	3.95	0.01041	6.129	0.01102
metab_210	neg_4632	neg	47.9	5.823	0.05127	3.638	0.1983
metab_192	neg_2810	neg	55	4.465	0.2658	6.652	0.01071
metab_231	neg_6770	neg	57.9	7.158	0.007092	5.012	0.1585
metab_170	neg_594	neg	54.9	4.495	0.1852	6.642	0.006359
metab_229	neg_6526	neg	46.7	3.954	0.2031	6.102	0.01044
metab_101	pos_10271	pos	42.4	4.231	0.1854	6.366	0.03007
metab_198	neg_3463	neg	52.4	3.013	0.5932	5.244	0.01035
metab_156	pos_1636	pos	46.3	5.459	0.03924	3.309	0.3373
metab_210	neg_4614	neg	58.3	4.344	0.2538	6.474	0.01281
metab_904	pos_9205	pos	52.7	5.046	0.105	2.919	0.2213
metab_101	pos_10294	pos	49.5	5.277	0.02304	3.159	0.2148
metab_213	neg_4971	neg	54.8	5.342	0.0842	7.442	0.006961
metab_119	pos_12182	pos	55.5	3.389	0.6799	5.635	0.01532
metab_842	pos_8587	pos	43.7	3.518	0.02049	5.611	0.06863
metab_184	neg_1975	neg	45.5	4.445	0.09844	6.535	0.012
metab_196	neg_3215	neg	45.9	5.153	0.03676	3.067	0.08921
metab_169	neg_474	neg	47.7	5.504	0.01642	3.435	0.004899
metab_100	pos_10238	pos	48.7	3.002	0.1783	5.074	0.0313
metab_164	pos_16718	pos	43.7	4.258	0.09043	6.317	0.02547
metab_217	neg_5389	neg	50	3.774	0.1858	5.841	0.01209
metab_479	pos_4917	pos	55.9	4.126	0.1629	6.184	0.00816
metab_687	pos_706	pos	57.6	5.752	0.09037	7.795	0.0124
metab_207	neg_4335	neg	47.9	6.411	0.01248	4.372	0.04448

metab_517	pos_5313	pos	55.1	3.549	0.004973	5.586	0.01431
metab_169	neg_493	neg	42.4	5.894	0.044	3.834	0.259
metab_130	pos_1352	pos	55.5	5.135	0.1078	7.166	0.005537
metab_192	neg_2779	neg	55.3	6.899	0.01025	4.878	0.0376
metab_194	neg_2995	neg	56	4.232	0.06684	6.251	0.01653
metab_203	neg_3913	neg	45.9	5.195	0.05342	3.178	0.004898
metab_174	neg_938	neg	44.9	3.54	0.4234	5.618	0.0291
metab_120	pos_12301	pos	48.9	3.532	0.1394	5.549	0.0124
metab_210	neg_4658	neg	46.9	5.295	0.0398	3.272	0.2094
metab_169	pos_1769	pos	49	5.7	0.03217	3.646	0.3799
metab_188	neg_2409	neg	57.1	3.812	0.09133	5.805	0.0104
metab_170	neg_605	neg	41.6	4.528	0.1833	6.526	0.02493
metab_137	pos_14018	pos	47.8	4.987	0.01445	3.001	0.1215
metab_179	neg_1538	neg	49.4	4.378	0.01041	6.355	0.02225
metab_150	pos_15285	pos	56.6	4.876	0.08336	6.855	0.009646
metab_618	pos_6336	pos	48.3	4.158	0.07823	6.133	0.01837
metab_188	neg_2382	neg	51.1	3.692	0.09384	5.664	0.02282
metab_218	neg_5491	neg	48	3.96	0.235	5.95	0.01149
metab_210	neg_4649	neg	49.9	4.946	0.3072	6.952	0.0179
metab_211	neg_4747	neg	57.6	8.448	0.01068	6.479	0.07572
metab_193	neg_2934	neg	49.1	6.169	0.04128	4.173	0.2726
metab_223	neg_5941	neg	58.2	4.005	0.2819	5.999	0.01694
metab_228	neg_6467	neg	55.1	6.305	0.01427	4.346	0.09651
metab_227	neg_6367	neg	54.3	4.077	0.01041	6.032	0.01361
metab_140	pos_1467	pos	39.9	4.338	0.1471	6.293	0.02653
metab_188	neg_2441	neg	59.1	5.751	0.1221	7.7	0.006517
metab_175	neg_1078	neg	50.4	3.898	0.2878	5.874	0.02924
metab_175	neg_1092	neg	54.8	5.521	0.01948	3.6	0.13
metab_232	neg_6903	neg	49.7	5.669	0.06409	3.754	0.1979
metab_190	neg_2571	neg	51.3	4.409	0.2677	6.335	0.01407
metab_138	pos_1446	pos	44.5	5.821	0.02613	3.904	0.2128
metab_184	neg_1992	neg	46	3.976	0.2297	5.888	0.004575
metab_193	neg_2856	neg	48.1	4.494	0.01041	6.381	0.01636
metab_478	pos_490	pos	52.3	4.696	0.06969	6.579	0.02056
metab_208	neg_4440	neg	45.8	4.717	0.01041	6.597	0.01974
metab_122	pos_12506	pos	46.5	3.347	0.05838	5.222	0.02278
metab_214	neg_4992	neg	56.5	4.003	0.1896	5.887	0.01109
metab_219	neg_5510	neg	48.9	4.119	0.01041	5.98	0.01216

metab_192	neg_2826	neg	48.5	3.757	0.01041	5.61	0.01807
metab_226	neg_6286	neg	55.8	3.84	0.08296	5.692	0.02895
metab_124	pos_12700	pos	45.7	5.855	0.009726	4	0.1176
metab_137	pos_14019	pos	55.7	6.95	0.01034	5.104	0.01015
metab_171	neg_647	neg	55.9	4.089	0.1581	5.945	0.01006
metab_135	pos_1405	pos	50.9	6.142	0.02055	4.296	0.1074
metab_125	pos_1296	pos	56.6	4.715	0.1011	6.552	0.01781
metab_229	neg_6612	neg	49.6	3.577	0.2284	5.423	0.02628
metab_218	neg_5413	neg	56.9	4.291	0.108	6.113	0.0171
metab_420	pos_4312	pos	47.2	5.03	0.0159	3.207	0.1102
metab_190	neg_2599	neg	54.8	3.895	0.01041	5.71	0.01017
metab_618	pos_6331	pos	52.5	4.623	0.02543	2.806	0.07182
metab_212	neg_4881	neg	49.6	5.272	0.02684	3.459	0.06269
metab_194	neg_2962	neg	48.4	4.523	0.1968	6.348	0.01263
metab_117	pos_11987	pos	44	5.646	0.04401	3.787	0.3588
metab_210	neg_4643	neg	58.6	8.186	0.007468	6.382	0.06892
metab_128	pos_13044	pos	47.4	5.443	0.02257	3.632	0.137
metab_187	neg_2297	neg	53.8	4.095	0.0405	5.894	0.02572
metab_149	pos_15235	pos	48.8	5.047	0.03433	3.235	0.1997
metab_218	neg_5450	neg	50.3	4.283	0.1785	6.091	0.0165
metab_231	neg_6729	neg	57.4	4.802	0.2854	6.629	0.01765
metab_193	neg_2926	neg	46.4	4.505	0.01041	6.294	0.01812
metab_954	pos_9712	pos	44.7	3.714	0.0881	5.501	0.04666
metab_173	neg_899	neg	47	4.621	0.1717	6.415	0.009623
metab_130	pos_1348	pos	48.5	4.051	0.1582	5.839	0.04859
metab_110	pos_11276	pos	51.8	3.266	0.03231	5.044	0.04635
metab_213	neg_4926	neg	47.3	4.105	0.4174	5.954	0.02654
metab_453	pos_4655	pos	54	3.936	0.2056	5.725	0.006088
metab_222	neg_5865	neg	50.3	4.531	0.1468	6.308	0.03208
metab_101	pos_10267	pos	52.1	4.825	0.02172	3.042	0.1878
metab_215	neg_5146	neg	44.2	6.043	0.0519	4.268	0.1413
metab_195	neg_3126	neg	46.2	5.463	0.01041	7.227	0.01452
metab_173	neg_884	neg	54.4	4.651	0.1398	6.424	0.008125
metab_143	pos_1498	pos	54.6	5.017	0.05659	6.781	0.01167
metab_196	neg_3244	neg	50.7	6.308	0.02357	4.545	0.07204
metab_159	pos_16213	pos	47	4.064	0.0886	5.827	0.01373
metab_232	neg_6871	neg	50	5.625	0.07134	3.848	0.2043
metab_316	pos_3267	pos	49.6	4.27	0.2998	6.064	0.02238

metab_169	neg_499	neg	45.8	3.689	0.01041	5.44	0.01813
metab_105	pos_1094	pos	53.8	5.482	0.06812	7.234	0.008756
metab_139	pos_14156	pos	53.6	4.739	0.07487	6.489	0.01479
metab_209	neg_4536	neg	52.6	4.62	0.0533	2.87	0.04857
metab_170	neg_507	neg	48.9	5.728	0.04624	7.457	0.01507
metab_190	neg_2642	neg	47.2	4.565	0.01041	6.289	0.01818
metab_111	pos_11344	pos	57.6	4.565	0.2703	6.322	0.01127
metab_193	neg_2885	neg	58.3	6.544	0.008781	4.82	0.03621
metab_155	pos_15821	pos	48.6	4.645	0.1127	6.373	0.01855
metab_182	neg_1830	neg	49.1	4.141	0.1074	5.868	0.02526
metab_226	neg_6254	neg	48.1	4.26	0.2652	6.004	0.0165
metab_210	neg_4598	neg	44.6	4.287	0.01279	5.997	0.04068
metab_203	neg_3884	neg	47.3	3.438	0.05898	5.141	0.03869
metab_170	neg_558	neg	53.6	4.58	0.2675	6.309	0.006878
metab_149	pos_15239	pos	57.3	7.664	0.01927	5.969	0.01116
metab_183	neg_1941	neg	48.2	4.431	0.02458	6.119	0.01508
metab_137	pos_14023	pos	47.1	4.764	0.01241	3.033	0.3131
metab_100	pos_10223	pos	47.9	5.207	0.02382	3.515	0.1689
metab_189	neg_2522	neg	50.7	3.943	0.7888	5.837	0.01401
metab_706	pos_7224	pos	49.3	5.295	0.1166	3.609	0.1405
metab_193	neg_2924	neg	51.8	3.898	0.0709	5.566	0.02602
metab_180	neg_1557	neg	57.5	3.922	0.1088	5.593	0.01764
metab_234	neg_7125	neg	49.3	6.439	0.08527	8.102	0.01733
metab_222	neg_5888	neg	46.9	4.407	0.1397	6.07	0.01371
metab_455	pos_466	pos	47.1	5.367	0.01959	3.7	0.1558
metab_232	neg_6877	neg	46.9	7.432	0.02604	5.776	0.0716
metab_185	neg_2067	neg	55	3.61	0.1455	5.274	0.01706
metab_193	neg_2873	neg	52.3	4.076	0.08722	5.731	0.02107
metab_521	pos_5349	pos	45	3.948	0.004974	5.594	0.03283
metab_128	pos_1324	pos	54.8	4.42	0.2083	6.082	0.02812
metab_221	neg_5770	neg	55.3	7.59	0.01039	5.95	0.05078
metab_180	neg_1615	neg	51.1	5.051	0.01041	6.687	0.02069
metab_225	neg_6162	neg	52.3	4.048	0.01041	5.683	0.01075
metab_171	neg_689	neg	56.6	4.12	0.05507	5.747	0.02226
metab_226	neg_6255	neg	55.7	4.437	0.3362	6.112	0.00748
metab_258	pos_2672	pos	43.8	6.33	0.0142	4.706	0.08883
metab_287	pos_2977	pos	50.9	4.952	0.06899	6.566	0.009982
metab_431	pos_4423	pos	44.8	5.646	0.02213	3.905	0.5547

metab_96(	pos_9830	pos	49.6	3.235	0.5478	4.979	0.04486
metab_19:	neg_2739	neg	51.7	5.096	0.06582	6.709	0.01604
metab_14(	pos_14869	pos	51.2	5.107	0.05971	6.715	0.009963
metab_19(	neg_2560	neg	45.4	5.091	0.05561	3.453	0.2533
metab_15	pos_15779	pos	45.9	5.041	0.1258	6.651	0.007313
metab_13(	pos_13896	pos	57.4	4.904	0.04471	6.504	0.008759
metab_15(	pos_15283	pos	59.3	6.286	0.0351	7.878	0.007237
metab_18:	neg_1664	neg	48.5	5.619	0.02013	3.996	0.2542
metab_13:	pos_13617	pos	52.5	5.008	0.01759	3.381	0.2801
metab_23	neg_6898	neg	44.8	5.326	0.07135	3.725	0.1394
metab_17	neg_1498	neg	47.7	4.141	0.01041	5.727	0.01738
metab_17:	neg_870	neg	51.6	3.798	0.1826	5.399	0.01515
metab_22	neg_6496	neg	57.7	4.923	0.01041	6.504	0.01601
metab_10	pos_10396	pos	51.8	4.539	0.1441	6.128	0.03482
metab_21(	neg_4641	neg	47.7	6.816	0.01652	5.234	0.08486
metab_11	pos_11433	pos	47.3	4.643	0.1198	6.226	0.01708
metab_22	neg_6416	neg	50.5	4.542	0.01041	6.118	0.02074
metab_15(	pos_15328	pos	46.5	5.524	0.04431	7.099	0.006145
metab_40	pos_4166	pos	56.2	3.619	0.2781	5.226	0.01626
metab_33	pos_3452	pos	50.9	5.467	0.02635	7.031	0.009542
metab_14	pos_15061	pos	56.1	5.752	0.06854	7.316	0.006959
metab_13:	pos_13378	pos	47.5	4.362	0.09709	5.929	0.02663
metab_23:	neg_6973	neg	54.4	4.074	0.199	5.654	0.01039
metab_17	neg_779	neg	55.4	4.69	0.1768	6.263	0.007847
metab_13	pos_13999	pos	50.8	4.062	0.1361	5.63	0.02125
metab_40	pos_4193	pos	51.7	4.963	0.05512	6.52	0.007773
metab_17	neg_1435	neg	45.9	4.191	0.2633	5.779	0.03681
metab_15	pos_15532	pos	53.3	6.086	0.01187	4.533	0.06621
metab_21	neg_5344	neg	47.6	4.224	0.05105	5.773	0.0184
metab_13(	pos_1351	pos	57.2	5.68	0.03422	7.228	0.009114
metab_19	neg_3480	neg	52.8	3.367	0.6816	5.105	0.06338
metab_17	neg_1403	neg	54.8	5.517	0.07607	7.059	0.01929
metab_14	pos_14651	pos	51.3	4.956	0.05935	6.497	0.01317
metab_12	pos_12456	pos	46.2	5.618	0.02022	4.076	0.08704
metab_14	pos_1524(	pos	58.9	8.213	0.02394	6.682	0.005523
metab_14	pos_15156	pos	52	5.064	0.1408	6.598	0.005596
metab_20	neg_4358	neg	54	6.055	0.01041	7.575	0.0254
metab_15(	pos_1527(	pos	55.8	4.933	0.1519	6.462	0.00651

metab_17[illegible]	neg_1006	neg	48.9	4.532	0.1098	6.049	0.02431
metab_17[illegible]	neg_528	neg	46	4.06	0.1507	5.58	0.02223
metab_19[illegible]	neg_2608	neg	47.2	5.608	0.05818	7.11	0.009023
metab_11[illegible]	pos_12142	pos	55	3.454	0.1852	4.972	0.01428
metab_21[illegible]	neg_5092	neg	44.3	5.645	0.03129	4.134	0.1493
metab_11[illegible]	pos_1237	pos	57.6	4.56	0.07851	6.062	0.009824
metab_10[illegible]	pos_11088	pos	47.8	4.887	0.1976	6.408	0.03248
metab_21[illegible]	neg_5031	neg	43.5	4.144	0.08498	5.646	0.02111
metab_89[illegible]	pos_9115	pos	57.1	3.852	0.09167	5.363	0.1308
metab_90[illegible]	pos_9186	pos	44.5	4.753	0.3278	6.305	0.08364
metab_19[illegible]	neg_3273	neg	53.8	3.73	0.1644	5.241	0.01679
metab_25[illegible]	pos_2688	pos	53.4	5.287	0.01435	6.781	0.01075
metab_10[illegible]	pos_10677	pos	45.3	5.828	0.0306	7.323	0.04623
metab_12[illegible]	pos_12584	pos	51.2	4.404	0.0467	5.898	0.01281
metab_17[illegible]	neg_896	neg	46.8	4.529	0.01041	6.021	0.01468
metab_21[illegible]	neg_4629	neg	47.7	4.352	0.01041	5.844	0.00879
metab_19[illegible]	neg_2817	neg	47.1	4.108	0.03462	5.6	0.03037
metab_18[illegible]	neg_2190	neg	47.9	6.537	0.008023	5.059	0.04165
metab_17[illegible]	neg_655	neg	43.6	4.211	0.01041	5.686	0.02855
metab_23[illegible]	neg_6865	neg	49.8	4.176	0.01041	5.648	0.03391
metab_21[illegible]	neg_5128	neg	50.4	4.19	0.2369	5.691	0.02208
metab_15[illegible]	pos_15439	pos	46.5	5.729	0.0302	4.232	0.2262
metab_21[illegible]	neg_5297	neg	43.5	4.012	0.01041	5.481	0.02097
metab_10[illegible]	pos_1047	pos	54.1	5	0.05533	6.469	0.01155
metab_23[illegible]	neg_6750	neg	57.8	5.215	0.1819	6.701	0.003094
metab_23[illegible]	neg_6802	neg	47.1	3.965	0.01041	5.433	0.05052
metab_22[illegible]	neg_5708	neg	52.2	4.994	0.1022	6.464	0.006017
metab_13[illegible]	pos_13381	pos	48	4.776	0.07557	6.243	0.01218
metab_18[illegible]	neg_2289	neg	58.7	5.308	0.0487	6.771	0.008516
metab_15[illegible]	pos_1604	pos	46.8	4.131	0.2343	5.623	0.01547
metab_22[illegible]	neg_5698	neg	58.2	6.543	0.004091	5.084	0.02625
metab_18[illegible]	neg_1765	neg	45.4	3.773	0.01041	5.232	0.03251
metab_13[illegible]	pos_1350	pos	58	5.125	0.04697	6.583	0.009212
metab_21[illegible]	neg_4928	neg	48.1	5.576	0.0282	4.094	0.2267
metab_44[illegible]	pos_4536	pos	47.6	4.419	0.08041	5.871	0.01852
metab_19[illegible]	neg_2952	neg	55.4	4.036	0.2395	5.513	0.01742
metab_23[illegible]	neg_6848	neg	48.4	4.191	0.01041	5.634	0.03603
metab_13[illegible]	pos_14021	pos	58.5	7.302	0.01207	5.86	0.008095

metab_128	pos_13027	pos	45	4.029	0.1745	5.487	0.01657
metab_146	pos_14933	pos	54.7	5.692	0.0247	7.129	0.008366
metab_174	neg_962	neg	44.9	4.073	0.6571	5.692	0.02796
metab_209	neg_4545	neg	44	4.497	0.5538	6.071	0.02098
metab_174	neg_939	neg	46.1	4.104	0.01016	5.536	0.02455
metab_149	pos_15253	pos	53.9	4.87	0.06039	6.296	0.01443
metab_228	neg_6499	neg	44.2	4.66	0.109	6.092	0.01358
metab_185	neg_2047	neg	54.5	4.465	0.1786	5.906	0.01372
metab_182	neg_1789	neg	45.9	3.729	0.01175	5.148	0.01686
metab_215	neg_5156	neg	56.1	5.945	0.01065	4.523	0.1168
metab_286	pos_2966	pos	44	4.584	0.08797	5.997	0.0141
metab_226	neg_6227	neg	55.7	5.113	0.07729	6.522	0.01546
metab_371	pos_3825	pos	55.4	4.889	0.05887	6.294	0.01547
metab_173	neg_927	neg	51.6	5.228	0.1142	6.638	0.007701
metab_170	neg_525	neg	48.8	4.39	0.1397	5.801	0.02071
metab_174	neg_946	neg	54.1	5.049	0.03835	6.448	0.01689
metab_189	neg_2461	neg	49.7	4.3	0.05304	5.699	0.02126
metab_132	pos_13443	pos	57.8	4.926	0.03863	6.322	0.009264
metab_136	pos_13913	pos	56.5	5.309	0.02914	6.699	0.01022
metab_231	neg_6781	neg	54.3	5.521	0.03125	6.911	0.01154
metab_187	neg_2260	neg	49.3	5.221	0.06861	6.611	0.009378
metab_183	neg_1916	neg	49.2	6.594	0.005714	5.209	0.01995
metab_124	pos_12657	pos	57.1	4.675	0.04994	6.059	0.01796
metab_149	pos_15234	pos	55.6	6.856	0.02696	5.478	0.0119
metab_145	pos_14758	pos	54.3	4.6	0.0541	5.979	0.01939
metab_137	pos_13985	pos	46	5.406	0.04006	3.987	0.2699
metab_225	neg_6196	neg	46.7	4.86	0.1419	6.248	0.008937
metab_178	neg_1377	neg	46	3.807	0.04078	5.177	0.01023
metab_137	pos_13963	pos	46.1	4.765	0.1447	6.145	0.02135
metab_149	pos_15189	pos	49.3	5.172	0.04269	6.541	0.01634
metab_180	neg_1599	neg	49.7	4.847	0.06767	6.216	0.008248
metab_137	pos_13988	pos	47.7	5.507	0.01729	4.132	0.1247
metab_214	neg_5020	neg	56	4.559	0.2798	5.967	0.01085
metab_181	neg_1647	neg	50.5	4.458	0.2819	5.869	0.01678
metab_209	neg_4572	neg	50.5	6.79	0.02419	5.426	0.01207
metab_219	neg_5514	neg	44.6	5.42	0.01969	4.043	0.1688
metab_106	pos_10850	pos	50.5	4.519	0.3745	5.954	0.04022
metab_280	pos_2900	pos	45	4.552	0.1142	5.92	0.0166

metab_146	pos_14887	pos	51.2	4.545	0.1725	5.92	0.03724
metab_124	pos_1286	pos	49.3	5.686	0.02694	7.04	0.01031
metab_856	pos_883	pos	46.6	5.388	0.04417	6.742	0.006857
metab_188	neg_2416	neg	57.3	4.821	0.1043	6.176	0.008003
metab_221	neg_5766	neg	55	4.184	0.198	5.552	0.01907
metab_219	neg_5588	neg	54.2	6.543	0.01514	5.199	0.02962
metab_225	neg_6163	neg	50.1	5.468	0.02805	4.103	0.1957
metab_170	neg_585	neg	55.5	4.721	0.1199	6.07	0.009431
metab_207	neg_4355	neg	44.3	4.885	0.04986	3.498	0.2897
metab_194	neg_3045	neg	52.3	4.566	0.2827	5.948	0.02234
metab_270	pos_2797	pos	45.7	4.407	0.07918	5.742	0.01906
metab_174	neg_981	neg	57	4.77	0.3097	6.15	0.01756
metab_151	pos_15389	pos	48.9	3.895	0.2591	5.259	0.03012
metab_211	neg_4708	neg	48.8	5.569	0.07532	6.893	0.004038
metab_398	pos_4092	pos	43.8	5.266	0.04228	3.865	0.3861
metab_297	pos_3073	pos	46.4	4.297	0.08383	5.621	0.01919
metab_191	neg_2663	neg	48.9	5.468	0.1252	4.129	0.1286
metab_212	neg_4869	neg	48.6	5.15	0.2012	6.493	0.01945
metab_230	pos_2392	pos	53.8	5.424	0.03804	4.081	0.2152
metab_215	neg_5183	neg	49.7	5.468	0.04021	4.112	0.2648
metab_287	pos_2978	pos	50.2	4.171	0.2128	5.511	0.01655
metab_215	neg_5133	neg	53.2	4.93	0.1587	6.254	0.009421
metab_228	neg_6480	neg	47.9	4.284	0.01041	5.591	0.0206
metab_231	neg_6801	neg	45.7	6.374	0.0454	5.062	0.1202
metab_123	pos_12546	pos	44.7	4.48	0.1141	5.79	0.00964
metab_981	pos_9983	pos	49.1	4.498	0.04482	3.179	0.1665
metab_234	neg_7048	neg	53.5	6.402	0.02703	5.102	0.03814
metab_177	neg_1295	neg	57.8	5.52	0.04075	6.815	0.01873
metab_662	pos_6782	pos	47.3	3.847	0.2863	5.186	0.04392
metab_199	neg_3516	neg	49.5	6.573	0.03212	5.285	0.02644
metab_167	neg_278	neg	48.8	5.49	0.04083	4.195	0.105
metab_170	neg_536	neg	50.6	4.107	0.01041	5.392	0.04399
metab_891	pos_9082	pos	43.2	3.949	0.08305	5.243	0.1036
metab_234	neg_7035	neg	47.4	5.048	0.06478	3.737	0.2083
metab_113	pos_11531	pos	46.5	6.2	0.01955	4.886	0.2393
metab_183	neg_1859	neg	46.7	4.092	0.3206	5.432	0.0195
metab_203	neg_3904	neg	49.4	5.925	0.03695	4.646	0.04214
metab_161	pos_16424	pos	56.9	5.48	0.01222	4.203	0.03687

metab_181	neg_1705	neg	46.4	5.084	0.01966	3.787	0.1873
metab_843	pos_8596	pos	43.4	5.256	0.1249	6.54	0.02768
metab_224	neg_6023	neg	50.1	5.475	0.03926	6.75	0.01068
metab_176	neg_1151	neg	45.6	5.435	0.03829	4.158	0.07333
metab_224	neg_6099	neg	49.4	4.85	0.07556	6.12	0.01243
metab_241	pos_2505	pos	57.2	5.236	0.04829	6.502	0.01163
metab_219	pos_228	pos	57.1	6.393	0.01268	5.13	0.01367
metab_903	pos_9199	pos	45.5	5.396	0.2807	6.709	0.07833
metab_176	neg_1175	neg	57.8	5.44	0.05493	6.702	0.01253
metab_230	neg_6684	neg	53.1	3.94	0.01041	5.2	0.01548
metab_173	neg_902	neg	58.4	7.426	0.01789	6.165	0.02143
metab_141	pos_14353	pos	43.7	4.655	0.1071	5.922	0.02688
metab_177	neg_1269	neg	57.2	5.445	0.05052	6.699	0.01433
metab_172	neg_737	neg	55.4	4.444	0.1318	5.703	0.009041
metab_130	pos_13236	pos	53.5	4.279	0.1463	5.541	0.02524
metab_219	neg_5513	neg	52	4.579	0.1103	5.835	0.0154
metab_730	pos_7465	pos	52.7	3.649	0.1521	4.927	0.1583
metab_211	neg_4686	neg	49.5	4.977	0.07424	6.229	0.02249
metab_188	neg_2430	neg	42.8	4.525	0.4318	5.877	0.04609
metab_183	neg_1892	neg	50.1	4.051	0.09418	5.303	0.02936
metab_188	neg_2379	neg	45.7	5.523	0.03085	4.278	0.017
metab_134	pos_13636	pos	48.7	4.635	0.06127	5.88	0.008576
metab_161	pos_16416	pos	50.9	3.585	0.1972	4.853	0.01777
metab_230	neg_6681	neg	49.9	6.814	0.012	5.571	0.02397
metab_207	neg_4310	neg	56.6	5.294	0.04836	6.538	0.01547
metab_125	pos_12730	pos	52.1	4.753	0.07003	5.997	0.01212
metab_206	neg_4225	neg	51.2	4.342	0.1588	5.599	0.03227
metab_216	neg_5286	neg	58.4	5.395	0.02931	6.636	0.008466
metab_143	pos_14609	pos	47.6	4.827	0.149	6.08	0.01966
metab_180	neg_1601	neg	50.2	5.071	0.09224	6.309	0.01148
metab_215	neg_5103	neg	45.6	5.916	0.026	4.682	0.04042
metab_170	neg_606	neg	50.8	4.717	0.01041	5.949	0.01553
metab_223	neg_5923	neg	55.1	5.128	0.04972	6.358	0.01457
metab_231	neg_6751	neg	48	6.937	0.007115	5.709	0.005825
metab_181	neg_1698	neg	56.6	5.102	0.08688	6.323	0.01038
metab_171	neg_651	neg	50.8	4.597	0.08519	5.818	0.02752
metab_208	neg_4389	neg	45.1	3.822	0.3224	5.101	0.03824
metab_896	pos_9132	pos	56.7	4.624	0.08519	5.848	0.1013

metab_183	neg_1901	neg	56.6	5.053	0.0684	6.266	0.01769
metab_228	neg_6502	neg	54.8	4.982	0.1474	6.205	0.01273
metab_197	neg_3360	neg	51.9	4.453	0.3071	5.72	0.02503
metab_111	pos_11316	pos	43.5	5.04	0.4025	6.341	0.02545
metab_229	neg_6533	neg	55.4	4.748	0.2317	5.992	0.02079
metab_234	neg_7100	neg	52.8	4.797	0.05982	6.004	0.0195
metab_191	neg_2681	neg	47.9	4.4	0.1258	5.612	0.01857
metab_230	neg_6657	neg	58.1	6.009	0.03014	7.209	0.01378
metab_149	pos_15209	pos	42.5	5.497	0.07514	3.782	1.363
metab_172	neg_812	neg	52.4	4.706	0.2255	5.935	0.01117
metab_444	pos_4557	pos	53	4.877	0.06627	6.075	0.03663
metab_177	neg_1294	neg	56.2	5.396	0.03741	6.588	0.01699
metab_110	pos_11275	pos	53.9	5.127	0.091	6.319	0.009879
metab_190	neg_2606	neg	45.3	4.731	0.1657	5.935	0.02087
metab_189	neg_2505	neg	47.8	5.622	0.0147	4.434	0.0683
metab_220	neg_5656	neg	50.1	4.607	0.3768	5.877	0.02703
metab_199	neg_3495	neg	52.6	5.313	0.03801	4.124	0.08282
metab_206	neg_4251	neg	57.2	5.511	0.03756	6.695	0.0136
metab_237	neg_7366	neg	55.9	6.014	0.1168	4.809	0.142
metab_221	neg_5697	neg	54.3	5.846	0.02868	7.026	0.01094
metab_427	pos_4389	pos	50.8	4.078	0.1435	5.269	0.03293
metab_458	pos_4707	pos	47.7	4.619	0.2624	5.833	0.02666
metab_234	neg_7073	neg	55.2	3.945	0.132	5.125	0.03162
metab_203	neg_3943	neg	57	6.594	0.01295	5.427	0.02123
metab_124	pos_12623	pos	45.9	6.142	0.03448	4.968	0.1009
metab_173	neg_922	neg	51.8	5.309	0.2028	6.501	0.01757
metab_164	pos_16755	pos	47.9	5.63	0.1019	4.441	0.1632
metab_127	pos_1314	pos	53.9	4.458	0.1222	5.625	0.04482
metab_178	neg_1402	neg	50.5	5.144	0.06636	6.303	0.01967
metab_736	pos_7517	pos	58	5.433	0.08938	6.597	0.08661
metab_192	neg_2849	neg	48.3	7.605	0.01386	6.454	0.01235
metab_724	pos_7396	pos	49.1	4.258	0.1724	3.069	0.1671
metab_160	pos_16330	pos	44.7	4.555	0.04655	3.383	0.1799
metab_180	neg_1603	neg	52.7	5.716	0.02129	6.863	0.01571
metab_222	pos_232	pos	51.2	3.536	0.2253	4.712	0.02417
metab_310	pos_3208	pos	51	5.368	0.04294	4.215	0.1209
metab_201	pos_2091	pos	53.1	4.444	0.09443	5.585	0.0179
metab_207	neg_4356	neg	47.7	4.441	0.2597	5.618	0.03202

metab_409	pos_4203	pos	47	6.17	0.009171	5.037	0.03542
metab_316	pos_3265	pos	54.6	4.523	0.08901	5.659	0.02456
metab_203	neg_3876	neg	57.7	5.331	0.04117	4.199	0.03391
metab_137	pos_13931	pos	57.3	5.569	0.03376	6.685	0.01005
metab_582	pos_5972	pos	51.5	5.179	0.03577	4.063	0.03126
metab_172	neg_753	neg	46.5	4.455	0.26	5.612	0.01529
metab_228	neg_6464	neg	47.2	4.564	0.01041	5.674	0.02343
metab_729	pos_7454	pos	54.2	5.817	0.0959	4.696	0.09981
metab_112	pos_11443	pos	45.7	5.225	0.1249	6.342	0.01647
metab_151	pos_15408	pos	49	5.31	0.0338	6.416	0.02731
metab_232	neg_6839	neg	49	5.475	0.05481	4.37	0.004901
metab_211	neg_4782	neg	46.8	4.598	0.3296	5.771	0.01687
metab_201	neg_3685	neg	46.3	5.922	0.03429	4.82	0.04863
metab_199	neg_3518	neg	55.4	5.633	0.03639	4.533	0.03321
metab_231	neg_6771	neg	42.3	5.433	0.04608	4.244	0.3802
metab_205	neg_4124	neg	56.3	5.778	0.02508	4.678	0.05628
metab_737	pos_7526	pos	43.5	5.096	0.06717	6.205	0.1036
metab_211	neg_4776	neg	49	4.6	0.2266	5.734	0.02851
metab_120	pos_1240	pos	46	4.801	0.06837	5.899	0.01889
metab_112	pos_1167	pos	52.2	4.969	0.1212	6.066	0.02951
metab_172	neg_800	neg	50.2	5.728	0.01974	6.808	0.01193
metab_194	neg_3014	neg	49.6	4.172	0.2056	5.283	0.0296
metab_113	pos_11559	pos	49.3	4.871	0.1074	5.955	0.03858
metab_183	neg_1914	neg	52.2	5.583	0.02401	6.658	0.01606
metab_151	pos_15468	pos	52.6	5.914	0.0244	6.988	0.008722
metab_125	pos_12760	pos	57.2	4.283	0.1087	5.365	0.0174
metab_210	neg_4656	neg	56.7	4.259	0.01041	5.331	0.02871
metab_231	neg_6774	neg	46.9	4.475	0.01041	5.546	0.04049
metab_217	pos_226	pos	57	6.226	0.01023	5.157	0.02324
metab_111	pos_1150	pos	56.7	5.205	0.03178	6.269	0.01353
metab_166	pos_1741	pos	48.1	5.072	0.05316	6.136	0.02019
metab_226	neg_6305	neg	46.4	4.387	0.169	5.472	0.05344
metab_608	pos_6229	pos	47.5	4.697	0.02657	3.626	0.117
metab_123	pos_12557	pos	49.3	4.443	0.06523	5.501	0.02643
metab_194	neg_3055	neg	56.3	3.941	0.09953	5.004	0.03164
metab_297	pos_3074	pos	44.7	5.963	0.01592	4.901	0.1119
metab_122	pos_12480	pos	41.7	5.246	0.03634	4.165	0.2018
metab_167	neg_264	neg	45.6	5.794	0.05682	6.845	0.01471

metab_175	neg_1034	neg	44.6	5.672	0.01325	4.624	0.02602
metab_720	pos_739	pos	51.4	5.925	0.02883	6.972	0.02623
metab_207	neg_4340	neg	45.7	4.286	0.01041	5.329	0.01475
metab_111	pos_11309	pos	47.4	5.453	0.02536	4.409	0.04625
metab_231	neg_6725	neg	45.4	5.27	0.07999	6.314	0.01626
metab_173	pos_1807	pos	52.6	5.431	0.03491	4.383	0.1206
metab_170	neg_576	neg	47	5.071	0.2441	6.15	0.01766
metab_169	neg_411	neg	49.5	5.014	0.04058	6.05	0.01447
metab_185	neg_2124	neg	49.4	5.72	0.04411	4.667	0.1489
metab_230	neg_6646	neg	53.9	5.255	0.03365	6.289	0.01455
metab_479	pos_4913	pos	44.5	6.235	0.02319	5.201	0.06802
metab_171	pos_1787	pos	52.8	5.71	0.01896	6.738	0.0129
metab_148	pos_15145	pos	52.1	4.739	0.1188	5.776	0.02516
metab_597	pos_6122	pos	49.1	5.104	0.103	6.135	0.01876
metab_158	pos_16102	pos	47.1	5.169	0.0291	6.191	0.01624
metab_336	pos_3469	pos	53.9	5.809	0.01829	6.826	0.008277
metab_168	neg_395	neg	55.7	5.526	0.01979	4.508	0.04821
metab_220	neg_5606	neg	49.3	5.374	0.04631	6.388	0.005655
metab_226	neg_6231	neg	51.1	5.749	0.03607	4.737	0.06883
metab_171	neg_652	neg	50	6.659	0.01351	5.655	0.01014
metab_336	pos_3474	pos	42.6	5.66	0.0259	4.628	0.1859
metab_208	neg_4412	neg	46.9	5.673	0.01041	6.676	0.01493
metab_737	pos_7534	pos	45.9	4.696	0.2075	5.732	0.07808
metab_199	neg_3515	neg	55.5	5.971	0.04082	4.969	0.03595
metab_168	neg_398	neg	57.9	6.913	0.01434	5.914	0.02245
metab_208	neg_4442	neg	52.1	5.738	0.03039	6.738	0.01387
metab_287	pos_2976	pos	50	6.201	0.02008	7.198	0.006099
metab_161	pos_16467	pos	36.9	5.814	0.0921	4.808	0.06053
metab_231	neg_6738	neg	50.4	4.497	0.1952	5.52	0.01718
metab_213	neg_4897	neg	44.6	4.091	0.2556	5.126	0.0343
metab_209	neg_4552	neg	55.6	6.031	0.03504	7.017	0.009433
metab_149	pos_15205	pos	53.2	7.228	0.01912	6.243	0.01388
metab_170	neg_603	neg	47.8	5.515	0.04438	6.497	0.01395
metab_661	pos_6766	pos	43.8	4.344	0.1532	5.338	0.01681
metab_174	neg_995	neg	57.2	6.051	0.0147	5.079	0.0121
metab_371	pos_3826	pos	46.7	5.204	0.02881	4.224	0.09893
metab_128	pos_13092	pos	53.6	4.452	0.09865	5.43	0.02044
metab_904	pos_9209	pos	48.7	6.148	0.1327	7.135	0.07484

metab_147	pos_14993	pos	47.1	6.269	0.01031	5.298	0.05608
metab_139	pos_14171	pos	57.3	5.633	0.0143	6.6	0.01374
metab_197	neg_3354	neg	58.9	7.61	0.01818	6.643	0.01134
metab_173	neg_914	neg	48	5.495	0.03368	4.522	0.08099
metab_209	neg_4569	neg	56.1	6.088	0.02616	7.055	0.009476
metab_232	neg_6836	neg	50	5.718	0.06165	6.687	0.03481
metab_911	pos_9279	pos	46.5	5.88	0.174	4.888	0.07722
metab_179	neg_1482	neg	56.8	5.28	0.02138	4.318	0.04485
metab_181	pos_1889	pos	50.8	5.5	0.03751	4.517	0.1707
metab_531	pos_5449	pos	42.9	4.983	0.09998	5.947	0.02289
metab_168	pos_1752	pos	49.4	5.803	0.04151	4.84	0.1133
metab_161	pos_16463	pos	54.3	5.629	0.0977	4.664	0.09598
metab_977	pos_9936	pos	50.6	3.496	0.2684	4.501	0.04743
metab_232	neg_6854	neg	55.8	4.936	0.01041	5.882	0.007032
metab_181	neg_1708	neg	50.1	5.835	0.02482	4.886	0.0516
metab_630	pos_6457	pos	45.8	4.917	0.02693	5.864	0.02719
metab_206	neg_4175	neg	47.5	5.669	0.05966	6.612	0.01075
metab_192	neg_2794	neg	48.2	4.386	0.01041	5.322	0.02233
metab_298	pos_3085	pos	45.5	4.636	0.04564	5.572	0.017
metab_170	neg_532	neg	50.8	4.715	0.2103	5.685	0.02865
metab_184	neg_2030	neg	48.8	6.379	0.01673	7.311	0.01154
metab_214	neg_5044	neg	50.2	5.075	0.05633	4.118	0.1605
metab_187	neg_2284	neg	48.2	4.926	0.1167	5.869	0.01599
metab_118	pos_1224	pos	47.3	5.928	0.03612	4.996	0.07006
metab_218	neg_5473	neg	46.6	5.502	0.06048	6.431	0.02099
metab_728	pos_7437	pos	44.6	5.862	0.09678	4.923	0.09362
metab_222	neg_5878	neg	43.2	4.999	0.1983	5.955	0.03635
metab_190	neg_2583	neg	45.3	5.6	0.03804	4.676	0.06773
metab_186	neg_2181	neg	48.9	6.653	0.01301	5.737	0.01353
metab_213	neg_4934	neg	44.3	5.667	0.03356	4.747	0.05927
metab_209	neg_4524	neg	51.4	4.005	0.1634	4.943	0.04655
metab_208	neg_4448	neg	48.4	5.673	0.0344	4.756	0.04731
metab_153	pos_15589	pos	49	5.512	0.01407	6.421	0.01042
metab_186	neg_2189	neg	52.8	6.15	0.01476	5.24	0.02402
metab_697	pos_7131	pos	52.9	4.787	0.05238	3.86	0.1425
metab_653	pos_6691	pos	55.8	5.762	0.04445	4.853	0.03844
metab_187	neg_2343	neg	51.1	5.518	0.07048	6.428	0.01224
metab_137	pos_1431	pos	51.4	5.863	0.0192	4.956	0.05081

metab_224	neg_6059	neg	58.1	5.534	0.03492	6.439	0.0116
metab_132	pos_13523	pos	57.7	5.238	0.01414	6.141	0.003838
metab_190	neg_2569	neg	46.7	6.351	0.02706	7.254	0.008547
metab_184	neg_2034	neg	53	5.091	0.02242	4.188	0.04046
metab_161	pos_16426	pos	47.6	4.828	0.01713	3.931	0.02088
metab_214	neg_5008	neg	46.3	6.562	0.008046	5.665	0.04144
metab_903	pos_9198	pos	44.3	5.933	0.1507	6.85	0.07407
metab_140	pos_14289	pos	45.2	5.15	0.05684	6.044	0.01073
metab_194	neg_3024	neg	47.1	6.026	0.02214	5.137	0.02105
metab_222	neg_5801	neg	58.2	6.467	0.01505	7.355	0.008129
metab_653	pos_6684	pos	50.1	4.303	0.06813	3.384	0.1846
metab_231	neg_6790	neg	53.2	5.785	0.01041	6.671	0.01462
metab_632	pos_6476	pos	48.9	3.702	0.1705	4.614	0.03228
metab_464	pos_4760	pos	45.9	4.474	0.1701	5.385	0.02731
metab_188	neg_2435	neg	46.7	4.966	0.05872	4.057	0.152
metab_100	pos_10243	pos	47.4	4.707	0.09145	5.599	0.03829
metab_231	neg_6788	neg	55.1	6.23	0.0155	5.347	0.02872
metab_231	neg_6797	neg	52.6	5.021	0.1888	5.932	0.03273
metab_194	neg_3059	neg	49.4	5.344	0.04043	4.464	0.06278
metab_302	pos_3129	pos	45.8	5.641	0.03619	4.762	0.06219
metab_207	neg_4360	neg	46.6	5.347	0.08941	6.225	0.01809
metab_167	pos_1746	pos	52	7.62	0.01289	6.751	0.01801
metab_187	neg_2341	neg	49	4.925	0.1444	5.812	0.01663
metab_622	pos_6375	pos	49.6	4.406	0.06356	5.277	0.02402
metab_407	pos_4185	pos	58.4	6.123	0.02849	5.257	0.01448
metab_226	neg_6253	neg	49.1	5.414	0.04054	6.281	0.01885
metab_205	neg_4157	neg	45.7	4.296	0.2005	5.197	0.03034
metab_233	pos_2415	pos	48.6	6.172	0.01898	5.308	0.02403
metab_228	neg_6465	neg	54.8	4.734	0.1593	5.619	0.02065
metab_897	pos_9143	pos	43	4.516	0.1866	5.417	0.1094
metab_214	neg_5076	neg	46.4	4.749	0.08402	5.616	0.02767
metab_216	neg_5280	neg	43.6	5.483	0.0515	4.616	0.06837
metab_196	neg_3232	neg	43.4	5.313	0.1768	6.2	0.02709
metab_210	neg_4650	neg	55.7	4.761	0.3097	5.693	0.01914
metab_192	neg_2829	neg	47	4.645	0.1374	5.519	0.01765
metab_219	neg_5501	neg	55.2	5.172	0.03399	6.029	0.02041
metab_167	neg_272	neg	49.2	6.276	0.1564	7.162	0.1138
metab_193	neg_2958	neg	51.5	5.959	0.0214	5.105	0.0373

metab_282	pos_2922	pos	48.9	5.768	0.03753	4.913	0.0365
metab_228	neg_6474	neg	48	6.205	0.0164	5.353	0.01542
metab_227	neg_6378	neg	47.3	5.195	0.07767	6.052	0.01259
metab_166	neg_123	neg	52.3	5.922	0.03648	5.068	0.02996
metab_654	pos_6702	pos	55.9	5.951	0.08928	5.079	0.1169
metab_600	pos_6153	pos	46	5.092	0.02241	4.24	0.04039
metab_234	neg_7092	neg	49.3	5.931	0.02857	5.08	0.03348
metab_211	neg_4694	neg	48.2	5.471	0.09496	6.325	0.01397
metab_231	neg_6814	neg	46.2	5.2	0.1034	4.306	0.2116
metab_207	neg_4367	neg	46.5	4.454	0.01041	5.298	0.02236
metab_980	pos_9972	pos	56.1	4.66	0.03972	3.798	0.1425
metab_184	neg_1995	neg	44.2	5.833	0.01558	4.991	0.01675
metab_111	pos_11317	pos	42.6	5.895	0.03008	5.052	0.06858
metab_173	neg_857	neg	48.2	5.441	0.05648	6.28	0.01836
metab_167	pos_1744	pos	54.2	5.789	0.007362	4.953	0.03822
metab_175	neg_1061	neg	38.8	5.798	0.08298	6.639	0.01689
metab_139	pos_14218	pos	46.5	5.541	0.01532	4.701	0.09412
metab_193	neg_2915	neg	48.6	5.321	0.125	6.163	0.02511
metab_194	neg_2990	neg	54.2	5.039	0.09381	5.874	0.01962
metab_312	pos_3224	pos	50.4	6.057	0.02002	5.232	0.02198
metab_526	pos_5398	pos	44.4	4.697	0.1785	5.551	0.02175
metab_232	neg_6868	neg	53.3	6.136	0.03382	5.311	0.0426
metab_145	pos_14796	pos	49.8	5.414	0.01841	6.236	0.004167
metab_212	neg_4806	neg	46.7	5.445	0.01041	6.266	0.02074
metab_226	neg_6208	neg	54.7	5.023	0.01614	4.198	0.07003
metab_218	neg_5493	neg	50.2	5.385	0.02653	4.563	0.04663
metab_121	pos_12349	pos	54.3	4.888	0.02397	4.061	0.09459
metab_228	neg_6451	neg	44.4	5.196	0.1672	6.039	0.0133
metab_153	pos_15580	pos	47.7	7.28	0.01594	6.462	0.01145
metab_206	neg_4231	neg	49.5	4.976	0.1013	5.804	0.03305
metab_232	neg_6863	neg	57.1	7.209	0.01414	6.392	0.02156
metab_209	neg_4514	neg	48.2	5.329	0.0716	6.151	0.01874
metab_169	neg_485	neg	49.1	5.141	0.1299	4.286	0.1607
metab_218	neg_5468	neg	54	5.825	0.009333	6.64	0.0194
metab_198	neg_3430	neg	45.5	5.489	0.0356	4.672	0.04742
metab_670	pos_6855	pos	46.3	4.357	0.1351	5.189	0.05543
metab_194	neg_2991	neg	49.8	5.537	0.05744	4.718	0.06873
metab_152	pos_15495	pos	52.1	5.913	0.01228	6.724	0.009965

metab_223	neg_5931	neg	53.5	5.075	0.1192	5.899	0.02317
metab_172	neg_799	neg	48.5	5.811	0.01813	5	0.02113
metab_170	neg_511	neg	44.9	6.031	0.05563	6.842	0.02013
metab_443	pos_4545	pos	48.5	4.362	0.08654	5.181	0.06766
metab_198	neg_3442	neg	45.2	6.087	0.05276	5.273	0.06847
metab_133	pos_13571	pos	50.2	6.474	0.014	5.668	0.02027
metab_149	pos_15228	pos	44.5	4.686	0.3196	5.575	0.04902
metab_727	pos_7430	pos	47.2	5.97	0.09306	5.152	0.08206
metab_127	pos_1321	pos	46.9	5.18	0.06208	4.371	0.05187
metab_102	pos_10431	pos	45.8	4.679	0.01993	3.868	0.09437
metab_226	neg_6276	neg	43.7	6.059	0.02801	6.862	0.0511
metab_179	neg_1511	neg	48.1	5.472	0.02584	4.669	0.07217
metab_211	neg_4697	neg	46.5	6.037	0.04027	5.24	0.01696
metab_233	neg_6958	neg	46	5.088	0.09655	5.894	0.03701
metab_106	pos_10839	pos	48	4.168	0.1846	4.997	0.04621
metab_177	neg_1307	neg	43.8	5.007	0.0765	5.806	0.0312
metab_252	pos_2618	pos	46.3	5.764	0.01371	4.97	0.03339
metab_233	neg_7005	neg	58.2	6.401	0.03414	5.605	0.04689
metab_193	neg_2913	neg	47.9	5.484	0.02578	4.691	0.04125
metab_232	neg_6910	neg	49.3	6.85	0.00857	6.061	0.02023
metab_227	neg_6319	neg	55.4	6.072	0.02598	5.283	0.03328
metab_121	pos_12335	pos	51.8	4.864	0.0203	4.07	0.09543
metab_544	pos_5583	pos	53.3	4.636	0.1049	5.428	0.04636
metab_180	neg_1627	neg	48.6	4.595	0.191	5.41	0.019
metab_227	neg_6410	neg	47.6	6.648	0.007293	5.869	0.01665
metab_151	pos_1584	pos	53	4.977	0.1159	5.769	0.02825
metab_128	pos_13054	pos	46.4	4.949	0.03074	4.166	0.06593
metab_229	neg_6519	neg	54	5.976	0.03944	6.753	0.009139
metab_169	neg_466	neg	46.1	5.986	0.03832	5.178	0.1897
metab_143	pos_14600	pos	51.2	5.865	0.009623	6.637	0.009122
metab_613	pos_6282	pos	50.5	3.792	0.02865	4.564	0.02832
metab_171	neg_662	neg	58.2	6.564	0.0137	7.332	0.00375
metab_128	pos_1335	pos	54.7	5.824	0.02836	6.592	0.013
metab_225	neg_6206	neg	52.4	5.472	0.0443	6.239	0.0106
metab_960	pos_9771	pos	48	5.704	0.0797	6.478	0.0444
metab_344	pos_3550	pos	56.8	4.675	0.04892	5.441	0.007216
metab_231	neg_6791	neg	55.3	6.178	0.02938	5.41	0.05673
metab_170	neg_563	neg	56.6	7.195	0.004725	6.433	0.007631

metab_551	pos_565	pos	46.6	5.224	0.03661	5.984	0.01737
metab_179	neg_1469	neg	45.3	6.489	0.01271	5.728	0.03293
metab_133	pos_13532	pos	48.1	5.537	0.03576	6.298	0.02269
metab_130	pos_13232	pos	46.2	6.318	0.01198	5.561	0.01989
metab_193	neg_2935	neg	58.2	7.158	0.008655	6.401	0.01172
metab_123	pos_12601	pos	50.5	4.493	0.1036	5.262	0.03779
metab_106	pos_1102	pos	57	5.801	0.01005	5.04	0.06441
metab_631	pos_6469	pos	48.5	4.776	0.03279	4.011	0.08537
metab_171	neg_624	neg	45.7	5.022	0.01041	5.781	0.06029
metab_190	neg_2626	neg	51.5	4.924	0.1047	5.691	0.0215
metab_703	pos_722	pos	47.6	6.101	0.03484	6.857	0.03738
metab_212	neg_4820	neg	45.1	5.217	0.06512	4.451	0.08884
metab_156	pos_15939	pos	43.5	5.445	0.01796	6.2	0.04398
metab_234	neg_7041	neg	52.7	5.89	0.02977	5.137	0.03916
metab_140	pos_1472	pos	48.9	5.495	0.09322	6.254	0.0297
metab_273	pos_2831	pos	55.6	4.847	0.06801	5.6	0.01638
metab_167	neg_248	neg	57.7	6.941	0.04319	6.191	0.02621
metab_156	pos_15904	pos	51.5	6.196	0.02277	6.946	0.03344
metab_229	neg_6564	neg	46.3	5.968	0.02649	6.715	0.01923
metab_731	pos_7468	pos	46.9	6.087	0.1259	6.856	0.07989
metab_126	pos_1309	pos	48.3	7.517	0.01509	6.77	0.01486
metab_138	pos_14084	pos	54.6	5.333	0.01406	6.079	0.01046
metab_489	pos_502	pos	53.2	5.597	0.02554	6.343	0.02153
metab_174	neg_936	neg	47.1	5.706	0.0382	6.452	0.01825
metab_124	pos_12627	pos	53.1	4.935	0.02931	4.185	0.0679
metab_232	neg_6862	neg	54.8	5.011	0.1197	4.239	0.1154
metab_224	neg_6063	neg	50.8	6.025	0.03151	6.77	0.0219
metab_208	neg_4470	neg	56.5	5.06	0.331	5.894	0.03335
metab_423	pos_4347	pos	52.6	5.7	0.0488	6.446	0.04396
metab_135	pos_13742	pos	46.5	6.183	0.02165	5.44	0.03782
metab_176	neg_1147	neg	49.6	5.48	0.01945	4.737	0.04011
metab_219	neg_5593	neg	53	6.062	0.0142	6.802	0.005653
metab_139	pos_14198	pos	55.6	5.272	0.01858	6.012	0.0121
metab_222	neg_5881	neg	48.8	5.03	0.1611	5.796	0.01294
metab_448	pos_4603	pos	45	5.699	0.05901	6.441	0.03274
metab_413	pos_4243	pos	45.8	4.619	0.04742	5.359	0.0217
metab_125	pos_12813	pos	46.6	5.172	0.01828	5.908	0.02958
metab_961	pos_9780	pos	47.8	4.532	0.1022	5.282	0.06813

metab_557	pos_5718	pos	43.2	4.843	0.364	5.68	0.01572
metab_134	pos_13635	pos	49.3	4.786	0.04284	5.518	0.01207
metab_128	pos_13078	pos	47	4.945	0.03603	5.678	0.02727
metab_216	neg_5212	neg	46.6	5.547	0.02612	4.812	0.06084
metab_188	neg_2427	neg	47.6	6.74	0.0311	6.009	0.01346
metab_175	neg_1095	neg	44	5.815	0.02416	5.083	0.05133
metab_197	neg_3343	neg	46.4	5.724	0.04773	4.993	0.04279
metab_586	pos_602	pos	50.2	6.857	0.01651	6.129	0.01079
metab_184	neg_2043	neg	45.3	5.068	0.06018	4.324	0.1145
metab_182	neg_1807	neg	52.2	4.784	0.09294	5.52	0.01744
metab_161	pos_1683	pos	56.6	5.256	0.01211	5.981	0.01281
metab_188	neg_2434	neg	50.1	5.167	0.0461	4.431	0.09563
metab_137	pos_1430	pos	54	5.934	0.01193	5.208	0.03862
metab_213	neg_4976	neg	42.8	5.568	0.06078	4.829	0.09811
metab_402	pos_4134	pos	44.1	5.529	0.03543	6.253	0.02942
metab_161	pos_1687	pos	53.6	5.424	0.03239	6.146	0.01128
metab_218	neg_5415	neg	46.6	5.798	0.02724	6.52	0.01172
metab_232	neg_6895	neg	45.7	6.694	0.1279	7.436	0.06843
metab_207	neg_4349	neg	47.2	5.169	0.09911	4.412	0.1642
metab_140	pos_14286	pos	56.3	6.183	0.01776	6.902	0.01582
metab_173	neg_843	neg	57.7	5.595	0.04635	6.316	0.009468
metab_176	pos_183	pos	41.2	4.744	0.194	3.944	0.2421
metab_172	neg_776	neg	48.9	6.923	0.007401	6.207	0.01079
metab_211	pos_2190	pos	45.1	5.507	0.04942	6.226	0.03033
metab_170	neg_560	neg	46	5.12	0.1512	4.38	0.004901
metab_146	pos_14932	pos	46.9	5.66	0.02592	4.944	0.04886
metab_182	neg_1834	neg	49.7	5.438	0.0231	4.718	0.07379
metab_112	pos_11455	pos	46.2	6.608	0.01649	7.32	0.01132
metab_122	pos_12476	pos	56	6.103	0.02558	5.394	0.01218
metab_127	pos_12997	pos	50.6	5.009	0.01812	4.298	0.0459
metab_212	neg_4878	neg	57.5	6.317	0.006769	5.61	0.01062
metab_228	neg_6462	neg	50.6	6.655	0.01335	7.362	0.0157
metab_230	neg_6629	neg	49.7	5.684	0.01772	4.977	0.02006
metab_204	neg_4009	neg	50.8	5.368	0.03597	4.659	0.0273
metab_168	neg_351	neg	51.3	4.733	0.09749	5.447	0.01515
metab_198	neg_3381	neg	52.9	5.364	0.04168	4.653	0.06518
metab_178	neg_1407	neg	52.2	5.943	0.03807	6.647	0.007258
metab_224	neg_6031	neg	52.7	5.792	0.01835	6.493	0.01541

metab_396	pos_4076	pos	45.6	5.553	0.04928	6.256	0.01462
metab_391	pos_4027	pos	50.2	5.563	0.01795	4.863	0.0322
metab_198	neg_3394	neg	45	5.637	0.03403	4.937	0.03274
metab_538	pos_5524	pos	45	5.126	0.03534	4.42	0.08863
metab_211	neg_4756	neg	51.2	4.878	0.1552	4.151	0.08117
metab_111	pos_11383	pos	48.7	5.739	0.02529	6.434	0.01466
metab_176	neg_1194	neg	56.8	5.579	0.006086	6.272	0.01143
metab_120	pos_12265	pos	43.8	5.202	0.03623	5.896	0.009816
metab_180	neg_1569	neg	47	5.57	0.03147	4.875	0.04267
metab_226	neg_6229	neg	51.5	4.97	0.1398	5.681	0.0275
metab_198	neg_3467	neg	43.7	4.887	0.07208	4.181	0.1007
metab_222	neg_5835	neg	45.2	5.452	0.0251	6.141	0.01112
metab_219	pos_2276	pos	51.4	5.38	0.05147	4.686	0.04722
metab_318	pos_3291	pos	46.5	6.205	0.01288	6.893	0.01919
metab_825	pos_851	pos	47.2	5.434	0.03269	4.744	0.07108
metab_219	neg_5503	neg	54.5	5.287	0.1019	5.982	0.01266
metab_184	neg_1979	neg	48.5	6.023	0.009441	5.34	0.02386
metab_171	neg_681	neg	53.7	5.869	0.01369	6.548	0.01501
metab_163	pos_1701	pos	46.1	4.978	0.01302	4.302	0.04224
metab_149	pos_15164	pos	44.1	5.554	0.07929	6.235	0.0177
metab_151	pos_1580	pos	42.3	5.648	0.02759	4.974	0.02862
metab_170	neg_590	neg	49	5.216	0.3696	6.002	0.04704
metab_294	pos_3048	pos	46.9	4.858	0.01702	5.529	0.01678
metab_457	pos_4694	pos	47.1	5.194	0.0377	5.866	0.01717
metab_208	neg_4429	neg	47.8	4.498	0.01041	5.164	0.02459
metab_818	pos_8351	pos	49.7	5.393	0.1139	6.077	0.0565
metab_525	pos_5397	pos	47.5	4.473	0.004974	5.139	0.02716
metab_219	neg_5544	neg	45.9	5.148	0.08561	5.822	0.03366
metab_130	pos_13284	pos	55.7	5.623	0.02941	4.956	0.03678
metab_494	pos_5076	pos	53	4.899	0.04073	5.565	0.01044
metab_216	neg_5261	neg	48.8	5.027	0.05149	5.696	0.03683
metab_230	neg_6619	neg	48.1	5.448	0.0329	4.784	0.02677
metab_208	neg_4475	neg	50.2	4.362	0.3018	5.111	0.02433
metab_122	pos_12441	pos	52.3	5.345	0.03114	6.004	0.008075
metab_155	pos_15883	pos	47.8	5.086	0.07472	5.753	0.03814
metab_609	pos_6240	pos	47.5	4.179	0.05281	4.84	0.04092
metab_198	neg_3457	neg	44.9	6.12	0.01239	6.775	0.009574
metab_189	neg_2513	neg	49.5	5.192	0.02896	4.533	0.05296

metab_281	pos_2910	pos	56.2	5.279	0.02388	5.933	0.01467
metab_188	neg_2412	neg	45.9	5.233	0.09623	5.897	0.00949
metab_115	pos_1196	pos	47.5	7.435	0.009493	6.783	0.01772
metab_310	pos_3210	pos	50	5.084	0.01165	5.73	0.01036
metab_167	neg_290	neg	55.3	5.245	0.03879	5.892	0.01386
metab_227	neg_6320	neg	49.4	5.591	0.0257	4.942	0.05155
metab_181	neg_1731	neg	45.6	5.348	0.06923	4.695	0.05543
metab_970	pos_9868	pos	43.8	3.901	0.004974	4.544	0.03634
metab_170	neg_542	neg	43.9	5.753	0.04061	6.395	0.02207
metab_234	neg_7036	neg	47.7	4.596	0.06297	5.241	0.02447
metab_719	pos_738	pos	52.2	5.137	0.04016	5.78	0.04403
metab_117	pos_11921	pos	57.8	5.675	0.02424	6.313	0.01766
metab_326	pos_3367	pos	48	5.385	0.02706	4.746	0.02375
metab_232	pos_2414	pos	57.6	5.748	0.0134	5.11	0.02929
metab_156	pos_15902	pos	47.1	5.539	0.02493	6.178	0.0322
metab_376	pos_3871	pos	56.4	5.938	0.01839	6.575	0.01204
metab_198	neg_3426	neg	51.3	5.155	0.01611	4.516	0.03649
metab_150	pos_15288	pos	50.2	5.058	0.1492	5.722	0.05137
metab_146	pos_1531	pos	51.8	4.729	0.2037	5.41	0.03937
metab_175	neg_1085	neg	47	6.118	0.02172	6.752	0.01537
metab_204	neg_3984	neg	46.3	5.758	0.02213	5.126	0.02094
metab_133	pos_13584	pos	47.1	5.004	0.0286	5.637	0.01634
metab_174	neg_986	neg	51.7	5.995	0.02583	6.627	0.01008
metab_171	neg_631	neg	46.1	4.604	0.03556	5.237	0.01995
metab_228	neg_6443	neg	47.5	5.611	0.01603	6.243	0.02375
metab_213	neg_4916	neg	53.4	5.912	0.02506	5.279	0.03576
metab_190	neg_2609	neg	52	4.91	0.2348	5.6	0.02103
metab_101	pos_10346	pos	54.5	3.614	0.1118	4.263	0.05386
metab_102	pos_10385	pos	49.8	5.023	0.01971	5.655	0.03998
metab_128	pos_13076	pos	45.4	4.815	0.03227	5.445	0.01758
metab_196	neg_3183	neg	53.6	5.459	0.03336	4.829	0.03065
metab_198	neg_3454	neg	49.9	6.104	0.02187	5.476	0.02271
metab_401	pos_4126	pos	53.1	5.018	0.01815	5.646	0.0284
metab_432	pos_4437	pos	43.9	5.763	0.03263	5.128	0.08064
metab_189	neg_2497	neg	51.2	5.212	0.04304	4.583	0.03414
metab_203	pos_211	pos	51.7	3.771	0.3292	4.495	0.04468
metab_229	neg_6517	neg	48.9	5.393	0.05729	4.764	0.04154
metab_210	pos_2186	pos	54.2	5.683	0.02171	6.306	0.01966

metab_224	neg_6036	neg	46.8		5.461	0.04625	4.837	0.03565
metab_208	neg_4474	neg	47.1		5.528	0.02835	4.907	0.02316
metab_205	neg_4075	neg	47.8		5.403	0.08777	6.035	0.04688
metab_155	pos_15832	pos	52.6		5.77	0.01423	6.39	0.02341
metab_154	pos_15728	pos	51.6		6.506	0.007806	7.123	0.00633
metab_205	neg_4089	neg	47.6		5.265	0.0228	5.881	0.01285
metab_182	neg_1764	neg	57.1		5.687	0.02795	5.071	0.02713
metab_231	neg_6815	neg	48.1		4.773	0.3947	5.516	0.026
metab_124	pos_12686	pos	49.6		4.646	0.02366	5.26	0.01818
metab_153	pos_15678	pos	50.6		6.985	0.009549	7.597	0.02998
metab_475	pos_4878	pos	52.6		4.912	0.04925	5.524	0.009618
metab_137	pos_14020	pos	50.6		5.756	0.01461	5.148	0.01164
metab_127	pos_12989	pos	51.8		4.79	0.07704	5.406	0.01128
metab_120	pos_12284	pos	50.1		5.241	0.01205	4.626	0.07508
metab_158	pos_16168	pos	48.6		5.492	0.01463	6.1	0.03065
metab_208	neg_4467	neg	53.8		5.232	0.05361	5.842	0.01514
metab_994	pos_10114	pos	54.1		4.523	0.09188	5.142	0.04548
metab_148	pos_15097	pos	47.8		5.497	0.02015	6.102	0.01548
metab_544	pos_5585	pos	44.5		4.661	0.004974	5.267	0.04135
metab_705	pos_724	pos	49.8		6.922	0.0114	7.526	0.02002
metab_215	neg_5089	neg	56.3		5.388	0.04204	5.993	0.01017
metab_937	pos_969	pos	50.8		7.071	0.01088	7.674	0.02905
metab_616	pos_6312	pos	47.6		7.038	0.01567	7.637	0.03339
metab_142	pos_1487	pos	43.9		5.74	0.01932	5.139	0.05588
metab_537	pos_5509	pos	51.4		4.508	0.09746	5.117	0.02064
metab_647	pos_6630	pos	45.1		4.577	0.04391	5.175	0.01527
metab_180	neg_1611	neg	47.1		5.243	0.05522	4.642	0.04717
metab_139	pos_14219	pos	50.7		5.321	0.01675	5.914	0.01143
metab_149	pos_15218	pos	53.7		5.894	0.02359	6.488	0.01542
metab_156	pos_15906	pos	50.5		6.551	0.008314	7.144	0.02276
metab_167	neg_237	neg	50.1		5.423	0.1735	4.763	0.1895
metab_278	pos_2881	pos	55		5.173	0.04045	5.768	0.03528
metab_394	pos_4051	pos	51.1		5.568	0.02026	4.977	0.02787
metab_181	neg_1665	neg	49.1		6.301	0.02757	5.712	0.01351
metab_221	neg_5758	neg	47.5		5.349	0.05022	4.757	0.004901
metab_213	neg_4965	neg	50.5		5.874	0.01747	5.285	0.02886
metab_156	pos_15908	pos	53.5		6.047	0.01998	6.636	0.02951
metab_180	pos_1875	pos	55.9		6.241	0.02589	5.653	0.0214

metab_228	neg_6500	neg	49	5.36	0.01041	5.947	0.03492
metab_231	neg_6759	neg	42.5	5.28	0.01041	5.868	0.05439
metab_195	neg_3141	neg	48.5	5.297	0.01635	4.709	0.05142
metab_223	neg_5977	neg	54.8	5.474	0.02258	6.057	0.007922
metab_210	neg_4637	neg	48.4	5.788	0.04108	6.373	0.01623
metab_440	pos_4518	pos	52.2	5.356	0.1595	5.97	0.0325
metab_106	pos_10799	pos	45	4.65	0.004974	5.233	0.04696
metab_149	pos_15233	pos	45.5	5.083	0.1254	5.684	0.04055
metab_222	neg_5806	neg	46.2	5.499	0.06301	6.083	0.01628
metab_179	neg_1473	neg	55.1	4.966	0.1625	5.576	0.01772
metab_115	pos_11709	pos	48.7	4.741	0.08306	5.328	0.02359
metab_201	neg_3740	neg	46.1	5.498	0.05783	4.914	0.04705
metab_120	pos_12277	pos	52.6	5.129	0.03255	5.706	0.01641
metab_153	pos_15671	pos	55.4	6.993	0.005602	6.419	0.01172
metab_210	neg_4666	neg	50.4	6.445	0.01248	5.871	0.02278
metab_128	pos_13053	pos	52.1	4.869	0.03859	5.444	0.01635
metab_150	pos_15274	pos	54.1	6.086	0.04572	6.662	0.00731
metab_189	neg_2520	neg	48.8	6.427	0.03413	5.851	0.02942
metab_218	neg_5406	neg	49.3	6.445	0.02016	7.018	0.01055
metab_234	neg_7134	neg	47.9	5.705	0.01718	5.132	0.02665
metab_219	neg_5496	neg	52.5	4.759	0.194	5.378	0.03591
metab_205	neg_4072	neg	46.8	5.353	0.02858	4.78	0.01414
metab_173	neg_931	neg	54.3	4.614	0.01041	5.185	0.02776
metab_235	neg_7154	neg	44	4.611	0.01041	5.181	0.02777
metab_212	neg_4834	neg	49.4	5.185	0.04184	4.617	0.01721
metab_188	neg_2396	neg	48.8	5.503	0.03675	4.936	0.02865
metab_474	pos_486	pos	43.8	5.347	0.0229	5.91	0.01891
metab_366	pos_3771	pos	51.8	5.42	0.04403	5.986	0.03627
metab_203	neg_3964	neg	44.9	4.207	0.1824	4.811	0.05804
metab_223	neg_5967	neg	51	6.654	0.02085	6.095	0.01044
metab_502	pos_5152	pos	45.6	5.972	0.01841	6.53	0.008409
metab_149	pos_15187	pos	50.6	5.175	0.1013	5.744	0.03414
metab_223	neg_5997	neg	46.7	5.602	0.00772	5.047	0.03511
metab_119	pos_12187	pos	54.6	4.969	0.03281	5.524	0.03026
metab_234	neg_7113	neg	52.9	4.866	0.06511	5.426	0.02903
metab_116	pos_11863	pos	42.7	5.893	0.06188	6.454	0.04891
metab_178	neg_1370	neg	56.3	5.403	0.02404	4.85	0.03623
metab_182	neg_1787	neg	43.5	5.745	0.02086	6.297	0.02053

metab_122	pos_12458	pos	46.9	5.058	0.02419	5.61	0.00515
metab_216	neg_5284	neg	44.3	4.998	0.06772	4.44	0.004901
metab_150	pos_15346	pos	51.6	6.854	0.02166	6.304	0.01089
metab_211	neg_4763	neg	55.9	6.063	0.02611	6.613	0.01418
metab_468	pos_4802	pos	54.3	4.671	0.07354	5.23	0.03955
metab_230	neg_6680	neg	52.6	6.159	0.01008	5.61	0.01895
metab_214	neg_5077	neg	48.1	5.994	0.0264	5.446	0.007898
metab_154	pos_15691	pos	49	5.693	0.01173	5.144	0.03097
metab_115	pos_11757	pos	43.6	5.321	0.05644	5.876	0.04876
metab_209	neg_4499	neg	54.1	6.291	0.01583	5.744	0.01329
metab_932	pos_964	pos	54.8	6.634	0.09303	7.195	0.03178
metab_180	neg_1637	neg	49.4	4.991	0.1349	5.562	0.02416
metab_215	neg_5108	neg	45.5	5.74	0.03	5.191	0.03398
metab_188	neg_2351	neg	51.6	7.156	0.02255	6.61	0.01462
metab_171	neg_641	neg	57.6	6.553	0.006994	7.097	0.01962
metab_116	pos_11851	pos	48.3	4.52	0.07892	5.073	0.03021
metab_216	neg_5256	neg	55.4	5.641	0.03023	5.096	0.02825
metab_194	neg_2988	neg	50.7	6.663	0.01992	7.206	0.01128
metab_211	neg_4764	neg	56.2	5.776	0.03782	6.32	0.0147
metab_117	pos_11932	pos	45.2	5.599	0.01791	6.142	0.01577
metab_194	neg_2997	neg	52.2	5.506	0.0419	6.051	0.01033
metab_217	neg_5316	neg	55.5	5.355	0.06519	5.902	0.00369
metab_178	neg_1429	neg	47.1	5.843	0.01338	5.301	0.01712
metab_125	pos_12777	pos	46.2	5.756	0.04655	6.3	0.02529
metab_171	neg_715	neg	57	6.468	0.008047	7.008	0.01197
metab_212	neg_4836	neg	50	6.695	0.04225	6.153	0.02064
metab_496	pos_509	pos	45.9	4.958	0.0742	5.508	0.05509
metab_100	pos_10204	pos	51.8	4.833	0.03719	5.374	0.03221
metab_108	pos_10981	pos	41.6	5.641	0.06167	6.187	0.03612
metab_115	pos_11787	pos	54.1	5.481	0.02479	6.019	0.01807
metab_122	pos_12492	pos	48	5.195	0.01316	5.731	0.0162
metab_205	neg_4096	neg	55.6	5.649	0.02232	5.107	0.06411
metab_196	neg_3202	neg	52.5	5.84	0.02658	5.299	0.05943
metab_174	neg_947	neg	50.4	5.428	0.05453	5.967	0.01541
metab_192	neg_2801	neg	43.9	5.693	0.02983	5.158	0.01681
metab_211	neg_4768	neg	50.4	6.66	0.01219	6.126	0.02441
metab_212	neg_4813	neg	48.9	5.421	0.02741	5.96	0.06274
metab_135	pos_13808	pos	50.5	5.895	0.02878	6.43	0.02668

metab_183	neg_1886	neg	55.1	6.152	0.02955	6.687	0.03356
metab_208	neg_4472	neg	52.8	4.997	0.04362	4.462	0.0255
metab_230	neg_6626	neg	52.6	5.691	0.01031	5.16	0.01739
metab_206	neg_4241	neg	44.4	5.224	0.01763	4.692	0.03094
metab_155	pos_15794	pos	58	7.066	0.017	6.534	0.0321
metab_124	pos_1285	pos	47.1	5	0.01531	4.467	0.04816
metab_230	neg_6699	neg	56	5.536	0.0213	5.004	0.03079
metab_190	neg_2584	neg	45.2	5.416	0.07362	4.875	0.04926
metab_313	pos_3241	pos	47.4	4.816	0.02173	4.28	0.06477
metab_185	neg_2113	neg	48	5.591	0.07244	5.052	0.04786
metab_221	neg_5709	neg	53.8	6.594	0.01093	6.066	0.009969
metab_169	neg_469	neg	56.3	6.565	0.01139	6.037	0.01289
metab_578	pos_5931	pos	45.5	5.506	0.06065	6.039	0.02668
metab_186	neg_2193	neg	46.3	5.803	0.02123	5.275	0.03454
metab_380	pos_3916	pos	48.8	4.712	0.05543	5.242	0.01601
metab_138	pos_14040	pos	50.4	5.272	0.0168	4.747	0.01685
metab_213	neg_4944	neg	56.1	5.803	0.02414	6.328	0.009079
metab_134	pos_13656	pos	43.6	4.783	0.02481	5.307	0.0246
metab_226	neg_6247	neg	51.4	5.663	0.02907	6.188	0.02442
metab_461	pos_4735	pos	49	4.988	0.1402	5.54	0.03948
metab_231	neg_6789	neg	57.1	6.192	0.01411	6.714	0.0147
metab_149	pos_15210	pos	52.5	5.176	0.004974	5.697	0.01496
metab_231	neg_6779	neg	57.7	6.559	0.04084	7.083	0.01921
metab_175	neg_1120	neg	48.5	5.466	0.08992	6	0.04354
metab_986	pos_10032	pos	55.8	5.029	0.03133	4.508	0.02364
metab_214	neg_4996	neg	45.6	5.497	0.02274	4.978	0.01785
metab_194	neg_2994	neg	45.3	4.866	0.2542	5.457	0.02077
metab_214	neg_5061	neg	53.1	5.155	0.01041	5.672	0.0122
metab_238	pos_2466	pos	46	5.236	0.01882	5.754	0.02347
metab_443	pos_4544	pos	48.6	4.656	0.004974	5.174	0.04622
metab_111	pos_11308	pos	55.5	5.607	0.04659	6.125	0.01144
metab_130	pos_13252	pos	50.2	5.051	0.02621	5.568	0.02157
metab_176	neg_1233	neg	48.9	6.319	0.01405	6.834	0.01103
metab_112	pos_1164	pos	45.6	5.875	0.01364	5.361	0.02772
metab_212	neg_4884	neg	47.2	5.782	0.03749	6.296	0.03131
metab_115	pos_1195	pos	45.9	5.54	0.01049	5.029	0.02278
metab_222	neg_5824	neg	51.7	6.44	0.01691	5.93	0.0135
metab_208	neg_4437	neg	48.7	6.193	0.01709	5.683	0.01357

metab_188	neg_2444	neg	43.2	5.725	0.06337	5.202	0.07008
metab_207	neg_4328	neg	50.8	6.005	0.02696	5.494	0.03337
metab_209	neg_4551	neg	51.3	4.76	0.2886	5.36	0.0384
metab_234	neg_7072	neg	46	5.933	0.02583	5.423	0.03516
metab_154	pos_1607	pos	49.7	5.639	0.01617	5.122	0.07611
metab_124	pos_12655	pos	50.3	5.079	0.113	5.607	0.0414
metab_193	neg_2939	neg	53.9	4.997	0.07529	5.513	0.01747
metab_156	pos_15903	pos	55.6	6.616	0.01394	7.122	0.01907
metab_175	neg_1082	neg	53.9	5.731	0.01708	6.238	0.02009
metab_117	pos_11906	pos	56	4.865	0.0363	5.373	0.01525
metab_388	pos_3996	pos	54.6	5.718	0.02568	6.225	0.02642
metab_217	neg_5372	neg	54.5	5.282	0.03717	5.788	0.01205
metab_189	neg_2466	neg	54.2	4.988	0.03614	4.482	0.02452
metab_184	neg_1980	neg	52.8	5.135	0.04853	5.643	0.03534
metab_227	neg_6330	neg	46.5	6.349	0.01743	6.853	0.01176
metab_183	neg_1847	neg	48.3	6.028	0.01118	5.526	0.02018
metab_180	neg_1549	neg	46.6	6.176	0.01128	5.674	0.01795
metab_187	neg_2257	neg	50	6.226	0.02364	6.726	0.005418
metab_470	pos_4830	pos	49.4	6.073	0.02364	6.575	0.03227
metab_468	pos_4804	pos	56.9	6.656	0.01816	7.155	0.007425
metab_693	pos_7087	pos	46.8	5.184	0.1698	4.612	0.1864
metab_206	neg_4208	neg	48.1	6.281	0.006559	5.783	0.01388
metab_171	neg_663	neg	47.5	5.352	0.1569	5.884	0.01325
metab_208	neg_4450	neg	52.5	6.026	0.04871	5.521	0.05838
metab_151	pos_15407	pos	45.6	5.18	0.061	4.675	0.04124
metab_119	pos_12119	pos	41.5	5.234	0.05495	5.737	0.02668
metab_192	neg_2772	neg	44.1	5.725	0.04155	5.225	0.004901
metab_548	pos_5625	pos	43.9	5.246	0.07162	5.75	0.01457
metab_134	pos_13632	pos	46	4.83	0.1066	5.346	0.05437
metab_408	pos_4191	pos	46.3	4.947	0.02442	4.451	0.02684
metab_108	pos_1124	pos	53.4	6.689	0.01592	7.184	0.0194
metab_108	pos_1125	pos	53.5	6.944	0.01424	7.437	0.01592
metab_152	pos_15477	pos	51.7	6.559	0.008484	6.066	0.00806
metab_185	neg_2080	neg	48.4	5.97	0.0183	5.477	0.01911
metab_186	neg_2235	neg	47.9	5.006	0.07379	5.508	0.02317
metab_214	neg_5039	neg	48.7	5.204	0.1062	5.713	0.01446
metab_149	pos_15243	pos	54.1	5.316	0.04795	5.811	0.009152
metab_137	pos_13932	pos	48.1	6.089	0.01622	5.596	0.03433

metab_136	pos_13925	pos	52.2	5.306	0.02572	5.798	0.0157
metab_181	neg_1699	neg	50.2	5.892	0.03961	5.399	0.01668
metab_104	pos_10605	pos	46	5.976	0.1287	6.491	0.03595
metab_171	neg_694	neg	54.3	5.844	0.02895	6.335	0.01062
metab_737	pos_7530	pos	42.1	5.47	0.06579	4.948	0.1373
metab_170	neg_551	neg	56.1	5.578	0.03992	6.07	0.007173
metab_214	neg_5062	neg	42.2	6.115	0.03382	5.624	0.02636
metab_112	pos_11489	pos	44.3	6.022	0.03459	6.512	0.01762
metab_172	neg_786	neg	49.3	5.54	0.06734	5.04	0.04749
metab_216	neg_5216	neg	42.4	5.451	0.05751	4.956	0.03261
metab_122	pos_12499	pos	47.6	5.607	0.01315	5.115	0.05243
metab_208	neg_4441	neg	43.3	5.791	0.0478	5.298	0.02354
metab_140	pos_14319	pos	51.4	5.022	0.05194	5.515	0.02372
metab_126	pos_12828	pos	45.5	5.214	0.02974	5.703	0.0132
metab_205	neg_4105	neg	48.9	4.543	0.07004	5.038	0.02468
metab_226	neg_6308	neg	51.7	5.249	0.1611	5.772	0.01497
metab_224	neg_6024	neg	51	5.306	0.03769	4.813	0.05061
metab_139	pos_1461	pos	49.3	5.578	0.0145	5.089	0.03959
metab_185	neg_2062	neg	51	5.376	0.03752	4.885	0.04093
metab_193	neg_2919	neg	51	4.82	0.04624	4.33	0.03627
metab_150	pos_15281	pos	45.5	5.605	0.06474	5.088	0.131
metab_196	neg_3206	neg	49.5	5.067	0.05211	5.558	0.03269
metab_925	pos_957	pos	50.8	6.434	0.01269	5.95	0.01231
metab_225	neg_6164	neg	54.6	6.044	0.01757	5.56	0.009152
metab_113	pos_11499	pos	48.9	6.065	0.03191	6.551	0.01889
metab_641	pos_6565	pos	44.7	5.058	0.05049	5.546	0.01912
metab_155	pos_15798	pos	49.5	5.812	0.0241	5.328	0.02159
metab_150	pos_15310	pos	47.7	4.604	0.05871	5.093	0.03752
metab_135	pos_13764	pos	49.9	4.838	0.02687	5.321	0.02721
metab_176	neg_1205	neg	44.7	6.158	0.02004	5.675	0.02391
metab_198	neg_3382	neg	50.3	5.648	0.01339	5.166	0.0299
metab_193	neg_2909	neg	48	6.059	0.05359	5.574	0.02315
metab_201	neg_3725	neg	51.9	5.722	0.3369	6.317	0.04971
metab_217	neg_5334	neg	45.2	5.359	0.05564	5.842	0.01178
metab_150	pos_15276	pos	50.9	5.699	0.03772	6.178	0.009926
metab_144	pos_14713	pos	53.1	5.566	0.01103	6.044	0.02468
metab_211	neg_4725	neg	49.5	5.341	0.0973	5.833	0.02037
metab_223	neg_5993	neg	45.1	5.194	0.07901	5.68	0.03022

metab_462	pos_4740	pos	47.7	5.326	0.07798	5.81	0.01383
metab_231	neg_6736	neg	45.3	6.288	0.02885	6.762	0.008309
metab_194	neg_3037	neg	52.5	5.741	0.06313	5.263	0.02247
metab_213	neg_4935	neg	49.1	5.433	0.03197	4.961	0.03077
metab_819	pos_8354	pos	48.9	4.714	0.05853	5.194	0.061
metab_137	pos_13933	pos	55.4	5.527	0.02703	5.055	0.04437
metab_177	neg_1312	neg	47.8	5.648	0.03659	5.177	0.02911
metab_231	neg_6728	neg	44.3	5.147	0.07146	5.622	0.02085
metab_229	neg_6582	neg	48	5.286	0.07735	5.763	0.01149
metab_125	pos_12796	pos	55	4.772	0.03162	5.24	0.02136
metab_196	neg_3243	neg	57	6.956	0.01765	6.489	0.02126
metab_199	neg_3512	neg	55.7	6.903	0.02243	6.436	0.01708
metab_116	pos_11819	pos	47.1	5.06	0.06011	5.535	0.03512
metab_440	pos_4512	pos	51.3	5.014	0.0235	5.481	0.02017
metab_407	pos_4186	pos	56.7	4.904	0.02423	5.371	0.02125
metab_122	pos_12474	pos	56.1	5.944	0.01855	6.409	0.006576
metab_203	neg_3959	neg	46.4	5.89	0.03133	5.425	0.02747
metab_185	neg_2127	neg	47.6	5.665	0.04158	6.131	0.01538
metab_224	neg_6057	neg	45	6.057	0.03112	5.593	0.02749
metab_225	pos_2342	pos	54	7.168	0.008482	7.632	0.03275
metab_299	pos_3097	pos	45.3	4.948	0.03696	5.413	0.02112
metab_103	pos_10511	pos	46.8	4.965	0.01751	4.501	0.03803
metab_176	neg_1225	neg	54	6.402	0.009609	5.94	0.01456
metab_204	neg_4031	neg	53.2	6.62	0.01717	6.158	0.0176
metab_266	pos_2756	pos	50.3	5.612	0.01652	6.073	0.005726
metab_400	pos_4109	pos	51.9	4.816	0.04282	5.28	0.01025
metab_423	pos_4343	pos	46.6	4.789	0.06144	5.258	0.03341
metab_218	neg_5475	neg	48.7	6.046	0.02328	5.585	0.02154
metab_179	pos_1871	pos	49.1	5.753	0.02172	5.292	0.02506
metab_213	neg_4946	neg	53	5.046	0.06658	5.514	0.02586
metab_178	pos_1861	pos	49.8	6.096	0.01423	6.556	0.0157
metab_957	pos_9741	pos	49.7	5.509	0.03648	5.972	0.03569
metab_123	pos_12525	pos	51.7	5.034	0.02959	5.495	0.02567
metab_150	pos_15278	pos	54.5	4.981	0.04153	5.442	0.01601
metab_205	neg_4163	neg	51.3	5.936	0.0201	5.474	0.04279
metab_170	neg_541	neg	52.3	5.482	0.1137	5.961	0.01635
metab_204	neg_4018	neg	46.8	5.664	0.02812	5.204	0.01801
metab_189	neg_2518	neg	46.5	5.677	0.03431	6.137	0.007306

metab_22	neg_6035	neg	47.1	5.658	0.02306	5.198	0.03039
metab_21	neg_5220	neg	49.8	5.024	0.03059	4.56	0.05656
metab_19	neg_3142	neg	46.7	5.397	0.06605	4.93	0.03909
metab_28	pos_2997	pos	44.7	5.452	0.02923	5.912	0.02154
metab_64	pos_6648	pos	46.8	4.757	0.03846	5.217	0.0176
metab_17	neg_599	neg	47.8	7.196	0.008219	6.738	0.02629
metab_18	neg_2123	neg	50.3	5.607	0.05113	6.068	0.01106
metab_17	neg_920	neg	50.4	6.266	0.02366	6.723	0.02085
metab_16	neg_228	neg	46.5	5.275	0.01223	4.818	0.03183
metab_47	pos_4887	pos	48	4.861	0.03831	5.321	0.03346
metab_14	pos_1427	pos	50	5.261	0.09519	5.736	0.06378
metab_10	pos_10618	pos	49.8	5.895	0.03582	6.352	0.01903
metab_23	neg_6622	neg	52.2	5.569	0.02609	5.112	0.03647
metab_18	neg_1951	neg	52.7	5.513	0.08238	5.979	0.01781
metab_15	pos_15467	pos	48.1	6.606	0.009312	6.151	0.02337
metab_23	neg_6799	neg	49.7	5.107	0.01041	5.565	0.04551
metab_19	neg_3254	neg	48.3	6.758	0.01161	6.304	0.009404
metab_42	pos_4390	pos	47.3	5.072	0.03817	5.529	0.01746
metab_13	pos_1408	pos	44.1	5.697	0.04291	6.155	0.03316
metab_27	pos_2879	pos	53	4.948	0.01778	4.491	0.04135
metab_19	neg_3504	neg	43.9	4.752	0.01041	5.206	0.02033
metab_34	pos_3566	pos	54.5	5.222	0.03217	5.678	0.02608
metab_14	pos_14294	pos	52	7.108	0.007562	7.563	0.03768
metab_13	pos_1368	pos	56	5.902	0.02899	6.356	0.01472
metab_19	neg_2696	neg	56.2	5.343	0.07207	5.805	0.0232
metab_21	neg_4588	neg	47.8	6.396	0.01793	5.944	0.01302
metab_22	neg_6391	neg	47.8	5.592	0.02622	5.138	0.03264
metab_23	neg_6784	neg	51.4	5.712	0.05442	6.168	0.008151
metab_22	neg_6105	neg	47.4	5.868	0.01572	5.417	0.01523
metab_21	neg_5057	neg	48	5.894	0.02829	5.442	0.02162
metab_38	pos_3926	pos	47.7	5.239	0.02816	4.788	0.03001
metab_16	pos_1665	pos	46.5	7.778	0.02503	8.229	0.02837
metab_23	neg_7076	neg	45.4	4.984	0.03665	4.528	0.05819
metab_20	neg_4252	neg	47.8	4.813	0.1198	5.284	0.03053
metab_15	pos_1547	pos	52.5	6.537	0.0163	6.088	0.02642
metab_12	pos_12954	pos	45.2	4.896	0.02398	4.438	0.07639
metab_49	pos_5111	pos	47.9	5.172	0.01786	4.72	0.05503
metab_12	pos_1290	pos	51.2	5.523	0.02938	5.97	0.007368

metab_178	neg_1400	neg	56.9	5.702	0.01353	6.148	0.0216
metab_183	neg_1937	neg	54.5	6.354	0.01192	6.799	0.01723
metab_181	neg_1726	neg	51.1	5.819	0.02025	5.372	0.03888
metab_220	neg_5682	neg	56.9	6.052	0.01253	5.608	0.007644
metab_103	pos_10549	pos	57.7	6.45	0.01528	6.896	0.034
metab_217	neg_5347	neg	51	5.93	0.02169	5.486	0.01692
metab_216	neg_5268	neg	56.5	5.98	0.02611	6.424	0.01315
metab_175	neg_1098	neg	49.1	5.884	0.007706	5.442	0.00549
metab_211	neg_4737	neg	56.3	5.64	0.2395	6.155	0.01553
metab_995	pos_10123	pos	49.4	4.908	0.02833	5.352	0.03205
metab_190	neg_2580	neg	47.1	5.058	0.05148	4.604	0.06904
metab_118	pos_12053	pos	53	4.958	0.04211	5.403	0.01306
metab_378	pos_3893	pos	48.8	6.099	0.02687	6.541	0.011
metab_210	pos_219	pos	48.8	5.334	0.01411	5.777	0.03115
metab_394	pos_4054	pos	46	5.65	0.02769	6.091	0.0188
metab_167	neg_256	neg	48.8	5.732	0.0174	5.291	0.01565
metab_180	neg_1628	neg	45.9	6.326	0.02726	5.885	0.01578
metab_216	neg_5249	neg	45.5	5.474	0.01631	5.913	0.008716
metab_169	neg_500	neg	47.1	6.165	0.02693	5.722	0.03834
metab_633	pos_6484	pos	51.2	4.951	0.0124	5.391	0.01505
metab_212	neg_4853	neg	47.5	5.058	0.09289	4.603	0.0358
metab_223	neg_5922	neg	46.6	5.11	0.02044	5.549	0.02506
metab_203	neg_3940	neg	49.7	6.756	0.02607	6.317	0.01587
metab_122	pos_12427	pos	53.5	4.995	0.02722	5.432	0.02382
metab_945	pos_9619	pos	44.3	5.202	0.09945	5.668	0.098
metab_148	pos_15113	pos	45.9	5.579	0.02043	6.015	0.008822
metab_475	pos_487	pos	46	5.218	0.01814	5.652	0.02124
metab_125	pos_1300	pos	49.2	5.515	0.02167	5.075	0.05713
metab_203	neg_3949	neg	48.4	6.182	0.02872	5.749	0.01405
metab_178	neg_1412	neg	48.6	6.545	0.01109	6.112	0.01458
metab_168	neg_329	neg	58.4	6.597	0.01666	7.029	0.01747
metab_463	pos_4749	pos	51.5	5.645	0.02522	6.079	0.02077
metab_215	neg_5173	neg	48.2	5.128	0.04571	4.691	0.02982
metab_191	neg_2743	neg	47.9	5.917	0.07422	6.359	0.01783
metab_219	pos_2275	pos	46	6.083	0.03309	5.651	0.0319
metab_104	pos_10626	pos	43.2	4.708	0.05336	5.15	0.06899
metab_164	pos_16774	pos	47.4	6.904	0.03563	7.335	0.01141
metab_140	pos_14295	pos	51.1	5.951	0.02001	6.382	0.03902

metab_177	neg_1332	neg	46.1	5.496	0.01346	5.068	0.02123
metab_122	pos_12488	pos	44	5.477	0.0373	5.906	0.00706
metab_224	neg_6090	neg	54.5	6.68	0.01049	6.253	0.02606
metab_498	pos_5112	pos	55.8	5.103	0.04405	5.533	0.006365
metab_114	pos_11619	pos	53.4	5.746	0.01917	6.172	0.008142
metab_174	neg_1030	neg	45.6	5.636	0.04061	5.206	0.02816
metab_201	neg_3694	neg	45.9	5.166	0.1297	5.624	0.05968
metab_157	pos_16074	pos	47	5.698	0.01485	6.127	0.03762
metab_184	neg_2011	neg	48.3	5.466	0.0502	5.907	0.08307
metab_213	neg_4949	neg	55.8	5.581	0.03954	6.009	0.01532
metab_206	neg_4229	neg	52.5	5.827	0.02949	5.397	0.04632
metab_205	neg_4166	neg	51.5	6.68	0.02027	6.254	0.02375
metab_223	neg_5983	neg	42.3	6.207	0.008299	5.78	0.03709
metab_208	neg_4461	neg	46.4	5.623	0.01578	5.199	0.01483
metab_218	neg_5440	neg	44.7	5.28	0.04469	5.707	0.02258
metab_957	pos_9742	pos	58.3	6.288	0.00881	5.865	0.01792
metab_186	neg_2230	neg	52	5.951	0.01657	6.373	0.008453
metab_171	neg_678	neg	46.6	5.372	0.01041	5.794	0.01612
metab_173	neg_905	neg	47	5.482	0.05445	5.053	0.03664
metab_226	neg_6282	neg	55.1	6.388	0.01925	6.81	0.01334
metab_207	neg_4325	neg	49.7	6.344	0.008587	5.924	0.007381
metab_842	pos_8590	pos	49.6	5.245	0.05596	5.683	0.08598
metab_941	pos_9583	pos	45.5	5.373	0.04323	4.936	0.09101
metab_168	neg_349	neg	54.1	5.042	0.03554	5.464	0.0191
metab_100	pos_1043	pos	53.3	6.173	0.02099	5.752	0.03063
metab_208	neg_4395	neg	54.7	4.734	0.03684	4.31	0.03581
metab_215	neg_5098	neg	53.5	5.361	0.0386	5.782	0.009098
metab_189	neg_2470	neg	57.7	5.572	0.07085	5.14	0.04835
metab_234	neg_7071	neg	49.9	6.227	0.03359	5.806	0.02193
metab_584	pos_5994	pos	47.2	6.341	0.01229	6.764	0.05414
metab_231	neg_6744	neg	44.4	5.162	0.01041	5.582	0.03959
metab_224	neg_6039	neg	45.9	5.476	0.02069	5.058	0.02083
metab_148	pos_15110	pos	44.8	5.647	0.04695	6.069	0.02481
metab_459	pos_4716	pos	54.9	5.253	0.03032	5.671	0.01051
metab_157	pos_16046	pos	48.8	5.783	0.006398	5.366	0.0351
metab_189	neg_2459	neg	54.2	6.153	0.01993	5.739	0.005458
metab_171	neg_650	neg	52.4	4.705	0.01041	5.123	0.04409
metab_421	pos_4322	pos	48.5	6.705	0.01236	7.119	0.01421

metab_198	neg_3387	neg	47.5	6.311	0.01792	5.897	0.01031
metab_194	neg_3043	neg	50.4	6.29	0.02138	5.876	0.01088
metab_122	pos_1267	pos	44.4	5.526	0.05364	5.103	0.04918
metab_180	neg_1563	neg	50.9	5.365	0.11	5.799	0.01431
metab_176	neg_1234	neg	46.8	5.716	0.02606	5.302	0.01696
metab_189	neg_2541	neg	47.2	6.281	0.01153	6.692	0.0126
metab_196	neg_3228	neg	49.4	5.077	0.08373	5.501	0.02224
metab_207	neg_4300	neg	46.4	5.669	0.02503	5.258	0.0179
metab_188	neg_2358	neg	48.6	5.753	0.03701	6.165	0.009728
metab_187	neg_2269	neg	47.7	5.012	0.03499	4.599	0.03285
metab_201	neg_3729	neg	45.5	4.973	0.449	5.553	0.06329
metab_223	neg_5934	neg	55.2	5.439	0.01925	5.849	0.01657
metab_222	neg_5889	neg	55.8	5.398	0.01846	4.989	0.01907
metab_192	neg_2783	neg	55.8	5.32	0.08133	5.741	0.02037
metab_234	neg_7112	neg	47.5	5.599	0.01271	6.008	0.02606
metab_196	neg_3208	neg	52.1	5.912	0.0175	5.502	0.03302
metab_196	neg_3171	neg	47.4	4.566	0.01041	4.974	0.01464
metab_228	neg_6428	neg	42.8	5.794	0.02065	5.381	0.05554
metab_299	pos_3098	pos	53.9	6.647	0.01383	7.054	0.02454
metab_196	neg_3224	neg	46.1	6.458	0.009578	6.052	0.008506
metab_226	neg_6271	neg	52.3	5.009	0.04893	4.597	0.03073
metab_180	neg_1634	neg	51	5.466	0.07811	5.049	0.004901
metab_193	neg_2894	neg	52.1	4.977	0.09771	5.398	0.02675
metab_197	neg_3284	neg	45.2	6.219	0.01276	5.814	0.03422
metab_220	neg_5643	neg	52	5.264	0.02577	4.858	0.03482
metab_669	pos_6853	pos	45.5	5.277	0.04921	4.863	0.06408
metab_132	pos_13433	pos	54.2	4.844	0.02679	4.432	0.06925
metab_612	pos_6270	pos	52.9	4.672	0.06942	5.084	0.02365
metab_232	neg_6830	neg	48.1	5.866	0.0421	6.271	0.01999
metab_604	pos_6192	pos	42.2	5.388	0.0462	5.793	0.01915
metab_188	neg_2436	neg	45.1	5.277	0.03747	4.873	0.02552
metab_511	pos_5245	pos	45.7	6.322	0.01937	6.725	0.03363
metab_227	neg_6407	neg	57.8	6.072	0.01218	5.67	0.01848
metab_197	neg_3278	neg	45.3	6.08	0.01587	5.679	0.01692
metab_210	neg_4670	neg	52.3	5.107	0.0982	5.526	0.02724
metab_215	neg_5140	neg	46.6	5.936	0.01186	5.535	0.02327
metab_109	pos_1134	pos	45.6	6.105	0.007428	6.504	0.005596
metab_171	neg_629	neg	45.8	4.773	0.1153	5.203	0.07339

metab_198	neg_3405	neg	45.6	5.091	0.03737	4.689	0.02691
metab_183	neg_1923	neg	48.6	5.409	0.03049	4.999	0.0731
metab_126	pos_12873	pos	49	5.366	0.02489	4.965	0.0343
metab_229	pos_2377	pos	42.6	5.856	0.02261	6.257	0.04075
metab_191	neg_2654	neg	48.6	6.3	0.01083	5.902	0.0212
metab_206	neg_4217	neg	45.1	6.559	0.01227	6.163	0.009477
metab_194	neg_2980	neg	52.1	5.11	0.03921	4.71	0.01999
metab_217	neg_5352	neg	53.8	5.107	0.02936	4.709	0.02567
metab_133	pos_13551	pos	47.5	6.19	0.01629	6.584	0.02369
metab_211	neg_4715	neg	45.4	5.637	0.05842	5.234	0.03527
metab_985	pos_1021	pos	50.5	6.009	0.01074	5.616	0.01298
metab_408	pos_4192	pos	56.3	5.694	0.02073	5.3	0.01453
metab_223	neg_5990	neg	48	5.589	0.08543	5.996	0.02905
metab_996	pos_1032	pos	47	5.776	0.01304	5.383	0.01429
metab_225	neg_6118	neg	54	6.049	0.01946	6.442	0.01673
metab_221	neg_5704	neg	53.6	6.637	0.01568	6.245	0.009204
metab_168	neg_352	neg	50.3	5.815	0.01721	5.422	0.01914
metab_233	neg_7032	neg	48.9	5.914	0.02066	5.52	0.02904
metab_153	pos_15594	pos	51.2	6.334	0.009237	6.725	0.01212
metab_206	neg_4198	neg	47.7	6.02	0.01587	5.629	0.0172
metab_231	neg_6813	neg	45.8	6.766	0.009646	6.376	0.009178
metab_214	neg_4987	neg	48	5.129	0.03896	4.733	0.03864
metab_117	pos_11914	pos	44.8	5.188	0.073	5.59	0.03789
metab_238	pos_2468	pos	49.2	5.817	0.02777	6.208	0.009267
metab_168	neg_401	neg	48.1	5.371	0.05449	4.969	0.05903
metab_117	pos_11927	pos	55.6	5.301	0.01842	5.691	0.02055
metab_578	pos_5930	pos	42.9	6.028	0.0245	6.419	0.0202
metab_211	neg_4691	neg	45.5	5.758	0.03655	5.366	0.01569
metab_226	neg_6280	neg	46.5	5.508	0.07681	5.909	0.02406
metab_171	neg_665	neg	47.1	5.126	0.01041	5.514	0.01698
metab_158	pos_16167	pos	50.3	5.122	0.03978	5.515	0.03471
metab_355	pos_3656	pos	55.4	6.027	0.01824	5.639	0.01123
metab_205	neg_4123	neg	49.4	5.706	0.0169	5.317	0.02393
metab_231	neg_6734	neg	53	5.114	0.01041	5.503	0.03179
metab_210	neg_4626	neg	47.3	5.584	0.02537	5.197	0.00781
metab_167	neg_233	neg	47.1	5.97	0.01847	5.584	0.01167
metab_131	pos_13382	pos	52.2	5.863	0.01612	5.475	0.03439
metab_184	neg_2025	neg	45.8	6.459	0.02425	6.073	0.0189

metab_170	neg_547	neg	47.2	5.16	0.1118	5.57	0.03845
metab_220	neg_5615	neg	53.8	5.962	0.02011	5.577	0.01096
metab_230	neg_6630	neg	50.6	5.33	0.01843	4.943	0.03098
metab_972	pos_9888	pos	50.6	4.838	0.02265	4.44	0.08165
metab_161	pos_16417	pos	30	4.984	0.02717	5.371	0.02848
metab_203	neg_3963	neg	47.6	4.578	0.1414	4.996	0.01772
metab_224	neg_6079	neg	49.9	5.564	0.01041	5.947	0.0209
metab_411	pos_4227	pos	42.8	6.271	0.007668	5.889	0.01609
metab_328	pos_3392	pos	46.3	5.499	0.03859	5.885	0.02799
metab_145	pos_14766	pos	45.6	5.105	0.02745	4.722	0.02676
metab_206	neg_4181	neg	45.4	5.299	0.04978	4.913	0.01072
metab_124	pos_1282	pos	40.6	5.857	0.05835	6.248	0.03889
metab_121	pos_12336	pos	55.5	4.964	0.01651	4.583	0.01859
metab_205	neg_4106	neg	46.7	5.667	0.03123	5.284	0.02587
metab_100	pos_10251	pos	46.7	4.804	0.08415	5.2	0.03148
metab_193	neg_2925	neg	49.6	6.129	0.01821	5.749	0.01353
metab_205	neg_4122	neg	47.2	5.543	0.04748	5.926	0.0167
metab_198	neg_3390	neg	47.1	5.873	0.02496	5.493	0.02092
metab_204	neg_4026	neg	46.4	5.78	0.02617	5.4	0.01673
metab_204	neg_4044	neg	43.3	6.063	0.03167	5.683	0.01961
metab_102	pos_10383	pos	49.4	4.039	0.04317	4.42	0.01181
metab_208	neg_4454	neg	54.6	6.031	0.02724	5.653	0.01446
metab_836	pos_862	pos	48.8	6.245	0.009817	5.868	0.01697
metab_168	neg_314	neg	47.9	5.374	0.02455	5.752	0.02121
metab_154	pos_15763	pos	52.7	7.042	0.004058	6.666	0.01052
metab_182	neg_1814	neg	46.7	6.148	0.02016	6.524	0.009015
metab_231	neg_6757	neg	45.9	6.015	0.01317	5.639	0.01314
metab_225	neg_6155	neg	49.1	5.922	0.01103	5.546	0.02098
metab_194	pos_2026	pos	47.4	6.75	0.03151	7.127	0.00927
metab_213	neg_4904	neg	51.3	6.351	0.01357	6.724	0.009863
metab_167	neg_285	neg	46.2	5.678	0.04477	5.299	0.03087
